# DSB structure impacts DNA recombination leading to class switching and chromosomal translocations in human B cells

**DOI:** 10.1371/journal.pgen.1008101

**Published:** 2019-04-04

**Authors:** Clare C. So, Alberto Martin

**Affiliations:** Department of Immunology, University of Toronto, Toronto, Ontario, Canada; University of Washington School of Medicine, UNITED STATES

## Abstract

Class switch recombination (CSR) requires activation-induced cytidine deaminase (AID) to trigger DNA double strand breaks (DSBs) at the immunoglobulin heavy chain (*IGH)* in B cells. Joining of AID-dependent DSBs within *IGH* facilitate CSR and effective humoral immunity, but ligation to DSBs in non-*IGH* chromosomes leads to chromosomal translocations. Thus, the mechanism by which AID-dependent DSBs are repaired requires careful examination. The random activity of AID in *IGH* leads to a spectrum of DSB structures. In this report, we investigated how DSB structure impacts end-joining leading to CSR and chromosomal translocations in human B cells, for which models of CSR are inefficient and not readily available. Using CRISPR/Cas9 to model AID-dependent DSBs in *IGH* and non-*IGH* genes, we found that DSBs with 5’ and 3’ overhangs led to increased processing during end-joining compared to blunt DSBs. We observed that 5’ overhangs were removed and 3’ overhangs were filled in at recombination junctions, suggesting that different subsets of enzymes are required for repair based on DSB polarity. Surprisingly, while Cas9-mediated switching preferentially utilized NHEJ regardless of DSB structure, A-EJ strongly preferred repairing blunt DSBs leading to translocations in the absence of NHEJ. We found that DSB polarity influenced frequency of Cas9-mediated switching and translocations more than overhang length. Lastly, recombination junctions from staggered DSBs exhibited templated insertions, suggesting iterative resection and filling in during repair. Our results demonstrate that DSB structure biases repair towards NHEJ or A-EJ to complete recombination leading to CSR and translocations, thus helping to elucidate the mechanism of genome rearrangements in human B cells.

## Author summary

The production of different classes of antibodies/immunoglobulins (IgM, IgG, etc.) is essential for protection against diverse pathogens and effective immunity. This cellular process is triggered by the enzyme activation-induced cytidine deaminase (AID). AID mutates DNA predominantly in antibody genes, generating different types of DNA breaks. Repair of DNA breaks initiated by AID leads to the production of different antibody classes. Erroneous repair of this damage can also lead to chromosomal translocations, a hallmark of lymphomas and other cancers. In this study, we used CRISPR/Cas9 technology to model the different types of DNA breaks physiologically produced by AID. We found that the specific structure of these DNA breaks strongly influenced how they were repaired. That is, different types of DNA breaks inform different modes of rejoining. Our findings show that not all types of DNA breaks are treated equally by genome maintenance machinery in the cell. These observations provide insight into the molecular mechanisms behind antibody-dependent immunity and lymphomagenesis.

## Introduction

DNA recombination at the immunoglobulin heavy chain (*IGH*) locus is required for class switch recombination (CSR), the process that changes the class of immunoglobulin expressed by B cells, e.g., from IgM to IgG or IgA, etc. CSR increases diversity of immunoglobulin isotypes, which is necessary for comprehensive humoral immunity. Recombination at the *IGH* locus is initiated by activation-induced cytidine deaminase (AID) [1], which deaminates deoxycytidine to deoxyuridine mostly within WRC (W = A/T, R = A/G) motifs in *IGH* switch regions. These switch regions comprise 3–4 kilobases of non-coding DNA enriched for WRC motifs and are positioned upstream of each of the constant regions that encode immunoglobulin isotypes. Removal of deoxyuridines by the base-excision and mismatch repair pathways results in a DNA single strand break, or nick [2]. AID-dependent nicks on opposite DNA strands can melt into a DNA double strand break (DSB). Ligation of DSBs in donor and acceptor switch regions places the acceptor constant region downstream of the rearranged VDJ gene segment. Expression of the recombined VDJ-acceptor constant region sequence gives rise to an immunoglobulin of a new isotype.

Most AID-dependent DSBs at the *IGH* locus are either repaired faithfully with no effect on B cell receptor expression or in a productive manner leading to CSR. However, AID-dependent DSBs in the *IGH* locus can sometimes ligate to DSBs in other chromosomes, leading to chromosomal translocations that are hallmarks of B cell lymphomas [3–6]. Thus, characterizing the molecular mechanism of how AID-dependent DSBs are repaired within or between chromosomes will help explain the basis of antibody diversification and lymphomagenesis.

DSB repair during CSR primarily relies on canonical non-homologous end-joining (NHEJ) [7] and, to a lesser extent, alternative end-joining (A-EJ) [8,9]. NHEJ and A-EJ are largely distinguished by the extent of mutagenicity associated with repair. Broadly speaking, NHEJ involves the ligation of blunt or nearly blunt DSB ends with little to no insertions/deletions (indels). In contrast, A-EJ is characterized by exonuclease-driven deletions from the DSB end, known as resection, to expose short tracts of homologous nucleotides between non-homologous sequences, known as microhomology, which are used to join DSB ends [10]. Evidence of repair by A-EJ during CSR and other instances of DSB repair in mouse B cells has been best characterized when NHEJ is defective or suboptimal [9,11].

CSR is an attractive model for studying DSB repair and identifying NHEJ factors because it is a physiological, programmed process that is easily studied in mouse models. Primary mouse B cells undergo CSR *ex vivo* in response to lipopolysaccharide and cytokines while the CH12 mouse B cell line robustly undergoes CSR *in vitro* upon cytokine stimulation [12]. However, while overall CSR frequency is affected by mutation of putative NHEJ factors, the nature of the switch regions are not conducive to assessing the detailed role of these same factors. This is because AID can potentially act on thousands of deoxycytidines on both DNA strands in the switch regions, resulting in a mix of DSB structures, such as blunt ends or protruding 5’ or 3’ overhangs of varying length. Different factors or end-joining pathways might be needed to rejoin different types of ends. Furthermore, the high density of WRC motifs in the switch regions together with the seemingly random activity of AID make it difficult to determine the precise points where nicks and rejoining occurred. We have previously described an experimental system to work around these uncertainties. We used CRISPR/Cas9 and combinations of single guide RNAs (sgRNAs) to make predetermined DSBs similar to those that are expected from AID-mediated nicks to characterize DSB formation and repair in the CH12 mouse B cell line [13].

However, much less is understood about joining AID-dependent DSBs in human B cells, mainly due to limited experimental material. The CL-01 human B cell line can undergo CSR *in vitro* upon cytokine stimulation but at frequencies far below that of CH12 mouse B cells [14]. B cell subsets from human tonsils can also express AID and undergo CSR *ex vivo* upon cytokine stimulation [15], but these uncloned cells are difficult to experimentally manipulate and are not suitable for detailed biochemical studies.

In this study, we have induced class switching and chromosomal translocations in the BJAB human B cell line by creating predetermined DSB intermediates using CRISPR/Cas9. These DSBs emulate DSB structures expected from physiological AID deamination, allowing us to assess how DSBs of known structure are ultimately repaired. Thus, we are able to analyze the mechanism of DSB repair leading to genome rearrangements in human B cells with the same precision as in mouse B cells. By comparing how different DSB structures are repaired by human B cells leading to switching or translocations, we found that DSB structure can bias repair of distal DSBs towards NHEJ and A-EJ in human B cells.

## Results

### Cas9-mediated DSBs induce class switching from IgM to IgA in human B cells

To characterize end-joining of distal, non-homologous loci in human B cells, we used the BJAB human B cell line. BJAB cells are Burkitt’s lymphoma-derived cells that express surface IgM but do not harbor the *IGH-MYC* translocation typical of Burkitt’s lymphoma [16]. These properties allowed us to target the native *IGH* locus in BJAB cells with Cas9 and induce *de novo* genetic rearrangements within chromosomes (i.e. class switching from IgM to IgA) and between chromosomes (i.e. *IGH-BCL6* chromosomal translocations).

**Fig 1:**
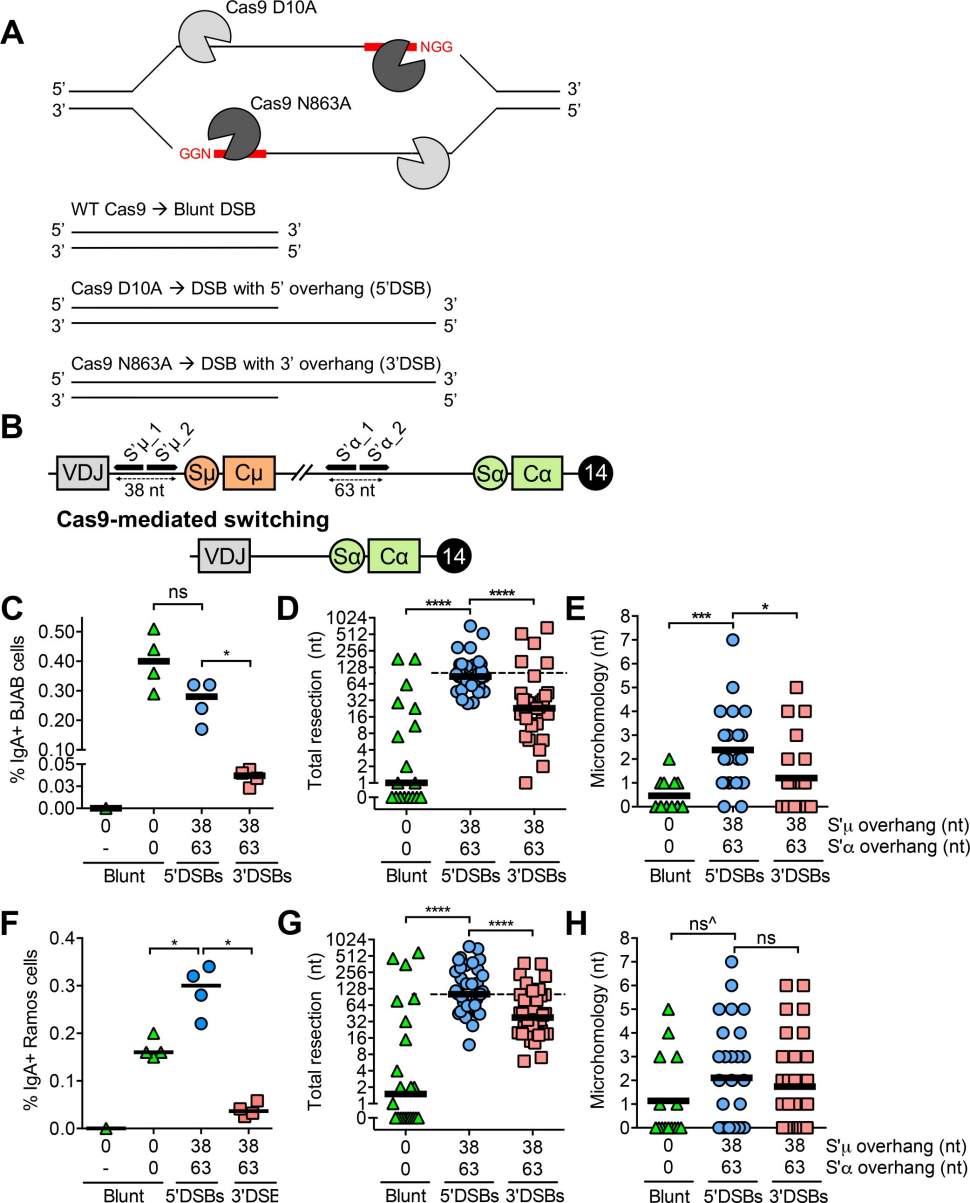
Cas9-mediated switching in human B cells. **(A)** Schematic depicting Cas9 variants and orientation of sgRNAs required to generate blunt, 5’, and 3’DSBs. One sgRNA and wild-type Cas9 produces a blunt DSB, while paired sgRNAs and Cas9 D10A or Cas9 N863A give rise to 5’ or 3’DSBs, respectively. **(B)** Schematic depicting sgRNA target regions in the immunoglobulin heavy chain (*IGH*) locus on human chromosome 14 in normal BJAB cells and the resulting chromosome after Cas9-mediated switching. The sgRNAs in this experiment target S’μ, a non-repetitive region between the rearranged VDJ gene segment and the switch μ (Sμ) region. Cleavage at the S’μ_1 sgRNA target site with wild-type Cas9 produces a blunt DSB, while cleavage with the Cas9 nickases and sgRNAs S’μ_1 and _2 produces a staggered DSB with a 38 nt overhang. A similar strategy is applied using sgRNAs targeting S’α to produce blunt DSBs or staggered DSBs with a 63 nt overhang. Schematic is not to scale. **(C)** Percentage of IgA+ BJAB cells from blunt, 5’, and 3’DSBs measured by flow cytometry, 5 days post-transfection with Cas9/sgRNA plasmids. “-”, mock transfection. **(D)** Total resection of S’μ and S’α at S’μ-S’α junctions from blunt, 5’, and 3’DSBs. Dotted line denotes the total overhang length in S’μ plus S’α (101 nt). **(E)** Microhomology usage at S’μ-S’α junctions from blunt, 5’, and 3’DSBs. Black line denotes mean microhomology usage. **(F)** Percentage of IgA+ Ramos cells from blunt, 5’, and 3’DSBs measured by flow cytometry, 3 days post-transfection with Cas9/sgRNA plasmids. “-”, mock transfection. **(G)** Total resection of S’μ and S’α at S’μ-S’α junctions from blunt, 5’, and 3’DSBs in Ramos cells. Dotted line denotes the total overhang length in S’μ plus S’α (101 nt). **(H)** Microhomology usage at S’μ-S’α junctions from blunt, 5’, and 3’DSBs in Ramos cells. Black line denotes mean microhomology usage. ^The vast majority of S’μ-S’α junctions from blunt DSBs that were sequenced exhibited 0 nt of resection and 0 nt of microhomology, but had to be excluded from analysis for being non-unique.

Throughout this study, we generated DSBs with varied end structures at AID target loci using Cas9 and single guide RNAs (sgRNAs) (Fig 1A). We first tested whether we could induce Cas9-mediated switching in BJAB cells with blunt DSBs, as we had previously done in mouse B cell lines [13]. To do so, we transiently transfected BJAB cells with two plasmids encoding Cas9 and sgRNAs targeting either the region upstream of switch region μ (S’μ) or α (S’α) to create DSBs (Fig 1A and 1B, S1 Table). We chose to target these regions in order to avoid the repetitive sequences present in the switch regions. Recombination between DSBs in S’μ and S’α replaces constant region μ (Cμ) with constant region α (Cα) downstream of the rearranged VDJ gene segment, allowing for surface IgA expression (Fig 1B). Cells were harvested 5 days post-transfection for flow cytometric detection of surface IgA (Fig 1C, S1 Fig). While a single blunt DSB in S’μ was not able to induce Cas9-mediated switching, blunt DSBs in both S’μ and S’α were able to induce Cas9-mediated switching in BJAB cells (Fig 1C). Therefore, CRISPR/Cas9 is a suitable method of inducing DSBs at the native *IGH* locus leading to class switching.

The majority of AID-dependent DSBs are staggered formed by nicks on opposite DNA strands [17]. Therefore, we next measured whether staggered DSBs in human B cells could also trigger Cas9-mediated switching. To produce staggered DSBs in BJAB cells, we used the Cas9 nickases, whereby a D10A or N863A substitution inactivate the Cas9 RuvC or HNH nuclease domains, respectively [18]. Expression of the Cas9 D10A nickase with a pair of sgRNAs produces a staggered DSB with a 5’ single-stranded DNA overhang (hereafter termed a 5’DSB) (Fig 1A). Applying the same strategy with the same sgRNAs in combination with the Cas9 N863A nickase gives rise to a 3’DSB (Fig 1A). Therefore, the position and overhang length of a particular DSB can be predefined using particular sgRNA combinations (S2 Table). By transfecting BJAB cells with four plasmids encoding either the Cas9 D10A or N863A nickases and each of four sgRNAs, we produced 5’ and 3’ overhangs of 38 nucleotides in S’μ and 63 nucleotides in S’α (Fig 1B). 5’DSBs with a 38 nt overhang in S’μ and a 63 nt overhang in S’α were able to induce Cas9-mediated switching at a frequency slightly lower than that of blunt DSBs, whereas 3’DSBs with identical overhang lengths were significantly poorer substrates (Fig 1C, S1 Fig). These results suggest that 5’DSBs are better substrates for Cas9-mediated switching than 3’DSBs in human B cells (see Discussion for alternative possibilities).

To assess whether these staggered DSB overhang lengths resembled those triggered by AID *in vivo*, we determined how far apart AID-mediated nicks are typically spaced during CSR. To do so, we analyzed AID-mediated mutations at Sμ from Ung^-/-^Msh2^-/-^ mice [2]. Mice that are doubly deficient in UNG and MSH2 are unable to excise AID-mediated uridines or repair dU:dG mismatches, respectively. Replication across these mismatches leads to mutations, >99% of which are transition mutations [2]. Thus, the distance between dC→dT and dG→dA transition mutations serves as a proxy for the distance between deaminated dCs on opposite strands. We found that the distance between dC→dT on opposite strands at Sμ from Ung^-/-^Msh2^-/-^ mice ranged from 0 to 360 nt, with an average of 38 nt and a median of 27 nt (S2 Fig, S3 Table). This result suggests that the lengths of staggered DSB overhangs we designed in the IGH locus (38 nt in S'μ and 63 nt in S'α) are within the expected physiological range of AID-mediated staggered DSBs.

It is important to note, however, that our analysis might underestimate the true distance between AID-mediated nicks because the transition mutations included in our analysis were unlikely to simultaneously arise or persist within a defined time-frame. At the same time, this analysis of AID-dependent mutations might overestimate the true distance between AID-mediate nicks as it was conducted 5’ of switch region μ, which contains less WRC motifs than the proper switch region μ. Therefore, although we cannot conclude with absolute certainty that the distance between transition mutations in the switch regions exactly represents dC deamination density, it seems that the overhang lengths we chose in this study are at least within what is caused by AID in mouse B cells.

We next analyzed S’μ-S’α junctions from blunt, 5’, and 3’DSBs to examine the footprint of end-joining pathways used for repair. To this end, we sequenced S’μ-S’α junctions from transfected cells and quantified the amount of resection and microhomology usage at S’μ-S’α junctions to discern repair by NHEJ and A-EJ. We found that blunt DSBs led to S’μ-S’α junctions with low amounts of resection (Fig 1D, S1 Appendix) and microhomology usage (Fig 1E), consistent with previous findings that blunt DSBs are primarily substrates for NHEJ [19]. In contrast, 5’DSBs led to S’μ-S’α junctions with more resection (Fig 1D) and microhomology usage (Fig 1E) than blunt or 3’DSBs.

Interestingly, we observed that the median amount of 5’DSB resection coincided with the sum length of S’μ and S’α overhangs, while the median amount of 3’DSB resection fell below this sum length (Fig 1D, dotted line). This pattern was also evident by visualizing individual resection of S’μ versus S’α overhangs from each unique junction (S3 Fig). This result suggests that 5’ overhangs tend to be removed prior to end-joining, while 3’ overhangs tend to be filled in.

To determine whether the S’μ-S’α end-joining patterns we observed in BJAB cells were a general property of human B cells, we induced Cas9-mediated switching in Ramos cells, another human B cell line. Blunt and 5’DSBs led to a greater frequency of Cas9-mediated switching to IgA in Ramos cells than 3’DSBs (Fig 1F). Like we observed in BJAB cells, 5’DSBs led to S’μ-S’α junctions in Ramos cells with increased resection compared to those from blunt or 3’DSBs (Fig 1G). In Ramos cells, like BJAB cells, 5’DSBs tended to be resected to the sum total overhang length in S’μ and S’α, while resection of 3’DSBs fell below this sum total (Fig 1G, dotted line), supporting the model that 5’DSBs are resected while 3’DSBs are filled in to complete end-joining. Interestingly, we observed that 5’DSBs led to increased microhomology usage at S’μ-S’α junctions compared to those from blunt DSBs but not 3’DSBs (Fig 1H).

Together, our results suggest that in human B cells, 5’ and 3’DSBs lead to more processing during end-joining than blunt DSBs to complete Cas9-mediated switching. Our results suggest that 5’DSBs lead to increased resection and increased microhomology typically associated with A-EJ, while 3’DSBs may be processed by a DNA polymerase prior to end-joining.

### DSB polarity influences end-joining properties more than sequence context

Previous reports have suggested that sequence context can influence Cas9 repair outcomes [20,21]. One study found that sgRNAs that target the same sequence at multiple sites within the genome lead to similar Cas9-mediated indel profiles [20]. Another recent study found that a thymine in the fourth position after the protospacer adjacent motif (PAM) was more predictive of single nucleotide indels than a guanine in the same position [21], suggesting that sequence might play a role in dictating end-joining characteristics. The mechanism behind this potential bias, however, remains unclear.

To determine whether the S’μ-S’α end-joining patterns we observed were due to the nature of the sequence targeted by Cas9 rather than DSB polarity, we designed sgRNAs targeting a slightly different region upstream of S’α, approximately 150 nt downstream of the original sgRNA pair (S4 Fig). Using this new pair of sgRNAs targeting S’α (S2 Table), we generated blunt, 5’, and 3’DSBs at S’μ and S’α to induce Cas9-mediated switching. Despite the different sequence targeted in S’α, we observed similar end-joining patterns in BJAB cells based on DSB structure. That is, 5’DSBs led to S’μ-S’α junctions with increased resection (S4 Fig) and microhomology (S4 Fig) than 3’DSBs. Again, we observed that the median amount of resection of 5’DSBs approached the sum total overhang length, while 3’DSBs tended to be resected below this amount (S4 Fig). These results support the notion that DSB polarity triggers different modes of end processing.

Although other studies have identified a role for sequence context in influencing DSB repair outcomes, we did not find evidence that simply changing sequence context significantly affected resection or microhomology at Cas9-mediated S’μ-S’α junctions. Rather, the polarity of a DSB within a given sequence had a more pronounced influence on end-joining properties.

### Cas9-induced 5’DSBs are preferential substrates for *IGH-BCL6* (der3) translocations

**Fig 2:**
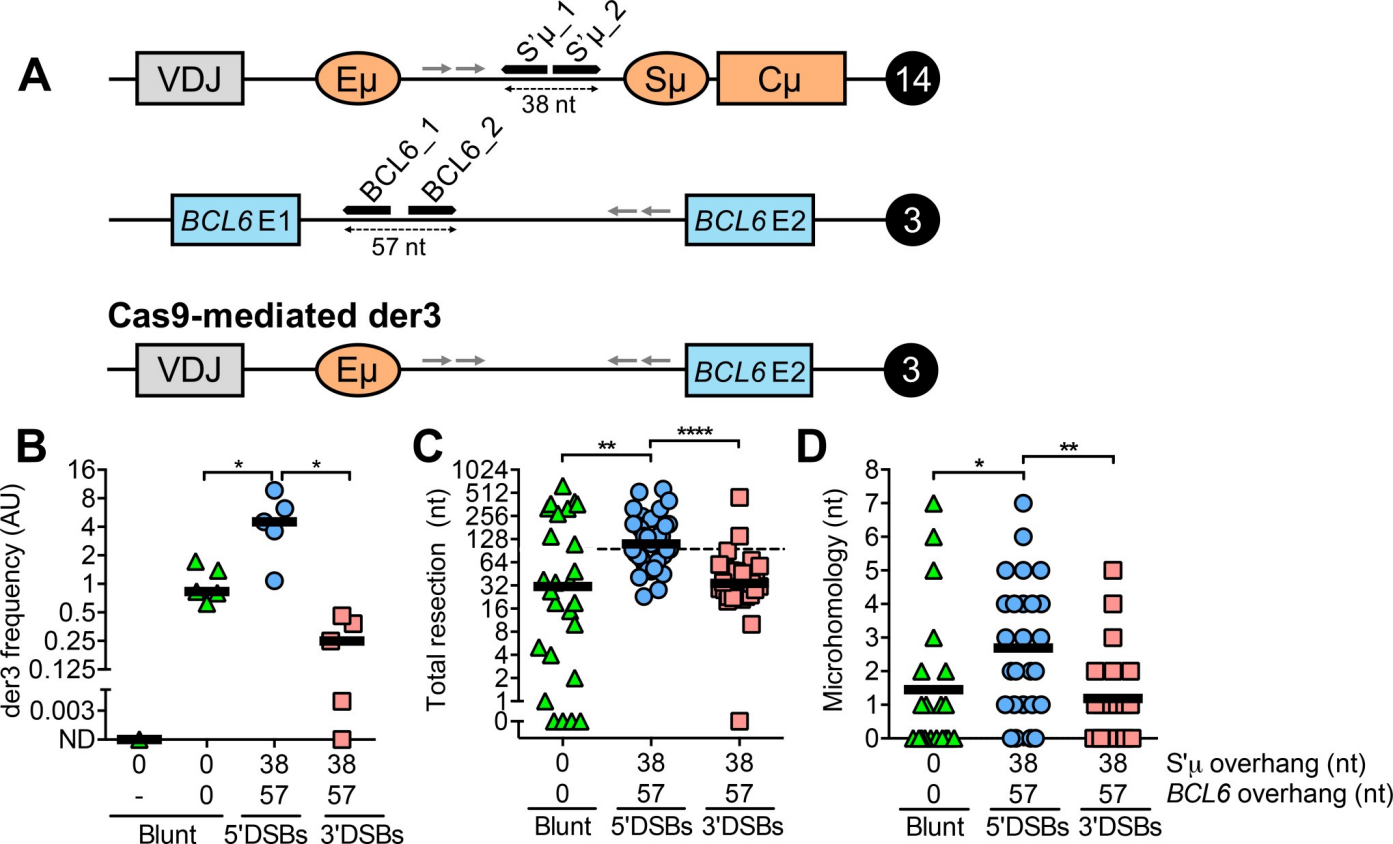
Cas9-mediated *IGH-BCL6* translocations (der3) in human B cells. **(A)** Schematic depicting sgRNA target regions in *IGH* on human chromosome 14 and *BCL6* on human chromosome 3. Primer binding sites are denoted by arrows and their sequences are presented in S1 Table. Cleavage at the S’μ_1 sgRNA target site with wild-type Cas9 produces a blunt DSB, while cleavage with the Cas9 nickases and sgRNAs S’μ_1 and _2 produces a staggered DSB with a 38 nt overhang. A similar strategy is applied using sgRNAs targeting *BCL6* to produce blunt DSBs or staggered DSBs with a 57 nt overhang. Recombination between sgRNA sites gives rise to derivative chromosome 3 (der3). Schematic is not to scale. **(B)** Frequency of Cas9-mediated der3 from blunt, 5’, and 3’DSBs measured by qPCR. Cells were harvested 5 days post-transfection with Cas9/sgRNA plasmids for genomic DNA isolation. “-”, mock transfection. **(C)** Total resection of S’μ and *BCL6* at S’μ-*BCL6* junctions from blunt, 5’, and 3’DSBs. Dotted line denotes the total overhang length in S’μ plus *BCL6* (95 nt). **(D)** Microhomology usage at S’μ-*BCL6* junctions from blunt, 5’, and 3’DSBs. Black line denotes mean microhomology usage.

AID activity can potentially lead to chromosomal translocations between *IGH* and so-called “off-target” genes. To determine whether DSB structure affects inter-chromosomal joining in human B cells, we designed sgRNAs targeting S’μ on chromosome 14 and *BCL6* on chromosome 3 (Fig 2A). Recombination between Cas9-induced DSBs at these sites results in the *IGH-BCL6* chromosomal translocation, hereafter termed “der3”, a marker of diffuse large B cell lymphoma and follicular lymphoma [22,23]. Cas9-mediated der3 was detected and quantified using a nested PCR assay. Briefly, approximately 45,000 genome equivalents (150 ng of human genomic DNA) were used for round 1 amplification of der3 by conventional PCR followed by round 2 amplification by quantitative PCR (qPCR). The amount of der3 in a given sample was normalized to the amount of a control genomic region on chromosome 13 amplified from total genomic DNA using a similar nested PCR assay.

By transiently transfecting plasmids encoding the relevant Cas9 enzymes and sgRNA combinations (S2 Table), we generated Cas9-induced der3 in BJAB cells with blunt, 5’, and 3’DSBs (S1 Fig). As we observed in Cas9-mediated switching, blunt and 5’DSBs were better substrates for Cas9-mediated der3 compared to 3’DSBs (Fig 2B, S1 Fig). Even though 3’DSBs led to the lowest frequency of Cas9-mediated translocations, we were able to generate enough translocations from 3’DSBs for S’μ-*BCL6* junction analysis with BJAB cells, which was previously not the case using CH12 mouse B cells [13].

To analyze the relative contributions of NHEJ and A-EJ to der3 formation, we sequenced S’μ-*BCL6* junctions from BJAB cells. We found that the pattern of resection and microhomology usage at S’μ-*BCL6* junctions was similar to what we observed at S’μ-S’α junctions. 5’DSBs led to S’μ-*BCL6* junctions with more resection (Fig 2C) and microhomology usage (Fig 2D) than blunt DSBs or 3’DSBs. We found that the median amount of resection for 5’DSBs was equivalent to the sum total overhang length in S’μ and *BCL6*, while the median amount of resection of 3’DSBs fell below the total overhang length (Fig 2D, dotted line, S3 Fig). This finding mirrors our observation at S’μ-S’α junctions that 5’ overhangs are likely removed prior to repair while 3’DSBs are filled in (Fig 1D, dotted line). Taken together, our data suggest that 5’ and 3’DSBs lead to more end processing than blunt DSBs leading to chromosomal translocations, with 5’DSBs leading to chromosomal translocation junctions exhibiting increased resection and microhomology usage.

### The role of NHEJ in Cas9-induced switching and der3 depends on DSB polarity in human B cells

**Fig 3:**
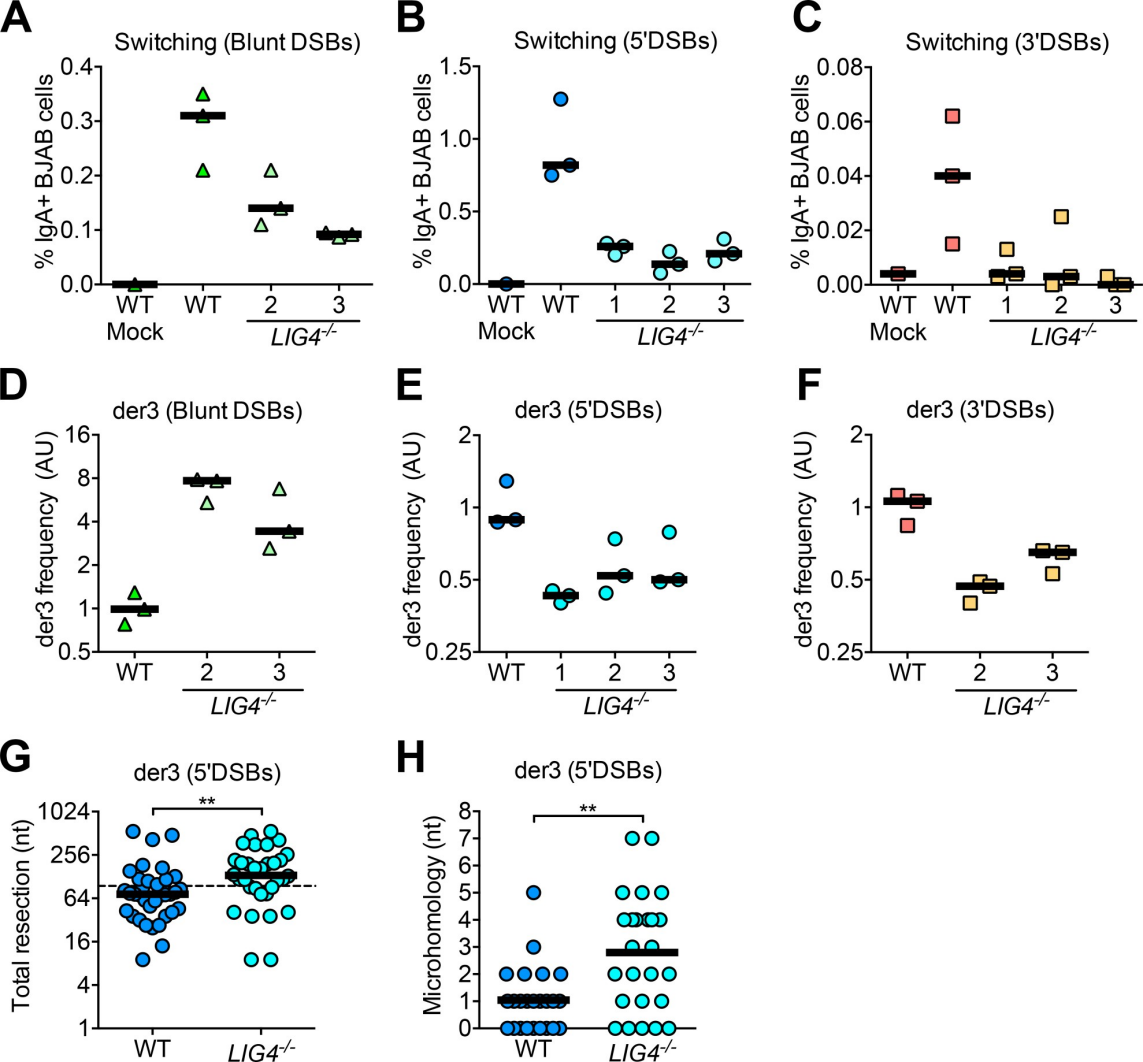
Effect of LIG4 deficiency on Cas9-mediated switching and der3 using blunt, 5’, and 3’DSBs in human B cells. **(A)** Percentage of IgA+ wild-type (WT) or *LIG4*^*-/-*^ BJAB cells using blunt, **(B)** 5’DSBs, or **(C)** 3’DSBs as measured by flow cytometry, 5 days post-transfection with Cas9/sgRNA plasmids. **(D)** Frequency of Cas9-mediated der3 in WT or *LIG4*^*-/-*^ BJAB cells using blunt, **(E)** 5’DSBs, or **(F)** 3’DSBs as measured by qPCR. Cells were harvested 5 days post-transfection with Cas9/sgRNA plasmids for genomic DNA isolation. **(G)** Total resection of S’μ and *BCL6* at der3 junctions from 5’DSBs generated in WT and *LIG4*^-/-^ BJAB cells. Dotted line denotes the total overhang length in S’μ plus *BCL6* (95 nt). **(H)** Microhomology usage at S’μ-*BCL6* junctions from 5’DSBs generated in WT and *LIG4*^-/-^ BJAB cells. Black line denotes mean microhomology usage.

CSR is thought to be largely driven by non-homologous end-joining (NHEJ) in human B cells. Patients with mutations or deficiencies in the NHEJ factors DNA ligase 4 (LIG4) and XRCC4-like factor (XLF) exhibit defects in CSR, as evidenced by low serum titers of IgG and IgA [24,25]. To confirm that Cas9-mediated switching recapitulated the requirement for NHEJ, we generated three LIG4-deficient BJAB clones using CRISPR/Cas9 gene editing (S5 Fig). We found that LIG4 deficiency reduced Cas9-mediated switching to IgA via blunt, 5’, and 3’DSBs (Fig 3A–3C). This result suggests that Cas9-mediated switching, like AID-mediated CSR, primarily relies on NHEJ.

We and others previously demonstrated that NHEJ deficiency leads to increased chromosomal translocations in mouse cells, suggesting that either NHEJ inhibits or A-EJ drives translocation in mouse cells [13,26–28]. Specifically, we showed that DNA ligase IV-deficiency led to a significant increase in Cas9-induced translocations from blunt and 5’DSBs in CH12 mouse B cells [13]. In contrast, it has been suggested that chromosomal translocations in human cells are NHEJ-dependent [29]. Ghezraoui et al illustrated that chromosomal translocations were significantly reduced in XRCC4- and LIG4-deficient human cells using 5’DSBs generated by zinc-finger nucleases, TALENs, and Cas9 D10A. The reason why A-EJ is more important for chromosomal translocations than CSR in mouse B cells remains unknown.

To test whether our model system recapitulates this finding, we measured Cas9-mediated der3 in LIG4-deficient BJAB cells. We found that *LIG4*^-/-^ BJAB cells had reduced Cas9-mediated der3 using either 5’ or 3’DSBs (Fig 3E and 3F). We also confirmed that resection and microhomology at S’μ-*BCL6* junctions from 5’DSBs were both increased in the absence of LIG4, as expected of repair by A-EJ (Fig 3G and 3H). Interestingly, we observed that Cas9-mediated der3 frequency was dramatically increased in *LIG4*^-/-^ BJAB cells using blunt DSBs (Fig 3D). The significance of this finding is considered in the Discussion. Taken together, our results suggest that the roles of NHEJ and A-EJ in rejoining chromosomal translocations in human B cells is dependent on DSB structure. These findings also support the notion that there are important differences in the molecular mechanism underlying chromosomal translocations between mouse and human B cells.

### Staggered DSB polarity influences Cas9-mediated switching more than overhang length

DSB intermediates leading to CSR presumably arise from AID-mediated nicks on opposite DNA strands that simultaneously persist. Since the switch regions are WRC motif-rich and AID can theoretically trigger nicks at any deoxycytidine within WRC motifs, the length of staggered DSB overhangs likely ranges greatly from one recombination event to the next. We previously demonstrated that nicks spaced 248 nt apart could induce Cas9-mediated switching in mouse B cells if oriented to produce a 5’DSB, but not a 3’DSB [13]. It is unknown whether these distal nicks require exonucleolytic processing to form a DSB.

**Fig 4:**
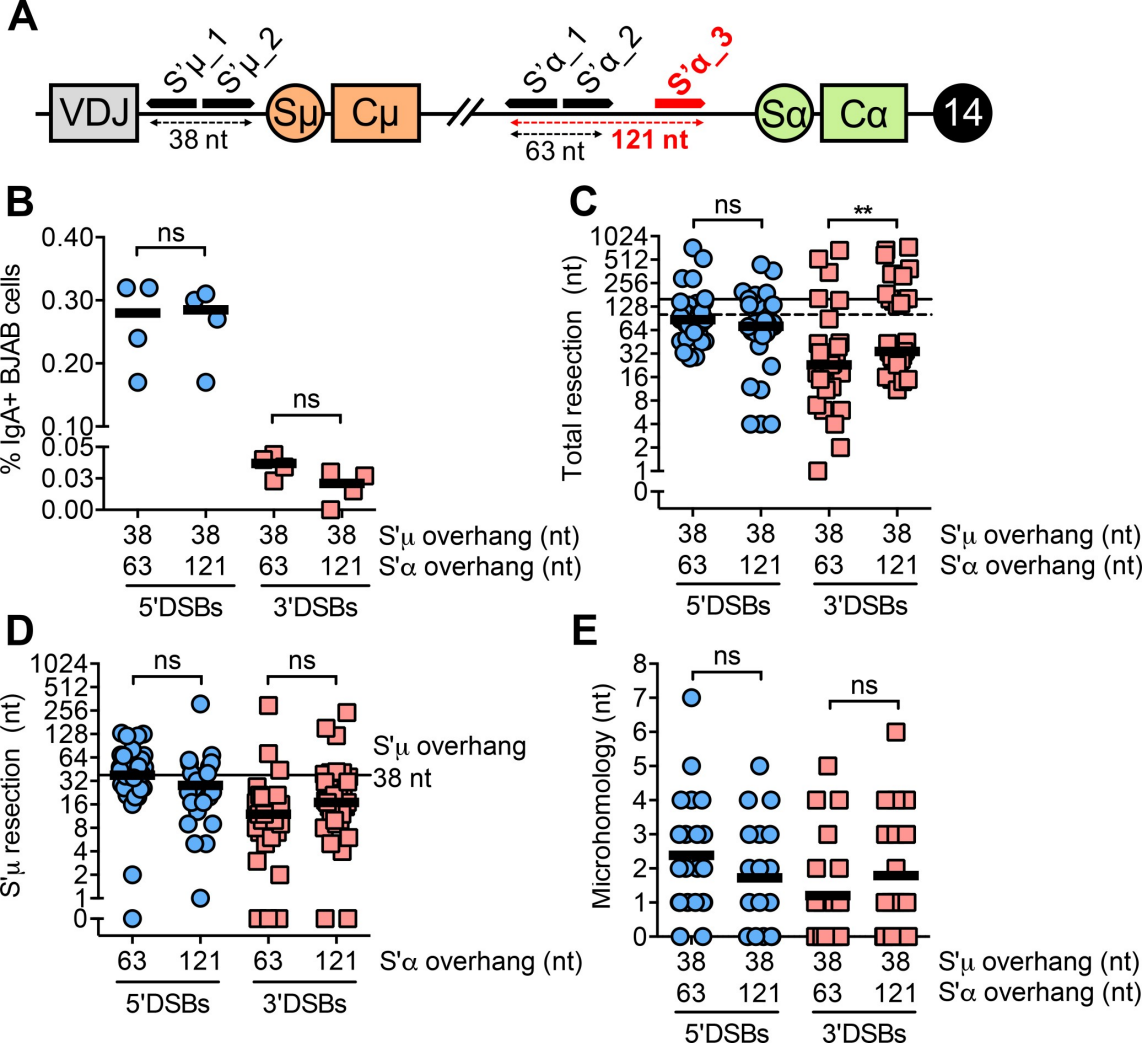
DSB polarity rather than overhang length primarily influences end-joining leading to Cas9-mediated switching. **(A)** Schematic depicting an additional sgRNA target site (shown in red) in S’α on the *IGH* locus on human chromosome 14. Cleavage by the Cas9 nickases at S’α_1 and S’α_2 or S’α_1 and S’α_3 gives rise to staggered DSBs with 63 or 121 nt overhangs, respectively. Schematic is not to scale. **(B)** Percentage of IgA+ BJAB cells from 5’ and 3’DSBs with either of two overhang lengths measured by flow cytometry, 5 days post-transfection with Cas9/sgRNA plasmids. Overhang lengths are denoted on the x-axis. **(C)** Total resection of S’μ and S’α at S’μ-S’α junctions from 5’ and 3’DSBs with either of two overhang lengths. Horizontal lines denote the total overhang lengths in S’μ plus S’α (dotted line = 101 nt, solid line = 159 nt). **(D)** Resection of 38 nt S’μ overhang at S’μ-S’α junctions when S’α overhang length is varied. **(E)** Microhomology usage at S’μ-S’α junctions from 5’ and 3’DSBs with either of two overhang lengths. Black line denotes mean microhomology usage.

We therefore tested whether increasing the distance between Cas9-mediated nicks, thereby producing 5’ and 3’DSBs with longer overhangs, affects the frequency and nature of Cas9-mediated switching. To address this, we used two pairs of sgRNAs targeting S’α to create 5’ and 3’DSBs with either 63 nt (short) or 121 nt (long) overhangs (Fig 4A). The overhang length in S’μ was kept constant at 38 nt. We did not observe changes in Cas9-mediated switching frequency when S’α overhangs were short or long (Fig 4B). Furthermore, 5’DSBs were better substrates for Cas9-mediated switching than 3’DSBs whether S’α overhangs were short or long (Fig 4B).

We next asked whether short or long overhangs affected resection and microhomology at S’μ-S’α junctions. We found that longer 5’ overhangs in S’α did not affect total resection at S’μ-S’α junctions, although longer 3’ overhangs in S’α did lead to a subtle increase in total resection (Fig 4C, S3 Fig). Importantly, we still observed that 5’DSBs were resected more than 3’DSBs, supporting the notion that 5’DSBs are resected while 3’DSBs are filled in, even when overhang length is increased. Longer overhangs in S’α did not stimulate increased resection of S’μ (Fig 4D), suggesting that ligation of staggered DSBs does not require symmetrical resection. We did not observe significant changes in microhomology at S’μ-S’α junctions whether 5’ or 3’DSB overhangs were short or long (Fig 4E). Altogether, these findings suggest that the polarity of the DSB influences Cas9-mediated switching frequency more than overhang length.

### Staggered DSB polarity influences Cas9-mediated der3 more than overhang length

**Fig 5:**
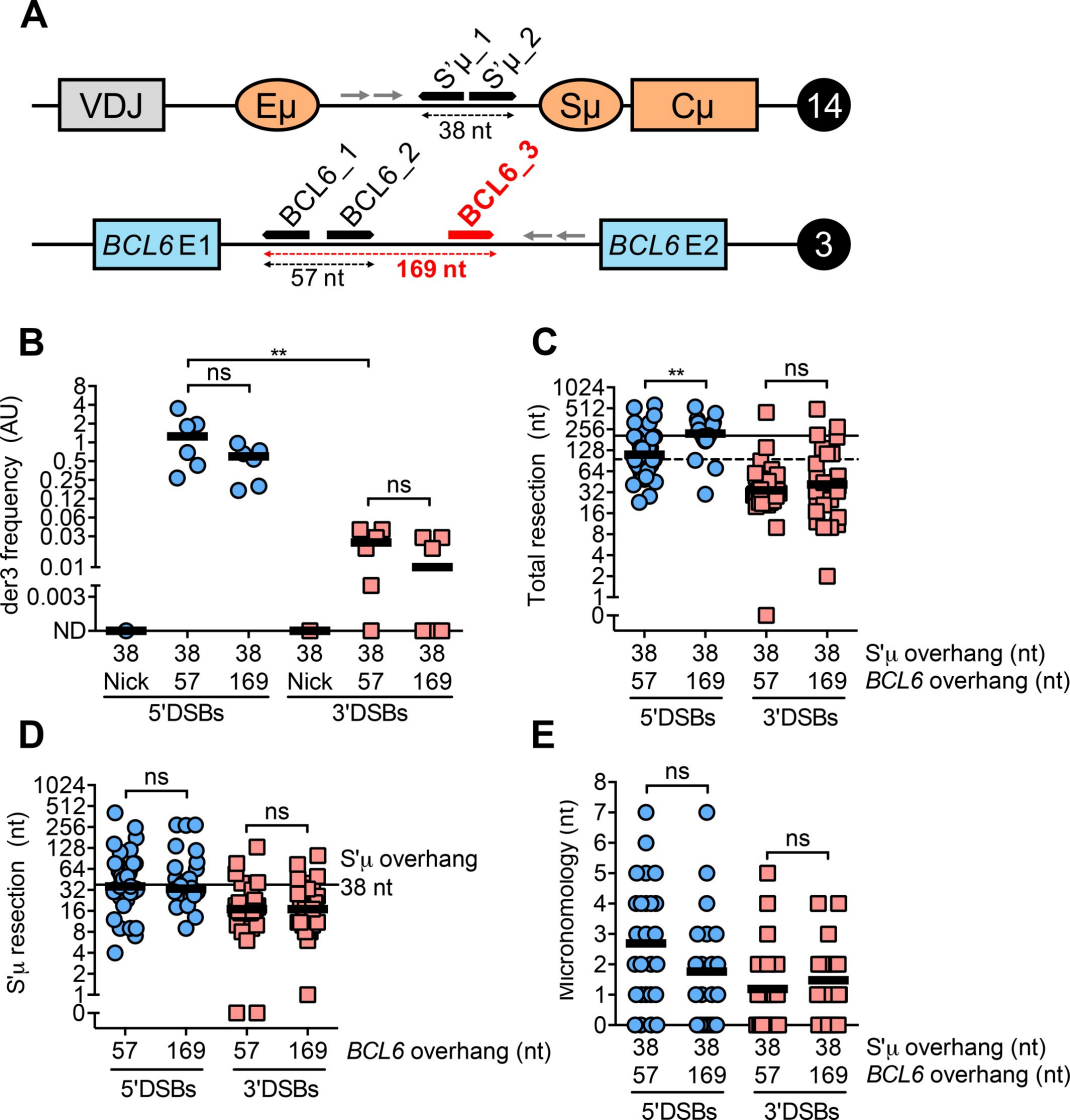
DSB polarity rather than overhang length primarily influences end-joining leading to Cas9-mediated der3. **(A)** Schematic depicting an additional sgRNA target site (shown in red) in the *BCL6* locus on human chromosome 3. Cleavage by the Cas9 nickases at BCL6_1 and BCL6_2 or BCL6_1 and BCL6_3 gives rise to staggered DSBs with 57 or 169 nt overhangs, respectively. Schematic is not to scale. **(B)** Frequency of Cas9-mediated der3 from 5’ and 3’DSBs with either of two overhang lengths measured by qPCR. Cells were harvested 5 days post-transfection with Cas9/sgRNA plasmids for genomic DNA isolation. Overhang lengths are denoted on the x-axis. **(C)** Total resection of S’μ and *BCL6* at S’μ-*BCL6* junctions from 5’ and 3’DSBs with either of two overhang lengths. Horizontal lines denote the total overhang lengths in S’μ plus *BCL6* (dotted line = 95 nt, solid line = 207 nt). **(D)** Resection of 38 nt S’μ overhang at S’μ-*BCL6* junctions when *BCL6* overhang length is varied. **(E)** Microhomology usage at S’μ-*BCL6* junctions from 5’ and 3’DSBs with either of two overhang lengths. Black line denotes mean microhomology usage.

WRC motifs are abundant in the switch regions but less dense in “off-target” genes [30]. Accordingly, the frequency of AID-dependent mutations is higher in immunoglobulin loci than “off-target” genes [31]. Therefore, one would predict that the density of AID-mediated nicks in “off-target” genes to be lower, giving rise to staggered DSBs with longer overhangs. To test whether longer 5’ or 3’ overhangs in *BCL6* affect the frequency and nature of Cas9-mediated der3, we used two pairs of sgRNAs in *BCL6* to generate 5’ and 3’DSBs with either a 57 or 169 nt overhang while keeping overhang length in S’μ constant (Fig 5A).

Like we observed in Cas9-mediated switching, increasing the overhang length in *BCL6* did not significantly affect Cas9-mediated der3 frequency (Fig 5B). 5’DSBs remained better substrates for Cas9-mediated der3 than 3’DSBs whether *BCL6* overhangs were short or long (Fig 5B), supporting the idea that DSB polarity influences suitability for recombination more than overhang length. 5’DSBs with longer overhangs in *BCL6* were resected to the new sum total of S’μ and *BCL6* overhangs, while 3’DSBs with longer overhangs in *BCL6* were filled in (Fig 5C, dotted versus solid line, S3 Fig). Increasing overhang length of staggered DSBs in *BCL6* had no significant effect on resection of S’μ (Fig 5D), supporting our hypothesis that resection of staggered DSBs in non-homologous partners is not typically symmetrical. As with Cas9-induced switching, we found no effect of overhang length on microhomology usage at Cas9-mediated der3 junctions (Fig 5E). Together, these data suggest that DSB polarity plays a more significant role than overhang length in dictating the frequency and nature of genome rearrangements in human B cells.

### 5’ and 3’DSBs lead to recombination junctions with templated insertions

**Fig 6:**
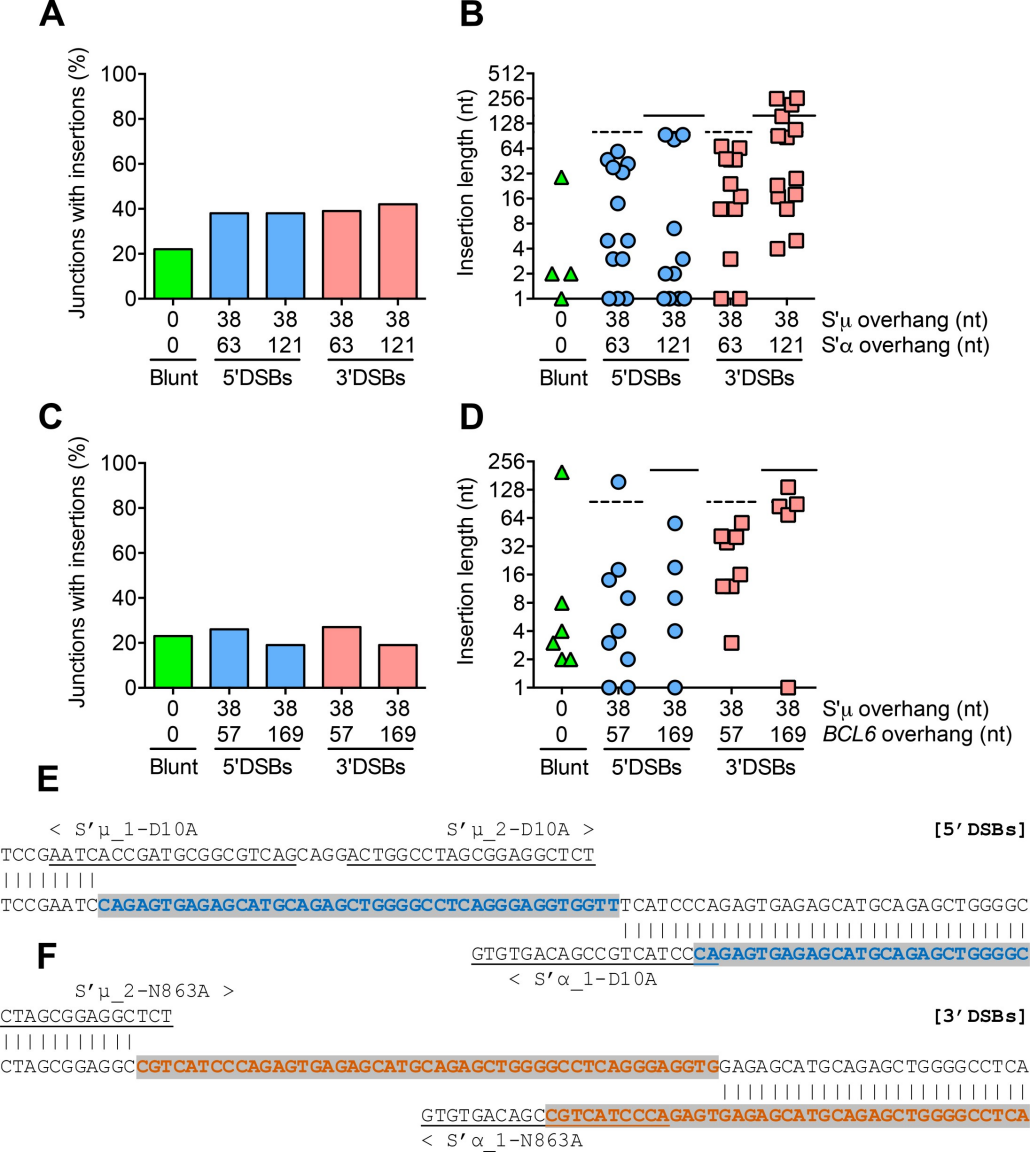
S’μ-S’α and S’μ-*BCL6* junctions from staggered DSBs exhibit “templated insertions”. **(A)** Percentage of all sequenced S’μ-S’α junctions containing insertions of any length. **(B)** Lengths of insertions at S’μ-S’α junctions formed from blunt DSBs and staggered DSBs with either of two overhang lengths. Horizontal lines denote the total overhang lengths in S’μ plus S’α (dotted line = 101 nt, solid line = 159 nt). **(C)** Percentage of all sequenced S’μ-*BCL6* junctions containing insertions of any length. **(D)** Lengths of insertions at S’μ-*BCL6* junctions formed from blunt DSBs and staggered DSBs with either of two overhang lengths. Horizontal lines denote the total overhang lengths in S’μ plus *BCL6* (dotted line = 95 nt, solid line = 207 nt). **(E)** Example of “templated insertion” at S’μ-S’α junction from 5’DSBs and **(F)** 3’DSBs. Underlined sequences represent sgRNA sequences. “<” and “>” point toward PAM sequences and indicate predicted Cas9 cut site 3 nt upstream of PAM sequence. Colored/highlighted nucleotides indicate sequence alignment.

Our sequencing analysis of recombination events revealed inserted nucleotides at a subset of S’μ-S’α and S’μ-*BCL6* junctions. Although the frequency of insertions at S’μ-S’α and S’μ-*BCL6* junctions was similar regardless of DSB structure or polarity (Fig 6A and 6C), staggered DSBs led to junctions with longer insertions than blunt DSBs (Fig 6B and 6D). That is, insertions at most junctions from blunt DSBs were only a few nucleotides long, whereas insertions at junctions from 5’ and 3’ DSBs were almost as long as the overhang itself (Fig 6B and 6D, dotted and solid lines). Indeed, these insertions were mostly composed of duplications of the S’μ, S’α, or *BCL6* overhangs themselves (Fig 6E and 6F). The presence of these “templated insertions” suggest that staggered DSBs undergo cycles of resection and filling in until repair is achieved. Our data suggest that the presence of a single-stranded DNA overhang may stimulate iterative repair more than a blunt DSB end.

## Discussion

In this study, we used CRISPR/Cas9 to generate site-specific, predefined DSB structures to model DNA lesions arising downstream of AID activity in human B cells. We found that DSB structure strongly influenced the mode of end processing associated with DNA repair leading to genome rearrangements like CSR or chromosomal translocations. The end processing profiles we observed at switch or translocation junctions were often, but not always, in alignment with generally accepted definitions of NHEJ and A-EJ. For example, we observed that 5’DSBs typically led to switch and translocation junctions exhibiting increased resection and increased microhomology, two characteristics of repair by A-EJ, compared to blunt or 3’DSBs. A previous study found that decreasing the density of nicks in the switch regions by knocking down AID, presumably leading to more staggered DSBs rather than blunt DSBs, increased microhomology usage at switch junctions [32]. We expand on this observation by showing that staggered DSBs specifically with 5’ overhangs skew towards increased microhomology usage at switch and translocation junctions. In addition, we previously found that 5’DSBs were preferentially repaired by A-EJ in CH12 mouse B cells [13], suggesting that the influence of DSB polarity on end-joining pathway choice is likely a general property of mammalian cells.

However, we also found that 3’DSBs undergo more end processing than blunt DSBs, although not in a manner characterized by resection. Specifically, we observed that while 5’ overhangs tended to be resected during end-joining, 3’ overhangs were filled in during end-joining. This result suggests that DSB polarity not only influences repair by NHEJ or A-EJ, but also how DSBs are processed and repaired by specific subsets of enzymes. It is not currently known which enzymes resect 5’DSBs or fill in 3’DSBs during end-joining. One recent study found that mutation of the 5’ to 3’ exonuclease CtIP, but not Mre11 or Exo1, increased the fraction of precisely ligated DSBs with no resection, suggesting that CtIP is required for resection of DSBs [33]. A second study showed that either CtIP knockdown or chemical inhibition of Mre11 endo- and exonuclease activity led to a reduction in chromatin-bound RPA, a marker of single-stranded DNA, in G2 phase after ionizing radiation-induced DNA damage [34]. This result suggests that several candidate 5’ to 3’ exonucleases can resect DSBs *in vivo*, but could potentially act redundantly. Alternatively, 5’ overhangs could be removed by a flap endonuclease such as FEN1 [35]. We acknowledge that the manner(s) by which nucleotides were resected in our study, whether through exonucleolytic degradation or endonucleolytic degradation, differ mechanistically. Our model allows us only to quantify the number of nucleotides lost from the native sequence in a given switch or translocation junction, not the mechanism by which those nucleotides were lost. However, we can reasonably conclude that any resection beyond the sum total overhang length was likely achieved through exonucleolytic degradation. Regardless of how 5’ overhangs are removed, our data suggest that removal of the 5’ overhang might be required for end-joining, perhaps in order to expose microhomology to facilitate ligation.

In contrast, filling in of 3’ overhangs during end-joining is poorly understood. One known example of physiological 3’DSBs in end-joining occurs during V(D)J recombination, whereby the opening of DNA hairpins at coding ends by the endonuclease Artemis results in 3’ overhangs [36]. These 3’ overhangs can be filled in, presumably by a DNA polymerase, giving rise to P nucleotides and junctional diversity within the B cell receptor repertoire. However, the mechanism for filling in 3’ overhangs leading to P-nucleotides has not yet been characterized. Identifying the enzymes that process 5’ and 3’DSBs leading to end-joining will help elucidate how the spectrum of AID-dependent DSBs are recognized, processed, and ligated to complete CSR or translocations. Our data support the model that different factors and end-joining pathways are indeed required to repair different DSB structures.

One subset of enzymes that is known to recognize and repair AID-dependent DSBs comprise the NHEJ pathway. In this report, we demonstrate that NHEJ is indeed required for Cas9-mediated switching regardless of DSB polarity (Fig 3A–3C). This finding supports the notion that NHEJ is the primary end-joining pathway leading to CSR in human B cells as it is in mouse B cells. Interestingly, we found that the role of NHEJ in chromosomal translocations was contingent on DSB polarity. Our data demonstrate that NHEJ deficiency led to a defect in Cas9-induced der3 using staggered DSBs, but a dramatic increase in der3 using blunt DSBs (Fig 3D–3F). This finding is similar to a previous study showing that chromosomal translocations were reduced in NHEJ-deficient human cells using endonucleases that generated staggered DSBs, but were unchanged using blunt DSBs [29]. We had also found that ligase IV deficiency in CH12 mouse B cells led to a more pronounced increase in translocations from blunt DSBs than 5’DSBs [13]. Furthermore, we observed in this study that translocation junctions from blunt DSBs exhibited more microhomology and resection than switch junctions from blunt DSBs (Figs 1D, 1E, 2C and 2D). Taken together, our results suggest that in the absence of NHEJ, A-EJ prefers joining blunt DSBs over staggered DSBs leading to chromosomal translocations in human B cells.

We propose two explanations for this observation. First, perhaps the act of converting a blunt DSB into a staggered DSB represents a key signaling step during A-EJ. Blunt DSBs can indeed be repaired by A-EJ; indeed, we observe that in wild-type cells, blunt DSBs occasionally lead to recombination junctions with elevated resection and microhomology usage (Figs 1D, 1E, 2D and 2E). Thus, blunt DSBs generated by Cas9 are sometimes resected into staggered DSBs when they are finally repaired. A-EJ in NHEJ-deficient cells might magnify this processing, which could stimulate chromosomal translocations. Still, it is unclear why this would not also apply to CSR, evoking the larger question of why A-EJ drives chromosomal translocations but not CSR in mouse B cells.

Second, A-EJ is typically suppressed or overshadowed by NHEJ, so the preference for A-EJ to repair blunt DSBs leading to chromosomal translocations may be due to a lack of competition from NHEJ. Several studies have demonstrated that NHEJ suppresses A-EJ during CSR, as evidenced by an overall increase in microhomology at switch junctions from when NHEJ is depleted in mouse B cells [7,9]. This may be because binding of NHEJ factors to DSB ends inhibits resection, a step shared between homologous recombination and A-EJ, or because NHEJ simply occurs faster than A-EJ [37]. We observed that the majority of switch and translocations junctions from blunt DSBs in this study exhibited little to no microhomology, suggesting repair predominantly by NHEJ (Figs 1E and 2E). In NHEJ-deficient cells, however, A-EJ is free to engage blunt DSBs that are typically repaired by NHEJ without competition for DSB ends, inhibition of resection, or time constraints. These atypical circumstances could lead to increased irregular end-joining such as chromosomal translocations.

In this report, we observed that 5’DSBs were better substrates for Cas9-mediated switching and translocations than 3’DSBs (Figs 1C and 2B). This pattern was independent of overhang length (Figs 4B and 5B). However, the reason behind this bias is unknown. One possible explanation is that 5’ to 3’ resection is an important step in end-joining as it is for homologous recombination. For example, Cas9-induced 5’DSBs stimulate higher rates of homologous recombination than 3’DSBs using the DR-GFP homologous recombination reporter assay [38,39]. Another study found that Cas9-induced 5’DSBs led to more gene conversion, a pathway that relies on homologous recombination, than 3’DSBs [40]. In support of this hypothesis, we observed in this study that 5’ overhangs tended to be resected prior to end-joining while 3’ overhangs were filled in. Perhaps the lack of a 5’ overhang to resect makes 3’DSBs poor substrates for end-joining.

Another possible explanation for the increased level of Cas9-mediated switching and der3 using 5’DSBs versus 3’DSBs could be due to different enzymatic activity of the Cas9 D10A and N863A nickases. That is, Cas9 D10A could simply lead to more DSBs than Cas9 N863A. Some studies have demonstrated that currently available RuvC-null Cas9 nickases (e.g. D10A) generate more indels than HNH-null Cas9 nickases (e.g. N863A or H840A) [41,42]. However, indel formation is likely an inaccurate measure of cleavage efficiency, as we and others demonstrate that DSB polarity itself can influence repair by NHEJ or A-EJ which may in turn lead to different frequencies of indel formation [13,40]. Nonetheless, Cas9 D10A and N863A nickases have been shown to have similar nicking efficiency *in vitro* [40]. An ideal study would use the same nuclease to produce 5’ and 3’DSBs, which in theory could be achieved using the Cas9 D10A nickase and orienting sgRNAs with PAMs facing outwards or inwards, respectively. In practice, however, paired sgRNAs with inward-facing PAMs are incompatible with Cas9 cleavage [40,41].

While we believe that the Cas9/Cas9 nickase system is the best currently available method to model AID-dependent DSB intermediates for CSR and chromosomal translocations, we acknowledge that Cas9 does not perfectly mimic AID in all aspects. For example, AID is a deaminase while Cas9 is a nuclease. Modeling AID-dependent DSBs with Cas9 therefore overlooks two intermediate enzymatic steps—deamination of dC by AID, and excision of dUs by UNG to create an abasic site, followed by the creation of a nick by APE—between AID deamination and nick formation. Future studies could use an enzymatically dead Cas9 fused to cytidine deaminase rather than the Cas9 nickase to more accurately model AID function [43]. Another limitation of our model system is that we targeted Cas9 cleavage 5’ of the switch regions rather than the repetitive switch regions themselves to avoid introducing multiple DSBs with one sgRNA. Although the loci 5’ of the switch regions are physiological sites of AID deamination [44], our model does not take into account the formation of secondary structures in the repetitive switch regions that could be conducive to deoxycytidine deamination and DSBs [45]. Cas9 may also imperfectly model AID during CSR and chromosomal translocations in its residence time on DNA after cleavage and its influence on DNA repair processes.

In conclusion, we found that DSB structure strongly influences repair by NHEJ and A-EJ in human B cells. Our data suggest that the spectrum of AID-dependent DSBs is not uniformly recognized and repaired by human B cells. These findings expand our knowledge of DSB repair in B cells leading to humoral immunity or lymphomagenesis and may eventually inform therapeutic interventions designed to promote desired DSB repair outcomes.

## Methods

### Cell culture conditions

BJAB cells were cultured in RPMI 1640 medium with L-Glutamine supplemented with 10% fetal bovine serum, 5% NCTC 109, 50 μM β-mercaptoethanol, and penicillin/streptomycin. Ramos cells were cultured in IMDM supplemented with 10% fetal bovine serum and penicillin/streptomycin. All cells were cultured at 37°C and 5% CO_2_.

### Construction of Cas9- and sgRNA expression vectors

sgRNAs were chosen based on favorable on- and off-target scores calculated by Benchling [46]. sgRNAs were cloned into plasmids encoding Cas9 (Addgene: #42330), Cas9D10A (Addgene: #42335), and Cas9N863A. Cas9N863A plasmid was constructed by site-directed mutagenesis of Addgene #42330.

### Transfection of CRISPR/Cas9 elements for chromosomal translocations and class switch recombination

One million BJAB cells in standard electroporation buffer were transfected with 4 μg of each relevant plasmid using a BioRad Gene Pulser using the following parameters: 350 V, 975 μF, ∞ Ω, and 4 mm gap. BJAB cells were incubated on ice for 5 minutes and then transferred into complete media. One million Ramos cells in IMDM were transfected using the following parameters: 250 V, 950 μF, ∞ Ω, and 4 mm gap. Ramos cells were incubated at room temperature for 5 minutes and then transferred into complete media. For chromosomal translocations, cells were harvested 5 days post-transfection and lysed to isolate genomic DNA by standard phenol-chloroform extraction and ethanol precipitation. For Cas9-induced switching, cells were harvested 5 days (BJAB) or 3 days (Ramos) post-transfection for genomic DNA isolation by proteinase K digestion and flow cytometry analysis of IgA expression (see below).

### CRISPR/Cas9-mediated genome editing

One million BJAB cells in standard electroporation buffer were transfected with 4 μg of sgRNAs LIG4 BRCTd G1 and G2 targeting DNA ligase 4 (S1 Table). Cells were plated at 0.5 cells/well in a 96-well tissue culture plate 3 days post-transfection to obtain individual clones. Clones were screened for mutations by the mismatch cleavage assay as previously described [13]. Knockout clones were confirmed by sequencing.

### Quantitative PCR (qPCR) for chromosomal translocation quantification

Der3 translocations were detected by nested PCR. Round 1 was amplified from 8 separate reactions using primers S’μ F.1 and BCL6 R.1 (S1 Table) and 150 ng of phenol chloroform-isolated genomic DNA by conventional PCR. Amplicons from the 8 reactions were then pooled and diluted ten-fold to use as template for Round 2. Round 2 was amplified by qPCR using S’μ F.2 and BCL6 R.2 primers (S1 Table) and SsoAdvanced Universal SYBR Green Supermix (BioRad). A control genomic region on chromosome 13 (S1 Table) was amplified from 10 ng of genomic DNA template by nested PCR to obtain reference C_t_ values, using round 1 primers c13 F.1 and R.1 followed by round 2 primers c13 F.2 and R.2 (S1 Table). Der3 frequencies were calculated using the ΔΔC_t_ method, using S’μ_1 + BCL6_1 WT Cas9 (Fig 2B, second column) or S’μ_1+2 + BCL6_1+2 Cas9 D10A (Fig 5B, second column) as control samples.

### Flow cytometry quantification of IgA-switched cells

Cells were harvested 5 days post-transfection, washed, and incubated with anti-IgA antibodies (Southern Biotech, cat. 2050–09) for 30 min at 4°C in the dark. Cells were washed and analyzed for IgA expression on a BD LSR II at the Faculty of Medicine Flow Cytometry Facility (University of Toronto, Toronto, Ontario, Canada).

### Amplification, cloning, and sequencing of S’μ-BCL6 and S’μ-S’α junctions

Der3 junctions for sequencing analysis were amplified by nested conventional PCR from genomic DNA using primers S’μ F.1 and BCL6 R.1 (round 1) and S’μ F.2 and BCL6 R.2 (round 2) (S1 Table). S’μ-S’α junctions were amplified from genomic DNA by nested PCR using primers S’μ F.1 and S’α R.1 (round 1) and S’μ F.2 and S’α R.2 (round 2) (S1 Table). Junction PCR products were purified using standard spin columns, cloned into a TA-cloning vector (pGEM-T Easy, Promega), and transformed into chemically competent bacteria. Plasmids containing proper inserts were purified and Sanger sequenced at The Center for Applied Genomics (The Hospital for Sick Children, Toronto, Ontario, Canada).

### Sequencing analysis of S’μ-Bcl6 and S’μ-S’α junctions

Alignments of S’μ*-BCL6* and S’μ-S’α junctions to reference sequences were performed using Basic Local Alignment Search Tool (BLAST). The following reference sequences were used for analysis and are available from the National Center for Biotechnology Information (NCBI): *IGH* (NCBI Accession NG_001019) and *BCL6* (NCBI Accession NG_007149.1). Resection is defined as the number of nucleotides in the native sequence spanning from 3 nt upstream of the PAM of the most distal sgRNA to the point where the junction changes alignment from one reference sequence to another, including any microhomologous nucleotides, that are not in the recombined sequence. Insertions are defined as the number of nucleotides in the recombined sequence that are not in the native sequence, starting from the end of alignment between the recombined and native sequences. All junctions included in sequencing data in this report are unique to genomic DNA harvested from a single transfection, and are reported in S1 Appendix.

### Statistical analysis

All statistical comparisons were made using the Mann-Whitney test. Stars denote p-values: ns>0.05, *≤0.05, **≤0.01, ***≤0.001, ****≤0.0001.

## Supporting information

S1 Fig Visualization of Cas9-mediated switching and der3 in BJAB cells. **(A)** Representative flow cytometry plots showing IgA+ BJAB cells as a result of Cas9-mediated switching, 5 days post-transfection with Cas9/sgRNA plasmids. Live BJAB cells were identified by forward and side scatter, then gated on singlets. **(B)** Representative gel electrophoresis images showing Cas9-mediated der3 amplified by conventional, nested PCR. Leftmost lane is a 100 nt ladder. Expected product size, assuming no resection and no insertion, is approximately 1040 nt. Each gel shows 8 independent PCRs amplified from 150 ng of genomic DNA template.
(TIF)

S2 Fig Quantification of distance between AID-mediated mutations at murine switch region μ. Graphical representation of distance between AID-mediated transition mutations in switch region μ from UNG^-/-^MSH2^-/-^ mice. 507 pairs of transition mutations on opposite strands from 127 analyzed sequences are depicted above. All values represent the distance between mutations (i.e. immediately adjacent mutations are assigned a distance of 0 nt). Black line denotes mean distance between AID-mediated transition mutations. See S3 Table for more information.
(TIF)

S3 Fig Resection of S’μ versus S’α or *BCL6* at junctions from short and long staggered DSBs. **(A)** Graphical representation of S’μ resection versus S’α resection using short and **(B)** long 5’ or 3’ overhangs in S’α. S’μ overhangs are kept constant. Dotted lines denote overhang lengths in S’μ and S’α. Each data point represents a unique S’μ-S’α junction. **(C)** Graphical representation of S’μ resection versus *BCL6* resection using short and **(D)** long 5’ or 3’ overhangs in *BCL6*. Dotted lines denote overhang lengths in S’μ and *BCL6*. Each data point represents a unique S’μ-*BCL6* junction. S’μ overhang length is kept constant in all panels. The total resection measured at each unique junction is presented in Figs 1, 2, 4 and 5.
(TIF)

S4 Fig DSB polarity influences end-joining pathway choice despite altered sequence context. **(A)** Schematic depicting sgRNAs designed to target a different region upstream of switch region α (S’α_I and S’α_II, shown in blue) and the resulting chromosome after Cas9-mediated switching. WT Cas9 and S’α_I produces a blunt DSB, while either Cas9 nickase with S’α_I and S’α_II gives rise to a staggered DSB with a 58 nt overhang. Schematic is not to scale. **(B)** Total resection of S’μ and S’α at S’μ-S’α junctions from blunt, 5’, and 3’DSBs. Dotted line denotes the total overhang length in S’μ plus S’α (96 nt). **(C)** Microhomology usage at S’μ-S’α junctions from blunt, 5’, and 3’DSBs. Black line denotes mean microhomology usage. ^The vast majority of S’μ-S’α junctions from blunt DSBs that were sequenced exhibited 0 nt of resection and 1 nt of microhomology, but had to be excluded from analysis for being non-unique.
(TIF)

S5 Fig CRISPR/Cas9 editing of *LIG4* in BJAB cells. Sequence alignments of *LIG4* from wild-type BJAB cells and three *LIG4*^-/-^ BJAB clones. Amino acid translation and residue number of wild-type *LIG4* are denoted above the nucleotide sequence. Underlined sequences represent LIG4 BRCTd G1 and G2 sgRNA sequences (S1 Table).
(TIF)

S1 Table Oligonucleotides used in this study. (DOCX)

S2 Table Cas9 and sgRNA combinations used to produce blunt, 5’, and 3’DSBs in S’μ, S’α, and *BCL6*. (DOCX)

S3 Table Analysis of AID-dependent mutations in switch region μ of Ung^-/-^Msh2^-/-^ mice. (DOCX)

S1 Appendix S’μ-S’α and S’μ-BCL6 junction sequence alignments. (DOCX)

S2 Appendix Numerical data underlying all graphs and figures. (XLSX)
