# Supplementary figures and images for: DSB structure impacts DNA recombination leading to class switching and chromosomal translocations in human B cells
Source: PLoS Genet. 2019 Apr 4;15(4):e1008101. doi: 10.1371/journal.pgen.1008101 (PMC6467426; doi:10.1371/journal.pgen.1008101)

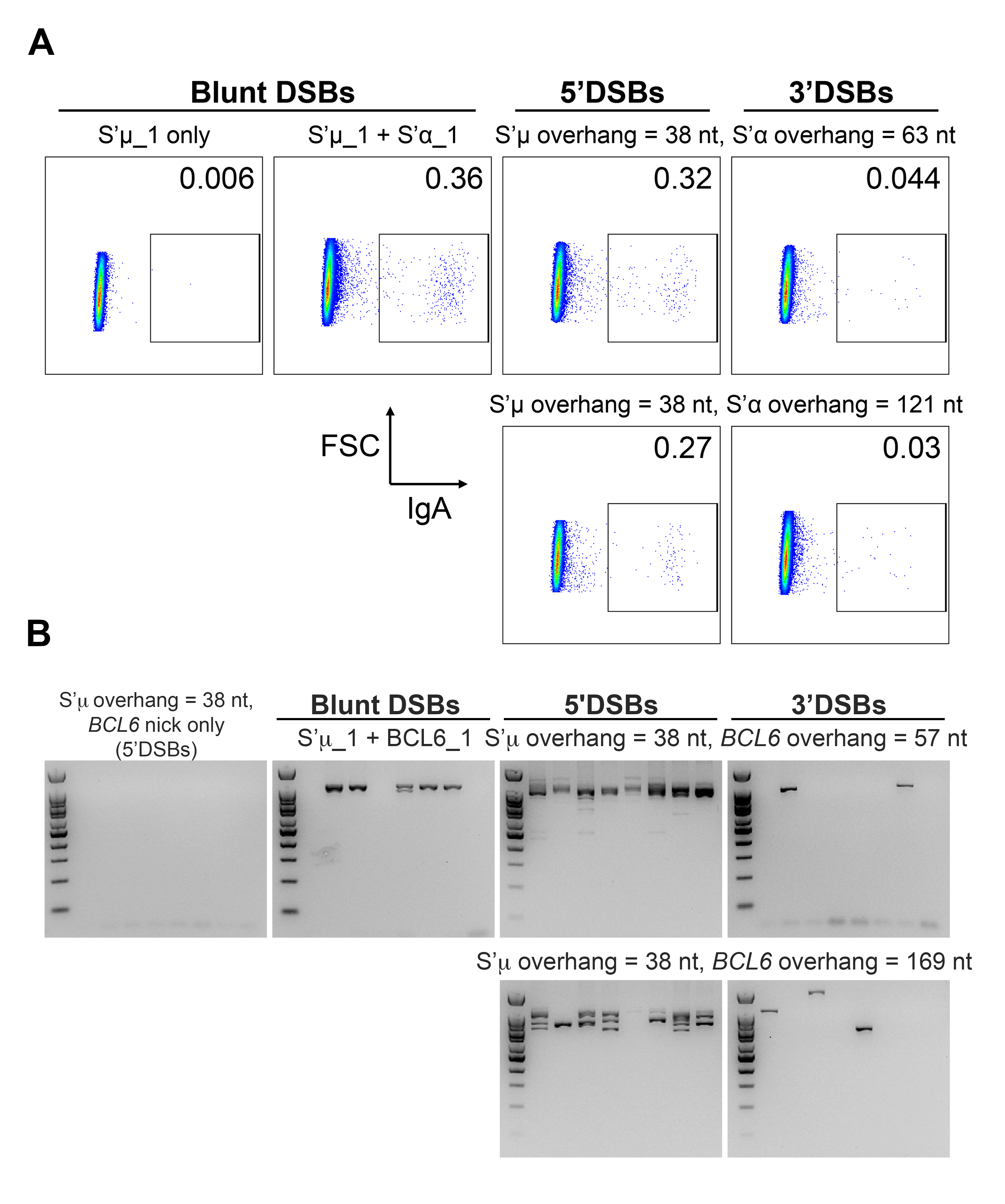

Supplement: S1 Fig — (A) Representative flow cytometry plots showing IgA+ BJAB cells as a result of Cas9-mediated switching, 5 days post-transfection with Cas9/sgRNA plasmids. Live BJAB cells were identified by forward and side scatter, then gated on singlets. (B) Representative gel electrophoresis images showing Cas9-mediated der3 amplified by conventional, nested PCR. Leftmost lane is a 100 nt ladder. Expected product size, assuming no resection and no insertion, is approximately 1040 nt. Each gel shows 8 independent PCRs amplified from 150 ng of genomic DNA template. (TIF) [file pgen.1008101.s001.tif]

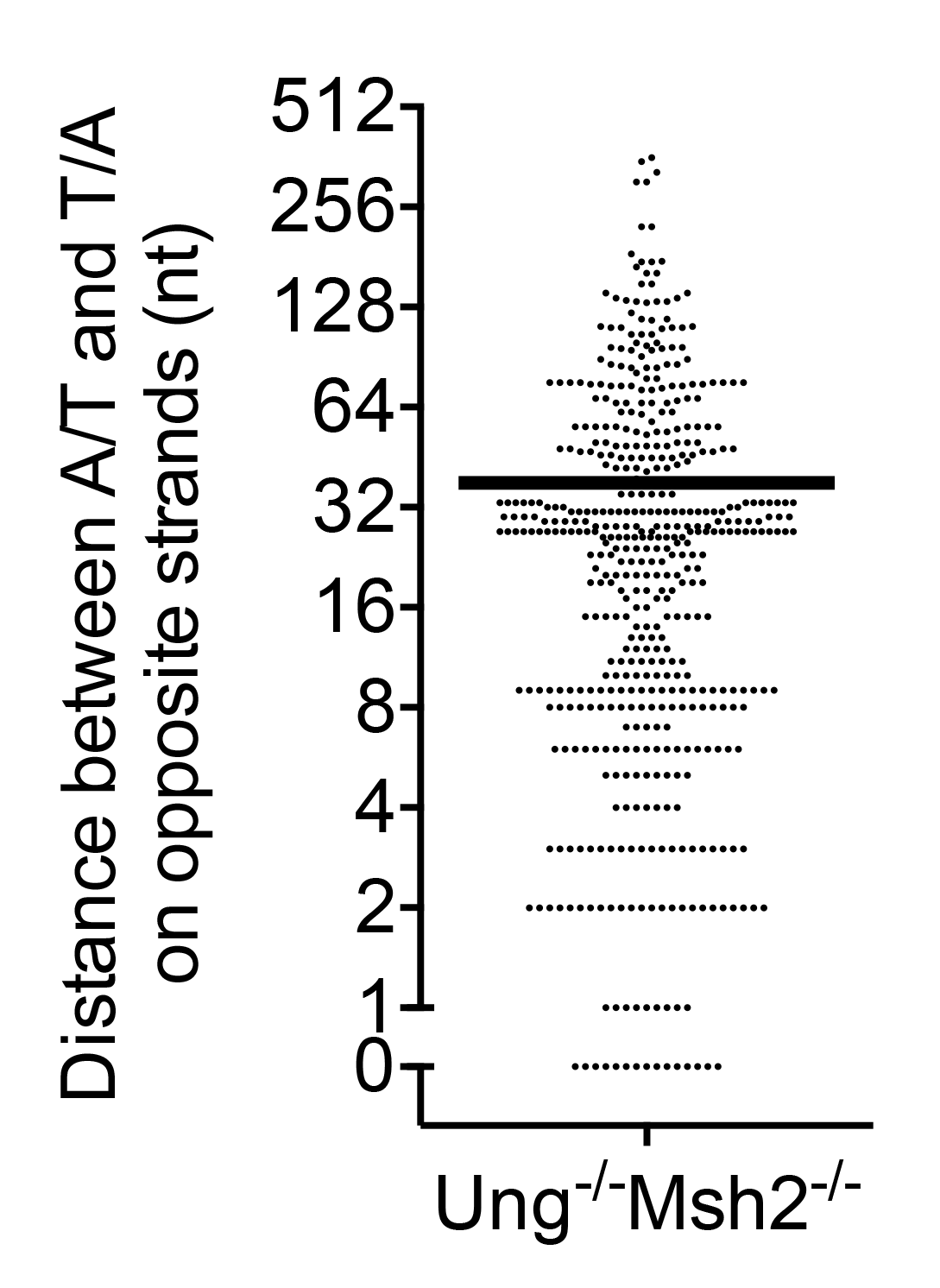

Supplement: S2 Fig — Graphical representation of distance between AID-mediated transition mutations in switch region μ from UNG-/-MSH2-/- mice. 507 pairs of transition mutations on opposite strands from 127 analyzed sequences are depicted above. All values represent the distance between mutations (i.e. immediately adjacent mutations are assigned a distance of 0 nt). Black line denotes mean distance between AID-mediated transition mutations. See S3 Table for more information. (TIF) [file pgen.1008101.s002.tif]

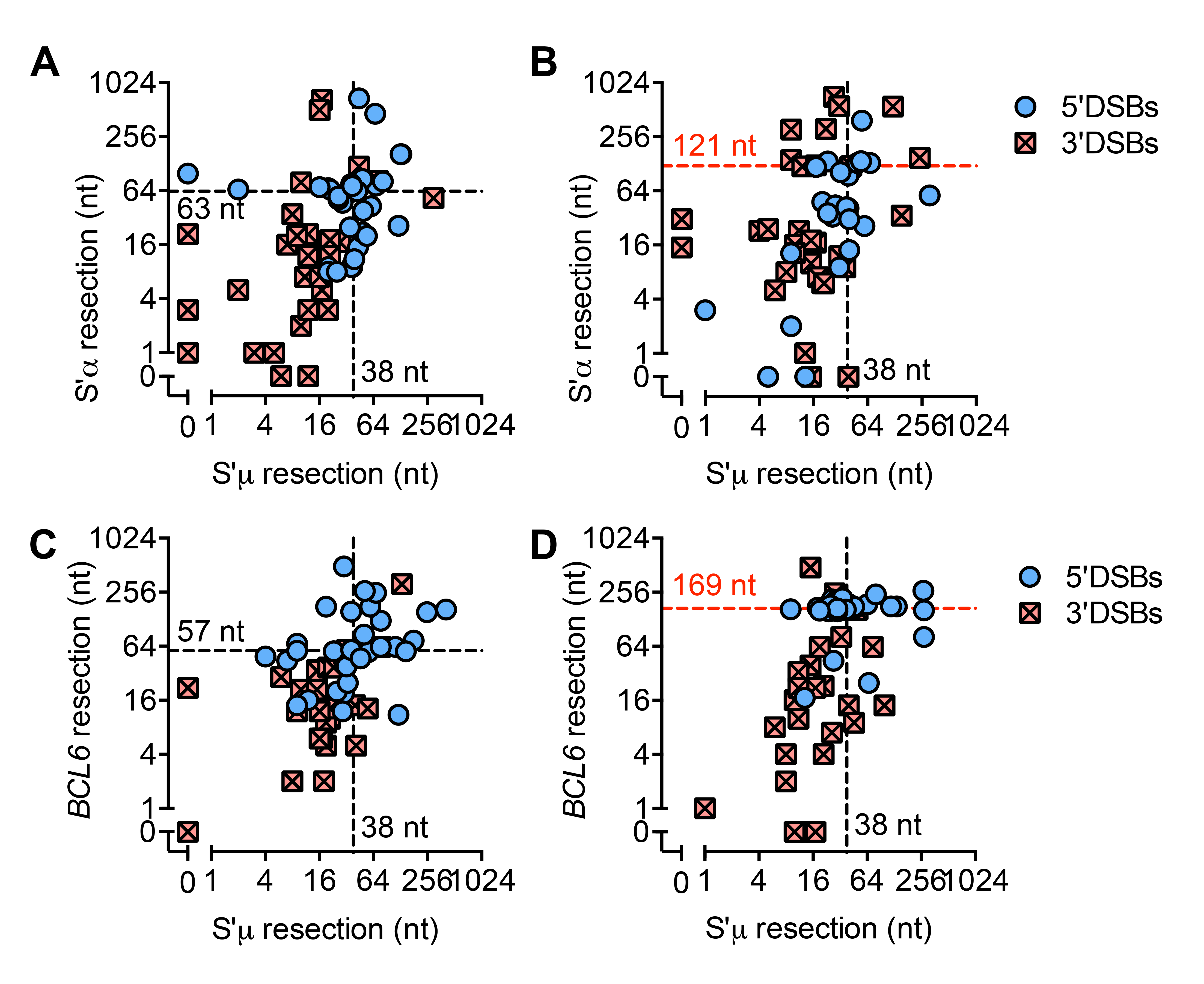

Supplement: S3 Fig — (A) Graphical representation of S’μ resection versus S’α resection using short and (B) long 5’ or 3’ overhangs in S’α. S’μ overhangs are kept constant. Dotted lines denote overhang lengths in S’μ and S’α. Each data point represents a unique S’μ-S’α junction. (C) Graphical representation of S’μ resection versus BCL6 resection using short and (D) long 5’ or 3’ overhangs in BCL6. Dotted lines denote overhang lengths in S’μ and BCL6. Each data point represents a unique S’μ-BCL6 junction. S’μ overhang length is kept constant in all panels. The total resection measured at each unique junction is presented in Figs 1, 2, 4 and 5. (TIF) [file pgen.1008101.s003.tif]

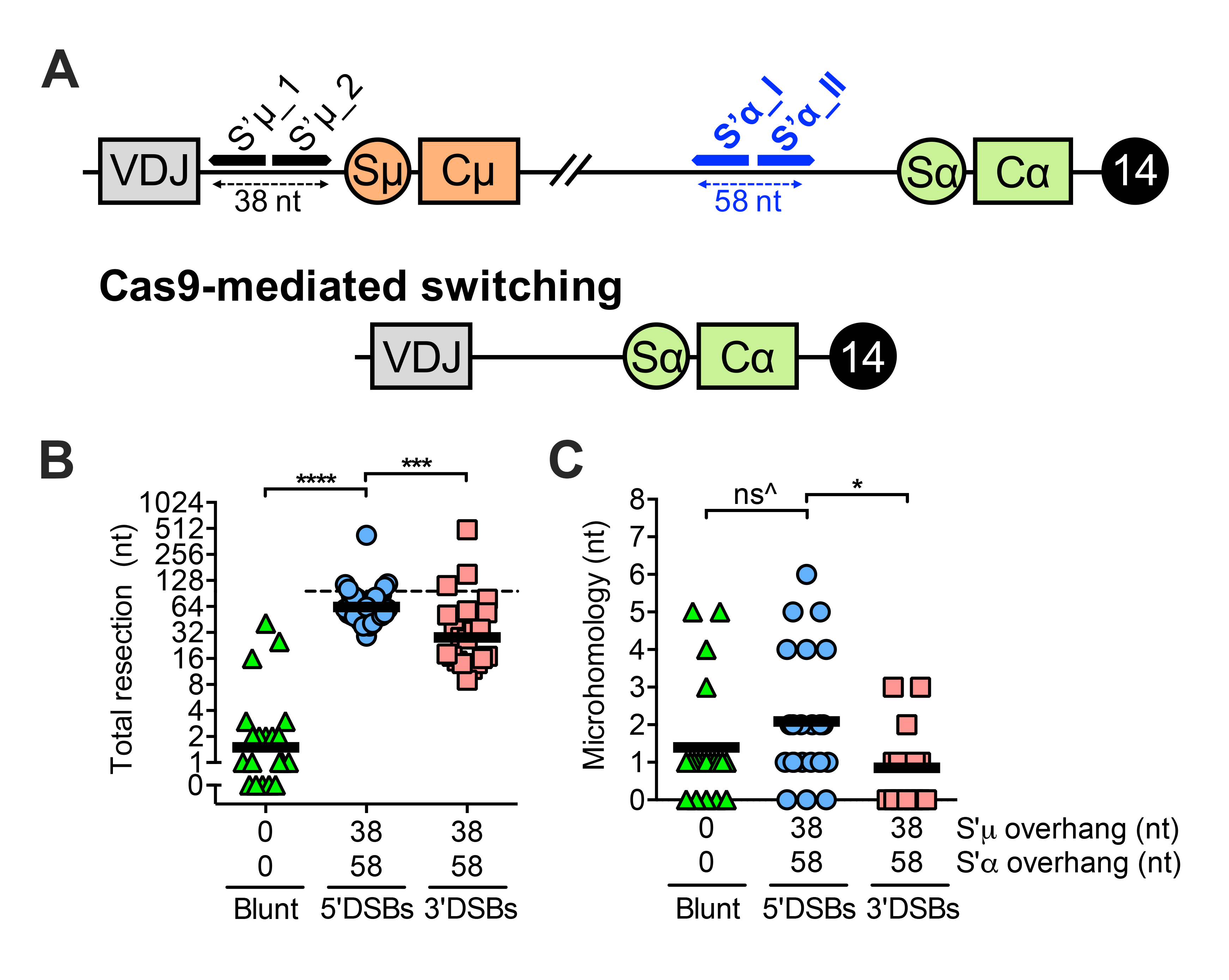

Supplement: S4 Fig — (A) Schematic depicting sgRNAs designed to target a different region upstream of switch region α (S’α_I and S’α_II, shown in blue) and the resulting chromosome after Cas9-mediated switching. WT Cas9 and S’α_I produces a blunt DSB, while either Cas9 nickase with S’α_I and S’α_II gives rise to a staggered DSB with a 58 nt overhang. Schematic is not to scale. (B) Total resection of S’μ and S’α at S’μ-S’α junctions from blunt, 5’, and 3’DSBs. Dotted line denotes the total overhang length in S’μ plus S’α (96 nt). (C) Microhomology usage at S’μ-S’α junctions from blunt, 5’, and 3’DSBs. Black line denotes mean microhomology usage. ^The vast majority of S’μ-S’α junctions from blunt DSBs that were sequenced exhibited 0 nt of resection and 1 nt of microhomology, but had to be excluded from analysis for being non-unique. (TIF) [file pgen.1008101.s004.tif]

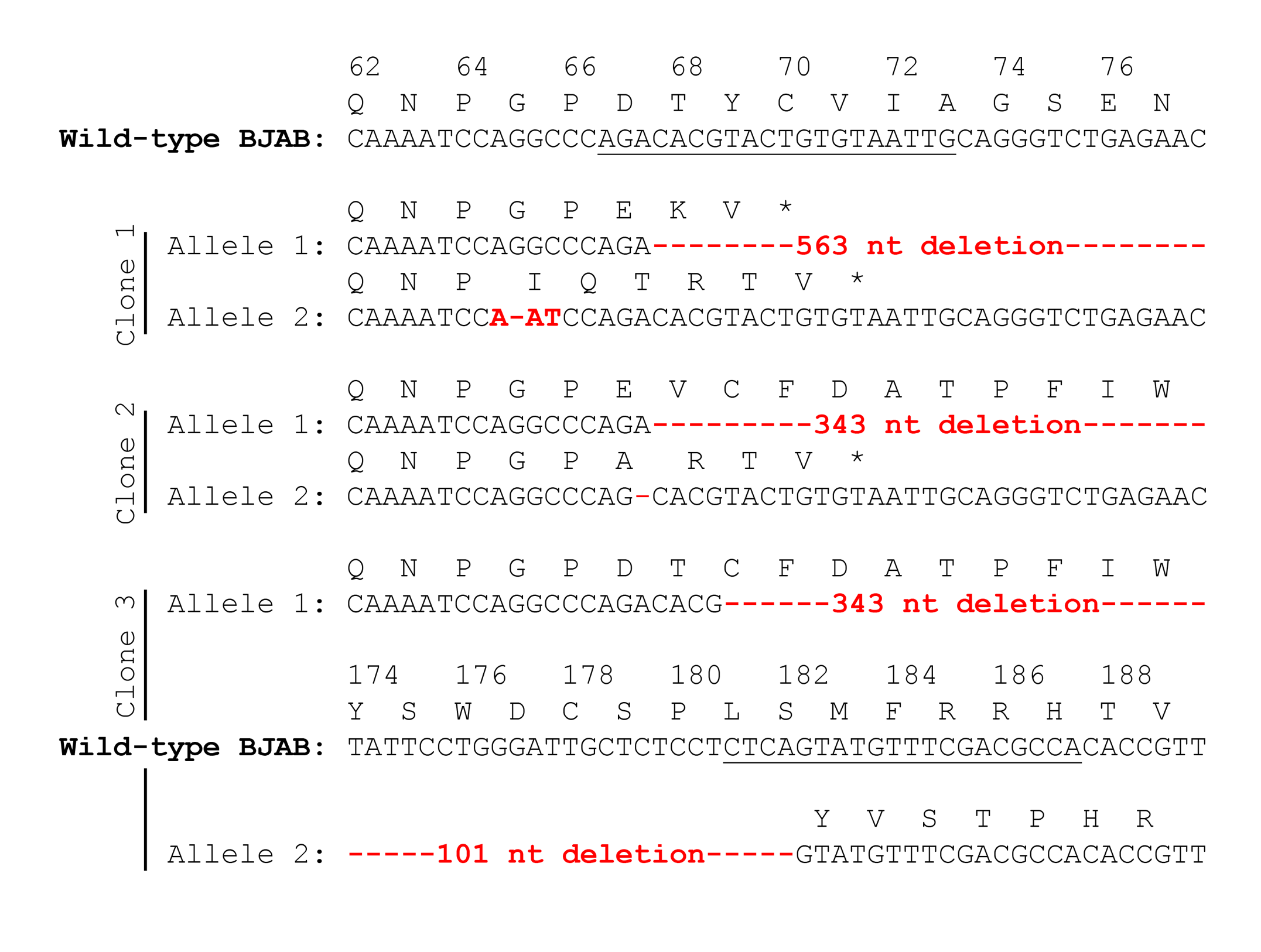

Supplement: S5 Fig — Sequence alignments of LIG4 from wild-type BJAB cells and three LIG4-/- BJAB clones. Amino acid translation and residue number of wild-type LIG4 are denoted above the nucleotide sequence. Underlined sequences represent LIG4 BRCTd G1 and G2 sgRNA sequences (S1 Table). (TIF) [file pgen.1008101.s005.tif]
