# Supplementary material for: DSB structure impacts DNA recombination leading to class switching and chromosomal translocations in human B cells
Source: PLoS Genet. 2019 Apr 4;15(4):e1008101. doi: 10.1371/journal.pgen.1008101 (PMC6467426; doi:10.1371/journal.pgen.1008101)
Supplement: S1 Table — (DOCX) [file pgen.1008101.s006.docx]

**S1 Table. Oligonucleotides used in this study.**

| **Name** | **Type** | **Purpose** | **Sequence (5’ to 3’)** |
| --- | --- | --- | --- |
| S’μ_1 | sgRNA | Cas9-induced DSBs | CTGACGCCGCATCGGTGATT |
| S’μ_2 | sgRNA |  | ACTGGCCTAGCGGAGGCTCT |
| BCL6_1 | sgRNA |  | ATAATGATCATGAGCAGCGG |
| BCL6_2 | sgRNA |  | TCCGCTCTTGCCAAATGCTT |
| BCL6_3 | sgRNA |  | ATTTATTTATTCTAGCTGTC |
| S’α_1 | sgRNA |  | GTCCTCACGTGGTGGAAAGA |
| S’α_2 | sgRNA |  | AACCAGGAATCGACCCTTCC |
| S’α_3 | sgRNA |  | CTGCTGAACCCACCAGTCTA |
| S’α_I | sgRNA |  | GTCCTCACGTGGTGGAAAGA |
| S’α_II | sgRNA |  | AACCAGGAATCGACCCTTCC |
| LIG4 BRCTd G1 | sgRNA | LIG4 gene editing | CAATTACACAGTACGTGTCT |
| LIG4 BRCTd G2 | sgRNA |  | TGGCGTCGAAACATACTGAG |
| S’μ F.1 | Primer | PCR amplification of S’μ-*BCL6* or S’μ-S’α junctions | ATGGCTGAGGAATGTGTCTCAG |
| S’μ F.2 | Primer |  | CTGCTAGTTTGTGCAAACAGC |
| BCL6 R.1 | Primer |  | CGGAGTTACCCAGAAGGACA |
| BCL6 R.2 | Primer |  | GAGGCCAGGAGCGACGGAGCA |
| S’α R.1 | Primer |  | TGTGAGTGGACTCCCGTTGAG |
| S’α R.2 | Primer |  | AGGTGGCACTCCTACTGTGG |
| c13 F.1 | Primer | qPCR | TGTTTGGCTGGAAGAAGAGGGT |
| c13 F.2 | Primer |  | AGTGAAGTGAATTAAGCCTGATGAG |
| c13 R.1 | Primer |  | CTCCATGAGTTCCAGTGGGTGT |
| c13 R.2 | Primer |  | GAGGGCATCTTTTCCATCTCTAGG |
