# Supplementary material for: DSB structure impacts DNA recombination leading to class switching and chromosomal translocations in human B cells
Source: PLoS Genet. 2019 Apr 4;15(4):e1008101. doi: 10.1371/journal.pgen.1008101 (PMC6467426; doi:10.1371/journal.pgen.1008101)
Supplement: S2 Table — (DOCX) [file pgen.1008101.s007.docx]

**S2 Table. Cas9 and sgRNA combinations used to produce blunt, 5’, and 3’DSBs in S’μ, S’α, and *BCL6*.**

| **S’μ overhang (nt)** | **S’α overhang (nt)** | ***BCL6* overhang (nt)** | **sgRNAs required (S1 Table)** | **Cas9 variant** |
| --- | --- | --- | --- | --- |
| 0 (Blunt) |  |  | S’μ_1 | WT |
| 0 (Blunt) | 0 (Blunt) |  | S’μ_1, S’α_1 | WT |
| 38 | Nick |  | S’μ_1, S’μ_2, S’α_1 | D10A or N863A |
| 38 | 63 |  | S’μ_1, S’μ_2, S’α_1, S’α_2 | D10A or N863A |
| 38 | 121 |  | S’μ_1, S’μ_2, S’α_1, S’α_3 | D10A or N863A |
| 0 (blunt) | 0 (blunt) |  | S’μ_1, S’α_I | WT |
| 38 | 58 |  | S’μ_1, S’μ_2, S’α_I, S’α_II | D10A or N863A |
| 0 (Blunt) |  | 0 (Blunt) | S’μ_1, BCL6_1 | WT |
| 38 |  | Nick | S’μ_1, S’μ_2, BCL6_1 | D10A or N863A |
| 38 |  | 57 | S’μ_1, S’μ_2, BCL6_1, BCL6_2 | D10A or N863A |
| 38 |  | 169 | S’μ_1, S’μ_2, BCL6_1, BCL6_3 | D10A or N863A |
