# Supplementary material for: DSB structure impacts DNA recombination leading to class switching and chromosomal translocations in human B cells
Source: PLoS Genet. 2019 Apr 4;15(4):e1008101. doi: 10.1371/journal.pgen.1008101 (PMC6467426; doi:10.1371/journal.pgen.1008101)
Supplement: S3 Table — (DOCX) [file pgen.1008101.s008.docx]

**S3 Table. Analysis of AID-dependent mutations in switch region μ of Ung^-/-^Msh2^-/-^ mice.**

| Total sequences analyzed | 127 |
| --- | --- |
| Total sequences with at least one pair of opposing A/T and T/A mutations | 76/127 (60%) |
| Total pairs of opposing A/T and T/A mutations | 507 |
| Median distance (nt) | 27 |
| Mean distance (nt) | 37.9 |
