# Supplementary material for: DSB structure impacts DNA recombination leading to class switching and chromosomal translocations in human B cells
Source: PLoS Genet. 2019 Apr 4;15(4):e1008101. doi: 10.1371/journal.pgen.1008101 (PMC6467426; doi:10.1371/journal.pgen.1008101)
Supplement: S1 Appendix — (DOCX) [file pgen.1008101.s009.docx]

**S1 Appendix. S’μ-S’α and S’μ-BCL6 junction sequence alignments**

||| = sequence alignment

NNN = microhomology

NNN = insertion

___ = sgRNA sequence

< and > = point towards PAM

**BJAB, S’μ-S’α junctions from blunt DSBs (WT Cas9, S’μ_1 + S’α_1)**

< S’mu_1

GCTGAAGACAGGACTGTGGAGACACCTTAGAAGGACAGATTCTGTTCCGAATCACCGATGCGGCGTCAG

|||||||||| |||||||||||||||||||||||||||||||||||||||||

GCTGAAGACAAGACTGTGGAGACACCTTAGAAGGACAGATTCTGTTCCGAATTGACAGCCGTCATCCCAGAGTGAGAGCATGCAGAGCTGGGGCCTCAGGGAGGTGGTTAGG

||||||||||||||||||||||||||||||||||||||||||||||||||||||||||||

GTGTGACAGCCGTCATCCCAGAGTGAGAGCATGCAGAGCTGGGGCCTCAGGGAGGTGGTTAGG

< S’alpha_1

< S’mu_1

TGCTGAAGACAGGACTGTGGAGACACCTTAGAAGGACAGATTCTGTTCCGAATCACCGATGCGGCGTCAG

|||||||||||||||||||||||||||||||||||||||||||||||||||||

TGCTGAAGACAGGACTGTGGAGACACCTTAGAAGGACAGATTCTGTTCCGAATATCCCAGAGTGAGAGCATGCAAAGCTGGGGCCTCAGGGAGGTGGTTAGGGCCTGAGG

||||||||||||||||||||| |||||||||||||||||||||||||||||||||||

GTGTGACAGCCGTCATCCCAGAGTGAGAGCATGCAGAGCTGGGGCCTCAGGGAGGTGGTTAGGGCCTGAGG

< S’alpha_1

< S’mu_1

TGAATTATTTCAGTTAAGCATGTTAGTTGGTGGCATAAGAGAAAACTCAATCAGATA...51 nt...AATCACCGATGCGGCGTCAG

|||||||||||||||||||||||||||||||||||||||||||||||||||

TGAATTATTTCAGTTAAGCATGTTAGTTGGTGGCATAAGAGAAAACTCAATGACAGCCGTCATCCCAGAGTGAGAGCATGCAGAGCTGGGGCCTCAGGGAGGTGGTT

|||||||||||||||||||||||||||||||||||||||||||||||||||||||||

GTGTGACAGCCGTCATCCCAGAGTGAGAGCATGCAGAGCTGGGGCCTCAGGGAGGTGGTT

< S’alpha_1

< S’mu_1

GCTGAAGACAGGACTGTGGAGACACCTTAGAAGGACAGATTCTGTTCCGAATCACCGATGCGGCGTCAG

|||||||||| |||||||||||||||||||||||||||||||||||||||||

GCTGAAGACAAGACTGTGGAGACACCTTAGAAGGACAGATTCTGTTCCGAATTGACAGCCGTCATCCCAGAGTGAGAGCATGCAGAGCTGGGGCCTCAGGGAGGTGGTTAGG

||||||||||||||||||||||||||||||||||||||||||||||||||||||||||||

GTGTGACAGCCGTCATCCCAGAGTGAGAGCATGCAGAGCTGGGGCCTCAGGGAGGTGGTTAGG

< S’alpha_1

< S’mu_1

GACAGGACTGTGGAGACACCTTAGAAGGACAGATTCTGTTCCGAATCACCGATGCGGCGTCAG

|||||||||||||||||| |||||||||||||||||||||||||||

GACAGGACTGTGGAGACA-CTTAGAAGGACAGATTCTGTTCCGAATGACAGCCGTCATCCCAGAGTGAGAGCATGCAGAGCTGGGGCCTCAGGGAGGTGGTT

|||||||||||||||||||||||||||||||||||||||||||||||||||||||||

GTGTGACAGCCGTCATCCCAGAGTGAGAGCATGCAGAGCTGGGGCCTCAGGGAGGTGGTT

< S’alpha_1

< S’mu_1

GCTGAAGACAGGACTGTGGAGACACCTTAGAAGGACAGATTCTGTTCCGAATCACCGATGCGGCGTCAG

|||||||||| |||||||||||||||||||||||||||||||||||||||||

GCTGAAGACAAGACTGTGGAGACACCTTAGAAGGACAGATTCTGTTCCGAATTGACAGCCGTCATCCCAGAGTGAGAGCATGCAGAGCTGGGGCCTCAGGGAGGTGGTTAGG

||||||||||||||||||||||||||||||||||||||||||||||||||||||||||||

GTGTGACAGCCGTCATCCCAGAGTGAGAGCATGCAGAGCTGGGGCCTCAGGGAGGTGGTTAGG

< S’alpha_1

< S’mu_1

GTGCTGAAGACAGGACTGTGGAGACACCTTAGAAGGACAGATTCTGTTCCGAATCACCGATGCGGCGTCAG

||||||||||||||||||||||||||||||||||||||||||||||||||||||

GTGCTGAAGACAGGACTGTGGAGACACCTTAGAAGGACAGATTCTGTTCCGAATGCAGAGCTGGGGCCTCAGGGAGGTGGTTAGGGCCTGAGGGGGAACACTCAAAAATGTG

||||||||||||||||||||||||||||||||||||||||||||||| ||||||||||||

GTGTGACAGCCGTCATCCCAGAGTGAGAGCATGCAGAGCTGGGGCCTCAGGGAGGTGGTTAGGGCCTGAGGGGGAACCCTCAAAAATGTG

< S’alpha_1

< S’mu_1

GCTGAAGACAGGACTGTGGAGACACCTTAGAAGGACAGATTCTGTTCCGAATCACCGATGCGGCGTCAG

|||||||||| |||||||||||||||||||||||||||||||||||||||||

GCTGAAGACAAGACTGTGGAGACACCTTAGAAGGACAGATTCTGTTCCGAATTGACAGCCGTCATCCCAGAGTGAGAGCATGCAGAGCTGGGGCCTCAGGGAGGTGGTTAGG

||||||||||||||||||||||||||||||||||||||||||||||||||||||||||||

GTGTGACAGCCGTCATCCCAGAGTGAGAGCATGCAGAGCTGGGGCCTCAGGGAGGTGGTTAGG

< S’alpha_1

< S’mu_1

TAGTGCTGAAGACAGGACTGTGGAGACACCTTAGAAGGACAGATTCTGTTCCGAATCACCGATGCGGCGTCAG

||||||||||||||||||||||||||||||||||||||||||||||||||||||||

TAGTGCTGAAGACAGGACTGTGGAGACACCTTAGAAGGACAGATTCTGTTCCGAATTGACAGCCGTCATCCCAGAGTGAGAGCATGCAGAGCTGGGGCCTCAGGGAGGTGGT

||||||||||||||||||||||||||||||||||||||||||||||||||||||||

GTGTGACAGCCGTCATCCCAGAGTGAGAGCATGCAGAGCTGGGGCCTCAGGGAGGTGGT

< S’alpha_1

< S’mu_1

AGACAGGACTGTGGAGACACCTTAGAAGGACAGATTCTGTTCCGAATCACCGATGCGGCGTCAG

||||| ||||||||||||||| |||||||||||||||||||||||||

AGACAAGACTGTGGAGACACCCTAGAAGGACAGATTCTGTTCCGAATTATGACAGCCGTCATCCCAGAGTGAGAGCATGCAGAGCTGGGGCCTCAGGGAGGTGGTT

|||||||||||||||||||||||||||||||||||||||||||||||||||||||||

GTGTGACAGCCGTCATCCCAGAGTGAGAGCATGCAGAGCTGGGGCCTCAGGGAGGTGGTT

< S’alpha_1

< S’mu_1

AGACAGGACTGTGGAGACACCTTAGAAGGACAGATTCTGTTCCGAATCACCGATGCGGCGTCAG

|||||||||||||||||||||||||||||||||||||||

AGACAGGACTGTGGAGACACCTTAGAAGGACAGATTCTGAGGGTCTGTGTCTGCCCGAAACTCCTGTGGTGACAGCCGTCATCCCAGAGTGAGAGC

||||||||||||||||||||||||||||

GTGTGACAGCCGTCATCCCAGAGTGAGAGC

< S’alpha_1

< S’mu_1

TAGTGCTGAAGACAGGACTGTGGAGACACCTTAGAAGGACAGATTCTGTTCCGAATCACCGATGCGGCGTCAG

|||||||||||||| |||||||||||||||||||||||||||||||||||||||||

TAGTGCTGAAGACAAGACTGTGGAGACACCTTAGAAGGACAGATTCTGTTCCGAATGACAGCCGTCATCCCAGAGTGAGAGCATGCAGAGCTGGGGCCTCAGGGAGGTGGTTA

||||||||||||||||||||||||||||||||||||||||||||||||||||||||||

GTGTGACAGCCGTCATCCCAGAGTGAGAGCATGCAGAGCTGGGGCCTCAGGGAGGTGGTTA

< S’alpha_1

< S’mu_1

TAGTGCTGAAGACAGGACTGTGGAGACACCTTAGAAGGACAGATTCTGTTCCGAATCACCGATGCGGCGTCAG

||||||||||||||||||||||||||||||||||||||||||||||||||||||||

TAGTGCTGAAGACAGGACTGTGGAGACACCTTAGAAGGACAGATTCTGTTCCGAATTGACAGCCGTCATCCCAGAGTGAGAGCATGCAGAGCTGGGGCCTCAGGGAGGTGGT

||||||||||||||||||||||||||||||||||||||||||||||||||||||||

GTGTGACAGCCGTCATCCCAGAGTGAGAGCATGCAGAGCTGGGGCCTCAGGGAGGTGGT

< S’alpha_1

< S’mu_1

ATCAGATAGTGCTGAAGACAGGACTGTGGAGACACCTTAGAAGGACAGATTCTGTTCCGAATCACCGATGCGGCGTCAG

|||||||||||||||||||| |||||||||||||||||||| ||||||||||

ATCAGATAGTGCTGAAGACAAGACTGTGGAGACACCTTAGACGGACAGATTCAGCGAAAAGATGGGGGTCTGTGAACCAGGAATCGGCCCTTCCTGGAACTTGACCCTG

||| ||||||||||||||||||||||||||||||| ||||||||||||||||||||||

GTGTGACAGCCGTCATCCCA...147 nt...AGGACGCAGTGAAAAGATGGGGGTCTGTGAACCAGGAATCGACCCTTCCTGGAACTTGACCCTG

< S’alpha_1

< S’mu_1

CAATCAGATAGTGCTGAAGACAGGACTGTGGAGACACCTTAGAAGGACAGATTCTGTTCCGAATCACCGATGCGGCGTCAG

||||||||||||||||||||| |||||||||||||||||||||||||||||

CAATCAGATAGTGCTGAAGACGAGACTGTGGAGACACCTTAGAAGGACAGATACAGCGAAAAGATGGGGGTCTGTGAACCAGGAATCGGCCCTTCCTGGAACTTGACCCT

||| ||||||||||||||||||||||||||||||| |||||||||||||||||||||

GTGTGACAGCCGTCATCCCA...147 nt...AGGACGCAGTGAAAAGATGGGGGTCTGTGAACCAGGAATCGACCCTTCCTGGAACTTGACCCT

< S’alpha_1

< S’mu_1

AAGAGAAAACTCAATCAGATAGTGCTGAAGACAGGACTGTGGAGACACCTTAGAAGGACAGATTCTGTTCCGAATCACCGATGCGGCGTCAG

|||||||||||||||||||||||||||||||||||||||||||||||||||||

AAGAGAAAACTCAATCAGATAGTGCTGAAGACAGGACTGTGGAGACACCTTAGTGACAGCCGTCATCCCAGAGTGAGAGCATGCAGAGCTGGGGCCTCAGGGAGGTGG

||||||||||||||||||||||||||||||||||||||||||||||||||||||||

GTGTGACAGCCGTCATCCCAGAGTGAGAGCATGCAGAGCTGGGGCCTCAGGGAGGTGG

< S’alpha_1

< S’mu_1

TAGTGCTGAAGACAGGACTGTGGAGACACCTTAGAAGGACAGATTCTGTTCCGAATCACCGATGCGGCGTCAG

||||||||||||||||||||||||||||||||||||||||||||||||||||||||

TAGTGCTGAAGACAGGACTGTGGAGACACCTTAGAAGGACAGATTCTGTTCCGAATTGACAGCCGTCATCCCAGAGTGAGAGCATGCAGAGCTGGGGCCTCAGGGAGGTGGT

||||||||||||||||||||||||||||||||||||||||||||||||||||||||

GTGTGACAGCCGTCATCCCAGAGTGAGAGCATGCAGAGCTGGGGCCTCAGGGAGGTGGT

< S’alpha_1

< S’mu_1

AGTGCTGAAGACAGGACTGTGGAGACACCTTAGAAGGACAGATTCTGTTCCGAATCACCGATGCGGCGTCAG

|||||||||||||||||||||||||||| |||||||||||||||||||||||||

AGTGCTGAAGACAGGACTGTGGAGACACG-TAGAAGGACAGATTCTGTTCCGAATTCGACAGCCGTCATCCCAGAGTGAGAGCATGCAGAGCTGGGGCCTCAGGGAGGTGGTT

||||||||||||||||||||||||||||||||||||||||||||||||||||||||

GTGTGACAGCCGTCATCCCAGAGTGAGAGCATGCAGAGCTGGGGCCTCAGGGAGGTGGTT

< S’alpha_1

**BJAB, S’μ-S’α junctions from blunt DSBs (WT Cas9, S’μ_1-S’α_I)**

< S’mu_1

GTGCTGAAGACAGGACTGTGGAGACACCTTAGAAGGACAGATTCTGTTCCGAATCACCGATGCGGCGTCAG

|||||||||||| |||||||||||||||||||||||||||||||||||||||||

GTGCTGAAGACAAGACTGTGGAGACACCTTAGAAGGACAGATTCTGTTCCGAATTTCCACCACGTGAGGACGCAGTGAAAAGATGGGGGTCTGTGA

|||||||||||||||||||||||||||||||||||||||||||

TCTTTCCACCACGTGAGGACGCAGTGAAAAGATGGGGGTCTGTGA

< S’alpha_I

< S’mu_1

AGACAGGACTGTGGAGACACCTTAGAAGGACAGATTCTGTTCCGAATC-ACCGATGCGGCGTCAG

||||| |||||||||||||||||||||||||||||||||||||||||| |||

AGACAAGACTGTGGAGACACCTTAGAAGGACAGATTCTGTTCCGAATCCACCACGTGAGGACGCAGTGAAAAGATGGGGGTCTGTGAACCAGGAATCGAC

||||||||||||||||||||||||||||||||||||||||||||||||||||||

TCTTTCCACCACGTGAGGACGCAGTGAAAAGATGGGGGTCTGTGAACCAGGAATCGAC

< S’alpha_I

< S’mu_1

GTGCTGAAGACAGGACTGTGGAGACACCTTAGAAGGACAGATTCTGTTCCGAATCACCGATGCGGCGTCAG

|||||||||||| |||||||||||||||||||||||||||||||||||||||||

GTGCTGAAGACAAGACTGTGGAGACACCTTAGAAGGACAGATTCTGTTCCGAATTTCCACCACGTGAGGACGCAGTGAAAAGATGGGGGTCTGTGA

|||||||||||||||||||||||||||||||||||||||||||

TCTTTCCACCACGTGAGGACGCAGTGAAAAGATGGGGGTCTGTGA

< S’alpha_I

< S’mu_1

ACAGGACTGTGGAGACACCTTAGAAGGACAGATTCTGTTCCGAATCACCGATGCGGCGTCAG

||||||||||||||||||||||||||||||||||||||||||||||

ACAGGACTGTGGAGACACCTTAGAAGGACAGATTCTGTTCCGAATCTTCCACCATGTGAGGACGCAGTGAAAAGATGGGGGTCTGTGAACCAGGAATC

|||||||| |||||||||||||||||||||||||||||||||||||||||||

TCTTTCCACCACGTGAGGACGCAGTGAAAAGATGGGGGTCTGTGAACCAGGAATC

< S’alpha_I

< S’mu_1

GACTGTGGAGACACCTTAGAAGGACAGATTCTGTTCCGAATC-ACCGATGCGGCGTCAG

|||||||||||||||||||||||||||||||||||||||||| |||

GACTGTGGAGACACCTTAGAAGGACAGATTCTGTTCCGAATCCACCACGTGAGGACGCAGTGAAAAGATGGGGGTCTGTGAACCAGGAATCGACCCTTCC

||||||||||||||||||||||||||||||||||||||||||||||||||||||||||||

TCTTTCCACCACGTGAGGACGCAGTGAAAAGATGGGGGTCTGTGAACCAGGAATCGACCCTTCC

< S’alpha_I

< S’mu_1

GTGCTGAAGACAGGACTGTGGAGACACCTTAGAAGGACAGATTCTGTTCCGAATCACCGATGCGGCGTCAG

|||||||||||| |||||||||||||||||||||||||||||||||||||||||

GTGCTGAAGACAAGACTGTGGAGACACCTTAGAAGGACAGATTCTGTTCCGAATTTCCACCACGTGAGGACGCAGTGAAAAGATGGGGGTCTGTGA

|||||||||||||||||||||||||||||||||||||||||||

TCTTTCCACCACGTGAGGACGCAGTGAAAAGATGGGGGTCTGTGA

< S’alpha_I

< S’mu_1

CTCAATCAGATAGTGCTGAAGACAGGACTGTGGAGACACCTTAGAAGGACAGATTCTGTTCCGAATCACCGATGCGGCGTCAG

|||||||||||||||||||||||||||||||||||||||||

CTCAATCAGATAGTGCTGAAGACAGGACTGTGGAGACACCTGAGGACGCAGTGAAAAGATGGGGGTCTGTGAACCAGGAATCGACCCTTCCTGGAA

||| ||||||||||||||||||||||||||||||||||||||||||||||||||||||||

TCTTTCCACCACGTGAGGACGCAGTGAAAAGATGGGGGTCTGTGAACCAGGAATCGACCCTTCCTGGAA

< S’alpha_I

< S’mu_1

AGACAGGACTGTGGAGACACCTTAGAAGGACAGATTCTGTTCCGAATCACCGATGCGGCGTCAG

|||||||||||||||||||||||||||||||||||||||||||||||

AGACAGGACTGTGGAGACACCTTAGAAGGACAGATTCTGTTCCGAATTTCCACCATGTGAGGACGCAGTGAAAAGATGGGGGTCTGTGAACCAGGAAT

||||||||| ||||||||||||||||||||||||||||||||||||||||||

TCTTTCCACCACGTGAGGACGCAGTGAAAAGATGGGGGTCTGTGAACCAGGAAT

< S’alpha_I

< S’mu_1

AGACAGGACTGTGGAGACACCTTAGAAGGACAGATTCTGTTCCGAATCACCGATGCGGCGTCAG

| |||||| ||||||||||||||||||||||| ||||||||||||||

ARACAGGAYTGTGGAGACACCTTAGAAGGACARATTCTGTTCCGAATTCCACCATGTGAGGACGCAGCGAAAAGATGGGGGTCTGTGAACCAGGAATCG

|||||||| |||||||||||| |||||||||||||||||||||||||||||||

TCTTTCCACCACGTGAGGACGCAGTGAAAAGATGGGGGTCTGTGAACCAGGAATCG

< S’alpha_I

< S’mu_1

AGACAGGACTGTGGAGACACCTTAGAAGGACAGATTCTGTTCCGAATCACCGATGCGGCGTCAG

||||| ||||||||||||||||||||||||||||||||||||||||||

AGACAAGACTGTGGAGACACCTTAGAAGGACAGATTCTGTTCCGAATCTCCACCACGTGAGGACGCAGTGAAAAGATGGGGGTCTGTGAACCAGGAATCGACCCTTCC

||||||||||||||||||||||||||||||||||||||||||||||||||||||||||||

TCTTTCCACCACGTGAGGACGCAGTGAAAAGATGGGGGTCTGTGAACCAGGAATCGACCCTTCC

< S’alpha_I

< S’mu_1

AGACAGGACTGTGGAGACACCTTAGAAGGACAGATTCTGTTCCGAATCACCGATGCGGCGTCAG

|||||||||||||||||||||||||||||||||||||||||||||||

AGACAGGACTGTGGAGACACCTTAGAAGGACAGATTCTGTTCCGAATTTCCACCATGTGAGGACGCAGTGAAAAGATGGGGGTCTGTGAACCAGGAAT

||||||||| ||||||||||||||||||||||||||||||||||||||||||

TCTTTCCACCACGTGAGGACGCAGTGAAAAGATGGGGGTCTGTGAACCAGGAAT

< S’alpha_I

< S’mu_1

AGACAGGACTGTGGAGACACCTTAGAAGGACAGATTCTGTTCCGAATCACCGATGCGGCGTCAG

|||||||||||||||||||||||||||||||||||||||||||||||||

AGACAGGACTGTGGAGACACCTTAGAAGGACAGATTCTGTTCCGAATCATTCCACCATGTGAGGACGCAGCGAAAAGATGGGGGTCTGTGAACCAGGAATCG

|||||||| |||||||||||| |||||||||||||||||||||||||||||||

TCTTTCCACCACGTGAGGACGCAGTGAAAAGATGGGGGTCTGTGAACCAGGAATCG

< S’alpha_I

< S’mu_1

AGACAGGACTGTGGAGACACCTTAGAAGGACAGATTCTGTTCCGAATCACCGATGCGGCGTCAG

||||| |||||||||||||||||||||||||||||||||||||||||

AGACAAGACTGTGGAGACACCTTAGAAGGACAGATTCTGTTCCGAATTTCCACCACGTGAGGACGCAGTGAAAAGATGGGGGTCTGTGAACCAGGAATCGA

|||||||||||||||||||||||||||||||||||||||||||||||||||||||

TCTTTCCACCACGTGAGGACGCAGTGAAAAGATGGGGGTCTGTGAACCAGGAATCGA

< S’alpha_I

< S’mu_1

GACTGTGGAGACACCTTAGAAGGACAGATTCTGTTCCGAATCACCGATGCGGCGTCAG

||||||||||||||||||||||||||||||||||||||||||||||||||||||

GACTGTGGAGACACCTTAGAAGGACAGATTCTGTTCCGAATCACCGATGCGGCGAGGACGCAGTAAAAAGATGGGGGTCTGTGAACCAGGAATCGACCCTTCCTGGAA

||||||||||| |||||||||||||||||||||||||||||||||||||||||||

TCTTTCCACCACGTGAGGACGCAGTGAAAAGATGGGGGTCTGTGAACCAGGAATCGACCCTTCCTGGAA

< S’alpha_I

< S’mu_1

AGACAGGACTGTGGAGACACCTTAGAAGGACAGATTCTGTTCCGAATCACCGATGCGGCGTCAG

||||| |||||||||||||||||||||||| ||||||||||||||||

AGACAAGACTGTGGAGACACCTTAGAAGGAGAGATTCTGTTCCGAATTCCACCACGTGAGGACGCAGTGAAAAGATGGGGGTCTGTGAACCAGGGATCGACC

|||||||||||||||||||||||||||||||||||||||||||||||| |||||||

TCTTTCCACCACGTGAGGACGCAGTGAAAAGATGGGGGTCTGTGAACCAGGAATCGACC

< S’alpha_I

< S’mu_1

AGACAGGACTGTGGAGACACCTTAGAAGGACAGATTCTGTTCCGAATCACCGATGCGGCGTCAG

||||| |||||||||||||||||||||||||||||||||||||||||

AGACAAGACTGTGGAGACACCTTAGAAGGACAGATTCTGTTCCGAATTTCCACCACGTGAGGACGCAGTGAAAAGATGGGGGTCTGTGAACCAGGAATCGA

|||||||||||||||||||||||||||||||||||||||||||||||||||||||

TCTTTCCACCACGTGAGGACGCAGTGAAAAGATGGGGGTCTGTGAACCAGGAATCGA

< S’alpha_I

< S’mu_1

AGACAGGACTGTGGAGACACCTTAGAAGGACAGATTCTGTTCCGAATCACCGATGCGGCGTCAG

||||| ||||||||||||||||||||||||||||||||||||||||||

AGACAAGACTGTGGAGACACCTTAGAAGGACAGATTCTGTTCCGAATCTTCCACCACGTGAGGACGCAGTGAAAAGATGGGGGTCTGTGAACCAGGAATCGACC

||||||||||||||||||||||||||||||||||||||||||||||||||||||||

TCTTTCCACCACGTGAGGACGCAGTGAAAAGATGGGGGTCTGTGAACCAGGAATCGACC

< S’alpha_I

< S’mu_1

AGACAGGACTGTGGAGACACCTTAGAAGGACAGATTCTGTTCCGAATCACCGATGCGGCGTCAG

||||| |||||||||||||||||||||||||||||||||||||||||

AGACAAGACTGTGGAGACACCTTAGAAGGACAGATTCTGTTCCGAATTTCCACCACGTGAGGACGCAGTGAAAAGATGGGGGTCTGTGAACCAGGAATCGA

|||||||||||||||||||||||||||||||||||||||||||||||||||||||

TCTTTCCACCACGTGAGGACGCAGTGAAAAGATGGGGGTCTGTGAACCAGGAATCGA

< S’alpha_I

< S’mu_1

AGACAGGACTGTGGAGACACCTTAGAAGGACAGATTCTGTTCCGAATCACCGATGCGGCGTCAG

|||||||||||||||||||||||||||||||||||||||||||||||||

AGACAGGACTGTGGAGACACCTTAGAAGGACAGATTCTGTTCCGAATCATTCCACCATGTGAGGACGCAGCGAAAAGATGGGGGTCTGTGAACCAGGAATCG

|||||||| |||||||||||| |||||||||||||||||||||||||||||||

TCTTTCCACCACGTGAGGACGCAGTGAAAAGATGGGGGTCTGTGAACCAGGAATCG

< S’alpha_I

< S’mu_1

CAATCAGATAGTGCTGAAGACAGGACTGTGGAGACACCTTAGAAGGACAGATTCTGTTCCGAATCACCGATGCGGCGTCAG

|||||||||||| |||||||||||||||||||||||||||||||||||||||||

CAATCAGATAGTTCTGAAGACAGGACTGTGGAGACACCTTAGAAGGACAGATTCCACCATGTGAGGACGCAGCGAAAAGATGGGGGTCTGTGAACCAGGAACCGGCCC

|||||||| |||||||||||| |||||||||||||||||||||||||||| || |||

TCTTTCCACCACGTGAGGACGCAGTGAAAAGATGGGGGTCTGTGAACCAGGAATCGACCC

< S’alpha_I

**BJAB, S’μ-S’α junctions from 5’DSBs, short overhangs (Cas9 D10A, S’μ_1 + S’μ_2 + S’α_1 + S’α_2)**

< S’mu_1 S’mu_2 >

GAAGACAGGACTGTGGAGACACCTTAGAAGGACAGATTCTGTTCCGAATCACCGATGCGGCGTCAGCAGGACTGGCCTAGCGGAGGCTCT

||||||||||||||||||||||||||||||||||||||||||||||||||

GAAGACAGGACTGTGGAGACACCTTAGAAGGACAGATTCTGTTCCGAATCTTCATCCCAGAGTGAGAGCATGCAGAGCTGGGGCCTCAGGGAGGTGGTTAGGGCCTGAG

||||||||||||||||||||||||||||||||||||||||||||||||||||||||||

GTGTGACAGCCGTCATCCCAGAGTGAGAGCATGCAGAGCTGGGGCCTCAGGGAGGTGGTTAGGGCCTGAG

< S’alpha_1 S’alpha_2 >

< S’mu_1 S’mu_2 >

CACCTTAGAAGGACAGATTCTGTTCCGAATCACCGATGCGGCGTCAGCAGGACTGGCCTAGCGGAGGCTCT

|||||||||||||||||||||||||||||||

CACCTTAGAAGGACAGATTCTGTTCCGAATCCAGAGTGAGAGCATGCAGAGCTGGGGCCTCAGGGAGGTGGTTTCATCCCAGAGTGAGAGCATGCAGAGCTGGGGC

|||||||||||||||||||||||||||||||||

GTGTGACAGCCGTCATCCCAGAGTGAGAGCATGCAGAGCTGGGGC

< S’alpha_1

< S’mu_1 S’mu_2 >

GAGACACCTTAGAAGGACAGATTCTGTTCCGAATCACCGATGCGGCGTCAGCAGGACTGGCCTAGCGGAGGCTCT

|||||||||||||||||||||||||||||||||||||||||||||||||||||||

GAGACACCTTAGAAGGACAGATTCTGTTCCGAATCACCGATGCGGCGTCAGCAGGGGGAACCCTCAAAAATGTGATTCGGTCCCTAATCTCACTAGAGACCCTAAAGAA

|||||||||||||||||||||||||||||||||||||||||||||||||||||||||

GTGTGACAGCCGTCATCCCA...42 nt...GGCCTGAGGGGGAACCCTCAAAAATGTGATTCGGTCCCTAATCTCACTAGAGACCCTAAAGAA

< S’alpha_1 S’alpha_2 >

< S’mu_1 S’mu_2 >

GATAGTGCTGAAGACAGGACTGTGGAGACACCTTAGAAGGACAGATTCTGTTCCGAATCACCGATGCGGCGTCAGCAGGACTGGCCTAGCGGAGGCTCT

|||||||||||||||| ||||||||||||| ||||||||||||||||||||||

GATAGTGCTGAAGACAAGACTGTGGAGACA-CTTAGAAGGACAGATTCTGTTCTGCCTCAGAGTGAGAGCATGCAGAGCTGGGGCCTCGGGGAGGTGGTTAGGG

|||||||||||||||||||||||||||||| |||||||||||||||

GTGTGACAGCCGTCATCCCAGAGTGAGAGCATGCAGAGCTGGGGCCTCAGGGAGGTGGTTAGGG

< S’alpha_1 S’alpha_2 >

< S’mu_1 S’mu_2 >

GCTGAAGACAGGACTGTGGAGACACCTTAGAAGGACAGATTCTGTTCCGAATCACCGATGCGGCGTCAGCAGGACTGGCCTAGCGGAGGCTCT

|||||||||||||||||||||||| ||||| ||||||||||||||||||||||||||

GCTGAAGACAGGACTGTGGAGACA-CTTAGGAGGACAGATTCTGTTCCGAATCACCGTCATCCCAGAGTGAGAGCATGCAGAGCTGGGGCCTCAGGGAGGTGGTTAGGGC

||||||||||||||||||||||||||||||||||||||||||||||||||||||||

GTGTGACAGCCGTCATCCCAGAGTGAGAGCATGCAGAGCTGGGGCCTCAGGGAGGTGGTTAGGGC

< S’alpha_1 S’alpha_2 >

< S’mu_1 S’mu_2 >

ACTGTGGAGACACCTTAGAAGGACAGATTCTGTTCCGAATCACCGATGCGGCGTCAGCAGGACTGGCCTAGCGGAGGCTCT

|||||||||||||||||||||||||||||||||||||||||||||||||||||||||

ACTGTGGAGACACCTTAGAAGGACAGATTCTGTTCCGAATCACCGATGCGGCGTCAGGGAGGTGGTTAGGGCCTGAGGGGGAACACTCAAAAATGTGACTCGGTCCC

||| ||||||||||||||||||||||||||||||| ||||||||||||| ||||||||

GTGTGACAGCCGTCATCCCAGAGTGAGAGCATGCAGAGCTGGGGCCTCAGGGAGGTGGTTAGGGCCTGAGGGGGAACCCTCAAAAATGTGATTCGGTCCC

< S’alpha_1 S’alpha_2 >

S’mu_2 >

ATAAGAGAAAACTCAATCAGATAGTGCTGAAGACAGGACTGTGGAGACACCTTAGAA...41 nt...ACTGGCCTAGCGGAGGCTCT

|||||||||||||||||||||||||||||| |||||||||||||||| |||

ATAAGAGAAAACTCAATCAGATAGTGCTGAGGACAGGACTGTGGAGA-ACCCTCAAAAATGTGATTCGGTCCCTAATCTCACTAGAGACCCTAAAGAAAAGGGAC

|| |||||||||||||||||||||||||||||||||||||||||||||||||||||||||

GTGTGACAGCCGTCATCCCA...47 nt...GAGGGGGA-ACCCTCAAAAATGTGATTCGGTCCCTAATCTCACTAGAGACCCTAAAGAAAAGGGAC

< S’alpha_1

< S’mu_1 S’mu_2 >

CTTAGAAGGACAGATTCTGTTCCGAATCACCGATGCGGCGTCAGCAGGACTGGCCTAGCGGAGGCTCT

||||||||||||||||||||||||||||||||||| |||||||||

CTTAGAAGGACAGATTCTGTTCCGAATCACCGATGTGGCGTCAGCGGGACTGGCCTAGCTCATCCCAGAGTGAGAGCATGCAGAGCTGGGGCCTCAGGGAG

||||||||||||||||||||||||||||||||||||||||||

GTGTGACAGCCGTCATCCCAGAGTGAGAGCATGCAGAGCTGGGGCCTCAGGGAG

< S’alpha_1 S’alpha_2 >

S’mu_2 >

GGTGGCATAAGAGAAAACTCAATCAGATAGTGCTGAAGACAGGACTGTGGAGACACCTT...45 nt...ACTGGCCTAGCGGAGGCTCT

|||||||||||||||||||||||||||||||||||||||||||||||||||||

GGTGGCATAAGAGAAAACTCAATCAGATAGTGCTGAAGACAGGACTGTGGAGAGCCCTCAAAAATGTGATTCGGTCCCTAATCTCACTAGAGACCCTAAAGAAAAGGG

||||||||||||||||||||||||||||||||||||||||||||||||||||||

GTGTGACAGCCGTCATCCCA...50 nt...GGGGAACCCTCAAAAATGTGATTCGGTCCCTAATCTCACTAGAGACCCTAAAGAAAAGGG

< S’alpha_1

< S’mu_1 S’mu_2 >

GACTGTGGAGACACCTTAGAAGGACAGATTCTGTTCCGAATCACCGATGCGGCGTCAGCAGGACTGGCCTAGCGGAGGCTCT

|||||||||||||||||||||||||||||||||||||||| |||||||||||||

GACTGTGGAGACACCTTAGAAGGACAGATTCTGTTCCGAACCACCGATGCGGCGGTGGTTAGGGCCCGAGGGGGAACTCTCAAAAATGTGACTCGGTCCCTGATCTC

||||||||||||| |||||||||| ||||||||||||| ||||||||| ||||| GTGTGACAGCCGTCATCCCAGAGTGAGAGCATGCAGAGCTGGGGCCTCAGGGAGGTGGTTAGGGCCTGAGGGGGAACCCTCAAAAATGTGATTCGGTCCCTAATCTC

< S’alpha_1 S’alpha_2 >

< S’mu_1 S’mu_2 >

GCGTCAGCAGGACTGGCCTAGCGGAGGCTCT

||||||||||||||||||||||||||

GCGTCAGCAGGACTGGCCTAGCGGAGCTGGGGCCTCAGGGAGGTGGTTAGGGCCGGACTGGCCTAGCGGAGCTGGGGGAACACTCAAAAATGTGACTCGGTCCCTGATC

|||||||| ||||||||||||| ||||||||| |||

GTGTGACAGCCGTCATCCCAGAGTGAGAGCATGCAGAGCTGGGGCCTCAGGGAGGTGGTTAGGGCCTGAGGGGGAACCCTCAAAAATGTGATTCGGTCCCTAATC

< S’alpha_1 S’alpha_2 >

S’mu_2 >

AGTTTTAGGTAAAATGTGCATCATTATCCTGAATTATTTCAGTTAAGCATGTTAGTTG...103 nt...ACTGGCCTAGCGGAGGCTCT

||||||||||||||||||||||||||||||||||||||||||||||||||||

AGTTTTAGGTAAAATGTGCATCATTATCCTGAATTATTTCAGTTAAGCATGTGAGGACGCAGCGAAAAGATGGGGGTCTGTGAACCAGGAATCGGCCCTTCCTGGAA

|| |||||||||||| ||||||||||||||||||||||||||||||| ||||||||||||

GTGTGACAGCCGTCATCCCA...135 nt...TTCCACCACGTGAGGACGCAGTGAAAAGATGGGGGTCTGTGAACCAGGAATCGACCCTTCCTGGAA

< S’alpha_1

< S’mu_1 S’mu_2 >

TAGTGCTGAAGACAGGACTGTGGAGACACCTTAGAAGGACAGATTCTGTTCCGAATCACCGATGCGGCGTCAGCAGGACTGGCCTAGCGGAGGCTCT

||||||||||||||||||||||||||||||||||||||||||||||||||||

TAGTGCTGAAGACAGGACTGTGGAGACACCTTAGAAGGACAGATTCTGTTCCTAACGTGAGGGGGAACACTCAAAAATGTGACTCGGTCCCTGATCTCACTAGAGA

||||||||||| ||||||||||||| ||||||||| |||||||||||||

GTGTGACAGCCGTCATCCCA...40 nt...AGGGCCTGAGGGGGAACCCTCAAAAATGTGATTCGGTCCCTAATCTCACTAGAGA

< S’alpha_1 S’alpha_2 >

< S’mu_1 S’mu_2 >

AGACACCTTAGAAGGACAGATTCTGTTCCGAATCACCGATGCGGCGTCAGCAGGACTGGCCTAGCGGAGGCTCT

|||||| ||||||||||||||||||||||||||||||||||||||

AGACACTTTAGAAGGACAGATTCTGTTCCGAATCACCGATGCGGCTTAGGGCCTGAGGGGGAACCCTCAAAAATGTGATTCGGTCCCTAATCTCACTAGAG

||||||||||||||||||||||||||||||||||||||||||||||||||||||||

GTGTGACAGCCGTCATCCCA...32 nt...AGGTGGTTAGGGCCTGAGGGGGAACCCTCAAAAATGTGATTCGGTCCCTAATCTCACTAGAG

< S’alpha_1 S’alpha_2 >

< S’mu_1 S’mu_2 >

ATCACCGATGCGGCGTCAGCAGGACTGGCCTAGCGGAGGCTCT

||||||||||||||||||||||||

ATCACCGATGCGGCGTCAGCAGGAGCTCTGCATGCTCTCACTCTGGGATGCGGCGTCAGCAGGAGCTCTGCATGCTCACACTCGAACACTCAAAAATGTGACTCGGTCCCT

|||| ||||||||||||| |||||||||

GTGTGACAGCCGTCATCCCA...47 nt...GAGGGGGAACCCTCAAAAATGTGATTCGGTCCCT

< S’alpha_1

< S’mu_1 S’mu_2 >

ACAGGACTGTGGAGACACCTTAGAAGGACAGATTCTGTTCCGAATCACCGATGCGGCGTCAGCAGGACTGGCCTAGCGGAGGCTCT

||||||||||||||||||||||||||||||||||||||||||||||||

ACAGGACTGTGGAGACACCTTAGAAGGACAGATTCTGTTCCGAATCACTCAAAAATGTGACTCGGTCCCTGATCTCACTAGAGACCCAAAAGAAAAGGGA

||||||||||||| ||||||||| |||||||||||||||| ||||||||||||

GTGTGACAGCCGTCATCCCA...52 nt...GGAACCCTCAAAAATGTGATTCGGTCCCTAATCTCACTAGAGACCCTAAAGAAAAGGGA

< S’alpha_1

< S’mu_1 S’mu_2 >

TCTGTTCCGAATCACCGATGCGGCGTCAGCAGGACTGGCCTAGCGGAGGCTCT

||||||||||||||||||||||||| ||||

TCTGTTCCGAATCACCGATGCGGCGACAGCCGTCATCCCAGAGTGAGAGCGCGTAGAGCTGGGGTCATCCCAGAGTGAGAGCATGCAGAGCTGGGGC

||||||||||||||||||||||||||||||||||

GTGTGACAGCCGTCATCCCAGAGTGAGAGCATGCAGAGCTGGGGC

< S’alpha_1

S’mu_2 >

GGCATAAGAGAAAACTCAATCAGATAGTGCTGAAGACAGGACTGTGGAGACACCTTAGaaggac...37 nt...actggcctagcggaggctct

||||||||||||||||||||||||||||||||||||||||||||||||||||||||||

GGCATAAGAGAAAACTCAATCAGATAGTGCTGAAGACAGGACTGTGGAGACACCTTAGCTCAGGGAGGTGGTTAGGGCCTGAGGGGGAACACTCAAAAATGTGACTCGGTCCCT

|||||||||||||||||||||||||||||||| ||||||||||||| |||||||||

GTGTGACAGCCGTCATCCCAGAGTGAGAGCATGCAGAGCTGGGGCCCTCAGGGAGGTGGTTAGGGCCTGAGGGGGAACCCTCAAAAATGTGATTCGGTCCCT

< S’alpha_1 S’alpha_2 >

< S’mu_1 S’mu_2 >

GTTCCGAATCACCGATGCGGCGTCAGCAGGACTGGCCTAGCGGAGGCTCT

||||||||||||||||||||||||||||||||||||||||||||||||||

GTTCCGAATCACCGATGCGGCGTCAGCAGGACTGGCCTAGCGGAGGCTCTGATCTCACTAGAGACCCAAAAGAAAAGGGACCCCAGAGAGCAGCCCTGCCCTCTTT

|||||||||||||||| ||||||||||||||||||||||||||||||||||||||

GTGTGACAGCCGTCATCCCA...76 nt...TCCCTAATCTCACTAGAGACCCTAAAGAAAAGGGACCCCAGAGAGCAGCCCTGCCCTCTTT

< S’alpha_1

< S’mu_1 S’mu_2 >

AAACTCAATCAGATAGTGCTGAAGACAGGACTGTGGAGACACCTTAGAAGGACAGattctgttccgAATCACCGATGCGGCGTCAGcaggactggcctagcggaggctct

||||||||||||||||||||||||||||||||| |||||||| ||||| ||||||

AAACTCAATCAGATAGTGCTGAAGACAGGACTGAGGAGACACTTTAGATGGACAGTGAGAGCATGCAGAGCTGGGGCCTCAGGGAGGTGGTTAGGGCGTGAGGGGGAACC

|||||||||||||||||||||||||||||||||||||||||||| ||||||||||||

GTGTGACAGCCGTCATCCCAGAGTGAGAGCATGCAGAGCTGGGGCCTCAGGGAGGTGGTTAGGGCCTGAGGGGGAACC

< S’alpha_1 S’alpha_2 >

< S’mu_1 S’mu_2 >

TCAGATAGTGCTGAAGACAGGACTGTGGAGACACCTTAGAAGGACAGATTCTGTTCCGAATCACCGATGCGGCGTCAGCAGGACTGGCCTAGCGGAGGCTCT

|||||||||||||||||||||||||||||||||||||||||||||||||||||

TCAGATAGTGCTGAAGACAGGACTGTGGAGACACCTTAGAAGGACAGATTCTGGGGCCTCAGGGAGGTGGTTAGGGCCTGAGGGGGAACCCTCAAAAATGTGATT

|||||||||||||||||||||||||||||||||||||||||||||||||||||||

GTGTGACAGCCGTCATCCCAGAGTGAGAGCATGCAGAGCTGGGGCCTCAGGGAGGTGGTTAGGGCCTGAGGGGGAACCCTCAAAAATGTGATT

< S’alpha_1 S’alpha_2 >

S’mu_2 >

AAAATGTGCATCATTATCCTGAATTATTTCAGTTAAGCATGTTAGTTGgtggca...97 nt...actggcctagcggaggctct

|||| ||||||||||||| |||||||||||||||||||||||||||||

AAAACGTGCATCATTATCGTGAATTATTTCAGTTAAGCATGTTAGTTGCATGCAGAGCTGGGGCCTCAGGGAGGTGGTTAGGG-CTGAGGGGGAACACTCA

|||||||||||||||||||||||||||||||||||| |||||||||||| ||||

GTGTGACAGCCGTCATCCCAGAGTGAGAGCATGCAGAGCTGGGGCCTCAGGGAGGTGGTTAGGGCCTGAGGGGGAACCCTCA

< S’alpha_1 S’alpha_2 >

< S’mu_1 S’mu_2 >

CAATCAGATAGTGCTGAAGACAGGACTGTGGAGACACCTTAGAAGGACAGATTCTGTTCCGAATCACCGATGCGGCGTCAGCAGGACTGGCCTAGCGGAGGCTCT

|||||||||||||||||||||||||||||||||||||||||||||||||||||||||

CAATCAGATAGTGCTGAAGACAGGACTGTGGAGACACCTTAGAAGGACAGATTCTGTGACTCGGTCCCTGATCTCACTAGAGACCCAAAAGAAAAGGGACCCCAGAGAGCAGCC

||||| ||||||||| |||||||||||||||| |||||||||||||||||||||||||||

GTGTGACAGCCGTCATCCCA...60 nt...CAAAAATGTGATTCGGTCCCTAATCTCACTAGAGACCCTAAAGAAAAGGGACCCCAGAGAGCAGCC

< S’alpha_1

< S’mu_1 S’mu_2 >

GATAGTGCTGAAGACAGGACTGTGGAGACACCTTAGAAGGACAGATTCTGTTCCGAATCACCGATGCGGCGTCAGCAGGACTGGCCTAGCGGAGGCTCT

|||||||||||||||||||||||||||||||||||||||||||||||| ||||||||||

GATAGTGCTGAAGACAGGACTGTGGAGACACCTTAGAAGGACAGATTCCGTTCCGAATCATCCCAGAGTGAGAGCATGCAGAGCTGGGGCCTCAGGGAGGTGGTT

||||||||||||||||||||||||||||||||||||||||||||||||

GTGTGACAGCCGTCATCCCAGAGTGAGAGCATGCAGAGCTGGGGCCTCAGGGAGGTGGTT

< S’alpha_1 Salpha 5.2 >

< S’mu_1 S’mu_2 >

CCTTAGAAGGACAGATTCTGTTCCGAATCACCGATGCGGCGTCAGCAGGACTGGCCTAGCGGAGGCTCT

|||||||||||||||||||||||||||||||||||||||||||||

CCTTAGAAGGACAGATTCTGTTCCGAATCACCGATGCGGCGTCAGTGGgggggAACACTCAAAAATGTGACTCGGTCCCTGATCTCACTAGAGACCC

|||||||| ||||||||||||| ||||||||| ||||||||||||||||

GTGTGACAGCCGTCATCCCA...43 nt...GCCTGAGGGGGAACCCTCAAAAATGTGATTCGGTCCCTAATCTCACTAGAGACCC

< S’alpha_1

< S’mu_1 S’mu_2 >

TCAGATAGTGCTGAAGACAGGACTGTGGAGACACCTTAGAAGGACAGATTCTGTTCCGAATCACCGATGCGGCGTCAGCAGGACTGGCCTAGCGGAGGCTCT

|||||||||||||||||||||||||||||||||||||||||||||||||||||

TCAGATAGTGCTGAAGACAGGACTGTGGAGACACCTTAGAAGGACAGATTCTGAGAGCATGCAGAGCTGGGGCCCCAGGGAGGTGGTTAGGGCCTGAGGGGGAACCCT

||||||||||||||||||||||| |||||||||||||||||||||||||||||||||

GTGTGACAGCCGTCATCCCAGAGTGAGAGCATGCAGAGCTGGGGCCTCAGGGAGGTGGTTAGGGCCTGAGGGGGAACCCT

< S’alpha_1 Salpha 5.2 >

S’mu_2 >

TAAGTTTTAGGTAAAATGTGCATCATTATCCTGAATTATTTCAGTTAAGCATGTTAGTTG...103 nt...ACTGGCCTAGCGGAGGCTCT

||||||||||||||||||||||||||||||||||||||||||||||||||||||

TAAGTTTTAGGTAAAATGTGCATCATTATCCTGAATTATTTCAGTTAAGCATGTGAGGACGCAGCGAAAAGATGGGGGTCTGTGAACCAGGAATCGGCCCTTCCT

|||||||||||| ||||||||||||||||||||||||||||||| ||||||||

GTGTGACAGCCGTCATCCCA...138 nt...CACCACGTGAGGACGCAGTGAAAAGATGGGGGTCTGTGAACCAGGAATCGACCCTTCCT

< S’alpha_1

< S’mu_1 S’mu_2 >

CAGATTCTGTTCCGAATCACCGATGCGGCGTCAGCAGGACTGGCCTAGCGGAGGCTCT

||||||||||||||||||||||| ||||||

CAGATTCTGTTCCGAATCACCGACGCGGCGACAGCCGTCATCCCAGAGTGAGAGCACGCAGAGCTGGGGTCATCCCAGAGTGAGAGCATGCAGAGCTGGGGCC

|||||||||||||||||||||||||||||||||||

GTGTGACAGCCGTCATCCCAGAGTGAGAGCATGCAGAGCTGGGGCC

< S’alpha_1

< S’mu_1 S’mu_2 >

AGACAGGACTGTGGAGACACCTTAGAAGGACAGATTCTGTTCCGAATCACCGATGCGGCGTCAGCAGGACTGGCCTAGCGGAGGCTCT

||||||||||||||||||||||||||||||||| |||||||||||||||

AGACAGGACTGTGGAGACACCTTAGAAGGACAGTTTCTGTTCCGAATCAACACTCAAAAATGTGACTCGGTCCCTGATCTCACTAGAGACCCAAAAGAAAAGGGA

||| ||||||||||||| ||||||||| |||||||||||||||| ||||||||||||

GTGTGACAGCCGTCATCCCA...48 nt...AGGGGGAACCCTCAAAAATGTGATTCGGTCCCTAATCTCACTAGAGACCCTAAAGAAAAGGGA

< S’alpha_1

S’mu_2 >

GTTAGTTGGTGGCATAAGAGAAAACTCAATCAGATAGTGCTGAAGACAGGACTG...57 nt...ACTGGCCTAGCGGAGGCTCT

||||||||||||||||||||||||||||||||||||||||||||||||

GTTAGTTGGTGGCATAAGAGAAAACTCAATCAGATAGTGCTGAAGACAAATGTGATCCGGTCCCTAATCTTACTAGAGACCCTAAAGAAAAGGGACCCCAGA

||||||||| ||||||||||||| |||||||||||||||||||||||||||||||

GTGTGACAGCCGTCATCCCA...57 nt...CCTCAAAAATGTGATTCGGTCCCTAATCTCACTAGAGACCCTAAAGAAAAGGGACCCCAGA

< S’alpha_1

S’mu_2 >

TTTTAGGTAAAATGTGCATCATTATCCTGAATTATTTCAGTTAAGCATGTTAGTTGGTGGCA...97 nt...ACTGGCCTAGCGGAGGCTCT

||||||||||||||||||||||||||||||||||||||||||||||||||||||||

TTTTAGGTAAAATGTGCATCATTATCCTGAATTATTTCAGTTAAGCATGTTAGTTGCATGCAGAGCTGGGGCCTCAGGGAGGTGGTTAGGGCCTGAGGGGGAAC

|||||||||||||||||||||||||||||||||||||||||||||||||

GTGTGACAGCCGTCATCCCAGAGTGAGAGCATGCAGAGCTGGGGCCTCAGGGAGGTGGTTAGGGCCTGAGGGGGAAC

< S’alpha_1 Salpha 5.2 >

< S’mu_1 S’mu_2 >

AGACAGGACTGTGGAGACACCTTAGAAGGACAGATTCTGTTCCGAATCACCGATGCGGCGTCAGCAGGACTGGCCTAGCGGAGGCTCT

||||| ||||||||||||||||||||||||||||||||||||||||||||

AGACAAGACTGTGGAGACACCTTAGAAGGACAGATTCTGTTCCGAATCACTCTGCATGCAGAGCTGGGGCCTCGGGGAGGTGGTTAGGGCCTGAGGGGGAAC

|||||||||||||||||||| ||||||||||||||||||||||||||||

GTGTGACAGCCGTCATCCCAGAGTGAGAGCATGCAGAGCTGGGGCCTCAGGGAGGTGGTTAGGGCCTGAGGGGGAAC

< S’alpha_1 S’alpha_2 >

< S’mu_1 S’mu_2 >

CAGATAGTGCTGAAGACAGGACTGTGGAGACACCTTAGAAGGACAGATTCTGTTCCGAATCACCGATGCGGCGTCAGCAGGACTGGCCTAGCGGAGGCTCT

||||||||||||||||||||||||||||||||||||||||||||||||||||| ||||

CAGATAGTGCTGAAGACAGGACTGTGGAGACACCTTAGAAGGACAGATTCTGTCCCGACTCCAGGGCCAGCATCGCTGTCGGGATCTCTCCAACGCAACGGCCAGCTCTC

||||||||||||||||||||||||| |||||||||||||||||||||||||||||||

GTGTGACAGCCGTCATCCCA...653 nt...GCCTCCCCCGACTCCAGGGCCAGCATCGCTGGCGGGATCTCTCCAACGCAACGGCCAGCTCTC

< S’alpha_1

S’mu_2 >

GTGGCATAAGAGAAAACTCAATCAGATAGTGCTGAAGACAGGACTGTGGAGACACCTTAGAAG...40 nt...ACTGGCCTAGCGGAGGCTCT

|||||||||||||||||||||||||||||||||||||||| ||||||||||||||||

GTGGCATAAGAGAAAACTCAATCAGATAGTGCTGAAGACAAGACTGTGGAGACACCTCCCTCCTCCTCTGGCCCCACCTGCCTTGATCCGGCCTCACTGTCACTCCCTGG

||||||||||||||||| ||||||||||| |||||||||||||||||||||||||||

GTGTGACAGCCGTCATCCCA...434 nt...CAGGCTACCTCCCTCCTCCTCTGACCCCACCTGCCCTGATCCGGCCTCACTGTCACTCCCTGG

< S’alpha_1

**BJAB, S’μ-S’α junctions from 5’DSBs, short overhangs (Cas9 D10A, S’μ_1 + S’μ_2 + S’α_I + S’α_II)**

< S’mu_1 S’mu_2 >

TGGAGACACCTTAGAAGGACAGATTCTGTTCCGAATCACCGATGCGGCGTCAGCAGGACTGGCCTAGCGGAGGCTCT

|||||||||||||| ||| |||||||||||||||||||||||||||||

TGGAGACACCTTAGGAGGGCAGATTCTGTTCCGAATCACCGATGCGGCCCCATGGCCAGGGCATCTTCCATTCACGCCCACGCCTTCCAGGACTCCGCTG

||||||||||||||||||||||||||||||||||||||| ||||||||||||||||

TCTTTCCACCACGTGAGGAC...370 nt...GTGGCCCGGCCCCATGGCCAGGGCATCTTCCATTCACGCCCACGCTTTCCAGGACTCCGCTG

< S’alpha_I

< S’mu_1 S’mu_2 >

GTGGAGACACCTTAGAAGGACAGATTCTGTTCCGAATCACCGATGCGGCGTCAGCAGGACTGGCCTAGCGGAGGCTCT

|||||||||||||||||||||||||||||||||||||||||||||||| |||

GTGGAGACACCTTAGAAGGACAGATTCTGTTCCGAATCACCGATGCGGCGTCTGTGAACCAGGAATCGACCCTTCCTGGAACTTGACCCTGCTGCCATC

|||| || ||||||||||||||||||||||||||||||||||||||||||||||||||

TCTTTCCACCACGTGAGGACGCAGTGAAAAGATG-GGGGTCTGTGAACCAGGAATCGACCCTTCCTGGAACTTGACCCTGCTGCCATC

< S’alpha_I S’alpha_II >

< S’mu_1 S’mu_2 >

AGATAGTGCTGAAGACAGGACTGTGGAGACACCTTAGAAGGACAGATTCTGTTCCGAATCACCGATGCGGCGTCAGCAGGACTGGCCTAGCGGAGGCTCT

|||||||||||||||||||||||||||||||||||||||||||||||||||||

AGATAGTGCTGAAGACAGGACTGTGGAGACACCTTAGAAGGACAGATTCTGTTGACCCTGCTGCCATCCTGACCTTGAACTTCCAGCTTCGAGGACCGTGAG

|||||||||||||||||||||||||||||||||||||||||| ||||||||

TCTTTCCACCACGTGAGGAC...44 nt...TGGAACTTGACCCTGCTGCCATCCTGACCTTGAACTTCCAGCTTCGAGAACCGTGAG

< S’alpha_I

< S’mu_1 S’mu_2 >

ACCTTAGAAGGACAGATTCTGTTCCGAATCACCGATGCGGCGTCAGCAGGACTGGCCTAGCGGAGGCTCT

||||||||||||||||||||| ||||||||||||||||||||||||||

ACCTTAGAAGGACAGATTCTGCTCCGAATCACCGATGCGGCGTCAGCACGTGAGGACGCAGTGAAAAGATGGGGGTCTGTGAACCAGGAATCGACCCTTCC

|||||||||||||||||||||||||||||||||||||||||||||||||||||||

TCTTTCCACCACGTGAGGACGCAGTGAAAAGATGGGGGTCTGTGAACCAGGAATCGACCCTTCC

< S’alpha_I S’alpha_II >

S’mu_2 >

AGACAGGACTGTGGAGACACCTTAGAAGGACAGATT...32 nt...ACTGGCCTAGCGGAGGCTCT

||||||||||||||||||||||||||||||

AGACAGGACTGTGGAGACACCTTAGAAGGAAATGCATTTTATCTGAATTTGCCACTTAAAATTCAGGGGGTCTGTGAACCAGGAATCGACCC

|||||||||||||||||||||||||||

TCTTTCCACCACGTGAGGACGCAGTGAAAAGATGGGGGTCTGTGAACCAGGAATCGACCC

< S’alpha_I

< S’mu_1 S’mu_2 >

TTAGAAGGACAGATTCTGTTCCGAATCACCGATGCGGCGTCAGCAGGACTGGCCTAGCGGAGGCTCT

|||||||||||||||||||

TTAGAAGGACAGATTCTGTCCCCGGACTACCCAGAGCTGGGATGCGTGGCTTCTGCTGCCGGGCCGACTGGCTGCTCAGGCCAGGAATCGACCCTT

||||||||||||||||

TCTTTCCACCACGTGAGGACGCAGTGAAAAGATGGGGGTCTGTGAACCAGGAATCGACCCTT

< S’alpha_I S’alpha_II >

< S’mu_1 S’mu_2 >

GGAGACACCTTAGAAGGACAGATTCTGTTCCGAATCACCGATGCGGCGTCAGCAGGACTGGCCTAGCGGAGGCTCT

||||||||||||||||||||||||||||||||||||||||||||||||

GGAGACACCTTAGAAGGACAGATTCTGTTCCGAATCACCGATGCGGCGAAAAGATGGGGGTCTGTGAACCAGGAATCGACCCTTCCTGGAACTTGACCCTG

||||||||||||||||||||||||||||||||||||||||||||||||||||||

TCTTTCCACCACGTGAGGACGCAGTGAAAAGATGGGGGTCTGTGAACCAGGAATCGACCCTTCCTGGAACTTGACCCTG

< S’alpha_I S’alpha_II >

< S’mu_1 S’mu_2 >

TCTGTTCCGAATCACCGATGCGGCGTCAGCAGGACTGGCCTAGCGGAGGCTCT

||||||||||||||||||||||||||||||||||||||||||||||||

TCTGTTCCGAATCACCGATGCGGCGTCAGCAGGACTGGCCTAGCGGAGTCTGTGAACCAGGAATCGACCCTTCCTGGAACTTGACCCTGCTGCCATCCTGAC

|||||||||||||||||||||||||||||||||||||||||||||||||||||||

TCTTTCCACCACGTGAGGACGCAGTGAAAAGATGGGGGTCTGTGAACCAGGAATCGACCCTTCCTGGAACTTGACCCTGCTGCCATCCTGAC

< S’alpha_I S’alpha_II >

< S’mu_1 S’mu_2 >

CTGTGGAGACACCTTAGAAGGACAGATTCTGTTCCGAATCACCGATGCGGCGTCAGCAGGACTGGCCTAGCGGAGGCTCT

|||||||||||||||||||||||||||||||||||||||||||

CTGTGGAGACACCTTAGAAGGACAGATTCTGTTCCGAATCACCTGTGGGTCTGTGAACCAGGAATCGACCCTTCCTGGAACTTGACCCTGCTGCCATC

||||||||||||||||||||||||||||||||||||||||||||||||||||

TCTTTCCACCACGTGAGGACGCAGTGAAAAGATGGGGGTCTGTGAACCAGGAATCGACCCTTCCTGGAACTTGACCCTGCTGCCATC

< S’alpha_I S’alpha_II >

< S’mu_1 S’mu_2 >

CACCTTAGAAGGACAGATTCTGTTCCGAATCACCGATGCGGCGTCAGCAGGACTGGCCTAGCGGAGGCTCT

||||||||||||||||||||||||||||||||||||||||||||||||||

CACCTTAGAAGGACAGATTCTGTTCCGAATCACCGATGCGGCGTCAGCAGTGAAAAGATGGGGGTCTGTGAACCAGGAATCGACCCTTCCTGGAACTTGA

||||||||||||||||||||||||||||||||||||||||||||||||||||||

TCTTTCCACCACGTGAGGACGCAGTGAAAAGATGGGGGTCTGTGAACCAGGAATCGACCCTTCCTGGAACTTGA

< S’alpha_I S’alpha_II >

< S’mu_1 S’mu_2 >

GACTGTGGAGACACCTTAGAAGGACAGATTCTGTTCCGAATCACCGATGCGGCGTCAGCAGGACTGGCCTAGCGGAGGCTCT

|||||||||||||||||||||||||||||||||||||||||||||||||

GACTGTGGAGACACCTTAGAAGGACAGATTCTGTTCCGAATCACCGATGAACCAGGAATCGACCCTTCCTGGAACTTGACCCTGCTGCCATCCTGACCT

||||||||||||||||||||||||||||||||||||||||||||||||||||

TCTTTCCACCACGTGAGGACGCAGTGAAAAGATGGGGGTCTGTGAACCAGGAATCGACCCTTCCTGGAACTTGACCCTGCTGCCATCCTGACCT

< S’alpha_I S’alpha_II >

S’mu_2 >

ACTCAATCAGATAGTGCTGAAGACAGGACTGTGGAGACACCTTAGAAGGACAGATT...32 nt...ACTGGCCTAGCGGAGGCTCT

||||||||||||||||||||||||||||||||||||||||||||||||||

ACTCAATCAGATAGTGCTGAAGACAGGACTGTGGAGACACCTTAGAAGGAGATGGGGGTCTGTGAACCAGGAATCGACCCTTCCTGGAACTTGACCCTGCTG

|||||||||||||||||||||||||||||||||||||||||||||||||||||

TCTTTCCACCACGTGAGGACGCAGTGAAAAGATGGGGGTCTGTGAACCAGGAATCGACCCTTCCTGGAACTTGACCCTGCTG

< S’alpha_I S’alpha_II >

< S’mu_1 S’mu_2 >

CAGGACTGTGGAGACACCTTAGAAGGACAGATTCTGTTCCGAATCACCGATGCGGCGTCAGCAGGACTGGCCTAGCGGAGGCTCT

||||||||||||||||||||||||||||||||||||||||||||||||

CAGGACTGTGGAGACACCTTAGAAGGACAGATTCTGTTCCGAATCACCCAGGAATCGACCCTTCCTGGAACTTGACCCTGCTGCCATCCTGACCTTGAACT

||||||||||||||||||||||||||||||||||||||||||||||||||||||

TCTTTCCACCACGTGAGGACGCAGTGAAAAGATGGGGGTCTGTGAACCAGGAATCGACCCTTCCTGGAACTTGACCCTGCTGCCATCCTGACCTTGAACT

< S’alpha_I S’alpha_II >

< S’mu_1 S’mu_2 >

TTCTGTTCCGAATCACCGATGCGGCGTCAGCAGGACTGGCCTAGCGGAGGCTCT

||||||||||||||||||||||||||||||||

TTCTGTTCCGAATCACCGATGCGGCGTCAGCATGTCTGTGAACCAGGAATCGACCCTTCCTGGAACTTGACCCTGCTGCCATCCTG

|||||||||||||||||||||||||||||||||||||||||||||||||||||

TCTTTCCACCACGTGAGGAC.11 nt.ATGGGGGTCTGTGAACCAGGAATCGACCCTTCCTGGAACTTGACCCTGCTGCCATCCTG

< S’alpha_I S’alpha_II >

< S’mu_1 S’mu_2 >

AGATAGTGCTGAAGACAGGACTGTGGAGACACCTTAGAAGGACAGATTCTGTTCCGAATCACCGATGCGGCGTCAGCAGGACTGGCCTAGCGGAGGCTCT

||||||||||||||||| ||||||||||||||||||||||||||||||||

AGATAGTGCTGAAGACAAGACTGTGGAGACACCTTAGAAGGACAGATTCTTGACCCTGCTGCCATCCTGACCTTGAACTTCCAGCTTC

||||||||||||||||||||||||||||||||||||||||

TCTTTCCACCACGTGAGGAC...43 nt...CTGGAACTTGACCCTGCTGCCATCCTGACCTTGAACTTCCAGCTTC

< S’alpha_I

< S’mu_1 S’mu_2 >

GACTGTGGAGACACCTTAGAAGGACAGATTCTGTTCCGAATCACCGATGCGGCGTCAGCAGGACTGGCCTAGCGGAGGCTCT

|||||||||||||||||||||||||||||||||||||||||||||||

GACTGTGGAGACACCTTAGAAGGACAGATTCTGTTCCGAATCACCGAGATGGGGGTCTGTGAACCAGGAATCGACCCTTCCTGGAACTTGACCCTGCTGCC

|||||||||||||||||||||||||||||||||||||||||||||||||||||||

TCTTTCCACCACGTGAGGACGCAGTGAAAAGATGGGGGTCTGTGAACCAGGAATCGACCCTTCCTGGAACTTGACCCTGCTGCC

< S’alpha_I S’alpha_II >

< S’mu_1 S’mu_2 >

GATTCTGTTCCGAATCACCGATGCGGCGTCAGCAGGACTGGCCTAGCGGAGGCTCT

|||||||||||||||||||||||||||||||

GATTCTGTTCCGAATCACCGATGCGGCGTCAACTGAAAAGATGGGGGTCTGTGAACCAGGAATCGACCGGGGGTCTGTGAACCAGGAATCGACCCTTCCTG

|||||||||||||||||||||||||||||||||

TCTTTCCACCACGTGAGGACGCAGTGAAAAGATGGGGGTCTGTGAACCAGGAATCGACCCTTCCTG

< S’alpha_I S’alpha_II >

< S’mu_1 S’mu_2 >

TGTGGAGACACCTTAGAAGGACAGATTCTGTTCCGAATCACCGATGCGGCGTCAGCAGGACTGGCCTAGCGGAGGCTCT

||||||||||||||||||||||||||||||||||||||||||||||||

TGTGGAGACACCTTAGAAGGACAGATTCTGTTCCGAATCACCGATGCGTCTGTGAACCAGGAATCGACCCTTCCTGGAACTTGACCCTGCTGCCATCCTG

|||||||||||||||||||||||||||||||||||||||||||||||||||||

TCTTTCCACCACGTGAGGACGCAGTGAAAAGATGGGGGTCTGTGAACCAGGAATCGACCCTTCCTGGAACTTGACCCTGCTGCCATCCTG

< S’alpha_I S’alpha_II >

< S’mu_1 S’mu_2 >

TGTGGAGACACCTTAGAAGGACAGATTCTGTTCCGAATCACCGATGCGGCGTCAGCAGGACTGGCCTAGCGGAGGCTCT

|||||||||||||||||||||||||||||||||||||||||

TGTGGAGACACCTTAGAAGGACAGATTCTGTTCCGAATCACAGCAGAGGACGCAGTGAAAAGATGGGGGTCTGTGAACCAGGAATCGACCCTTCCTG

||||||||||||||||||||||||||||||||||||||||||||||||||||

TCTTTCCACCACGTGAGGACGCAGTGAAAAGATGGGGGTCTGTGAACCAGGAATCGACCCTTCCTG

< S’alpha_I S’alpha_II >

< S’mu_1 S’mu_2 >

TGGAGACACCTTAGAAGGACAGATTCTGTTCCGAATCACCGATGCGGCGTCAGCAGGACTGGCCTAGCGGAGGCTCT

||||||||||||||||||||||||||||||||||||||||||| |||||

TGGAGACACCTTAGAAGGACAGATTCTGTTCCGAATCACCGATCCGGCGGTCTGTGAACCAGGAATCGACCCTTCCTGGAACTTGACCCTGCTGCCATCCTG

||||||||||||||||||||||||||||||||||||||||||||||||||||||

TCTTTCCACCACGTGAGGACGCAGTGAAAAGATGGGGGTCTGTGAACCAGGAATCGACCCTTCCTGGAACTTGACCCTGCTGCCATCCTG

< S’alpha_I S’alpha_II >

S’mu_2 >

CTCAATCAGATAGTGCTGAAGACAGGACTGTGGAGACACCTTAGAAGGACAGATT...32 nt...ACTGGCCTAGCGGAGGCTCT

|||||||||||||||||||||||| ||||||||||||||||||||||||

CTCAATCAGATAGTGCTGAAGACAAGACTGTGGAGACACCTTAGAAGGAACCGACCCTTCCTGGAACTTGACCCTGCTGCCATCCTGACCTTGAACTTC

||||| ||||||||||||||||||||||||||||||||||||||||||||||||

TCTTTCCACCACGTGAGGAC...22 nt...TGAACCAGGAATCGACCCTTCCTGGAACTTGACCCTGCTGCCATCCTGACCTTGAACTTC

< S’alpha_I S’alpha_II >

< S’mu_1 S’mu_2 >

AAGGACAGATTCTGTTCCGAATCACCG-ATGCGGCGTCAGCAGGACTGGCCTAGCGGAGGCTCT

||||||||||||||||||||||||||| ||| || |||

AAGGACAGATTCTGTTCCGAATCACCGCATGGGG-GTCTGTGAACCAGGAATCGACCCTTCCTGGAACTTGACCCTGCTGCCATC

|||||| ||||||||||||||||||||||||||||||||||||||||||||||||||

TCTTTCCACCACGTGAGGAC...28 nt...ATGGGG-GTCTGTGAACCAGGAATCGACCCTTCCTGGAACTTGACCCTGCTGCCATC

< S’alpha_I S’alpha_II >

< S’mu_1 S’mu_2 >

ACTGTGGAGACACCTTAGAAGGACAGATTCTGTTCCGAATCACCGATGCGGCGTCAGCAGGACTGGCCTAGCGGAGGCTCT

|||||||||||||||||||||||||| ||||||||||||||| ||||||||

ACTGTGGAGACACCTTAGAAGGACAGGCTCTGTTCCGAATCACTGATGCGGCCCTTCCTGGAACTTGACCCTGCTGCCATCCTGACCTTGAACTTCCAGC

|||||||||||||||||||||||||||||||||||||||||||||||||

TCTTTCCACCACGTGAGGAC...31 nt...AATCGACCCTTCCTGGAACTTGACCCTGCTGCCATCCTGACCTTGAACTTCCAGC

< S’alpha_I S’alpha_II >

< S’mu_1 S’mu_2 >

GGACTGTGGAGACACCTTAGAAGGACAGATTCTGTTCCGAATC-ACCGATGCGGCGTCAGCAGGACTGGCCTAGCGGAGGCTCT

|||||||||||||||||| |||||||||||||||||||||||| ||| |||

GGACTGTGGAGACACCTTGGAAGGACAGATTCTGTTCCGAATCCACC-ATGTGAGGACGCAGTGAAAAGATGGGGGTCTGTGAACCAGGAAT

|||||| | ||||||||||||||||||||||||||||||||||||||||||

TCTTTCCACC-ACGTGAGGACGCAGTGAAAAGATGGGGGTCTGTGAACCAGGAAT

< S’alpha_I

< S’mu_1 S’mu_2 >

GTTCCGAATCACCGATGCGGCGTCAGCAGGACTGGCCTAGCGGAGGCTCT

|||||||||||||||||

GTTCCGAATCACCGATGAAAAGATGGGGGTAGTGAAAAGATGGCGCAGTGAAAAGATGGGGGTAGTGAAAAGATGGGGGTCTGTG

||||||||||||||||||||||

TCTTTCCACCACGTGAGGACGCAGTGAAAAGATGGGGGTCTGTG

< S’alpha_I

< S’mu_1 S’mu_2 >

ATAGTGCTGAAGACAGGACTGTGGAGACACCTTAGAAGGACAGATTCTGTTCCGAATCACCGATGCGGCGTCAGCAGGACTGGCCTAGCGGAGGCTCT

||||||||||||||||||||||||||||||||||||||||||||||

ATAGTGCTGAAGACAGGACTGTGGAGACACCTTAGAAGGACAGATTGACGCAGCGAAAAGATGGGGGTCTGTGAACCAGGAATCGGCCCTTCCTGGAACT

||||||| ||||||||||||||||||||||||||||||| ||||||||||||||

TCTTTCCACCACGTGAGGACGCAGTGAAAAGATGGGGGTCTGTGAACCAGGAATCGACCCTTCCTGGAACT

< S’alpha_I S’alpha_II >

< S’mu_1 S’mu_2 >

TAGTGCTGAAGACAGGACTGTGGAGACACCTTAGAAGGACAGATTCTGTTCCGAATCACCGATGCGGCGTCAGCAGGACTGGCCTAGCGGAGGCTCT

|||||||||||||||||||||||||||||||||||||||||||

TAGTGCTGAAGACAGGACTGTGGAGACACCTTAGAAGGACAGAATCGACCCTTCCTGGAACTTGACCCTGCTGCCATCCTGACCTTGAACTTCCAGCT

|||||||||||||||||||||||||||||||||||||||||||||||||||||||||

TCTTTCCACCACGTGAGGAC...24 nt...AACCAGGAATCGACCCTTCCTGGAACTTGACCCTGCTGCCATCCTGACCTTGAACTTCCAGCT

< S’alpha_I S’alpha_II >

< S’mu_1 S’mu_2 >

CCTTAGAAGGACAGATTCTGTTCCGAATCACCGATGCGGCGTCAGCAGGACTGGCCTAGCGGAGGCTCT

|||||||||||||||||||||||||||||||||||||||||||||||||||||

CCTTAGAAGGACAGATTCTGTTCCGAATCACCGATGCGGCGTCAGCAGGACTGAAAAGATGGGGGTCTGTGGACCAGGAATCGACCCTTCCTGGAACTTGAC

|||||||||||||||||||| ||||||||||||||||||||||||||||||

TCTTTCCACCACGTGAGGACGCAGTGAAAAGATGGGGGTCTGTGAACCAGGAATCGACCCTTCCTGGAACTTGAC

< S’alpha_I S’alpha_II >

< S’mu_1 S’mu_2 >

CACCTTAGAAGGACAGATTCTGTTCCGAATCACCGATGCGGCGTCAGCAGGACTGGCCTAGCGGAGGCTCT

|||||||||||||||||||||||||||||||||||||||||||||

CACCTTAGAAGGACAGATTCTGTTCCGAATCACCGATGCGGCGTCCAGTGAAAAGATGGGGGTCTGTGAACCAGGAATCGACCCTTCCTGGAACTTGACC

|||||||||||||||||||||||||||||||||||||||||||||||||||||||

TCTTTCCACCACGTGAGGACGCAGTGAAAAGATGGGGGTCTGTGAACCAGGAATCGACCCTTCCTGGAACTTGACC

< S’alpha_I S’alpha_II >

< S’mu_1 S’mu_2 >

CACCTTAGAAGGACAGATTCTGTTCCGAATCACCGATGCGGCGTCAGCAGGACTGGCCTAGCGGAGGCTCT

||||||||||||||||||||||||||||||||||||||||||||||||

CACCTTAGAAGGACAGATTCTGTTCCGAATCACCGATGCGGCGTCAGCGGGGGTCTGTGAACCAGGAATCGACCCTTCCTGGAACTTGACCCTGCTGCC

|||||||||||||||||||||||||||||||||||||||||||||||||||

TCTTTCCACCACGTGAGGACGCAGTGAAAAGATGGGGGTCTGTGAACCAGGAATCGACCCTTCCTGGAACTTGACCCTGCTGCC

< S’alpha_I S’alpha_II >

< S’mu_1 S’mu_2 >

GACTGTGGAGACACCTTAGAAGGACAGATTCTGTTCCGAATCACCGATGCGGCGTCAGCAGGACTGGCCTAGCGGAGGCTCT

||||||||||||||||||||||||||||||||||||||||||||||

GACTGTGGAGACACCTTAGAAGGACAGATTCTGTTCCGAATCACCGGGGGTCTGTG-AAAGATGGGGGTCTGTGAACCAGGAATCGACCCTTCCTGGAAC

||| |||||||||||||||||||||||||||||||||||||||||||

TCTTTCCACCACGTGAGGACGCAGTGAAAAGATGGGGGTCTGTGAACCAGGAATCGACCCTTCCTGGAAC

< S’alpha_I S’alpha_II >

**BJAB, S’μ-S’α junctions from 5’DSBs, long overhangs (Cas9 D10A, S’μ_1 + S’μ_2 + S’α_1 + S’α_3)**

< S’mu_1 S’mu_2 >

ACAGATTCTGTTCCGAATCACCGATGCGGCGTCAGCAGGACTGGCCTAGCGGAGGCTCT

||||||||||||||||||||| || |||||||||||||||||||||||| |||||

ACAGATTCTGTTCCGAATCACTGACGCGGCGTCAGCAGGACTGGCCTAGTGGAGGCAGCCGTCATCCCAGAGTGAGAGCATGCAGAGCTGGGGCCTCAGGGAGGTGGTT

||||||||||||||||||||||||||||||||||||||||||||||||||||||

GTGTGACAGCCGTCATCCCAGAGTGAGAGCATGCAGAGCTGGGGCCTCAGGGAGGTGGTT

< S’alpha_1

< S’mu_1 S’mu_2 >

CAGATTCTGTTCCGAATCACCGATGCGGCGTCAGCAGGACTGGCCTAGCGGAGGCTCT

||||||||||||||||||||||||||||||||||||||||||||||||||

CAGATTCTGTTCCGAATCACCGATGCGGCGTCAGCAGGACTGGCCTAGCGGTGACAGCCGTCATCCCAGAGTGAGAGCATGCAGAGCTGGGGCCTCAGGGAGGTGGTTA

|||||||||||||||||||||||||||||||||||||||||||||||||||||||||||

GTGTGACAGCCGTCATCCCAGAGTGAGAGCATGCAGAGCTGGGGCCTCAGGGAGGTGGTTA

< S’alpha_1

< S’mu_1 S’mu_2 >

AAGACAGGACTGTGGAGACACCTTAGAAGGACAGATTCTGTTCCGAATCACCGATGCGGCGTCAGCAGGACTGGCCTAGCGGAGGCTCT

|||||| ||||||||||||||||||||||||||||||||||||||||||

AAGACAAGACTGTGGAGACACCTTAGAAGGACAGATTCTGTTCCGAATCCCTAATCTTACTAGAGACCCTAAAGAAAAGGGACCCCAGAGAGCAGCCCTG

|||||||||| ||||||||||||||||||||||||||||||||||||||||||

GTGTGACAGCCGTCATCCCA...70 nt...ATTCGGTCCCTAATCTCACTAGAGACCCTAAAGAAAAGGGACCCCAGAGAGCAGCCCTG

< S’alpha_1

< S’mu_1 S’mu_2 >

GACTGTGGAGACACCTTAGAAGGACAGATTCTGTTCCGAATCACCGATGCGGCGTCAGCAGGACTGGCCTAGCGGAGGCTCT

||||||||||||||||||||||||||| ||||||||||||||||||||||

GACTGTGGAGACACCTTAGAAGGACAGTCTCTGTTCCGAATCACCGATGCGTGCAGGGAGGTGGTTAGGGCCTGAGGGGGAACCCTCAAAAATGTGATTCGGTC

|||||||||||||||||||||||||||||||||||||||||||||||||||

GTGTGACAGCCGTCATCCCAGAGTGAGAGCATGCAGAGCTGGGGCCTCAGGGAGGTGGTTAGGGCCTGAGGGGGAACCCTCAAAAATGTGATTCGGTC

< S’alpha_1

< S’mu_1 S’mu_2 >

AGACAGGACTGTGGAGACACCTTAGAAGGACAGATTCTGTTCCGAATCACCGATGCGGCGTCAGCAGGACTGGCCTAGCGGAGGCTCT

||||||||||||||||||||||||||||||||||||||||||||||||||||||

AGACAGGACTGTGGAGACACCTTAGAAGGACAGATTCTGTTCCGAATCACCGATTCATCCCAGAGTGAGAGCATGCAGAGCTGGGGCCTCAGGGAGGTGGTTAGGGCC

||||||||||||||||||||||||||||||||||||||||||||||||||||||

GTGTGACAGCCGTCATCCCAGAGTGAGAGCATGCAGAGCTGGGGCCTCAGGGAGGTGGTTAGGGCC

< S’alpha_1

< S’mu_1 S’mu_2 >

ACTGTGGAGACACCTTAGAAGGACAGATTCTGTTCCGAATCACCGATGCGGCGTCAGCAGGACTGGCCTAGCGGAGGCTCT

||||||||||||||||||||||||||||||||||||||||||||||||||||| |||

ACTGTGGAGACACCTTAGAAGGACAGATTCTGTTCCGAATCACCGATGCGGCG-CAGAGCTGGGGCCTCAGGGAGGTGGTTAGGGCCTGAGGGGGAACCCTCAA

| ||||||||||||||||||||||||||||||||||||||||||||||||||

GTGTGACAGCCGTCATCCCAGAGTGAGAGCATG-CAGAGCTGGGGCCTCAGGGAGGTGGTTAGGGCCTGAGGGGGAACCCTCAA

< S’alpha_1

S’mu_2 >

GGCATAAGAGAAAACTCAATCAGATAGTGCTGAAGACAGGACTGTGGAGACACCTTA...44 nt...ACTGGCCTAGCGGAGGCTCT

|||||||||||||||||||||||||||||||||||||||||||||||||||

GGCATAAGAGAAAACTCAATCAGATAGTGCTGAAGACAGGACTGTGGAGACCAGAGAGCAGCCCTGCCCTCTTTCCACCATGTGGGGACGCAGTGAAAAGATGGGGG

|||||||||||||||||||||||||||||| ||| ||||||||||||||||||||||

GTGTGACAGCCGTCATCCCA...107 nt...GGGACCCCAGAGAGCAGCCCTGCCCTCTTTCCACCACGTGAGGACGCAGTGAAAAGATGGGGG

< S’alpha_1

< S’mu_1 S’mu_2 >

AGACAGGACTGTGGAGACACCTTAGAAGGACAGATTCTGTTCCGAATCACCGATGCGGCGTCAGCAGGACTGGCCTAGCGGAGGCTCT

||||||||||||||||||||||||||||||||||||||||||||||||

AGACAGGACTGTGGAGACACCTTAGAAGGACAGATTCTGTTCCGAATCGCTCAGGGAGGTGGTTAGGGCCTGAGGGGGAACCCTCAAAAATGTGATTCGGTCCCTA

|||||||||||||||||||||||||||||||||||||||||||||||||||||||||

GTGTGACAGCCGTCATCCCAGAGTGAGAGCATGCAGAGCTGGGGCCTCAGGGAGGTGGTTAGGGCCTGAGGGGGAACCCTCAAAAATGTGATTCGGTCCCTA

< S’alpha_1

< S’mu_1 S’mu_2 >

GATTCTGTTCCGAATCACCGATGCGGCGTCAGCAGGACTGGCCTAGCGGAGGCTCT

|||||||||||||||||||||||||||||||||

GATTCTGTTCCGAATCACCGATGCGGCGTCAGC[insert]GAGGTGGTTAGGGCCTGAGGGGGAAC

||||||||||||||||||||||||||

GTGTGACAGCCGTCATCCCA...25 nt...CTCAGGGAGGTGGTTAGGGCCTGAGGGGGAAC

< S’alpha_1

[insert]: TGGGGCCTCAGGGAGGTGGTTAGGGCCTGAGGGGGAACCCTCAAAAATGTGATTCGGTCCCTAATCTTACTAGAGACCCTAA

< S’mu_1 S’mu_2 >

TTAGAAGGACAGATTCTGTTCCGAATCACCGATGCGGCGTCAGCAGGACTGGCCTAGCGGAGGCTCT

|||||||||||||||||||||||||||||||||||||||||||||||||||||||

TTAGAAGGACAGATTCTGTTCCGAATCACCGATGCGGCGTCAGCAGGACTGGCCTCACAGCCGTCATCCCAGAGTGAGAGCACGCAGAGCTGGGGCCTC

|||||||||||||||||||||||||| ||||||||||||||||

GTGTGACAGCCGTCATCCCAGAGTGAGAGCATGCAGAGCTGGGGCCTC

< S’alpha_1

< S’mu_1 S’mu_2 >

TCAATCAGATAGTGCTGAAGACAGGACTGTGGAGACACCTTAGAAGGACAGATTCTGTTCCGAATCACCGATGCGGCGTCAGCAGGACTGGCCTAGCGGAGGCTCT

|||||||||||||||||||||||||||||||||||||||||||||||||||||||

TCAATCAGATAGTGCTGAAGACAGGACTGTGGAGACACCTTAGAAGGACAGATTCCAGAGAGCAGCCCTGCCCTCTTTCCACCATGTGAGGACGCAGTGAAAAGATGGGGG

|||||||||||||||||||||||||||||| ||||||||||||||||||||||||||

GTGTGACAGCCGTCATCCCA...107 nt...GGGACCCCAGAGAGCAGCCCTGCCCTCTTTCCACCACGTGAGGACGCAGTGAAAAGATGGGGG

< S’alpha_1

< S’mu_1 S’mu_2 >

GAAGGACAGATTCTGTTCCGAATCACCGATGCGGCGTCAGCAGGACTGGCCTAGCGGAGGCTCT

||||||||||||||||||||||||||||||||||||||||||||||||||||||||

GAAGGACAGATTCTGTTCCGAATCACCGATGCGGCGTCAGCAGGACTGGCCTAGCGGTGACAGCCGTCATCCCAGAGTGAGAGCATGCAGAGCTGGG

|||||||||||||||||||||||||||||||||||||||||

GTGTGACAGCCGTCATCCCAGAGTGAGAGCATGCAGAGCTGGG

< S’alpha_1

< S’mu_1 S’mu_2 >

AGGACAGATTCTGTTCCGAATCACCGATGCGGCGTCAGCAGGACTGGCCTAGCGGAGGCTCT

||||||||||||||||||||||||||||||||||||||||||||||

AGGACAGATTCTGTTCCGAATCACCGATGCGGCGTCAGCAGGACTGCCTAGCGGTGACAGCCGTCATCCCAGAGTGAGAGCATGCAGAGCT

||||||||||||||||||||||||||||||||||||||

GTGTGACAGCCGTCATCCCAGAGTGAGAGCATGCAGAGCT

< S’alpha_1

< S’mu_1 S’mu_2 >

GACTGTGGAGACACCTTAGAAGGACAGATTCTGTTCCGAATCACCGATGCGGCGTCAGCAGGACTGGCCTAGCGGAGGCTCT

|||||||||||||||||||||||||||||||||||||||||||||||||||

GACTGTGGAGACACCTTAGAAGGACAGATTCTGTTCCGAATCACCGATGCGTGCAGGGAGGTGGTTAGGGCCTGAGGGGGAACCCTCAAAAATGTGATTCGGTCCCTAATC

||||||||||||||||||||||||||||||||||||||||||||||||||||||||||

GTGTGACAGCCGTCATCCCAGAGTGAGAGCATGCAGAGCTGGGGCCTCAGGGAGGTGGTTAGGGCCTGAGGGGGAACCCTCAAAAATGTGATTCGGTCCCTAATC

< S’alpha_1

< S’mu_1 S’mu_2 >

GACTGTGGAGACACCTTAGAAGGACAGATTCTGTTCCGAATCACCGATGCGGCGTCAGCAGGACTGGCCTAGCGGAGGCTCT

|||||||||||||| ||||||||||||||||||||||||||||||||||||||| |||

GACTGTGGAGACACTTTAGAAGGACAGATTCTGTTCCGAATCACCGATGCGGCG-CAGAGCTGGGGCCTCAGGGAGGTGGTTAGGG-CTGAGGGGGAACCCTCAAAAAT

| ||||||||||||||||||||||||||||||| ||||||||||||||||||||||

GTGTGACAGCCGTCATCCCAGAGTGAGAGCATG-CAGAGCTGGGGCCTCAGGGAGGTGGTTAGGGCCTGAGGGGGAACCCTCAAAAAT

< S’alpha_1

< S’mu_1 S’mu_2 >

GTGCTGAAGACAGGACTGTGGAGACACCTTAGAAGGACAGATTCTGTTCCGAATCACCGATGCGGCGTCAGCAGGACTGGCCTAGCGGAGGCTCT

|||||||||||||||||||||||||||||||||||||||||||||||||||||

GTGCTGAAGACAGGACTGTGGAGACACCTTAGAAGGACAGATTCTGTTCCGAACCTCAGGGAGGTGGTTAGGGCCTGAGGGGGAACCCTCAAAAATGTGATTCGGTCCCTAAT

||||||||||||||||||||||||||||||||||||||||||||||||||||||||||||

GTGTGACAGCCGTCATCCCAGAGTGAGAGCATGCAGAGCTGGGGCCTCAGGGAGGTGGTTAGGGCCTGAGGGGGAACCCTCAAAAATGTGATTCGGTCCCTAAT

< S’alpha_1

< S’mu_1 S’mu_2 >

GATAGTGCTGAAGACAGGACTGTGGAGACACCTTAGAAGGACAGATTCTGTTCCGAATCACCGATGCGGCGTCAGCAGGACTGGCCTAGCGGAGGCTCT

|||||||||||||||||||||||||||||||||||||||||||||||||||||||||||

GATAGTGCTGAAGACAGGACTGTGGAGACACCTTAGAAGGACAGATTCTGTTCCGAATCGCTCAGGGAGGTGGTTAGGGCCTGAGGGGGAACCCTCAAAAATGTGATTCG

||||||||||||||||||||||||||||||||||||||||||||||||||

GTGTGACAGCCGTCATCCCAGAGTGAGAGCATGCAGAGCTGGGGCCTCAGGGAGGTGGTTAGGGCCTGAGGGGGAACCCTCAAAAATGTGATTCG

< S’alpha_1

< S’mu_1 S’mu_2 >

TGTGGAGACACCTTAGAAGGACAGATTCTGTTCCGAATCACCGATGCGGCGTCAGCAGGACTGGCCTAGCGGAGGCTCT

||||||||||||||||||||||||||||||||||||||||||||||||||||||||

TGTGGAGACACCTTAGAAGGACAGATTCTGTTCCGAATCACCGATGCGGCGTCAGCTGGGGCCTCAGGGAGGTGGTTAGGGCCTGAGGGGGAACCCTCAAAAATGTGATTCGG

||||||||||||||||||||||||||||||||||||||||||||||||||||||||||||

GTGTGACAGCCGTCATCCCAGAGTGAGAGCATGCAGAGCTGGGGCCTCAGGGAGGTGGTTAGGGCCTGAGGGGGAACCCTCAAAAATGTGATTCGG

< S’alpha_1

< S’mu_1 S’mu_2 >

AAAACTCAATCAGATAGTGCTGAAGACAGGACTGTGGAGACACCTTAGAAGGACAGATTCTGTTCCGAATCACCGATGCGGCGTCAGCAGGACTGGCCTAGCGGAGGCTCT

|||||||||||||||||||||||||||| |||||||||||||||||| ||||||||||

AAAACTCAATCAGATAGTGCTGAAGACAAGACTGTGGAGACACCTTARAAGGACAGATGCAGCCCTGCCCTCTTTCCACCACGTGAGGACGCAGTGAAAAGATGGGGGT

||| |||||||||||||||||||||||||||||||||||||||||||||||||||

GTGTGACAGCCGTCATCCCA...111 nt...CCCCAGAGA-GCAGCCCTGCCCTCTTTCCACCACGTGAGGACGCAGTGAAAAGATGGGGGT

< S’alpha_1

< S’mu_1 S’mu_2 >

GATAGTGCTGAAGACAGGACTGTGGAGACACCTTAGAAGGACAGATTCTGTTCCGAATCACCGATGCGGCGTCAGCAGGACTGGCCTAGCGGAGGCTCT

|||||||||||||||| ||||||||||||||||||||||||||||||||||||||||||

GATAGTGCTGAAGACAAGACTGTGGAGACACCTTAGAAGGACAGATTCTGTTCCGAATCCCAGAGTGAGAGCATGCAGAGCTGGGGCCTCAGGGAGGTGGTTAGGGCCTGAGG

|||||||||||||||||||||||||||||||||||||||||||||||||||||||||

GTGTGACAGCCGTCATCCCAGAGTGAGAGCATGCAGAGCTGGGGCCTCAGGGAGGTGGTTAGGGCCTGAGG

< S’alpha_1

< S’mu_1 S’mu_2 >

CAGATTCTGTTCCGAATCACCGATGCGGCGTCAGCAGGACTGGCCTAGCGGAGGCTCT

||||||||||||||||||||||||||||||||||||||||||||||

CAGATTCTGTTCCGAATCACCGATGCGGCGTCAGCAGGACTGGCCTCCCCAGAGTGAGAGCATGCAGAGCTGGGGCCTCAGGGAGGTGGTTAGGGCCTGAGGG

||||||||||||||||||||||||||||||||||||||||||||||||||||||||

GTGTGACAGCCGTCATCCCAGAGTGAGAGCATGCAGAGCTGGGGCCTCAGGGAGGTGGTTAGGGCCTGAGGG

< S’alpha_1

< S’mu_1 S’mu_2 >

TCTGTTCCGAATCACCGATGCGGCGTCAGCAGGACTGGCCTAGCGGAGGCTCT

|||||||||||||||||||||||||||||||||

TCTGTTCCGAATCACCGATGCGGCGTCAGCAGG[insert]AGAAAAGGGACCCCAGAGAGCAGCCCTGCCCTCTTTCCACCATGTGAGGACGCAGT

|||||||||||||||||||||||||||||||||||||||||| |||||||||||||

GTGTGACAGCCGTCATCCCA...95 nt...CCCTAAAGAAAAGGGACCCCAGAGAGCAGCCCTGCCCTCTTTCCACCACGTGAGGACGCAGT

< S’alpha_1

[insert]: GAGATTAGGGGCCGAATCACATTTTTGAGGGTTCCCCCTCAGGCCCTAACCACCTCCCTGAGGCCCCAGCTCTGCATGCTCTCACTCTGATCCC

S’mu_2 >

CATGTGATAATTAATCTCAAATACTTTTTCGATACCTCAGAGCATTATTTTCATAATGACTGT...288 nt...ACTGGCCTAGCGGAGGCTCT

|||||||||||||||||||||||||||||||||||||||||||||||||||||||||

CATGTGATAATTAATCTCAAATACTTTTTCGATACCTCAGAGCATTATTTTCATAATAGGGCCTGAGGGGGAACCCTCAAAAATGTGATTCGGTCCCTAATCTCACTAG

|||||||||||||||||||||||||||||||||||||||||||||||||||||

GTGTGACAGCCGTCATCCCA...33 nt...GGTGGTTAGGGCCTGAGGGGGAACCCTCAAAAATGTGATTCGGTCCCTAATCTCACTAG

< S’alpha_1

< S’mu_1 S’mu_2 >

AAACTCAATCAGATAGTGCTGAAGACAGGACTGTGGAGACACCTTAGAAGGACAGATTCTGTTCCGAATCACCGATGCGGCGTCAGCAGGACTGGCCTAGCGGAGGCTCT

||||||||||||||||||||| ||||||||||||||||||||||||||||

AAACTCAATCAGATAGTGCTGGAGACAGGACTGTGGAGACACCTTAGAAGCATGCAGAGCTGGGGCCTCAGGGAGGTGGTTAGGGCCTGAGGGGGAACCCTCAA

||||||||||||||||||||||||||||||||||||||||||||||||||||||||

GTGTGACAGCCGTCATCCCAGAGTGAGAGCATGCAGAGCTGGGGCCTCAGGGAGGTGGTTAGGGCCTGAGGGGGAACCCTCAA

< S’alpha_1

< S’mu_1 S’mu_2 >

AATCAGATAGTGCTGAAGACAGGACTGTGGAGACACCTTAGAAGGACAGATTC-TGTTCCGAATCACCGATGCGGCGTCAGCAGGACTGGCCTAGCGGAGGCTCT

||||||||||||||||||||| ||||||||||||||||||||||||||||||| |

AATCAGATAGTGCTGAAGACAAGACTGTGGAGACACCTTAGAAGGACAGATTC-TAACAAAAGCTCCAGAGCTTCCCTGAACCGCCAGGTGTGTCTTCCCAGGA

|| ||| ||||||||||||||||||||||||||||||||||||||||||||||||||

GTGTGACAGCCGTCATCCCA...357 nt...ATCCCAAGGTTCATAACAAAAGCTCCAGAGCTTCCCTGAACCGCCAGGTGTGTCTTCCCAGGA

< S’alpha_1

< S’mu_1 S’mu_2 >

GTGGAGACACCTTAGAAGGACAGATTCTGTTCCGAATCACCGATGCGGCGTCAGCAGGACTGGCCTAGCGGAGGCTCT

||||||||||||||||||||||||||||||||||||||||||||||||||||||

GTGGAGACACCTTAGAAGGACAGATTCTGTTCCGAATCACCGATGCGGCGTCAGAGCAGCCCTGCCCTCTTTCCACCATGTGAGGACGCAGTGAAAAGATGGGGGTCTGT

|||||||||||||||||||||||||| |||||||||||||||||||||||||||||||

GTGTGACAGCCGTCATCCCA...111 nt...CCCCAGAGAGCAGCCCTGCCCTCTTTCCACCACGTGAGGACGCAGTGAAAAGATGGGGGTCTGT

< S’alpha_1

< S’mu_1 S’mu_2 >

AGACAGGACTGTGGAGACACCTTAGAAGGACAGATTCTGTTCCGAATCACCGATGCGGCGTCAGCAGGACTGGCCTAGCGGAGGCTCT

|||||||||||||||||||||||||||||||||||||||||||||||||||||

AGACAGGACTGTGGAGACACCTTAGAAGGACAGATTCTGTTCCGAATCACCGACACTAGAGACCCTAAAGAAAAGGGACCCCAGAGAGCAGCCCTGCCCTCTTTCCACCA

|||||||||||||||||||||||||||||||||||||||||||||||||||||||||

GTGTGACAGCCGTCATCCCA...80 nt...TAATCTCACTAGAGACCCTAAAGAAAAGGGACCCCAGAGAGCAGCCCTGCCCTCTTTCCACCA

< S’alpha_1

< S’mu_1 S’mu_2 >

GGACAGATTCTGTTCCGAATCACCGATGCGGCGTCAGCAGGACTGGCCTAGCGGAGGCTCT

|||||||||||||||||||||||||||||||||||||||||

GGACAGATTCTGTTCCGAATCACCGATGCGGCGTCAGCAGG[insert]AGAAAAGGGACCCCAGAGAGCAGCCCTGCCCTCTTTCCACCA

||||||||||||||||||||||||||||||||||||||||||

GTGTGACAGCCGTCATCCCA...95 nt...CCCTAAAGAAAAGGGACCCCAGAGAGCAGCCCTGCCCTCTTTCCACCA

< S’alpha_1

[insert]: GAGATTAGGGACCGAATCACATTTTTGAGGGTTCCCCCTCAGGCCCTAACCACCTCCCTGAGGCCCCAGCTCTGCATGCTCTCACTCTGATCCC

< S’mu_1 S’mu_2 >

AGACAGGACTGTGGAGACACCTTAGAAGGACAGATTCTGTTCCGAATCACCGATGCGGCGTCAGCAGGACTGGCCTAGCGGAGGCTCT

|||||||||||||||||||||||||||||||||||||||||||||

AGACAGGACTGTGGAGACACCTTAGAAGGACAGATTCTGTTCCGATTTAGAGTTGGGGCCTCAGGGAGGTGGTTAGGGCCTGAGGGGGAACCCTCAAAAATGTG

|||| |||||||||||||||||||||||||||||||||||||||||||||||||||

GTGTGACAGCCGTCATCCCAGAGTGAGAGCATGCAGAGCTGGGGCCTCAGGGAGGTGGTTAGGGCCTGAGGGGGAACCCTCAAAAATGTG

< S’alpha_1

**BJAB, S’μ-S’α junctions from 3’DSBs, short overhangs (Cas9 N863A, S’μ_1 + S’μ_2 + S’α_1 + S’α_2)**

< S’mu_1 S’mu_2 >

GACTGTGGAGACACCTTAGAAGGACAGATTCTGTTCCGAATCACCGATGCGGCGTCAGCAGGACTGGCCTAGCGGAGGCTCT

||||||||||||||||||||||||||||||||||||||||||||||||||||

GACTGTGGAGACACCTTAGAAGGACAGATTCTGTTCCGAATCACCGATGCGGGAGTGAGAGCATGCAGAGCTGGGGCCTCGGGGAGGTGGTTAGGGCCTGAGGGGGAAC

|||||||||||||||||||||||||||| ||||||||||||||||||||||||||||

GTGTGACAGCCGTCATCCCAGAGTGAGAGCATGCAGAGCTGGGGCCTCAGGGAGGTGGTTAGGGCCTGAGGGGGAAC

< S’alpha_1 S’alpha_2 >

< S’mu_1 S’mu_2 >

ATTCTGTTCCGAATCACCGATGCGGCGTCAGCAGGACTGGCCTAGCGGAGGCTCT

|||||||||||||||||||||||||||||||||||||||||

ATTCTGTTCCGAATCACCGATGCGGCGTCAGCAGGACTGGCTCAAAACCTATGCGTCATCCCAGAGTGAGAGCACGCAGAGCTGGGGCCTC

||||||||||||||||||||| ||||||||||||||||

GTGTGACAGCCGTCATCCCAGAGTGAGAGCATGCAGAGCTGGGGCCTC

< S’alpha_1

< S’mu_1 S’mu_2 >

AAGGACAGATTCTGTTCCGAATCACCGATGCGGCGTCAGCAGGACTGGCCTAGCGGAGGCTCT

||||||||||||||||||||||||||||||||||||||||||||

AAGGACAGATTCTGTTCCGAATCACCGATGCGGCGTCAGCAGGAGCCGTCATCCCAGAGTGAGAGCATGCAGAGCTGGGGCCTCA

||||||||||||||||||||||||||||||||||||||||||

GTGTGACAGCCGTCATCCCAGAGTGAGAGCATGCAGAGCTGGGGCCTCA

< S’alpha_1

< S’mu_1 S’mu_2 >

ATTCTGTTCCGAATCACCGATGCGGCGTCAGCAGGACTGGCCTAGCGGAGGCTCT

|||||||||||||||||||||||||||||||||||||||||

ATTCTGTTCCGAATCACCGATGCGGCGTCAGCAGGACTGGCTCAAAGCCTATGCGTCATCCCATAGTGAGAGCATGCAGAGCTGGGGCCT

|||||||||| ||||||||||||||||||||||||||

GTGTGACAGCCGTCATCCCAGAGTGAGAGCATGCAGAGCTGGGGCCT

< S’alpha_1

< S’mu_1 S’mu_2 >

CGATGCGGCGTCAGCAGGACTGGCCTAGCGGAGGCTCT

||||||||||||||||||||||||

CGATGCGGCGTCAGCAGGACTGGCTCAAAACATATGCGTCATCCCAGAGTGAGAGCATGCAGAGCTGGGGCCTC

||||||||||||||||||||||||||||||||||||||

GTGTGACAGCCGTCATCCCAGAGTGAGAGCATGCAGAGCTGGGGCCTC

< S’alpha_1

< S’mu_1 S’mu_2 >

TTCTGTTCCGAATCACCGATGCGGCGTCAGCAGGACTGGCCTAGCGGAGGCTCT

|||||||||||||||||||||||||||||||||||||||||||||||||||

TTCTGTTCCGAATCACCGATGCGGCGTCAGCAGGACTGGCCTAGCGGAGGCGGGGACAGCCGTCATCCCAGAGTGAGAGCATGCAGAGCTGGGGCCTCAGGGAG

||||||||||||||||||||||||||||||||||||||||||||||||||

GTGTGACAGCCGTCATCCCAGAGTGAGAGCATGCAGAGCTGGGGCCTCAGGGAG

< S’alpha_1

S’mu_2 >

ATGCGGCGTCAGCAGGACTGGCCTAGCGGAGGCTCT

||||||||||||||||||||||||||||||||||

ATGCGGCGTCAGCAGGACTGGCCTAGCGGAGGCTGGGGCCTCGGGGAGGTGCAGCCGTCATCCCAGGGTGAGAGCATGCAGAGCTGGGGCCTCGGGGAGGTGGTTAGGGCCTGA

||||||||||||||| |||||||||||||||||||||||||| ||||||||||||||||||||

GTGTGACAGCCGTCATCCCAGAGTGAGAGCATGCAGAGCTGGGGCCTCAGGGAGGTGGTTAGGGCCTGA

< S’alpha_1 S’alpha_2 >

< S’mu_1 S’mu_2 >

CTTAGAAGGACAGATTCTGTTCCGAATCACCGATGCGGCGTCAGCAGGACTGGCCTAGCGGAGGCTCT

||||||||||||||||||||||||||||||||||||||||||||||||||

CTTAGAAGGACAGATTCTGTTCCGAATCACCGATGCGGCGTCAGCAGGACAGAGTGAGAGCATGCTGAGCTGGGGCCTCGGGGAGGTGGTTAGGGCCTGAG

|||||||||||||||| ||||||||||||| |||||||||||||||||||||

GTGTGACAGCCGTCATCCCAGAGTGAGAGCATGCAGAGCTGGGGCCTCAGGGAGGTGGTTAGGGCCTGAG

< S’alpha_1 S’alpha_2 >

< S’mu_1 S’mu_2 >

CTTAGAAGGACAGATTCTGTTCCGAATCACCGATGCGGCGTCAGCAGGACTGGCCTAGCGGAGGCTCT

||||||||||||||||||||||||||||||||||||||||||||||||||

CTTAGAAGGACAGATTCTGTTCCGAATCACCGATGCGGCGTCAGCAGGACCGTCATCCCAGGGTGAGAGCATGCAGAGCTGGGGCCTCGGGGAGGTGGTTAGGG

|||||||||||| |||||||||||||||||||||||||| |||||||||||||||

GTGTGACAGCCGTCATCCCAGAGTGAGAGCATGCAGAGCTGGGGCCTCAGGGAGGTGGTTAGGG

< S’alpha_1 S’alpha_2 >

< S’mu_1 S’mu_2 >

TAGAAGGACAGATTCTGTTCCGAATCACCGATGCGGCGTCAGCAGGACTGGCCTAGCGGAGGCTCT

||||||||||||||||||||||||||||||||||||||||||||||||||||||||

TAGAAGGACAGATTCTGTTCCGAATCACCGATGCGGCGTCAGCAGGACTGGCCTAGTAGAGTGAGAGCATGCAGAGCTGGGGCCTCAGGGAGGTGGTTAGGGCCTGAGG

||||||||||||||||||||||||||||||||||||||||||||||||||||

GTGTGACAGCCGTCATCCCAGAGTGAGAGCATGCAGAGCTGGGGCCTCAGGGAGGTGGTTAGGGCCTGAGG

< S’alpha_1 S’alpha_2 >

S’mu_2 >

CAGCAGGACTGGCCTAGCGGAGGCTCT

||||||||||||||||||||||||

CAGCAGGACTGGCCTAGCGGAGGCCGTCATCCCAGAGTGAGAGCATGCAGAGCTGGGGCCTCAGGGAGGTGGAGAGCATGCAGAGCTGGGGCCTCAGGGAGGTGGTTAGGGC

|||||||||||||||||||||||||||||||||||||||||

GTGTGACAGCCGTCATCCCAGAGTGAGAGCATGCAGAGCTGGGGCCTCAGGGAGGTGGTTAGGGC

< S’alpha_1 S’alpha_2 >

S’mu_2 >

TGGCATAAGAGAAAACTCAATCAGATAGTGCTGAAGACAGGACTGTGGAGACA...49 nt...ACTGGCCTAGCGGAGGCTCT

||||||||||| ||||||||||||||||||||||||||| |||||||

TGGCATAAGAGGAAACTCAATCAGATAGTGCTGAAGACAAGACTGTGATGTGACTCGGTCCCTGATCTCACTAGAGACCCAAAAGAAAAGGGACCCCAGAGAGC

|||||| ||||||||| |||||||||||||||| |||||||||||||||||||||||

GTGTGACAGCCGTCATCCCA...59 nt...TCAAAAATGTGATTCGGTCCCTAATCTCACTAGAGACCCTAAAGAAAAGGGACCCCAGAGAGC

< S’alpha_1

< S’mu_1 S’mu_2 >

GAAGGACAGATTCTGTTCCGAATCACCGATGCGGCGTCAGCAGGACTGGCCTAGCGGAGGCTCT

||||||||||||||||||||||||||||||||||||||||||||||||||||||

GAAGGACAGATTCTGTTCCGAATCACCGATGCGGCGTCAGCAGGACTGGCCTAGCAGAGTGAGAGCATGCAGAGCTGGGGCCTCAGGGAGGTGGTTAGGGCCTGAGGGG

|||||||||||||||||||||||||||||||||||||||||||||||||||||||

GTGTGACAGCCGTCATCCCAGAGTGAGAGCATGCAGAGCTGGGGCCTCAGGGAGGTGGTTAGGGCCTGAGGGG

< S’alpha_1 S’alpha_2 >

< S’mu_1 S’mu_2 >

TCTGTTCCGAATCACCGATGCGGCGTCAGCAGGACTGGCCTAGCGGAGGCTCT

|||||||||||||||||||||||| ||||||||||||||||||||

TCTGTTCCGAATCACCGATGCGGCATCAGCAGGACTGGCCTAGCGGACAGCCGTCATCCCAGAGTGAGAGCATGCAGAGCTGGGGCCTCGGGGAGGTGGTTAGGGCCTGA

|||||||||||||||||||||||||||||||||||||||||||| ||||||||||||||||||||

GTGTGACAGCCGTCATCCCAGAGTGAGAGCATGCAGAGCTGGGGCCTCAGGGAGGTGGTTAGGGCCTGA

< S’alpha_1 S’alpha_2 >

< S’mu_1 S’mu_2 >

CAGATTCTGTTCCGAATCACCGATGCGGCGTCAGCAGGACTGGCCTAGCGGAGGCTCT

|||||||||||||||||||||||||||||||||||||||||||||||||||||

CAGATTCTGTTCCGAATCACCGATGCGGCGTCAGCAGGACTGGCCTAGCGGAGCTGGGGCCTCGGGGAGATGGTTAGGGCCTGAGGGGGAACACTCAAAAAT

|| ||||||||||||| ||||| |||||||||||||||||||||| |||||||||

GTGTGACAGCCGTCATCCCAGAGTGAGAGCATGCAGAGCTGGGGCCTCAGGGAGGTGGTTAGGGCCTGAGGGGGAACCCTCAAAAAT

< S’alpha_1 S’alpha_2 >

< S’mu_1 S’mu_2 >

TTAGAAGGACAGATTCTGTTCCGAATCACCGATGCGGCGTCAGCAGGACTGGCCTAGCGGAGGCTCT

|||||||||||||||||||||||||||||||||||||||||||||||||||||||||

TTAGAAGGACAGATTCTGTTCCGAATCACCGATGCGGCGTCAGCAGGACTGGCCTAGAGTGAGAGCATGCAGAGCTGGGGCCTCGGGGAGGTGGTTAGGGCCTGAGGGG

|| ||||||||||||||||||||||||||||| ||||||||||||||||||||||||

GTGTGACAGCCGTCATCCCAGAGTGAGAGCATGCAGAGCTGGGGCCTCAGGGAGGTGGTTAGGGCCTGAGGGG

< S’alpha_1 S’alpha_2 >

< S’mu_1 S’mu_2 >

GGACAGATTCTGTTCCGAATCACCGATGCGGCGTCAGCAGGACTGGCCTAGCGGAGGCTCT

||||||||||||||||||||||||||||||||||||||||||||||||||||||||

GGACAGATTCTGTTCCGAATCACCGATGCGGCGTCAGCAGGACTGGCCTAGCGGAGGCCGTCATCCCAGAGTGAGAGCATGCAGAGCTGGGGCCTCGGGGAGGTGGTTAGGG

|||||||||||||||||||||||||||||||||||||||| |||||||||||||||

GTGTGACAGCCGTCATCCCAGAGTGAGAGCATGCAGAGCTGGGGCCTCAGGGAGGTGGTTAGGG

< S’alpha_1 S’alpha_2 >

< S’mu_1 S’mu_2 >

TTAGAAGGACAGATTCTGTTCCGAATCACCGATGCGGCGTCAGCAGGACTGGCCTAGCGGAGGCTCT

|||||||||||||||||||||||||||| |||||||||||||||||||||||| ||||

TTAGAAGGACAGATTCTGTTCCGAATCA-CGATGCGGCGTCAGCAGGACTGGC-TAGCTGACAGCCGTCATCCCAGAGTGAGAGCATGCAGAGCTGGGGCCTC

|||||||||||||||||||||||||||||||||||||||||||||

GTGTGACAGCCGTCATCCCAGAGTGAGAGCATGCAGAGCTGGGGCCTC

< S’alpha_1

S’mu_2 >

TCAGCAGGACTGGCCTAGCGGAGGCTCT

|||||||||||||||

TCAGCAGGACTGGCCACAGCCGTCATCCCAGAGTGAGGGCATGCAGAGCTGGGGCCTCAGGGAGGTGGTCAGCAGGACTGGCCACAGCCGTCATCCCAGAGTG

||||||||||||||||||||

GTGTGACAGCCGTCATCCCAGAGTG

< S’alpha_1

S’mu_2 >

GCGGCGTCAGCAGGACTGGCCTAGCGGAGGCTCT

|||||||||||||||||||

GCGGCGTCAGCAGGACTGGGTCCCAGAGTGAGAGCATGCAGAGCTGGGGCCTCAGGGAGGTGGTTAGGCGTCAGCAGGACTGGGTCCCAGAGTGAGAGCATGCAGAG

|||||||||||||||||||||||

GTGTGACAGCCGTCATCCCAGAGTGAGAGCATGCAGAG

< S’alpha_1

< S’mu_1 S’mu_2 >

CCTTAGAAGGACAGATTCTGTTCCGAATCACCGATGCGGCGTCAGCAGGACTGGCCTAGCGGAGGCTCT

||||||||||| ||||||||||||||||||||||||||||||||||||||||||

CCTTAGAAGGATAGATTCTGTTCCGAATCACCGATGCGGCGTCAGCAGGACTGGGAGAGCATGCAGAGCTAGGGGCCCCAGGGAGGTGGTTAGGGCCTGAGGGGGAAC

|||||||||||||||| |||||| ||||||||||||||||||||||||||||||

GTGTGACAGCCGTCATCCCAGAGTGAGAGCATGCAGAGCT-GGGGCCTCAGGGAGGTGGTTAGGGCCTGAGGGGGAAC

< S’alpha_1 S’alpha_2 >

< S’mu_1 S’mu_2 >

TTCTGTTCCGAATCACCGATGCGGCGTCAGCAGGACTGGCCTAGCGGAGGCTCT

||||||||||||||||||||||||||||||||||

TTCTGTTCCGAATCACCGATGCGGCGTCAGCAGGCGTCAGCAGGACAGGACTGGCCTGCAGACATAGCTGGCCTCCCCCGACTCCAGGGCCAGCATCG

||||||||||||||||||||||||||||||||||||||||

GTGTGACAGCCGTCATCCCA ...635 nt...AGCCCCCAGACATAGCTGGCCTCCCCCGACTCCAGGGCCAGCATCG

< S’alpha_1

S’mu_2 >

TCAGCAGGACTGGCCTAGCGGAGGCTCT

|||||||||||||

TCAGCAGGACTGGGTGACAGCCGTCATCCCAGAGTGAGAGCATGCAGAGCTGGGGCCTCGGTGACAGCCGTCATCCCAGAGTGAGAGC

||||||||||||||||||||||||||||

GTGTGACAGCCGTCATCCCAGAGTGAGAGC

< S’alpha_1

< S’mu_1 S’mu_2 >

GAAGGACAGATTCTGTTCCGAATCACCGATGCGGCGTCAGCAGGACTGGCCTAGCGGAGGCTCT

||||||||||||||||||||||||||||||||||||||||||||||||||||||

GAAGGACAGATTCTGTTCCGAATCACCGATGCGGCGTCAGCAGGACTGGCCTAGTGAGAGCATGCAGAGCTGGGGCCTCAGGGAGGTGGTTAGGGCCTGAGGGGGAACC

|||||||||||||||||||||||||||||||||||||||||||||||||||||||||

GTGTGACAGCCGTCATCCCAGAGTGAGAGCATGCAGAGCTGGGGCCTCAGGGAGGTGGTTAGGGCCTGAGGGGGAACC

< S’alpha_1 S’alpha_2 >

< S’mu_1 S’mu_2 >

CTTAGAAGGACAGATTCTGTTCCGAATCACCGATGCGGCGTCAGCAGGACTGGCCTAGCGGAGGCTCT

|||||||||||||||||||||||||||||||||||||||||||||||||||||||

CTTAGAAGGACAGATTCTGTTCCGAATCACCGATGCGGCGTCAGCAGGACTGGCCAAAATGTGACTCGGTCCCTGATCTCACTAGAGACCCAAAAGAAAAGGGACCCCAG

||||||||| ||||||||| |||||||||||||||| ||||||||||||||||||

GTGTGACAGCCGTCATCCCA...56 nt...CCCTCAAAAATGTGATTCGGTCCCTAATCTCACTAGAGACCCTAAAGAAAAGGGACCCCAG

< S’alpha_1

< S’mu_1 S’mu_2 >

GTGGAGACACCTTAGAAGGACAGATTCTGTTCCGAATCACCGATGCGGCGTCAGCAGGACTGGCCTAGCGGAGGCTCT

|||||||||||||||||||||||||||||||||||||||||||||||||||||||||

GTGGAGACACCTTAGAAGGACAGATTCTGTTCCGAATCACCGATGCGGCGTCAGCAGAGTGAGAGCATGCAGAGCTGGGGCCCCGGGGAGGTGGTTAGGGCCTGAG

|||||||||||||||||||||||||||| | |||||||||||||||||||||

GTGTGACAGCCGTCATCCCAGAGTGAGAGCATGCAGAGCTGGGGCCTCAGGGAGGTGGTTAGGGCCTGAG

< S’alpha_1 S’alpha_2 >

< S’mu_1 S’mu_2 >

AGGACAGATTCTGTTCCGAATCACCGATGCGGCGTCAGCAGGACTGGCCTAGCGGAGGCTCT

||||||||||||||||||||||||||||||||||||||||||||||||||||||||

AGGACAGATTCTGTTCCGAATCACCGATGCGGCGTCAGCAGGACTGGCCTAGCGGAGACAGCCGTCATCCCAGAGTGAGAGCATGCAGAGCTGGGGCCTCAGGGAGGT

||||||||||||||||||||||||||||||||||||||||||||||||||||

GTGTGACAGCCGTCATCCCAGAGTGAGAGCATGCAGAGCTGGGGCCTCAGGGAGGT

< S’alpha_1

S’mu_2 >

CTCAAATACTTTTTCGATACCTCAGAGCATTATTTTCATAATGACTGTGTTCACAATCTTT...275 nt...ACTGGCCTAGCGGAGGCTCT

|||||||||||||||||||||||||||||||||||||||||||||||||||||||

CTCAAATACTTTTTCGATACCTCAGAGCATTATTTTCATAATGACTGTGTTCACACGGTTAGGGCCTGAGGGGGAACACTCAAAAATGTGACTCGGTCCCTGATCTCACT

||||||||||||||||||||| ||||||||||||| ||||||||| ||||||||

GTGTGACAGCCGTCATCCCAGAGTGAGAGCATGCAGAGCTGGGGCCTCAGGGAGGTGGTTAGGGCCTGAGGGGGAACCCTCAAAAATGTGATTCGGTCCCTAATCTCACT

< S’alpha_1 S’alpha_2 >

< S’mu_1 S’mu_2 >

AGACAGGACTGTGGAGACACCTTAGAAGGACAGATTCTGTTCCGAATCACCGATGCGGCGTCAGCAGGACTGGCCTAGCGGAGGCTCT

||||||||||||||||||||||||||||||||||||||||||||||

AGACAGGACTGTGGAGACACCTTAGAAGGACAGATTCTGTTCCGAAAGGAAAAGGGACCCCAGAGAGCAGCCCTGCCCTCTTTCCACCATGTGAGGAC

|| ||| ||||||||||||||||||||||||||||||||||||||||| ||||||||

GTGTGACAGCCGTCATCCCA...90 nt...AGAGACCCTAAA-GAAAAGGGACCCCAGAGAGCAGCCCTGCCCTCTTTCCACCACGTGAGGAC

< S’alpha_1

< S’mu_1 S’mu_2 >

AATCACCGATGCGGCGTCAGCAGGACTGGCCTAGCGGAGGCTCT

|||||||||||||||||||||

AATCACCGATGCGGCGTCAGCCGTCATCCCAGAGTGAGAGCATGCAGAGCTGGGGCCTCAGGGAGGCGTCAGCCGTCATCCCAGAGTGAGAGCATGCAGAGCTGGGGC

|||||||||||||||||||||||||||||||||||||||

GTGTGACAGCCGTCATCCCAGAGTGAGAGCATGCAGAGCTGGGGC

< S’alpha_1

< S’mu_1 S’mu_2 >

GTGGAGACACCTTAGAAGGACAGATTCTGTTCCGAATCACCGATGCGGCGTCAGCAGGACTGGCCTAGCGGAGGCTCT

||||||||||||||||||||||||||||||||||||||||||||||||||||||||||||

GTGGAGACACCTTAGAAGGACAGATTCTGTTCCGAATCACCGATGCGGCGTCAGCAGGACACTCCCTGGATTTCACCCGACAGCCTCCGCCGGGGTGGCCCGGCCCCATGGCC

||||||||||||||||||||||||||||||||||||||||||||||||||||||

GTGTGACAGCCGTCATCCCA...481 nt...CACTGTCACTCCCTGGATTTCACCCGACAGCCTCCGCCGGGGTGGCCCGGCCCCATGGCC

< S’alpha_1

< S’mu_1 S’mu_2 >

GACACCTTAGAAGGACAGATTCTGTTCCGAATCACCGATGCGGCGTCAGCAGGACTGGCCTAGCGGAGGCTCT

||||||||||||||||||||||||||||||||||||||||||||||||||||

GACACCTTAGAAGGACAGATTCTGTTCCGAATCACCGATGCGGCGTCAGCAGCCCAGAGTGAGAGCATGCAGAGCTGGGGCCTCAGGGAGGTGGTTAGGGCCTGAG

|| ||||||||||||||||||||||||||||||||||||||||||||||||||||||

GTGTGACAGCCGTCATCCCAGAGTGAGAGCATGCAGAGCTGGGGCCTCAGGGAGGTGGTTAGGGCCTGAG

< S’alpha_1 S’alpha_2 >

< S’mu_1 S’mu_2 >

TTAGAAGGACAGATTCTGTTCCGAATCACCGATGCGGCGTCAGCAGGACTGGCCTAGCGGAGGCTCT

||||||||||||||||||||||||||||||||||||||||||||||||||||

TTAGAAGGACAGATTCTGTTCCGAATCACCGATGCGGCGTCAGCAGGACTGGCAGCCGTCATCCCAGAGTGAGAGCATGCAGAGCTGGGGCCTCGGGGAGGTGG

|||||||||||||||||||||||||||||||||||||||||| |||||||||

GTGTGACAGCCGTCATCCCAGAGTGAGAGCATGCAGAGCTGGGGCCTCAGGGAGGTGG

< S’alpha_1

**BJAB, S’μ-S’α junctions from 3’DSBs, short overhangs (Cas9 N863A, S’μ_1 + S’μ_2 + S’α_I + S’α_II)**

< S’mu_1 S’mu_2 >

GATTCTGTTCCGAATCACCGATGCGGCGTCAGCAGGACTGGCCTAGCGGAGGCTCT

||||||||||||||||||||||||||||||||||

GATTCTGTTCCGAATCACCGATGCGGCGTCAGCA[insert]ACGCAGTGAAAAGATGGGGGTCTGTGAACCAGGA

||||||||||||||||||||||||||||||||||

TCTTTCCACCACGTGAGGACGCAGTGAAAAGATGGGGGTCTGTGAACCAGGA

< S’alpha_I

[insert]: CCCAGAAGGGACAGAAGAACAGCCGCGAGAGAATGAAGCGGATCGAAGAGGGCATCAAAGAGCTGGGC

< S’mu_1 S’mu_2 >

CAGATTCTGTTCCGAATCACCGATGCGGCGTCAGCAGGACTGGCCTAGCGGAGGCTCT

||||||||||||||||||||||||||||||||||||||||||||||||

CAGATTCTGTTCCGAATCACCGATGCGGCGTCAGCAGGACTGGCCTAGCGTGAGGACGCAGTGAAAAGATGGGGGTCTGTGAACCAGGAATCGACCCTT

|||||||||||||||||||||||||||||||||||||||||||||||||||

TCTTTCCACCACGTGAGGACGCAGTGAAAAGATGGGGGTCTGTGAACCAGGAATCGACCCTT

< S’alpha_I

< S’mu_1 S’mu_2 >

AGATTCTGTTCCGAATCACCGATGCGGCGTCAGCAGGACTGGCCTAGCGGAGGCTCT

||||||||||||||||||||||||||||||||||||||||||||||||||||||

AGATTCTGTTCCGAATCACCGATGCGGCGTCAGCAGGACTGGCCTAGCGGAGGCTGCCATCCTGACCTTGAACTTCCAGCTTCGAGAACCGTGAGAAATGA

||||||||||||||||||||||||||||||||||||||||||||||||

TCTTTCCACCACGTGAGGAC...53 nt...ACCCTGCTGCCATCCTGACCTTGAACTTCCAGCTTCGAGAACCGTGAGAAATGA

< S’alpha_I

< S’mu_1 S’mu_2 >

GGAGACACCTTAGAAGGACAGATTCTGTTCCGAATCACCGATGCGGCGTCAGCAGGACTGGCCTAGCGGAGGCTCT

||||||||||||||||||||||||||||||||||||||||||||||||

GGAGACACCTTAGAAGGACAGATTCTGTTCCGAATCACCGATGCGGCGATGGGGGTCTGTGAACCAGGAATCGACCCTTCCTGGAACTTGACCCTGCTGCCA

|||||||||||||||||||||||||||||||||||||||||||||||||||||||

TCTTTCCACCACGTGAGGACGCAGTGAAAAGATGGGGGTCTGTGAACCAGGAATCGACCCTTCCTGGAACTTGACCCTGCTGCCA

< S’alpha_I S’alpha_II >

< S’mu_1 S’mu_2 >

CAGATTCTGTTCCGAATCACCGATGCGGCGTCAGCAGGACTGGCCTAGCGGAGGCTCT

||||||||||||||||||||||||||||||||||||||||||||||||||||

CAGATTCTGTTCCGAATCACCGATGCGGCGTCAGCAGGACTGGCCTAGCGGAGTGAGGACGCAGTGAAAAGATGGGGGTCTGTGAACCAGGAATCGAC

||||||||||||||||||||||||||||||||||||||||||||||

TCTTTCCACCACGTGAGGACGCAGTGAAAAGATGGGGGTCTGTGAACCAGGAATCGAC

< S’alpha_I

< S’mu_1 S’mu_2 >

ACAGATTCTGTTCCGAATCACCGATGCGGCGTCAGCAGGACTGGCCTAGCGGAGGCTCT

||||||||||||||||||| ||||||||||||||||||||||||||||||

ACAGATTCTGTTCCGAATCGCCGATGCGGCGTCAGCAGGACTGGCCTAGCTTCGAGAACCGTGAGAAATGAATGTTGCTGCTGAACCCACCAGTCTATGG

|||||||||||||||||||||||||||||||||||||||||||||||||||||

TCTTTCCACCACGTGAGGAC...77 nt...ACTTCCAGCTTCGAGAACCGTGAGAAATGAATGTTGCTGCTGAACCCACCAGTCTATGG

< S’alpha_I

< S’mu_1 S’mu_2 >

TTCTGTTCCGAATCACCGATGCGGCGTCAGCAGGACTGGCCTAGCGGAGGCTCT

||||||||||||||||||||||||||||||||||||||||||||||

TTCTGTTCCGAATCACCGATGCGGCGTCAGCAGGACTGGCCTAGCG[insert]GTGAGGACGCAGTGAAAAGATGGGGGTCTGTGAACCAGGAATCGACC

|||||||||||||||||||||||||||||||||||||||||||||||

TCTTTCCACCACGTGAGGACGCAGTGAAAAGATGGGGGTCTGTGAACCAGGAATCGACC

< S’alpha_I

[insert]: ATGCGGCGTCAGCAGGACTGGCCTAGCGGAGGCTCCACCAT

< S’mu_1 S’mu_2 >

TTCCGAATCACCGATGCGGCGTCAGCAGGACTGGCCTAGCGGAGGCTCT

||||||||||||||||||||||||||||||||||||||||||||||

TTCCGAATCACCGATGCGGCGTCAGCAGGACTGGCCTAGCGGAGGCTCT[insert]GTGAGGACGCAGCGAAAAGATGGGGGTCTGTGAACCAGGA

|||||||||||| |||||||||||||||||||||||||||

TCTTTCCACCACGTGAGGACGCAGTGAAAAGATGGGGGTCTGTGAACCAGGA

< S’alpha_I

[insert]: TCTGGGTTTATGAGATGTTTCATAAAAATATTCTGGAATTCAAAGCAAAGCACTATACGTAAAGTTACAAGCAAGTGGAAGGCAAGATGTTTCAACAGTTTTGTACTCAGCCATTTATGACCAACTCCAACGGTCACTGCAATCCTGACATTTCAGA

< S’mu_1 S’mu_2 >

AAGGACAGATTCTGTTCCGAATCACCGATGCGGCGTCAGCAGGACTGGCCTAGCGGAGGCTCT

|||||||||||||||||||||||||||||||||||||||||||

AAGGACAGATTCTGTTCCGAATCACCGATGCGGCGTCAGCAGGCGGCGTCAGCAGGACTGGCCTAGAGGACGCAGTGAAAAGATGGGGGTCTGT

|||||||||||||||||||||||||||||

TCTTTCCACCACGTGAGGACGCAGTGAAAAGATGGGGGTCTGT

< S’alpha_I

< S’mu_1 S’mu_2 >

GTTCCGAATCACCGATGCGGCGTCAGCAGGACTGGCCTAGCGGAGGCTCT

||||||||||||||||||||||||||||||

GTTCCGAATCACCGATGCGGCGTCAGCAGGCGGCGTCACAGACCCCCATCTTTTCACTGACGCCGCCTGCTGAGGACGCAGTGAAAAGATGGGGGTCTGT

||||||||||||||||||||||||||||||

TCTTTCCACCACGTGAGGACGCAGTGAAAAGATGGGGGTCTGT

< S’alpha_I

< S’mu_1 S’mu_2 >

TTCTGTTCCGAATCACCGATGCGGCGTCAGCAGGACTGGCCTAGCGGAGGCTCT

||||||||||||||||||||||||||||||||||||||

TTCTGTTCCGAATCACCGATGCGGCGTCAGCAGGACTGACCATGTGAGGACGCAGTGAAAAGATGGGGGACTGTGAACCAGGAATCGACCCT

|||| |||||||||||||||||||||||||||||||||||||||||||||||||

TCTTTCCACCACGTGAGGACGCAGTGAAAAGATGGGGGTCTGTGAACCAGGAATCGACCCT

< S’alpha_I

< S’mu_1 S’mu_2 >

GGACAGATTCTGTTCCGAATCACCGATGCGGCGTCAGCAGGACTGGCCTAGCGGAGGCTCT

||||||||||||||||||||||||||||||||||||||||||||||||||

GGACAGATTCTGTTCCGAATCACCGATGCGGCGTCAGCAGGACTGGCCTAAAAGATGGGGGTCTGTGAACCAGGAATCGACCCTTCCTGGAACTTGACCC

|||||||||||||||||||||||||||||||||||||||||||||||||||

TCTTTCCACCACGTGAGGACGCAGTGAAAAGATGGGGGTCTGTGAACCAGGAATCGACCCTTCCTGGAACTTGACCC

< S’alpha_I S’alpha_II >

< S’mu_1 S’mu_2 >

CTTAGAAGGACAGATTCTGTTCCGAATCACCGATGCGGCGTCAGCAGGACTGGCCTAGCGGAGGCTCT

||||||||||||||||||||||||||||||||||||||||||||||||||

CTTAGAAGGACAGATTCTGTTCCGAATCACCGATGCGGCGTCAGCAGGACCGACCATGTGAGGACGCAGTGAAAAGATGGGGGTCTGTGAACCAGGAAT

| |||| ||||||||||||||||||||||||||||||||||||||||||

TCTTTCCACCACGTGAGGACGCAGTGAAAAGATGGGGGTCTGTGAACCAGGAAT

< S’alpha_I

S’mu_2 >

TATCAACTTCTAAACTGCATTCATTTTTAAAGTAAGATGTTTAAGAAATTAAACAG...410 nt...ACTGGCCTAGCGGAGGCTCT

||||||||||||||||||||||||||||||||||||||||||||||||||

TATCAACTTCTAAACTGCATTCATTTTTAAAGTAAGATGTTTAAGAAATTCCTGGAACTTGACCCTGCTGCCATCCTGACCTTGAACTTCCAGCTTC

|||||||||||||||||||||||||||||||||||||||||||||||||

TCTTTCCACCACGTGAGGAC...34 nt...CGACCCTTCCTGGAACTTGACCCTGCTGCCATCCTGACCTTGAACTTCCAGCTTC

< S’alpha_I

< S’mu_1 S’mu_2 >

GGACTGTGGAGACACCTTAGAAGGACAGATTCTGTTCCGAATCACCGATGCGGCGTCAGCAGGACTGGCCTAGCGGAGGCTCT

|||||||||||||||||||||||||||||||||||||||||||||||||||||

GGACTGTGGAGACACCTTAGAAGGACAGATTCTGTTCCGAATCACCGATGCGGAAAAGATGGGGGTCTGTGAACCAGGAATCGACCCTTCCTGGAACTTGAC

||||||||||||||||||||||||||||||||||||||||||||||||||

TCTTTCCACCACGTGAGGACGCAGTGAAAAGATGGGGGTCTGTGAACCAGGAATCGACCCTTCCTGGAACTTGAC

< S’alpha_I S’alpha_II >

< S’mu_1 S’mu_2 >

AGGACAGATTCWGTTCCGAATCACCGATGCGGCGTCAGCAGGACTGGCCTAGCGGAGGCTCT

||||||||||| |||||||||||||||||||||||||||||||||||

AGGACAGATTCTGTTCCGAATCACCGATGCGGCGTCAGCAGGACTGGCCACCACGTGAGGACGCAKTGAAAAGATRGGGGTCTGTGAACCAGGA

|||||||||||||||||| ||||||||| ||||||||||||||||||

TCTTTCCACCACGTGAGGACGCAGTGAAAAGATGGGGGTCTGTGAACCAGGA

< S’alpha_I

< S’mu_1 S’mu_2 >

AGGACAGATTCTGTTCCGAATCACCGATGCGGCGTCAGCAGGACTGGCCTAGCGGAGGCTCT

||||||||||||||||||||||||||||||||||| | |||||

AGGACAGATTCTGTTCCGAATCACCGATGCGGCGTAACCAGGAATCGACCCTTCCTGGAACTTGACCCTGCTGCCATCCTGACCTTG

||||||||||||||||||||||||||||||||||||||||||||||||||||

TCTTTCCACCACGTGAGGAC...18 nt...TCTGTGAACCAGGAATCGACCCTTCCTGGAACTTGACCCTGCTGCCATCCTGACCTTG

< S’alpha_I S’alpha_II >

< S’mu_1 S’mu_2 >

GACTGTGGAGACACCTTAGAAGGACAGATTCTGTTCCGAATCACCGATGCGGCGTCAGCAGGACTGGCCTAGCGGAGGCTCT

|||||||||||||||||||| ||||||||||||||||||||||||||||||||||

GACTGTGGAGACACCTTAGAGGGACAGATTCTGTTCCGAATCACCGATGCGGCGTGAGGACGCAGTGAAAAGATGGGGGTCTGTGAACCAGGAATCGACCC

|||||||||||||||||||||||||||||||||||||||||||||||||

TCTTTCCACCACGTGAGGACGCAGTGAAAAGATGGGGGTCTGTGAACCAGGAATCGACCC

< S’alpha_I

S’mu_2 >

CCTGAATTATTTCAGTTAAGCATGTTAGTTGGTGGCATAAGAGAAAACTCAATC...80 nt...ACTGGCCTAGCGGAGGCTCT

||||| ||||||||||||||||||||||||||||||||||||||||||

CCTGAGTTATTTCAGTTAAGCATGTTAGTTGGTGGCATAAGAGAAAACAAATCGACCCTTCCTGGAACTTGACCCTGCTGCCATCCTGACCTTGAACTTCCA

|||||||||||||||||||||||||||||||||||||||||||||||||||||

TCTTTCCACCACGTGAGGAC...25 nt...ACCAGGAATCGACCCTTCCTGGAACTTGACCCTGCTGCCATCCTGACCTTGAACTTCCA

< S’alpha_I

< S’mu_1 S’mu_2 >

TCTGTTCCGAATCACCGATGCGGCGTCAGCAGGACTGGCCTAGCGGAGGCTCT

|||||||||||||||||||||||||||||||||||||||||||||

TCTGTTCCGAATCACCGATGCGGCGTCAGCAGGACTGGCCTAGCGGAGGACGCAGTGAAAAGATGGGGGTCTGTGAACCAGGAATCGACCCTTCCTGGAAC

||||||||||||||||||||||||||||||||||||||||||||||||||||||||

TCTTTCCACCACGTGAGGACGCAGTGAAAAGATGGGGGTCTGTGAACCAGGAATCGACCCTTCCTGGAAC

< S’alpha_I S’alpha_II >

< S’mu_1 S’mu_2 >

ATTCTGTTCCGAATCACCGATGCGGCGTCAGCAGGACTGGCCTAGCGGAGGCTCT

|||||||||||||||||||||||||||||||||||||||||||||||

ATTCTGTTCCGAATCACCGATGCGGCGTCAGCAGGACTGGCCTAGCGCGTGGACGCAGTGAAAAGATGGGGGTCTGTGAACCAGGAATCGACCCTTC

|||||||||||||||||||||||||||||||||||||||||||||||

TCTTTCCACCACGTGAGGACGCAGTGAAAAGATGGGGGTCTGTGAACCAGGAATCGACCCTTC

< S’alpha_I

**BJAB, S’μ-S’α junctions from 3’DSBs, long overhangs (Cas9 N863A, S’μ_1 + S’μ_2 + S’α_1 + S’α_3)**

< S’mu_1 S’mu_2 >

TAGTGCTGAAGACAGGACTGTGGAGACACCTTAGAAGGACAGATTCTGTTCCGAATCACCGATGCGGCGTCAGCAGGACTGGCCTAGCGGAGGCTCT

||||||||||||||||||||||||||||||||||||||||||||||||||||||

TAGTGCTGAAGACAGGACTGTGGAGACACCTTAGAAGGACAGATTCTGTTCCGAGAAAAGGGACCCCAGAGAGCAGCCCTGCCCTCTTTCCACCATGTGAGGACGCAGT

|||||||||||||||||||||||||||||||||||||||||| |||||||||||||

GTGTGACAGCCGTCATCCCA...95 nt...CCCTAAAGAAAAGGGACCCCAGAGAGCAGCCCTGCCCTCTTTCCACCACGTGAGGACGCAGT

< S’alpha_1

S’mu_2 >

GGTTAACTCGTTTTCTCTTTGTGATTAAGGAGAAACACTTTGATATTCTGATA...219 nt...ACTGGCCTAGCGGAGGCTCT

|||||||||||||||||||||||||||||||||||||||||||||||

GGTTAACTCGTTTTCTCTTTGTGATTAAGGAGAAACACTTTGATATT[insert]TCTTATCCCACCACGTGAGGACGCAGTGAAAAGATGGGGGTCTGTGAA

|||| | |||||||||||||||||||||||||||||||||||||||||

GTGTGACAGCCGTCATCCCA...126 nt...CTGCCCTCTT-T-CCACCACGTGAGGACGCAGTGAAAAGATGGGGGTCTGTGAA

< S’alpha_1

[insert]: ACATTCATTGTCTAGCAAATAGAGAAGCCTAGAGAAAGTTTTCTAGGATAGAGCAATGTAGTTTTGCATTCATACAATTAGAACTAT

< S’mu_1 S’mu_2 >

AGACAGGACTGTGGAGACACCTTAGAAGGACAGATTCTGTTCCGAATCACCGATGCGGCGTCAGCAGGACTGGCCTAGCGGAGGCTCT

||||||||||||||||||||||||||||||||||||||||||||||

AGACAGGACTGTGGAGACACCTTAGAAGGACAGATTCTGTTCCGAAGTCTGCCCGAAACTCCTGTGTGACAGCCGTCATCCCAGAGTGAGAGCA

|||||||||||||||||||||||||||||||

GTGTGACAGCCGTCATCCCAGAGTGAGAGCA

< S’alpha_1

< S’mu_1 S’mu_2 >

GACTGTGGAGACACCTTAGAAGGACAGATTCTGTTCCGAATCACCGATGCGGCGTCAGCAGGACTGGCCTAGCGGAGGCTCT

|||||||||||||||||||||||||||||||||||||||||||||||||||||

GACTGTGGAGACACCTTAGAAGGACAGATTCTGTTCCGAATCACCGATGCGGCTCCAACGCAACGGCCAGCTCTCTGGTGACCCACAGTAGGAGTGCCACCT

||||||||||||||||||||||||||||||||||||||||||||||||||

GTGTGACAGCCGTCATCCCA...687 nt...GGATCTCTCCAACGCAACGGCCAGCTCTCTGGTGACCCACAGTAGGAGTGCCACCT

< S’alpha_1

< S’mu_1 S’mu_2 >

ACCTTAGAAGGACAGATTCTGTTCCGAATCACCGATGCGGCGTCAGCAGGACTGGCCTAGCGGAGGCTCT

||||||||||||||||||||||||||||||||||||||||||||||||||||

ACCTTAGAAGGACAGATTCTGTTCCGAATCACCGATGCGGCGTCAGCAGGAC[insert]CATCCCAGAGTGAGAGCATGCAGAGCTGGGGCCTCACGAAGGTGGTTAGGGCC

|||||||||||||||||||||||||||||||||||| | ||||||||||||||

GTGTGACAGCCGTCATCCCAGAGTGAGAGCATGCAGAGCTGGGGCCTCAGGGAGGTGGTTAGGGCC

< S’alpha_1

[insert]: CAGGAACTGGACATCAACCGGCTGTCCGACTACGATGTGGACCATATCGTGCCTCAGAGCTTTCTGAAGGACGACTCCATCGACAACAAGGTGCTGACCAGAAGCGACAAGGCCCGGGGCAAGAGCGACAACGTGCCCTCCGAAGAGGTCGTGAAGAAGATGAAGAACTACTGGCGGCGGCCTCAGTGAGCGAGCGAGCGCGCAGAGGGGGAGTGGCCAACTCCATCACTAGGGGTTCCTGCGGCCGCTCCCCAG

< S’mu_1 S’mu_2 >

TTCCGAATCACCGATGCGGCGTCAGCAGGACTGGCCTAGCGGAGGCTCT

||||||||||||||||||||||||

TTCCGAATCACCGATGCGGCGTCACCGATGCGGCGTCAGCAGGACTGGCC[rest of insert]TTATTGCAGCCAGAAATGACTAAGA

|||||||||||||||||||||||||

GTGTGACAGCCGTCATCCCA...290 nt...TTTTTGTTATTGCAGCCAGAAATGACTAAGA

< S’alpha_1

[rest of insert]: CAGTAAGGAAAATCTTAGAAATGTTAAAAAAAAAAAAAAAAAAAAAGAAAAGTAATTCAAAGGATGAAAAATGACTATTTGCATAATATTTACCACAGTATTATATAGTGACAAGAAATGAGACCAACAA

< S’mu_1 S’mu_2 >

AGACAGGACTGTGGAGACACCTTAGAAGGACAGATTCTGTTCCGAATCACCGATGCGGCGTCAGCAGGACTGGCCTAGCGGAGGCTCT

||||||||||||||||||||||||||||||||||||||||||||||||||||

AGACAGGACTGTGGAGACACCTTAGAAGGACAGATTCTGTTCCGAATCACCGTCATCCCAGAGTGAGAGCATGCAGAGCTGGGGCCTCAGGGAGGTGG

|||||||||||||||||||||||||||||||||||||||||||||||||

GTGTGACAGCCGTCATCCCAGAGTGAGAGCATGCAGAGCTGGGGCCTCAGGGAGGTGG

< S’alpha_1

< S’mu_1 S’mu_2 >

ACACCTTAGAAGGACAGATTCTGTTCCGAATCACCGATGCGGCGTCAGCAGGACTGGCCTAGCGGAGGCTCT

||||||||||||||||||||||||||||||||||||||||||||||||||||||||||

ACACCTTAGAAGGACAGATTCTGTTCCGAATCACCGATGCGGCGTCAGCAGGACTGGCGAGCATGCAGAGCTGGGGCCTCAGGGAGGTGGTTAGGGCCTGAGGGGGAA

||||||||||||||||||||||||||||||||||||||||||||||||||

GTGTGACAGCCGTCATCCCAGAGTGAGAGCATGCAGAGCTGGGGCCTCAGGGAGGTGGTTAGGGCCTGAGGGGGAA

< S’alpha_1

< S’mu_1 S’mu_2 >

GACAGATTCTGTTCCGAATCACCGATGCGGCGTCAGCAGGACTGGCCTAGCGGAGGCTCT

|||||||||||||||||||||||||||||||||||||||||||||||||||||

GACAGATTCTGTTCCGAATCACCGATGCGGCGTCAGCAGGACTGGCCTAGCGGCCCTGCCCTCTTTCCACCACGTGAGGACGCAGTGAAAAGATGGGGGTCT

||| ||||||||||||||||||||||||||||||||||||||||||||||||||

GTGTGACAGCCGTCATCCCA...113 nt...CCAGAGAGCAGCCCTGCCCTCTTTCCACCACGTGAGGACGCAGTGAAAAGATGGGGGTCT

< S’alpha_1

< S’mu_1 S’mu_2 >

ATAGTGCTGAAGACAGGACTGTGGAGACACCTTAGAAGGACAGATTCTGTTCCGAATCACCGATGCGGCGTCAGCAGGACTGGCCTAGCGGAGGCTCT

|||||||||||||||||||||||||||||||||||||||||||||||||||||||

ATAGTGCTGAAGACAGGACTGTGGAGACACCTTAGAAGGACAGATTCTGTTCCGAGAAGAGGGACCCCAGAGAGCAGCCCTGCCCTCTTTCCACCATGTGAGGACGCAGT

|||| ||||||||||||||||||||||||||||||||||||| |||||||||||||

GTGTGACAGCCGTCATCCCA...95 nt...CCCTAAAGAAAAGGGACCCCAGAGAGCAGCCCTGCCCTCTTTCCACCACGTGAGGACGCAGT

< S’alpha_1

< S’mu_1 S’mu_2 >

GAAGACAGGACTGTGGAGACACCTTAGAAGGACAGATTCTGTTCCGAATCACCGATGCGGCGTCAGCAGGACTGGCCTAGCGGAGGCTCT

|||||||||||||||||||||||||||||||||||||||||||||

GAAGACAGGACTGTGGAGACACCTTAGAAGGACAGATTCTGTTCCTAGGAAAGGGACCCCAGAGAGCAGCCCTGCCCTCTTTCCACCATGTGAGGACGC

||||||||||||||||||||||||||||||||||||||| ||||||||||

GTGTGACAGCCGTCATCCCA...98 nt...TAAAGAAAAGGGACCCCAGAGAGCAGCCCTGCCCTCTTTCCACCACGTGAGGACGC

< S’alpha_1

< S’mu_1 S’mu_2 >

CACCTTAGAAGGACAGATTCTGTTCCGAATCACCGATGCGGCGTCAGCAGGACTGGCCTAGCGGAGGCTCT

|||||||||||||||||||||||||||||||||||||||||||||||||||||

CACCTTAGAAGGACAGATTCTGTTCCGAATCACCGATGCGGCGTCAGCAGGACTGACAGCCGTCATCCCAGAGTGAGAGCATGCAGAGCTGGGGCCTCAGGGAGGTGGT

||||||||||||||||||||||||||||||||||||||||||||||||||||||||

GTGTGACAGCCGTCATCCCAGAGTGAGAGCATGCAGAGCTGGGGCCTCAGGGAGGTGGT

< S’alpha_1

S’mu_2 >

GACTGGCCTAGCGGAG-GCTCT CCGATGCGGCGTCAGCAGGACTGGCCTAGCGGAG-GCT

|||||||||||||||| ||| |||||||||||||||||||||||||||||||||| |||

GACTGGCCTAGCGGAGAGCTAGAGCT...AGACCCCCGATGCGGCGTCAGCAGGACTGGCCTAGCGGAGAGCTGGGGCCTCAGGGAGGTGGTTAGGGCCTGAGGGG

||||||...|||||| |||||||||||||||||||||||||||||||||||||||

AGAGCT...AGACCC <S’alpha_1...14 nt...AGAGCTGGGGCCTCAGGGAGGTGGTTAGGGCCTGAGGGG

< S’mu_1 S’mu_2 >

TAGAAGGACAGATTCTGTTCCGAATCACCGATGCGGCGTCAGCAGGACTGGCCTAGCGGAGGCTCT

|||||||||||||||||||||||||||||||||||||||||||||||||||||||

TAGAAGGACAGATTCTGTTCCGAATCACCGATGCGGCGTCAGCAGGACTGGCCTAGTCATCCCAGAGTGAGAGCATGCAGAGCTGGGGCCTCAGGGAGGTGGTT

|||||||||||||||||||||||||||||||||||||||||||||||||

GTGTGACAGCCGTCATCCCAGAGTGAGAGCATGCAGAGCTGGGGCCTCAGGGAGGTGGTT

< S’alpha_1

S’mu_2 >

CACCGATGCGGCGTCAGCAGGACTGGCCTAGCGGAGGCTCT

||||||||||||||||||

CACCGATGCGGCGTCAGCGGCGTCAGCAGGACTGGCCTAGCGGGCATGC...CTAGAGCCGTCA...CTAGAGCCGTCATCCCAGAGTGAGAGCATGCAGAGC

||||||||||||||||||||||||||||||

GTGTGACAGCCGTCATCCCAGAGTGAGAGCATGCAGAGC

< S’alpha_1

< S’mu_1 S’mu_2 >

ACACCTTAGAAGGACAGATTCTGTTCCGAATCACCGATGCGGCGTCAGCAGGACTGGCCTAGCGGAGGCTCT

|||||||||||||||||||||||||||||||||||||||||||||||||||||

ACACCTTAGAAGGACAGATTCTGTTCCGAATCACCGATGCGGCGTCAGCAGGAGAGTGAGAGCATGCAGAGCTGGGGCCCCAGGGAGGTGGTTAGGGCCTGAGGGGGAACCC

||||||||||||||||||||||||||| ||||||||||||||||||||||||||||||||

GTGTGACAGCCGTCATCCCAGAGTGAGAGCATGCAGAGCTGGGGCCTCAGGGAGGTGGTTAGGGCCTGAGGGGGAACCC

< S’alpha_1

< S’mu_1 S’mu_2 >

TAGAAGGACAGATTCTGTTCCGAATCACCGATGCGGCGTCAGCAGGACTGGCCTAGCGGAGGCTCT

|||||||||||||||||||||||||||||||||||||||||||||||||||||||||||

TAGAAGGACAGATTCTGTTCCGAATCACCGATGCGGCGTCAGCAGGACTGGCCTAGCGGGAGCATGCAGAGCTGGGGCCTCAGGGAGGTGGTTAGGGCCTGAGGGGGAACC

||||||||||||||||||||||||||||||||||||||||||||||||||||

GTGTGACAGCCGTCATCCCAGAGTGAGAGCATGCAGAGCTGGGGCCTCAGGGAGGTGGTTAGGGCCTGAGGGGGAACC

< S’alpha_1

S’mu_2 >

AGGTAAAATGTGCATCATTATCCTGAATTATTTCAGTTAAGCATGTTAGTTGGTGGCAT...96 nt...ACTGGCCTAGCGGAGGCTCT

|||||||||||||||||||||||||||||||||||||||||||||||||||||

AGGTAAAATGTGCATCATTATCCTGAATTATTTCAGTTAAGCATGTTAGTTGGCCAGGGCATCTTCCATTCACGCCCACGCTTTCCAGGACCCCGCTGCAGCAGCTC

||||||||||||||||||||||||||||||||||||||||| |||||||||||||||

GTGTGACAGCCGTCATCCCA...530 nt...GCCCCATGGCCAGGGCATCTTCCATTCACGCCCACGCTTTCCAGGACTCCGCTGCAGCAGCTC

< S’alpha_1

< S’mu_1 S’mu_2 >

ATTCTGTTCCGAATCACCGATGCGGCGTCAGCAGGACTGGCCTAGCGGAGGCTCT

|||||||||||||||||||||||||||||||||||||||||||

ATTCTGTTCCGAATCACCGATGCGGCGTCAGCAGGACTGGCCT[insert]TTTTTGTTATTGCAGCCAGAAATGACTAAGACGCAGCAGCCACGTC

||||||||||||||||||||||||||||||| ||||||||||||||

GTGTGACAGCCGTCATCCCA...284 nt...ATGGTATTTTTGTTATTGCAGCCAGAAATGACTAAGATGCAGCAGCCACGTC

< S’alpha_1

[insert]: GGTGTGTGAACTTCGTCCTGTTTTGTCTGTTTGTCAATAAGTTCTTCTGGGGCTGTGAGTTTTCTACATTCTCACAAGAACGTTACTTAAT

< S’mu_1 S’mu_2 >

CTGTTCCGAATCACCGATGCGGCGTCAGCAGGACTGGCCTAGCGGAGGCTCT

|||||||||||||||||||||||||||||||||||||||

CTGTTCCGAATCACCGATGCGGCGTCAGCAGGACTGGCCACCGATGCGGCGTCAGCAGGACTGGCCTAGAGTGAGAGCATGCAGAGCTGGGGCCTCAGGGAG

|||||||||||||||||||||||||||||||||||

GTGTGACAGCCGTCATCCCAGAGTGAGAGCATGCAGAGCTGGGGCCTCAGGGAG

< S’alpha_1

< S’mu_1 S’mu_2 >

TTCCGAATCACCGATGCGGCGTCAGCAGGACTGGCCTAGCGGAGGCTCT

|||||||||||||||||||||||||||||||

TTCCGAATCACCGATGCGGCGTCAGCAGGACGGCGTCAGCAGGACTGGCCTAGCAGTGAGAGCATGCAGAGCTGGGGCCTCAGGGAGGTGGT

||||||||||||||||||||||||||||||||||||||

GTGTGACAGCCGTCATCCCAGAGTGAGAGCATGCAGAGCTGGGGCCTCAGGGAGGTGGT

< S’alpha_1

S’mu_2 >

GCGGCGTCAGCAGGACTGGCCTAGCGGAGGCTCT

|||||||||||||

GCGGCGTCAGCAGTGAGAG...CTAGAGCCGTCA...ATTCGGCAGAGC...CTAGAGCCGTCATCCCAGAGTGAGAGCATGCGGAGCTGGGGCCTCAG

||||||||||||||||||||||||| |||||||||||||||

GTGTGACAGCCGTCATCCCAGAGTGAGAGCATGCAGAGCTGGGGCCTCAG

< S’alpha_1

< S’mu_1 S’mu_2 >

TGTGGAGACACCTTAGAAGGACAGATTCTGTTCCGAATCACCGATGCGGC-GTCAGCAGGACTGGCCTAGCGGAGGCTCT

|||||||||||||||||||||||||||||||||||||||||||||||||| ||||

TGTGGAGACACCTTAGAAGGACAGATTCTGTTCCGAATCACCGATGCGGCCGTCATCCCAGAGTGAGAGCATGCAGAGCTGGGGCCTCAGGGAGGTGGTTAGGGCC

||||||||||||||||||||||||||||||||||||||||||||||||||||||||||

GTGTGACAGCCGTCATCCCAGAGTGAGAGCATGCAGAGCTGGGGCCTCAGGGAGGTGGTTAGGGCC

< S’alpha_1

< S’mu_1 S’mu_2 >

AGGACAGATTCTGTTCCGAATCACCGATGCGGCGTCAGCAGGACTGGCCTAGCGGAGGCTCT

||||||||||||||||||||||||||||||||||||||||||||||||||||||

AGGACAGATTCTGTTCCGAATCACCGATGCGGCGTCAGCAGGACTGGCCTAGCGAAAAGGGACCCCAGAGAGCAGCCCTGCCCTCTTTCCACCACGTGAGGA

|||| |||||||||||||||||||||||||||||||||||||||||||||||||

GTGTGACAGCCGTCATCCCA...90 nt...AGAGACCCTAAAGAAAAGGGACCCCAGAGAGCAGCCCTGCCCTCTTTCCACCACGTGAGGA

S’alpha_1

< S’mu_1 S’mu_2 >

AGGACAGATTCTGTTCCGAATCACCGATGCGGCGTCAGCAGGACTGGCCTAGCGGAGGCTCT

||||||||||| |||||||||||||||||||||||||||||||||||||||||

AGGACAGATTCCGTTCCGAATCACCGATGCGGCGTCAGCAGGACTGGCCTAGCGCCGTCATCCCAGAGTGAGAGCATGCAGAGCTGGGGCCTCAGGGAGGTGGTTAGGGC

|||||||||||||||||||||||||||||||||||||||||||||||||||||||||

GTGTGACAGCCGTCATCCCAGAGTGAGAGCATGCAGAGCTGGGGCCTCAGGGAGGTGGTTAGGGC

< S’alpha_1

< S’mu_1 S’mu_2 >

TGTTCCGAATCACCGATGCGGCGTCAGCAGGACTGGCCTAGCGGAGGCTCT

|||||||||||||||||||||||||||||||||||||||||||

TGTTCCGAATCACCGATGCGGCGTCAGCAGGACTGGCCTAGCGCGTCAGCAGGACTGGCCTAGCATGCAGAGCTGGGGCCTCAGGGAGGTGGTTA

||||||||||||||||||||||||||||||||||

GTGTGACAGCCGTCATCCCAGAGTGAGAGCATGCAGAGCTGGGGCCTCAGGGAGGTGGTTA

< S’alpha_1

< S’mu_1 S’mu_2 >

CACCTTAGAAGGACAGATTCTGTTCCGAATCACCGATGCGGCGTCAGCAGGACTGGCCTAGCGGAGGCTCT

||||||||||||||||||||||||||||||||||||||||||||||||||

CACCTTAGAAGGACAGATTCTGTTCCGAATCACCGATGCGGCGTCAGCAGCCGTCATCCCAGAGTGAGAGCATGCAGAGCTGGGGCCTCAGGGAGGTGGTTA

|||||||||||||||||||||||||||||||||||||||||||||||||||||||

GTGTGACAGCCGTCATCCCAGAGTGAGAGCATGCAGAGCTGGGGCCTCAGGGAGGTGGTTA

< S’alpha_1

< S’mu_1 S’mu_2 >

AGACAGGACTGTGGAGACACCTTAGAAGGACAGATTCTGTTCCGAATCACCGATGCGGCGTCAGCAGGACTGGCCTAGCGGAGGCTCT

||||||||||||||||||||||||||||||||||||||||||||||||||||||||

AGACAGGACTGTGGAGACACCTTAGAAGGACAGATTCTGTTCCGAATCACCGATGCCAGGGCATCTTCCATTCACGCCCACGCTTTCCAGGACTCCGCTGCAGCA

|||||||||||||||||||||||||||||||||||||||||||||||||||

GTGTGACAGCCGTCATCCCA...532 nt...CCCATGGCCAGGGCATCTTCCATTCACGCCCACGCTTTCCAGGACTCCGCTGCAGCA

< S’alpha_1

< S’mu_1 S’mu_2 >

TTAGAAGGACAGATTCTGTTCCGAATCACCGATGCGGCGTCAGCAGGACTGGCCTAGCGGAGGCTCT

|||||||||||||||||||||||||||||||||||||||||||||||||||

TTAGAAGGACAGATTCTGTTCCGAATCACCGATGCGGCGTCAGCAGGACTGGACAGCCGTCATCCCAGAGTGAGAGCATGCAGAGCTGGGGCCTCAGGGAGGTGGTTA

|||||||||||||||||||||||||||||||||||||||||||||||||||||||||

GTGTGACAGCCGTCATCCCAGAGTGAGAGCATGCAGAGCTGGGGCCTCAGGGAGGTGGTTA

< S’alpha_1

S’mu_2 >

TACTCACTTTAGGATAAGTTTTAGGTAAAATGTGCATCATTATCCTGA...129 nt...ACTGGCCTAGCGGAGGCTCT

||||||||||||||||||||||||||||||||||||||||||

TACTCACTTTAGGATAAGTTTTAGGTAAAATGTGCATCATTAGCCAGTCCTGCTGCTGGGGCCTCAGGGAGGTGGTTAGGGCCTGAGGGGGAA

|||||||||||||||||||||||||||||||||||||||

GTGTGACAGCCGTCATCCCAGAGTGAGAGCATGCAGAGCTGGGGCCTCAGGGAGGTGGTTAGGGCCTGAGGGGGAA

< S’alpha_1

< S’mu_1 S’mu_2 >

AGACACCTTAGAAGGACAGATTCTGTTCCGAATCACCGATGCGGCGTCAGCAGGACTGGCCTAGCGGAGGCTCT

|||||||||||||||||||||||||||||||||||||||||||||||||||||||

AGACACCTTAGAAGGACAGATTCTGTTCCGAATCACCGATGCGGCGTCAGCAGGAAGGGACCCCAGAGAGCAGCCCTGCCCTCTTTCCACCATGTGAGGACGCAGTG

|||||||||||||||||||||||||||||||||||||| ||||||||||||||

GTGTGACAGCCGTCATCCCA...99 nt...AAAGAAAAGGGACCCCAGAGAGCAGCCCTGCCCTCTTTCCACCACGTGAGGACGCAGTG

< S’alpha_1

< S’mu_1 S’mu_2 >

GAAGGACAGATTCTGTTCCGAATCACCGATGCGGCGTCAGCAGGACTGGCCTAGCGGAGGCTCT

|||||||||||| ||||||||||||||||||||||||||||||||||||||

GAAGGACAGATTATGTTCCGAATCACCGATGCGGCGTCAGCAGGACTGGCCGATGCCCAGAGTGAGAGCATGCAGAGCTGGGGCCTCAGGGAGGTGGCTAGGGCCT

|||||||||||||||||||||||||||||||||||||||||| ||||||||

GTGTGACAGCCGTCATCCCAGAGTGAGAGCATGCAGAGCTGGGGCCTCAGGGAGGTGGTTAGGGCCT

< S’alpha_1

< S’mu_1 S’mu_2 >

ATTCTGTTCCGAATCACCGATGCGGCGTCAGCAGGACTGGCCTAGCGGAGGCTCT

||||||||||||||||||||||||||||||||||||||||||||||||||||

ATTCTGTTCCGAATCACCGATGCGGCGTCAGCAGGACTGGCCTAGCGGAGGCGGCGTCAGAGTGAGAGCATGCAGAGCTGGGGCCTCAGGGAGGTGGTTAGGGCCTGAG

||||||||||||||||||||||||||||||||||||||||||||||||||||

GTGTGACAGCCGTCATCCCAGAGTGAGAGCATGCAGAGCTGGGGCCTCAGGGAGGTGGTTAGGGCCTGAG

< S’alpha_1

**BJAB, S’μ-BCL6 junctions from blunt DSBs (Cas9 WT, S’μ_1 + BCL6_1)**

< S’mu_1

GAGAAAACTCAATCAGATAGTGCTGAAGACAGGACTGTGGAGACACCTTAGaaggacagattctgttccgaatcaccgatgcggcgtcag

||||||||||||||||||||||||||||||| |||||||||||||||||||

GAGAAAACTCAATCAGATAGTGCTGAAGACAAGACTGTGGAGACACCTTAGTATTTTACCTTTTAATTCttttttttttCCGCTCTTGCCAAATGCTTTGGCTCCAAGTTT

|||||||||||||||||| |||||||||||||||||||||||||||||||||||||||||

CCGCTGCTCATGATCATTATTTTACCTTTTAATTC-TTTTTTTTTCCGCTCTTGCCAAATGCTTTGGCTCCAAGTTT

< BCL6_1

< S’mu_1

GATAGTGCTGAAGACAGGACTGTGGAGACACCTTAGAAGGACAGATTCTGTTCCGAatcaccgatgcggcgtcag

|||||||||||||||||||||||||||||||||||||||||||||||||||||||||||

GATAGTGCTGAAGACAGGACTGTGGAGACACCTTAGAAGGACAGATTCTGTTCCGAATCTTTTACCTTTTAATTC-ttttttttCCGCTCTTGCCAAATGCTTTGGCTCCA

|||||||||||||||| |||||||||||||||||||||||||||||||||||

CCGCTGCTCATGATCATTATTTTACCTTTTAATTCTTTTTTTTTCCGCTCTTGCCAAATGCTTTGGCTCCA

< BCL6_1

< S’mu_1

GAGAAAACTCAATCAGATAGTGCTGAAGACAGGACTGTGGAGACACCTTAGAAGGacagattctgttccgaatcaccgatgcggcgtcag

|||||||||||||||||||||||||||||||||||||||||||||||||||||||

GAGAAAACTCAATCAGATAGTGCTGAAGACAGGACTGTGGAGACACCTTAGAAGGGAATTAGGGGGCGGCCGGAGCAGAGAGGACGAGACAGTGCTTGGGGGGTGATTCG

|||||||||||||||||||||||||||||||||||||||||||||||||||||||||

CCGCTGCTCATGATCATTAT...280 nt...AAGGAGGGGAATTAGGGGGCGGCCGGAGCAGAGAGGACGAGACAGTGCTTGGGGGGTGATTCG

< BCL6_1

< S’mu_1

AGACAGGACTGTGGAGACACCTTAGAAGGACAGATTCTGTTCCGAatcaccgatgcggcgtcag

|||||||||||||||||||||||||||||||||||||||||||||||

AGACAGGACTGTGGAGACACCTTAGAAGGACAGATTCTGTTCCGAATCTGCTCATGATCATTATTTTACCTTTTAATTCtttttttttCCGCTCTTGCCA

|||||||||||||||||||||||||||||||||||||||||||||||||||||

CCGCTGCTCATGATCATTATTTTACCTTTTAATTCTTTTTTTTTCCGCTCTTGCCA

< BCL6_1

< S’mu_1

AAAATGTGCATCATTATCCTGAATTATTTCAGTTAAGCATGTTAGTTGGTGGCATaagaga...66 nt...aatcaccgatgcggcgtcag

|||||||||||||||||||||||||||||||||||||||||||||||||||||||

AAAATGTGCATCATTATCCTGAATTATTTCAGTTAAGCATGTTAGTTGGTGGCATTGCTTTTTTCCGCTCTTGCCAAATGCTTTGGCTCCAAGTTTTCTATGTGTATC

||||||||||||||||||||||||||||||||||||||||||||||||||

CCGCTGCTCATGATCATTATTTTACCTTTTAATTCTTTTTTTTTCCGCTCTTGCCAAATGCTTTGGCTCCAAGTTTTCTATGTGTATC

< BCL6_1

< S’mu_1

AGACAGGACTGTGGAGACACCTTAGAAGGACAGATTCTGTTCCGAatcaccgatgcggcgtcag

|||||||||||||||||||||||||||||||||||||||||||||||

AGACAGGACTGTGGAGACACCTTAGAAGGACAGATTCTGTTCCGAATGCGGAGAGGACGCGCGCTCGCGCTCTCGCTCTTTCTGCTGCTGCTTGCGTACGGCTTGTG

||||||||||||||||||||||||||||||||||||||||||||||||||||||||||||

CCGCTGCTCATGATCATTAT...368 nt...CAGACCGCGGAGAGGACGCGCGCTCGCGCTCTCGCTCTTTCTGCTGCTGCTTGCGTACGGCTTGTG

< BCL6_1

< S’mu_1

AGACAGGACTGTGGAGACACCTTAGAAGGACAGATTCTGTTCCGAatcaccgatgcggcgtcag

||||| |||||||||||||||||||||||||||||||||||||||||

AGACAAGACTGTGGAGACACCTTAGAAGGACAGATTCTGTTCCGAATGGAGCAGAGAGGACGAGACAGTGCTTGGGGGGTGATTCGGGCTAGTCTGGGGGCTGTCTG

||||||||||||||||||||||||||||||||||||||||||||||||||||||||||||

CCGCTGCTCATGATCATTAT...298 nt...GCGGCCGGAGCAGAGAGGACGAGACAGTGCTTGGGGGGTGATTCGGGCTAGTCTGGGGGCTGTCTG

< BCL6_1

< S’mu_1

AGACAGGACTGTGGAGACACCTTAGAAGGACAGATTCTGTTCCGAatcaccgatgcggcgtcag

|||||||||||||||||||||||||||||||||||||||||||||||

AGACAGGACTGTGGAGACACCTTAGAAGGACAGATTCTGTTCCGAATCTGCTCATGATCATTATTTTACCTTTTAATTCtttttttttCCGCTCTTGCCA

|||||||||||||||||||||||||||||||||||||||||||||||||||||

CCGCTGCTCATGATCATTATTTTACCTTTTAATTCTTTTTTTTTCCGCTCTTGCCA

< BCL6_1

< S’mu_1

TCAATCAGATAGTGCTGAAGACAGGACTGTGGAGACACCTTAGAAGGACAGATTCTGttccgaatcaccgatgcggcgtcag

||||||||||||||||||||||| |||||||||||||||||||||||||||||||||

TCAATCAGATAGTGCTGAAGACAAGACTGTGGAGACACCTTAGAAGGACAGATTCTGATCATTATTTTACCTTTTAATTCtttttttttCCGCTCTTGCCAAATGCTTTGG

||||||||||||||||||||||||||||||||||||||||||||||||||||||||

CCGCTGCTCATGATCATTATTTTACCTTTTAATTCTTTTTTTTTCCGCTCTTGCCAAATGCTTTGG

< BCL6_1

< S’mu_1

CAGATAGTGCTGAAGACAGGACTGTGGAGACACCTTAGAAGGACAGATTCTGTTCCgaatcaccgatgcggcgtcag

||||||||||||||||| ||||||||||||||||||||||||||||||||||||||

CAGATAGTGCTGAAGACGGGACTGTGGAGACACCTTAGAAGGACAGATTCTGTTCCtttttttCCGCTCTTGCCAAATGCTTTGGCTCCAAGTTTTCTATGTGTATCTATTGA

|||||||||||||||||||||||||||||||||||||||||||||||||||||||||

CCGCTGCTCATGATCATTATTTTACCTTTTAATTCTTTTTTTTTCCGCTCTTGCCAAATGCTTTGGCTCCAAGTTTTCTATGTGTATCTATTGA

< BCL6_1

< S’mu_1

CAACTACTCACTTTAGGATAAGTTTTAGGTAAAATGTGCATCATTATCCTGAATTATTTCagttaa...91 nt...aatcaccgatgcggcgtcag

||||||||||||||||||||||||||||||||||||||||||||||||||||||||||||

CAACTACTCACTTTAGGATAAGTTTTAGGTAAAATGTGCATCATTATCCTGAATTATTTCtttttttttCCGCTCTTGCCAAATGCTTTGGCTCCAAGTTTTCTATGTGTATC

||| ||||||||||||||||||||||||||||||||||||||||||||||||||||||||

CCGCTGCTCATGATCATTATTTTACCTTTTAATTCTTTTTTTTTCCGCTCTTGCCAAATGCTTTGGCTCCAAGTTTTCTATGTGTATC

< BCL6_1

< S’mu_1

AGACAGGACTGTGGAGACACCTTAGAAGGACAGATTCTGTTCCGAatcaccgatgcggcgtcag

||||| |||||||||||||||||||||||||||||||||||||||||

AGACAAGACTGTGGAGACACCTTAGAAGGACAGATTCTGTTCCGAATCTGCTCATGATCATTATTTTACCTTTTAATTCtttttttttCCGCTCTTGCC

||||||||||||||||||||||||||||||||||||||||||||||||||||

CCGCTGCTCATGATCATTATTTTACCTTTTAATTCTTTTTTTTTCCGCTCTTGCC

< BCL6_1

< S’mu_1

AAACTCAATCAGATAGTGCTGAAGACAGGACTGTGGAGACACCTTAGAAGGACAGATTCtgttccgaatcaccgatgcggcgtcag

||||||||||||||||||||||||||| |||||||||||||||||||||||||||||||

AAACTCAATCAGATAGTGCTGAAGACAAGACTGTGGAGACACCTTAGAAGGACAGATTCGCTGCTCATGATCATTATTTTACCTTTTAATTC-ttttttttCCGCTCTTGCC

|||||||||||||||||||||||||||||||||| |||||||||||||||||||

CCGCTGCTCATGATCATTATTTTACCTTTTAATTCTTTTTTTTTCCGCTCTTGCC

< BCL6_1

< S’mu_1

AGATAGTGCTGAAGACAGGACTGTGGAGACACCTTAGAAGGACAGATTCTG-TTCcgaatcaccgatgcggcgtcag

||||||||||||||||| |||||||||||||||||||| |||||| ||||| |||

AGATAGTGCTGAAGACAAGACTGTGGAGACACCTTAGAGGGACAGGTTCTGATTCGGGCTAGTCTGGGGGCTGTCTGGCCCCAGACCGCGGAGAGGACGCGCGCTCGCG

||||||||||||||||||||||||||||||||||||||||||||||||||||||||||||

CCGCTGCTCATGATCATTAT...330 nt...GGGGGGTGATTCGGGCTAGTCTGGGGGCTGTCTGGCCCCAGACCGCGGAGAGGACGCGCGCTCGCG

< BCL6_1

< S’mu_1

TAGTGCTGAAGACAGGACTGTGGAGACACCTTAGAAGGACAGATTCTGTTCCGAatcaccgatgcggcgtcag

||||||||||||||||||||||||||||||||||||||||||||||||||||||||

TAGTGCTGAAGACAGGACTGTGGAGACACCTTAGAAGGACAGATTCTGTTCCGAATTTACCTTTTAATTCtttttttttCCGCTCTTGCCAAATGCTTTGGCTCCAAGTT

|||||||||||||||||||||||||||||||||||||||||||||||||||||||

CCGCTGCTCATGATCATTATTTTACCTTTTAATTCTTTTTTTTTCCGCTCTTGCCAAATGCTTTGGCTCCAAGTT

< BCL6_1

< S’mu_1

GTGCTGAAGACAGGACTGTGGAGACACCTTAGAAGGACAGATTCTG-TTCcgaatcaccgatgcggcgtcag

|||||||||||| ||||||||||||||||||||||||||||||||| |||

GTGCTGAAGACAAGACTGTGGAGACACCTTAGAAGGACAGATTCTGATTCGGGCTAGTCTGGGGGCTGTCTGGCCCCAGACCGCGGAGAGGACGCGCGCT

||||||||||||||||||||||||||||||||||||||||||||||||||||||||

CCGCTGCTCATGATCATTAT...330 nt...GGGGGGTGATTCGGGCTAGTCTGGGGGCTGTCTGGCCCCAGACCGCGGAGAGGACGCGCGCT

< BCL6_1

< S’mu_1

AAAGTAAGATGTTTAAGAAATTAAACAGTCTTAGGGAGAGTTTATGACTGTattcaa...357 nt...aatcaccgatgcggcgtcag

|||||||||||||||||||||||||||||||||||||||||||||||||||

AAAGTAAGATGTTTAAGAAATTAAACAGTCTTAGGGAGAGTTTATGACTGTTATGTTTTGGTTTTTggaaaggaggtggaggagaggaaggaggggaattaggggg

|||||||||||||||||||||||||||||||||||||||||||||||||||||

CCGCTGCTCATGATCATTAT...240 nt...TTGTGTTGTTTTGGTTTTTGGAAAGGAGGTGGAGGAGAGGAAGGAGGGGAATTAGGGGG

< BCL6_1

< S’mu_1

AGACAGGACTGTGGAGACACCTTAGAAGGACAGATTCTGTTCCGAatcaccgatgcggcgtcag

||||| |||||||||||||||||||||||||||||||||||||||

AGACAAGACTGTGGAGACACCTTAGAAGGACAGATTCTGTTCCGA[insert]GCTCATGATCATTATTTTACCTTTTAATTCtttttttttCCGCTCTTGCCA

|||||||||||||||||||||||||||||||||||||||||||||||||||

CCGCTGCTCATGATCATTATTTTACCTTTTAATTCTTTTTTTTTCCGCTCTTGCCA

< BCL6_1

[insert]: TTGGTCTTGAACAGCAGGTCCACGATGGCCTTTTTCTGCTCGCCGCTCAGGAAGGCGGGCTTTCTCATTCCCTCGGTCACGTATTTCACTTTGGTCAGCTCGTTATACACGGTGAAGTACTCGTACAGCAGGCTGTGCTTGGGCAGCACCTTCTCGTTGGGCAGGTTCTTATCGAAGTTGGTCATCCGCTCGATGAA

< S’mu_1

AGACAGGACTGTGGAGACACCTTAGAAGGACAGATTCTGTTCCGAatcaccgatgcggcgtcag

||||| ||||||||||||||||||||||||||||||||||||||||

AGACAAGACTGTGGAGACACCTTAGAAGGACAGATTCTGTTCCGAATGCTCATGATCATTATTTTACCTTTTAATTCtttttttttCCGCTCTTGCCA

||||||||||||||||||||||||||||||||||||||||||||||||||||

CCGCTGCTCATGATCATTATTTTACCTTTTAATTCTTTTTTTTTCCGCTCTTGCCA

< BCL6_1

< S’mu_1

CTCAATCAGATAGTGCTGAAGACAGGACTGTGGAGACACCTTAGAAGGACAGATTCTGttccgaatcaccgatgcggcgtcag

||||||||||||||||||||||| |||||||||||||||||||||||||||||||||||

CTCAATCAGATAGTGCTGAAGACGAGACTGTGGAGACACCTTAGAAGGACAGATTCTGttttttttCCGCTCTTGCCAAATGCTTTGGCTCCAAGTTTTCTATGTGTATCTA

||||| ||||||||||||||||||||||||||||||||||||||||||||||||||||||

CCGCTGCTCATGATCATTATTTTACCTTTTAATTCT-TTTTTTTTCCGCTCTTGCCAAATGCTTTGGCTCCAAGTTTTCTATGTGTATCTA

< BCL6_1

< S’mu_1

ATCAGATAGTGCTGAAGACAGGACTGTGGAGACACCTTAGAAGGACAGATTCTGttccgaatcaccgatgcggcgtcag

|||||||||||||||||||| ||||||||||||||||||| |||||||||||||||

ATCAGATAGTGCTGAAGACAAGACTGTGGAGACACCTTAGGAGGACAGATTCTGTTTTTTttctttttttttCCGCTCTTGCCAAATGCTTTGGCTCCAAGTTTTCTATGTGT

|||||||||||||||||||||||||||||||||||||||||||||||||||||

CCGCTGCTCATGATCATTATTTTACCTTTTAATTCTTTTTTTTTCCGCTCTTGCCAAATGCTTTGGCTCCAAGTTTTCTATGTGT

< BCL6_1

< S’mu_1

AGACAGGACTGTGGAGACACCTTAGAAGGACAGATTCTGTTCCGAatcaccgatgcggcgtcag

|||||||||||||||||| ||||||||||||||||||||||||||||

AGACAGGACTGTGGAGACGCCTTAGAAGGACAGATTCTGTTCCGAATGCGGCGTATTggaaaggaggtggaggagaggaaggaggggaattaggggggcggccgg

||||||||||||||||||||||||||||||||||||| ||||||||||||

CCGCTGCTCATGATCATTAT...251 nt...TGGTTTTTGGAAAGGAGGTGGAGGAGAGGAAGGAGGGGAATTA-GGGGGCGGCCGG

< BCL6_1

< S’mu_1

GACTGTGGAGACACCTTAGAAGGACAGATTCTGTTCCGAatcaccgatgcggcgtcag

||||||||||||||||||||||||||||||||||||||||||

GACTGTGGAGACACCTTAGAAGGACAGATTCTGTTCCGAATCTGCTCATGATCATTATTTTACCTTTTAATTCtttttttttCCGCTCTTGCCAAATGCTT

||||||||||||||||||||||||||||||||||||||||||||||||||||||||||||

CCGCTGCTCATGATCATTATTTTACCTTTTAATTCTTTTTTTTTCCGCTCTTGCCAAATGCTT

< BCL6_1

< S’mu_1

GATAGTGCTGAAGACAGGACTGTGGAGACACCTTAGAAGGACAGATTCTGTTCCGAatcaccgatgcggcgtcag

|||||||||||||||| |||||||||||||||||||||||||||||||||||||||||

GATAGTGCTGAAGACAAGACTGTGGAGACACCTTAGAAGGACAGATTCTGTTCCGAATTAATTCtttttttttCCGCTCTTGCCAAATGCTTTGGCTCCAAGTTTTCTATG

||||||||||||||||||||||||||||||||||||||||||||||||||||||

CCGCTGCTCATGATCATTATTTTACCTTTTAATTCTTTTTTTTTCCGCTCTTGCCAAATGCTTTGGCTCCAAGTTTTCTATG

< BCL6_1

< S’mu_1

AGTGCTGAAGACAGGACTGTGGAGACACCTTAGAAGGACAGATTCTGTTCCGAatcaccgatgcggcgtcag

||||||||||||| ||||||||||||||||||||||||||||||||||||||||||

AGTGCTGAAGACAAGACTGTGGAGACACCTTAGAAGGACAGATTCTGTTCCGAATCCATTATTTTACCTTTTAATTCtttttttttCCGCTCTTGCCAAATGCTTTGGCTCC

||||||||||||||||||||||||||||||||||||||||||||||||||||||||

CCGCTGCTCATGATCATTATTTTACCTTTTAATTCTTTTTTTTTCCGCTCTTGCCAAATGCTTTGGCTCC

< BCL6_1

< S’mu_1

AGTGCTGAAGACAGGACTGTGGAGACACCTTAGAAGGACAGATTCTGTTCCGaatcaccgatgcggcgtcag

||||||||||||| |||||||||||||||||||| |||||||||||||||||

AGTGCTGAAGACAAGACTGTGGAGACACCTTAGAGGGACAGATTCTGTTCCGGATGCTCATGATCATTATCATACCTTTTAATTCtttttttttCCGCTCTTGCCAAAT

|||||||||||||||| |||||||||||||||||||||||||||||||||||||

CCGCTGCTCATGATCATTATTTTACCTTTTAATTCTTTTTTTTTCCGCTCTTGCCAAAT

< BCL6_1

**BJAB, S’μ-BCL6 junctions from 5’DSBs, short overhangs (Cas9 D10A, S’μ_1 + S’μ_2 + BCL6_1 + BCL6_2)**

< S’mu_1 S’mu_2 >

AGACAGGACTGTGGAGACACCTTAGAAGGACAGATTCTGTTCCGAatcaccgatgcggcgtcagcaggactggcctagcggaggctct

||||| |||||||||||||||||||| ||||||||||||||||||||||||||||

AGACAAGACTGTGGAGACACCTTAGAGGGACAGATTCTGTTCCGAATCACCGATGATACCTTTTAATTCtttttttttTCCGCTCTTGCCAAATGCTTTGGCTCCAAGTTT

||||||||||||| |||||||||||||||||||||||||||||||||||||||||

CCGCTGCTCATGATCATTATTTTACCTTTTAATTC-TTTTTTTTTCCGCTCTTGCCAAATGCTTTGGCTCCAAGTTT

< BCL6_1 BCL6_2 >

S’mu_2 >

GTTGGTGGCATAAGAGAAAACTCAATCAGATAGTGCTGAAGACAGGACTGTGGagacac...48 nt...actggcctagcggaggctct

|||||||||||||||||||||||||||||||||||||||||||| ||||||||

GTTGGTGGCATAAGAGAAAACTCAATCAGATAGTGCTGAAGACAAGACTGTGGCTCCAAGTTTTCTATGTGTATCTATTGATATAAATGTATATATTTATTTATTC

|| ||||||||||||||||||||||||||||||||||||||||||||||||||||||||

CCGCTGCTCATGATCATTAT...34 nt...CAAATGCTTTGGCTCCAAGTTTTCTATGTGTATCTATTGATATAAATGTATATATTTATTTATTC

< BCL6_1

< S’mu_1 S’mu_2 >

GACTGTGGAGACACCTTAGAAGGACAGATTCTGTTCCGAatcaccgatgcggcgtcagcaggactggcctagcggaggctct

||||||||||||||||||||||||||||||||||||||||||||||||||||||

GACTGTGGAGACACCTTAGAAGGACAGATTCTGTTCCGAATCACCGATGCGGCGACCTTTTAATTCttttttttTCCGCTCTTGCCAAATGCTTTGGCTCCAAGTTTTCT

||||||||||||||||||||||||||||||||||||||||||||||||||||||||

CCGCTGCTCATGATCATTATTTTACCTTTTAATTCTTTTTTTTTCCGCTCTTGCCAAATGCTTTGGCTCCAAGTTTTCT

< BCL6_1 BCL6_2 >

< S’mu_1 S’mu_2 >

AGACAGGACTGTGGAGACACCTTAGAAGGACAGATTCTGTTCCGAatcaccgatgcggcgtcagcaggactggcctagcggaggctct

||||||||||||||||||||||||||||||||||||||||||||| |||||||||||

AGACAGGACTGTGGAGACACCTTAGAAGGACAGATTCTGTTCCGAGTCACCGATGCGCTCTTGCCAAATGCTTTGGCTCCAAGTTTTCTATGTGTATCTATTGATATAAATG

|||||||||||||||||||||||||||||||||||||||||||||||||||||||||

CCGCTGCTCATGATCATTATTTTACCTTTTAATTCTTTTTTTTTCCGCTCTTGCCAAATGCTTTGGCTCCAAGTTTTCTATGTGTATCTATTGATATAAATG

< BCL6_1 BCL6_2 >

< S’mu_1 S’mu_2 >

GACTGTGGAGACACCTTAGAAGGACAGATTCTGTTCCGAatcaccgatgcggcgtcagcaggactggcctagcggaggctct

||||||||||||||||||||||||||||||||||||||||||||||||||

GACTGTGGAGACACCTTAGAAGGACAGATTCTGTTCCGAATCACCGATGCCAAATGCTTTGGCTCCAAGTTTTCTATGTGTATCTATTGATATAAATGTATATATTT

||||||||||||||||||||||||||||||||||||||||||||||||||||||||||||

CCGCTGCTCATGATCATTAT...25 nt...CGCTCTTGCCAAATGCTTTGGCTCCAAGTTTTCTATGTGTATCTATTGATATAAATGTATATATTT

< BCL6_1

S’mu_2 >

TTTTAGTATTTTTCAAGACCACTTTTCAACTACTCACTTTAGGATAAGTTTTaggtaa...149 nt...actggcctagcggaggctct

||||||||||||||||||||||||||||||||||||||||||||||||||||

TTTTAGTATTTTTCAAGACCACTTTTCAACTACTCACTTTAGGATAAGTTTTCTATGTGTATCTATTGATATAAATGTATATATTTATTTATTCTAGCTGTCAGG

||||||||||||||||||||||||||||||||||||||||||||||||||||||||||||

CCGCTGCTCATGATCATTAT...44 nt...GGCTCCAAGTTTTCTATGTGTATCTATTGATATAAATGTATATATTTATTTATTCTAGCTGTCAGG

< BCL6_1

< S’mu_1 S’mu_2 >

GACTGTGGAGACACCTTAGAAGGACAGATTCTGTTCCGAatcaccgatgcggcgtcagcaggactggcctagcggaggctct

||||||||||||||||||||||||||||||||||||||||||||||

GACTGTGGAGACACCTTAGAAGGACAGATTCTGTTCCGAATCACCGCTTAATTCtttttttttCCGCTCTCGCCAAATGCTTTGGCTCCAAGTTTTCTATGTGTATC

||||||||||||||||||||||| ||||||||||||||||||||||||||||||||||||

CCGCTGCTCATGATCATTATTTTACCTTTTAATTCTTTTTTTTTCCGCTCTTGCCAAATGCTTTGGCTCCAAGTTTTCTATGTGTATC

< BCL6_1 BCL6_2 >

< S’mu_1 S’mu_2 >

GACTGTGGAGACACCTTAGAAGGACAGATTCTGTTCCGAatcaccgatgcggcgtcagcaggactggcctagcggaggctct

||||||||||||||||||||||||||||||||||||||||||||||||

GACTGTGGAGACACCTTAGAAGGACAGATTCTGTTCCGAATCACCGATTTTCCGCTCTTGCCAAATGCTTTGGCTCCAAGTTTTCTATGTGTATCTATTGATATAAA

||||||||||||||||||||||||||||||||||||||||||||||||||||||||||||

CCGCTGCTCATGATCATTATTTTACCTTTTAATTCTTTTTTTTTCCGCTCTTGCCAAATGCTTTGGCTCCAAGTTTTCTATGTGTATCTATTGATATAAA

< BCL6_1 BCL6_2 >

< S’mu_1 S’mu_2 >

GACAGATTCTGTTCCGAATCACCGATGCGGCGTCAGCAGGActggcctagcggaggctct

||||||||||||||||||||||||||||||||||||||||||||||||

GACAGATTCTGTTCCGAATCACCGATGCGGCGTCAGCAGGACTGGCCTCATGAGTTTTCTATGTGTATCTATTGATATAAATGTATATATTTATTTATTCTAGCTGTCA

|||||||||||||||||||||||||||||||||||||||||||||||||||||||||

CCGCTGCTCATGATCATTAT...45 nt...GCTCCAAGTTTTCTATGTGTATCTATTGATATAAATGTATATATTTATTTATTCTAGCTGTCA

< BCL6_1

S’mu_2 >

GAAAACTCAATCAGATAGTGCTGAAGACAGGACTGTGGAGACACCTTAGAAGGAcagatt...32 nt...actggcctagcggaggctct

||||||||||||||||||||||||||||| ||||||||||||||||||||||||

GAAAACTCAATCAGATAGTGCTGAAGACAAGACTGTGGAGACACCTTAGAAGGATGCTTTGGCTCCAAGTTTTCTATGTGTATCTATTGATATAAATGTATATATTTATTTAT

||||||||||||||||||||||||||||||||||||||||||||||||||||||||||||

CCGCTGCTCATGATCATTAT...31 nt...TGCCAAATGCTTTGGCTCCAAGTTTTCTATGTGTATCTATTGATATAAATGTATATATTTATTTAT

< BCL6_1

S’mu_2 >

GTAAAATGTGCATCATTATCCTGAATTATTTCAGTTAAGCATGTTAGttggtggc...98 nt...actggcctagcggaggctct

|||||||||||||||||||||||||||||||||||||||||||||||||

GTAAAATGTGCATCATTATCCTGAATTATTTCAGTTAAGCATGTTAGTT[insert]cattattttaccttttaattctttttttttCC

||||||||||||||||||||||||||||||||

CCGCTGCTCATGATCATTATTTTACCTTTTAATTCTTTTTTTTTCC

< BCL6_1

[insert]:

TAATTCTTTTTTTTTCCGCTCTTGCCATTATTTTACCTTTACCTTTTAATTCTTTTTTTTTCCGCTCTTGCCATTATTTTACCTTTTAATTCTTTTTTTTCCGCTCTTGCCATTATTTTACCTTTACCTTTTAATTCTTTTTTTTCCGCTCTTGC

< S’mu_1 S’mu_2 >

TGGAGACACCTTAGAAGGACAGATTCTGTTCCGAatcaccgatgcggcgtcagcaggactggcctagcggaggctct

|||||||||||||||||||||||||||||||||||||||||||||||||||

TGGAGACACCTTAGAAGGACAGATTCTGTTCCGAATCACCGATGCGGCGTCGGCGTGGCGTCAGCGCTTTGGCTCCAAGTTTTCTATGTGTATCTATTGATATAAATGTATATA

|||||||||||||||||||||||||||||||||||||||||||||||||

CCGCTGCTCATGATCATTATTTTACCTTTTAATTCTTTTTTTTTCCGCTCTTGCCAAATGCTTTGGCTCCAAGTTTTCTATGTGTATCTATTGATATAAATGTATATA

< BCL6_1 BCL6_2 >

S’mu_2 >

ATGTTAGTTGGTGGCATAAGAGAAAACTCAATCAGATAGTGCTGAAGACAGGACTGTggagac...50 nt...actggcctagcggaggctct

|||||||||||||||||||||||||||||||||||||||||||||||||||| ||||

ATGTTAGTTGGTGGCATAAGAGAAAACTCAATCAGATAGTGCTGAAGACAGG-CTGTCAGGTGTTAAAATAAATGCCGAAGATTAGTCCCACGTCTCTCCCACCATA

| ||||||||||||||||||||||||||||||||||||||||||||||||||||||

CCGCTGCTCATGATCATTAT...95 nt...ATTCTAG-CTGTCAGGTGTTAAAATAAATGCCGAAGATTAGTCCCACGTCTCTCCCACCATA

< BCL6_1

< S’mu_1 S’mu_2 >

AGAAAACTCAATCAGATAGTGCTGAAGACAGGACTGTGGAGACACCTTAGAAGGAcagattctgttccgaatcaccgatgcggcgtcagcaggactggcctagcggaggctct

|||||||||||||||||||||||||||||| ||||||||||||||||||||||||

AGAAAACTCAATCAGATAGTGCTGAAGACAAGACTGTGGAGACACCTTAGAAGGATATAGATTGTTATGTATTTATTATTATTATTGTTGTCTTTGAGTGAATCGGCCGGT

||||||||||||||||||||||||||||||||||||||||||||||||||||||||||||

CCGCTGCTCATGATCATTAT...149 nt...CACCATAGGATATAGATTGTTATGTATTTATTATTATTATTGTTGTCTTTGAGTGAATCGGCCGGT

< BCL6_1

S’mu_2 >

AAGTAAGATGTTTAAGAAATTAAACAGTCTTAGGGAGAGTTTATgactgt...387 nt...actggcctagcggaggctct

||||||||||||||||||||||||||||||||||||||||||||

AAGTAAGATGTTTAAGAAATTAAACAGTCTTAGGGAGAGTTTATCCCACCATAGGATATAGATTGTTATGTATTTATTATTATTATTGTTGTCTTTGAGTGAA

||||||||||||||||||||||||||||||||||||||||||||||||||||||||||||

CCGCTGCTCATGATCATTAT...140 nt...CGTCTCTCCCACCATAGGATATAGATTGTTATGTATTTATTATTATTATTGTTGTCTTTGAGTGAA

< BCL6_1

S’mu_2 >

TAGTTGGTGGCATAAGAGAAAACTCAATCAGATAGTGCTGAAGACAGGACTGTGGAGACACCttagaa...41 nt...actggcctagcggaggctct

|||||||||||||||||||||||||||||||||||||||||||||| |||||||||||||||

TAGTTGGTGGCATAAGAGAAAACTCAATCAGATAGTGCTGAAGACAAGACTGTGGAGACACCCTCCCTTGTGTTGTTTTGGTTTTTggaaaggaggtggaggagaggaaggagg

||||||||||||||||||||||||||||||||||||||||||||||||||||||||

CCGCTGCTCATGATCATTAT...225 nt...TTTTGCCACCCTCCCTTGTGTTGTTTTGGTTTTTGGAAAGGAGGTGGAGGAGAGGAAGGAGG

< BCL6_1

< S’mu_1 S’mu_2 >

GACAGGACTGTGGAGACACCTTAGAAGGACAGATTCTGTTCCGAatcaccgatgcggcgtcagcaggactggcctagcggaggctct

||||||||||||||||||||||||||||||||||||||||||||||||||||||||

GACAGGACTGTGGAGACACCTTAGAAGGACAGATTCTGTTCCGAATCACCGATGCGCTTGCAAACTGCTTTCCTTGCTCCGTCGCTC

|||||||||||||||||||||||||||||||||

CCGCTGCTCATGATCATTAT...470 nt...TTTTCTCGCTTGCAAACTGCTTTCCTTGCTCCGTCGCTC

< BCL6_1

< S’mu_1 S’mu_2 >

AAGGACAGATTCTGTTCCGAATCACCGATGCGGCGTCAGCAGGActggcctagcggaggctct

|||||||||||||||||||||||||||||||||||||||||||||||||||||||

AAGGACAGATTCTGTTCCGAATCACCGATGCGGCGTCAGCAGGACTGGCCTAGCGCTCTTGCCAAATGCTTTGGCTCCAAGTTTTCTATGTGTATCTATTGATATAAATG

|||||||||||||||||||||||||||||||||||||||||||||||||||||||||

CCGCTGCTCATGATCATTATTTTACCTTTTAATTCTTTTTTTTTCCGCTCTTGCCAAATGCTTTGGCTCCAAGTTTTCTATGTGTATCTATTGATATAAATG

< BCL6_1 BCL6_2 >

< S’mu_1 S’mu_2 >

AGGACTGTGGAGACACCTTAGAAGGACAGATTCTGTTCCGAatcaccgatgcggcgtcagcaggactggcctagcggaggctct

||||||||||||||||||||||||||||||||||||||||||||||||||||

AGGACTGTGGAGACACCTTAGAAGGACAGATTCTGTTCCGAATCACCGATGCTACCTTTTAATTCATTTTACCTTTTAATTCtttttttttCCGCTCTTGCCAAAT

||| |||||||||||||||||||||||||||||||||||||||||

CCGCTGCTCATGATCATT-ATTTTACCTTTTAATTCTTTTTTTTTCCGCTCTTGCCAAAT

< BCL6_1 BCL6_2 >

< S’mu_1 S’mu_2 >

TAGTGCTGAAGACAGGACTGTGGAGACACCTTAGAAGGACAGATTCTGTTCCGAatcaccgatgcggcgtcagcaggactggcctagcggaggctct

||||||||||||||||||||||||||||||||||||||||||||||||||| |||||||

TAGTGCTGAAGACAGGACTGTGGAGACACCTTAGAAGGACAGATTCTGTTCAGAATCACTTTGGCTCCAAGTTTTCTATGTGTATCTATTGATATAAATGTATATATTTATTTA

||||||||||||||||||||||||||||||||||||||||||||||||||||||||

CCGCTGCTCATGATCATTAT...34 nt...CAAATGCTTTGGCTCCAAGTTTTCTATGTGTATCTATTGATATAAATGTATATATTTATTTA

< BCL6_1 BCL6_2 >

< S’mu_1 S’mu_2 >

GGACAGATTCTGTTCCGAATCACCGATGCGGCGTCAGCAGGActggcctagcggaggctct

||||||||||||||||||||||||||||||||||||||||||||||

GGACAGATTCTGTTCCGAATCACCGATGCGGCGTCAGCAGGACTGGcttttaccttttaattctttttaccttttaattctttttttttCCGCTCTTGCCAAAT

||||||||||||||||||||||||||||||||||||||||

CCGCTGCTCATGATCATTATTTTACCTTTTAATTCTTTTTTTTTCCGCTCTTGCCAAAT

< BCL6_1 BCL6_2 >

< S’mu_1 S’mu_2 >

TCAATCAGATAGTGCTGAAGACAGGACTGTGGAGACACCTTAGAAGGACAGATTCTGTTccgaatcaccgatgcggcgtcagcaggactggcctagcggaggctct

||||||||||||||||||||||| |||||||||||||||||||||||||||||||||||

TCAATCAGATAGTGCTGAAGACAAGACTGTGGAGACACCTTAGAAGGACAGATTCTGTTGTTTTGGTTTTTggaaaggaggtggaggagaggaaggaggggaattaggg

|| ||||||||||||||||||||||||||||||||||||||||||||||||||||||

CCGCTGCTCATGATCATTAT...234 nt...CCTCCCTTGTGTTGTTTTGGTTTTTGGAAAGGAGGTGGAGGAGAGGAAGGAGGGGAATTAGGG

< BCL6_1

< S’mu_1 S’mu_2 >

ATAGTGCTGAAGACAGGACTGTGGAGACACCTTAGAAGGACAGATTCTGTTCCGaatcaccgatgcggcgtcagcaggactggcctagcggaggctct

||||||||||||||| |||||||||||||||||||||||||||||||||| |||

ATAGTGCTGAAGACAAGACTGTGGAGACACCTTAGAAGGACAGATTCTGT-CCGCTCTTGCCAAATGCTTTGGCTCCAAGTTTTCTATGTGTATCTATTGATATAAATGT

| |||||||||||||||||||||||||||||||||||||||||||||||||||||||||||

CCGCTGCTCATGATCATTATTTTACCTTTTAATTCTTTTTTTTT-CCGCTCTTGCCAAATGCTTTGGCTCCAAGTTTTCTATGTGTATCTATTGATATAAATGT

< BCL6_1 BCL6_2 >

S’mu_2 >

TCACAATCTTTTTAGGTTAACTCGTTTTCTCTTTGTGATTAAGGAGAAACACTttgata...227 nt...actggcctagcggaggctct

|||||||||||||||||||||||||||||||||||||||||||||||||||||

TCACAATCTTTTTAGGTTAACTCGTTTTCTCTTTGTGATTAAGGAGAAACACTCCCACGTCTCTCCCACCATAGGATATAGATTGTTATGTATTTATTATTAT

|||||||||||||||||||||||||||||||||||||||||||||||||||

CCGCTGCTCATGATCATTAT...129 nt...GATTAGTCCCACGTCTCTCCCACCATAGGATATAGATTGTTATGTATTTATTATTAT

< BCL6_1

< S’mu_1 S’mu_2 >

AGAAGGACAGATTCTGTTCCGAATCACCGATGCGGCGTCAGCAGGActggcctagcggaggctct

|||||||||||||||||||||||||||||||||||||||||||||||||||||

AGAAGGACAGATTCTGTTCCGAATCACCGATGCGGCGTCAGCAGGACTGGCCTCCTATTTTACCTTTTAATTCttttttttTCCGCTCTTGCCAAATGCTTTGGC

||||||||||||||||||||||||||||||||||||||||||||||||||

CCGCTGCTCATGATCATTATTTTACCTTTTAATTCTTTTTTTTTCCGCTCTTGCCAAATGCTTTGGC

< BCL6_1 BCL6_2 >

< S’mu_1 S’mu_2 >

GTGGAGACACCTTAGAAGGACAGATTCTGTTCCGAATCACCGATGCGGCGTCAGCAGGActggcctagcggaggctct

||||||||||||||||||||||||||||||||||||||||||||||||||||||| |||

GTGGAGACACCTTAGAAGGACAGATTCTGTTCCGAATCACCGATGCGGCGTCAGCGGGATATAGATTGTTATGTATTTATTATTATTATTGTTGTCTTTGAGTGAATCGGCC

||||||||||||||||||||||||||||||||||||||||||||||||||||||||

CCGCTGCTCATGATCATTAT...150 nt...ACCATAGGATATAGATTGTTATGTATTTATTATTATTATTGTTGTCTTTGAGTGAATCGGCC

< BCL6_1

< S’mu_1 S’mu_2 >

TAGAAGGACAGATTCTGTTCCGAATCACCGATGCGGCGTCAGCAGGActggcctagcggaggctct

||||||||||||||||||||||||||||||||||||||||||||||||||||||

TAGAAGGACAGATTCTGTTCCGAATCACCGATGCGGCGTCAGCAGGACTGGCCTCTTTGGCTCCAAGTTTTCTATGTGTATCTATTGATATAAATGTATATATTTATTTA

||||||||||||||||||||||||||||||||||||||||||||||||||||||||

CCGCTGCTCATGATCATTAT...34 nt...CAAATGCTTTGGCTCCAAGTTTTCTATGTGTATCTATTGATATAAATGTATATATTTATTTA

< BCL6_1 BCL6_2 >

S’mu_2 >

ATCATTATCCTGAATTATTTCAGTTAAGCATGTTAGTTGGTGGCATAagagaa...89 nt...actggcctagcggaggctct

|||||||||||||||||||||||||||||||||||||||||||||||

ATCATTATCCTGAATTATTTCAGTTAAGCATGTTAGTTGGTGGCATACCACTCCAAGTTTTCTATGTGTATCTATTGATATAAATGTATATATTTATTTATTC

|||||||||||||||||||||||||||||||||||||||||||||||||||||

CCGCTGCTCATGATCATTAT...40 nt...CTTTGGCTCCAAGTTTTCTATGTGTATCTATTGATATAAATGTATATATTTATTTATTC

< BCL6_1 BCL6_2 >

S’mu_2 >

GTTGGTGGCATAAGAGAAAACTCAATCAGATAGTGCTGAAGACAGGACTGTGGagacac...48 nt...actggcctagcggaggctct

|||||||||||||||||||||||||||||||||||||||||||| ||||||||

GTTGGTGGCATAAGAGAAAACTCAATCAGATAGTGCTGAAGACAAGACTGTGGCTCCAAGTTTTCTATGTGTATCTATTGATATAAATGTATATATTTATTTATT

|| |||||||||||||||||||||||||||||||||||||||||||||||||||||||

CCGCTGCTCATGATCATTAT...34 nt...CAAATGCTTTGGCTCCAAGTTTTCTATGTGTATCTATTGATATAAATGTATATATTTATTTATT

< BCL6_1 BCL6_2 >

< S’mu_1 S’mu_2 >

AGTGCTGAAGACAGGACTGTGGAGACACCTTAGAAGGACAGATTCTGTTCCGaatcaccgatgcggcgtcagcaggactggcctagcggaggctct

||||||||||||| |||||||||||||||||||||||||||||||||| |||

AGTGCTGAAGACAAGACTGTGGAGACACCTTAGAAGGACAGATTCTGT-CCGCTCTTGCCAAATGCTTTGGCTCCAAGTTTTCTATGTGTATCTATTGATATAAA

| ||||||||||||||||||||||||||||||||||||||||||||||||||||||||

CCGCTGCTCATGATCATTATTTTACCTTTTAATTCTTTTTTTTT-CCGCTCTTGCCAAATGCTTTGGCTCCAAGTTTTCTATGTGTATCTATTGATATAAA

< BCL6_1 BCL6_2 >

S’mu_2 >

CAACTACTCACTTTAGGATAAGTTTTAGGTAAAATGTGCATCATTATCCTGAattatt...123 nt...actggcctagcggaggctct

||||||||||||||||||||||||||||||||||||||||||||||||||||

CAACTACTCACTTTAGGATAAGTTTTAGGTAAAATGTGCATCATTATCCTGAGCTTTGGCTCCAAGTTTTCTATGTGTATCTATTGATATAAATGTATATATTTATTTAT

||||||||||||||||||||||||||||||||||||||||||||||||||||||||||

CCGCTGCTCATGATCATTAT...33 nt...CCAAATGCTTTGGCTCCAAGTTTTCTATGTGTATCTATTGATATAAATGTATATATTTATTTAT

< BCL6_1 BCL6_2 >

S’mu_2 >

GTTGGTGGCATAAGAGAAAACTCAATCAGATAGTGCTGAAGACAGGACTGTGGagacac...48 nt...actggcctagcggaggctct

|||||||||||||||||||||||||||||||||||||||||||| ||||||||

GTTGGTGGCATAAGAGAAAACTCAATCAGATAGTGCTGAAGACAAGACTGTGGCTCCAAGTTTTCTATGTGTATCTATTGATATAAATGTATATATTTATTTA

|| |||||||||||||||||||||||||||||||||||||||||||||||||||||

CCGCTGCTCATGATCATTAT...34 nt...CAAATGCTTTGGCTCCAAGTTTTCTATGTGTATCTATTGATATAAATGTATATATTTATTTA

< BCL6_1 BCL6_2 >

< S’mu_1 S’mu_2 >

AGGACAGATTCTGTTCCGAATCACCGATGCGGCGTCAGCAGGActggcctagcggaggctct

|||||||||||||||||||||||||||||||||||||||||||||||||||||||

AGGACAGATTCTGTTCCGAATCACCGATGCGGCGTCAGCAGGACTGGCCTAGCGGGCCAAATGCTTTGGCTCCAAGTTCTCTATGTGTATCTATTGATATAAATGTATAT

||||||||||||||||||||||| |||||||||||||||||||||||||||||||

CCGCTGCTCATGATCATTATTTTACCTTTTAATTCTTTTTTTTTCCGCTCTTGCCAAATGCTTTGGCTCCAAGTTTTCTATGTGTATCTATTGATATAAATGTATAT

< BCL6_1 BCL6_2 >

< S’mu_1 S’mu_2 >

CAATCAGATAGTGCTGAAGACAGGACTGTGGAGACACCTTAGAAGGACAGATTCTgttccgaatcaccgatgcggcgtcagcaggactggcctagcggaggctct

|||||||||||||||||||||||||||||||||||||||||||||||||||||||

CAATCAGATAGTGCTGAAGACAGGACTGTGGAGACACCTTAGAAGGACAGATTCTATTGATATAAATGTATATATTTATTTATTCTAGCTGTCAGGTGTTAAAATAAATGC

|||||||||||||||||||||||||||||||||||||||||||||||||||||||||||

CCGCTGCTCATGATCATTAT...60 nt...TGTGTATCTATTGATATAAATGTATATATTTATTTATTCTAGCTGTCAGGTGTTAAAATAAATGC

< BCL6_1

< S’mu_1 S’mu_2 >

GTGCTGAAGACAGGACTGTGGAGACACCTTAGAAGGACAGATTCTGTTCCGAatcaccgatgcggcgtcagcaggactggcctagcggaggctct

|||||||||||| |||||||||||||||||||||||||||||||||||||||||||||

GTGCTGAAGACAAGACTGTGGAGACACCTTAGAAGGACAGATTCTGTTCCGAATCACCCACGTCTCTCCCACCATAGGATATAGATTGTTATGTATTTATTATTATTATTG

|||||||||||||||||||||||||||||||||||||||||||||||||||||||

CCGCTGCTCATGATCATTAT...130 nt...ATTAGTCCCACGTCTCTCCCACCATAGGATATAGATTGTTATGTATTTATTATTATTATTG

< BCL6_1

**BJAB, S’μ-BCL6 junctions from 5’DSBs, long overhangs (Cas9 D10A, S’μ_1 + S’μ_2 + BCL6_1 + BCL6_3)**

< S’mu_1 S’mu_2 >

CAATCAGATAGTGCTGAAGACAGGACTGTGGAGACACCTTAGAAGGACAGATTCTGttccgaatcaccgatgcggcgtcagcaggactggcctagcggaggctct

|||||||||||||||||||||| |||||||||||||||||||||||||||||||||

CAATCAGATAGTGCTGAAGACAAGACTGTGGAGACACCTTAGAAGGACAGATTCTGCATAGGATATAGATTGttatgtatttattattattattattGTCTTTGAGTGAATCGG

|||||||||||||||||||||||||||||||||||||| |||||||||||||||||||

CCGCTGCTCATGATCATTAT...135 nt...TCCCACGTCTCTCCCACCATAGGATATAGATTGTTATGTATTTATTATTATTATTGTTGTCTTTGAGTGAATCGG

< BCL6_1 BCL6_3 >

< BCL6_1

CCGCTGCTCATGATCATTATTTT(sequence continues below)

GACTGTGGAGACACCTTAGaaggac...37 nt...S’mu_2 ACCTTTTAATTCTTTTTTTTTCCGCTCTTGCCAAATGCTTTGGCTCCAAG

||||||||||||||||||| |||||||||||| |||||||||||||||||||||||||||||||||||||

GACTGTGGAGACACCTTAGTATTTTTCAAGACC...GACTGTGGAGACACCTTTTAATTC-ttttttttCCGCTCTTGCCAAATGCTTTGGCTCCAAG

||||||||||||||||||...|||||||||||||||||

TTAGTATTTTTCAAGACC...GACTGTGGAGACACCTTagaagg...39 nt...actggcctagcggaggctct

S’mu_2 >

S’mu_2 >

GAAAACTCAATCAGATAGTGCTGAAGACAGGACTGTGGAGACACCTtagaag...40 nt...actggcctagcggaggctct

||||||||||||||||||||||||||||| ||||||||||||||||

GAAAACTCAATCAGATAGTGCTGAAGACAAGACTGTGGAGACACCTATGTATTTATTATTATTATTGTTGTCTTTGAGTGAATCGGCCGGTTTGGGGAGGCT

|||||||||||||||||||||||||||||||||||||||||||||||||||||||||

CCGCTGCTCATGATCATTAT...163 nt...GATTGTTATGTATTTATTATTATTATTGTTGTCTTTGAGTGAATCGGCCGGTTTGGGGAGGCT

< BCL6_1

< S’mu_1 S’mu_2 >

CTGTGGAGACACCTTAGAAGGACAGATTCTGTTCCGAatcaccgatgcggcgtcagcaggactggcctagcggaggctct

||||||||||||||||||||||||||||||||||||||||||||||||||||||

CTGTGGAGACACCTTAGAAGGACAGATTCTGTTCCGAATCACCGATGCGGCGTCTTGGAGTGAATCGGCCGGTTTGGGGAGGCTTTTGCCACCCTCCCTTGTGTTGTTTTG

||||| ||||||||||||||||||||||||||||||||||||||||||||||||||||||

CCGCTGCTCATGATCATTAT...187 nt...ATTGTTGTCTTTGAGTGAATCGGCCGGTTTGGGGAGGCTTTTGCCACCCTCCCTTGTGTTGTTTTG

< BCL6_1

S’mu_2 >

GTTGGTGGCATAAGAGAAAACTCAATCAGATAGTGCTGAAGACAggactg...57 nt...actggcctagcggaggctct

||||||||||||||||||||||||||||||||||||||||||||

GTTGGTGGCATAAGAGAAAACTCAATCAGATAGTGCTGAAGACA[insert]ttttGCCACCCTCCCTTGTGTTGTTTTGGTTTTTggaaaggaggtggaggagagga

||||||||||||||||||||||||||||||||||||||||||||||||||||||||

CCGCTGCTCATGATCATTAT...219 nt...GGAGGCTTTTGCCACCCTCCCTTGTGTTGTTTTGGTTTTTGGAAAGGAGGTGGAGGAGAGGA

< BCL6_1

[insert]: AATAGTAGTAGACACATCACATGGCCAGAGCAGGGGCCACAGGAAGAGAGGCTTTT

< S’mu_1 S’mu_2 >

GACTGTGGAGACACCTTAGAAGGACAGATTCTGTTCCGAatcaccgatgcggcgtcagcaggactggcctagcggaggctct

||||||||||||||||||||||||||||||||||||||| ||||||||||

GACTGTGGAGACACCTTAGAAGGACAGATTCTGTTCCGAGTCACCGATGCACTAGGCCATGTTGTCTTTGAGTGAATCGGCCGGTTTGGGGAGGCTTTTGCCACCCTC

|||||||||||||||||||||||||||||||||||||||||||||||||

CCGCTGCTCATGATCATTAT...183 nt...TATTATTGTTGTCTTTGAGTGAATCGGCCGGTTTGGGGAGGCTTTTGCCACCCTC

< BCL6_1

< S’mu_1 S’mu_2 >

TGAAGACAGGACTGTGGAGACACCTTAGAAGGACAGATTCTGTTCCGAatcaccgatgcggcgtcagcaggactggcctagcggaggctct

|||||||| ||||| |||||||||||||||||||||||||||||||||||

TGAAGACAAGACTGCGGAGACACCTTAGAAGGACAGATTCTGTTCCGAATGTATTTATTATTATTATTGTTGTCTTTGAGTGAATCGGCCGGTTTGGGGAGGCTTT

||||||||||||||||||||||||||||||||||||||||||||||||||||||||||

CCGCTGCTCATGATCATTAT...164 nt...ATTGTTATGTATTTATTATTATTATTGTTGTCTTTGAGTGAATCGGCCGGTTTGGGGAGGCTTT

< BCL6_1

S’mu_2 >

CATTATTTTCATAATGACTGTGTTCACAATCTTTTTAGGTTAACTCGTTTTCTCTTTGTGattaag...243 nt...actggcctagcggaggctct

||||||||||||||||||||||||||||||||||||||||||||||||||||||| ||||

CATTATTTTCATAATGACTGTGTTCACAATCTTTTTAGGTTAACTCGTTTTCTCTATGTGTATCTATTGATATAAATGTATATATTTATTTATTCTAGCTGT

||| ||||||||||||||||||||||||||||||||||||||||||||||||||

CCGCTGCTCATGATCATTAT...47 nt...TCCAAGTTT-TCTATGTGTATCTATTGATATAAATGTATATATTTATTTATTCTAGCTGT

< BCL6_1

< S’mu_1 S’mu_2 >

GACTGTGGAGACACCTTAGAAGGACAGATTCTGTTCCGAatcaccgatgcggcgtcagcaggactggcctagcggaggctct

|||||||||||||||||||||||||||||||||||||||||||||||||||||||||

GACTGTGGAGACACCTTAGAAGGACAGATTCTGTTCCGAATCACCGATGCGGCGTCACGTCTCTCCCACCATAGGATATAGATTGTTATGTATTTATTATTATTATTGTTGT

|||||||||||||||||||||||||||||||||||||||||||||||||||||||||

CCGCTGCTCATGATCATTAT...132 nt...TAGTCCCACGTCTCTCCCACCATAGGATATAGATTGTTATGTATTTATTATTATTATTGTTGT

< BCL6_1 BCL6_3 >

< S’mu_1 S’mu_2 >

GAAGACAGGACTGTGGAGACACCTTAGAAGGACAGATTCTGTtccgaatcaccgatgcggcgtcagcaggactggcctagcggaggctct

||||||| ||||||||||||||||||||||||||||||||||

GAAGACAAGACTGTGGAGACACCTTAGAAGGACAGATTCTGTATAGATTGTTACGTAT---TTATTATTATTGTTGTCTTTGAGTGAATCGGCCGGT

|||||||||||| |||| ||||||||||||||||||||||||||||||||||||

CCGCTGCTCATGATCATTAT...153 nt...ATAGGATATAGATTGTTATGTATTTATTATTATTATTGTTGTCTTTGAGTGAATCGGCCGGT

< BCL6_1

< S’mu_1 S’mu_2 >

GACACCTTAGAAGGACAGATTCTGTTCCGAATCACCGATGCGGCGTCAGCAGGActggcctagcggaggctct

|||||||||||||||||||||||||||||||||||||||||||||||||||||||||

GACACCTTAGAAGGACAGATTCTGTTCCGAATCACCGATGCGGCGTCAGCAGGACTGTTTACCTTTTAATTCtttttttttCCGCTCTTGCCAAATGCTTTGGCTCCAAGTTTT

|||||||||||||||||||||||||||||||||||||||||||||||||||||||||

CCGCTGCTCATGATCATTATTTTACCTTTTAATTCTTTTTTTTTCCGCTCTTGCCAAATGCTTTGGCTCCAAGTTTT

< BCL6_1

< S’mu_1 S’mu_2 >

GACTGTGGAGACACCTTAGAAGGACAGATTCTGTTCCGAatcaccgatgcggcgtcagcaggactggcctagcggaggctct

|||||||||||||||||||||||||||||||||||||||||||||||||

GACTGTGGAGACACCTTAGAAGGACAGATTCTGTTCCGAATCACCGATGTATTTATTATTATTATTGTTGTCTTTGAGTGAATCGGCCGGTTTGGGGAGGCTT

|||||||||||||||||||||||||||||||||||||||||||||||||||||||||

CCGCTGCTCATGATCATTAT...164 nt...ATTGTTATGTATTTATTATTATTATTGTTGTCTTTGAGTGAATCGGCCGGTTTGGGGAGGCTT

< BCL6_1

S’mu_2 >

TTTCATAATGACTGTGTTCACAATCTTTTTAGGTTAACTCGTTTTCTCTTTGTgattaa...244 nt...actggcctagcggaggctct

|||||||||||||||||||||||||||||||||||||||||||||||||||||

TTTCATAATGACTGTGTTCACAATCTTTTTAGGTTAACTCGTTTTCTCTTTGTTTTGGTTTTTggaaaggaggtggaggagaggaaggaggggaattagggggcggcc

|||||||||||||||||||||||||||||||||||||||||||||||||||||||||||

CCGCTGCTCATGATCATTAT...239 nt...CTTGTGTTGTTTTGGTTTTTGGAAAGGAGGTGGAGGAGAGGAAGGAGGGGAATTAGGGGGCGGCC

< BCL6_1

< S’mu_1 S’mu_2 >

GACTGTGGAGACACCTTAGAAGGACAGATTCTGTTCCGAatcaccgatgcggcgtcagcaggactggcctagcggaggctct

|||||||||||||||||||||||||||||||||||||||||||||||||

GACTGTGGAGACACCTTAGAAGGACAGATTCTGTTCCGAATCACCGATGTTATTATTATTGTTGTCTTTGAGTGAATCGGCCGGTTTGGGGAGGCTTTTGCCACCC

|||||||||||||||||||||||||||||||||||||||||||||||||||||||||

CCGCTGCTCATGATCATTAT...173 nt...TATTTATTATTATTATTGTTGTCTTTGAGTGAATCGGCCGGTTTGGGGAGGCTTTTGCCACCC

< BCL6_1

S’mu_2 >

AGTTTTAGGTAAAATGTGCATCATTATCCTGAATTATTTCAGTTA-AGCATGTTAgttggt...101 nt...actggcctagcggaggctct

|||||||||||||||||||||||||||||||||||||||||| || || |||||

AGTTTTAGGTAAAATGTGCATCATTATCCTGAATTATTTCAGATATAGATTGTTATGTATTTATTATTATTATTGTTGTCTTTGAGTGAATCGGCC

|||||||||||||||||||||||||||||||||||||||||||||||||||||||

CCGCTGCTCATGATCATTAT...151 nt...CCATAGGATATAGATTGTTATGTATTTATTATTATTATTGTTGTCTTTGAGTGAATCGGCC

< BCL6_1

< S’mu_1 S’mu_2 >

GACTGTGGAGACACCTTAGAAGGACAGATTCTGTTCCGAatcaccgatgcggcgtcagcaggactggcctagcggaggctct

|||||||||||||||||||||||||||||||||||||||||||||||

GACTGTGGAGACACCTTAGAAGGACAGATTCTGTTCCGAATCACCGAGTGAATCGGCCGGTTTGGGGAGGCTTCTGCCACCCTCCCTTGTGTTGTTTTGGTTT

|||||||||||||||||||||||||||| |||||||||||||||||||||||||||||

CCGCTGCTCATGATCATTAT...193 nt...GTCTTTGAGTGAATCGGCCGGTTTGGGGAGGCTTTTGCCACCCTCCCTTGTGTTGTTTTGGTTT

< BCL6_1

< S’mu_1 S’mu_2 >

AGGACTGTGGAGACACCTTAGAAGGACAGATTCTGTTCCGAatcaccgatgcggcgtcagcaggactggcctagcggaggctct

| ||||||||||||||||||||||||||||||||||||||||||

AAGACTGTGGAGACACCTTAGAAGGACAGATTCTGTTCCGAATCCCCACCATAGGATATAGATTGTTATGTATTTATTATTATTATTGTTGTCTTTGAGTGAAT

||||||||||||||||||||||||||||||||||||||||||||||||||||||||||||

CCGCTGCTCATGATCATTAT...135 nt...TCCCACGTCTCTCCCACCATAGGATATAGATTGTTATGTATTTATTATTATTATTGTTGTCTTTGAGTGAAT

< BCL6_1 BCL6_3 >

S’mu_2 >

GTAAAATGTGCATCATTATCCTGAATTATTTCAGTTAAGCATGTTAGTTGGTggcata...95 nt...actggcctagcggaggctct

||||||||||||||||||||||||||||||||||||||||| ||||||||||

GTAAAATGTGCATCATTATCCTGAATTATTTCAGTTAAGCACGTTAGTTGGTTAGATTGTTATGTATTTATTATTATTATTGTTGTCTTTGAGTGAATCGGCCGGTTTGGGG

||||||||||||||||||||||||||||||||||||||||||||||||||||||||||||

CCGCTGCTCATGATCATTAT...155 nt...AGGATATAGATTGTTATGTATTTATTATTATTATTGTTGTCTTTGAGTGAATCGGCCGGTTTGGGG

< BCL6_1

< S’mu_1 S’mu_2 >

TCTGTTCCGAATCACCGATGCGGCGTCAGCAGGACTGGCCTAGCGGAGGCTCT

||||||||||| ||||||||||||||||||||

TCTGTTCCGAACCACCGATGCGGCGTCAGCAGCGGCGTCAGCATAGGACTGAGGATATAGATTGTTATGTATTTATTATTATTATTGTT

||||||||||||||||||||||||||||||||||||||

CCGCTGCTCATGATCATTAT...149 nt...CACCATAGGATATAGATTGTTATGTATTTATTATTATTATTGTT

< BCL6_1 BCL6_3 >

< S’mu_1 S’mu_2 >

CAGGACTGTGGAGACACCTTAGAAGGACAGATTCTGTTCCGAATCACCGATGCGGCGTCAGCAGGACTGGCCTAGCGGAGGCTCT

||||||||||||||||||||||||||||||||||||||||||||||||||||||||||

CAGGACTGTGGAGACACCTTAGAAGGACAGATTCTGTTCCGAATCACCGATGCGGCGTTGTTATGTATTTATTATTATTATTGTTGTCTTTGAGTGAATCGGCCGGTTTGGGG

||||||||||||||||||||||||||||||||||||||||||||||||||||||||

CCGCTGCTCATGATCATTAT...159 nt...TATAGATTGTTATGTATTTATTATTATTATTGTTGTCTTTGAGTGAATCGGCCGGTTTGGGG

< BCL6_1

< S’mu_1 S’mu_2 >

CAGATTCTGTTCCGAATCACCGATGCGGCGTCAGCAGGACTGGCCTAGCGGAGGCTCT

||||||||||||||||||||||||||||||||||||||||||||||

CAGATTCTGTTCCGAATCACCGATGCGGCGTCAGCAGGACTGGCCTCCA-CATAGGATATAGATTGTTATGTATTTATTATTATTATTGTTGTCTTTGAGTGAATC

||| ||||||||||||||||||||||||||||||||||||||||||||||||||||||||

CCGCTGCTCATGATCATTAT...135 nt...TCCCACGTCTCTCCCACCATAGGATATAGATTGTTATGTATTTATTATTATTATTGTTGTCTTTGAGTGAATC

< BCL6_1 BCL6_3 >

< S’mu_1 S’mu_2 >

GACTGTGGAGACACCTTAGAAGGACAGATTCTGTTCCGAATCACCGATGCGGCGTCAGCAGGACTGGCCTAGCGGAGGCTCT

||||||||||||||||||||||||||||||| |||||||||||||||||

GACTGTGGAGACACCTTAGAAGGACAGATTCCGTTCCGAATCACCGATGTACGTCTCTCCCACCATAGGATATAGATTGTTATGTATTTATTATTATTATTGTTGTCTTT

||||||||||||||||||||||||||||||||||||||||||||||||||||||||||||

CCGCTGCTCATGATCATTAT...133 nt...AGTCCCACGTCTCTCCCACCATAGGATATAGATTGTTATGTATTTATTATTATTATTGTTGTCTTT

< BCL6_1 BCL6_3 >

< S’mu_1 S’mu_2 >

AGACAGGACTGTGGAGACACCTTAGAAGGACAGATTCTGTTCCGAATCACCGATGCGGCGTCAGCAGGACTGGCCTAGCGGAGGCTCT

||||||||||||||||||||||||||||||||||||||||||||||||||||||||

AGACAGGACTGTGGAGACACCTTAGAAGGACAGATTCTGTTCCGAATCACCGATGCCATAGGATATAGATTGTTATGTATTTATTATTATTATTGTTGTCTTTGAGTGAATCGG

|||||||||||||||||||||||||||||||||||||||||||||||||||||||||||

CCGCTGCTCATGATCATTAT...135 nt...TCCCACGTCTCTCCCACCATAGGATATAGATTGTTATGTATTTATTATTATTATTGTTGTCTTTGAGTGAATCGG

< BCL6_1 BCL6_3 >

< S’mu_1 S’mu_2 >

GAGACACCTTAGAAGGACAGATTCTGTTCCGAATCACCGATGCGGCGTCAGCAGGACTGGCCTAGCGGAGGCTCT

|||||||||||||||||||||||||||||||||||||||||||||||||||||

GAGACACCTTAGAAGGACAGATTCTGTTCCGAATCACCGATGCGGCGTCAGCACAGGCTCTCCCACCATAGGATATAGATTGTTATGTATTTATTATTATTATTGTTGTC

|||||||||||||||||||||||||||||||||||||||||||||||||||||

CCGCTGCTCATGATCATTAT...135 nt...TCCCACGTCTCTCCCACCATAGGATATAGATTGTTATGTATTTATTATTATTATTGTTGTC

< BCL6_1 BCL6_3 >

< S’mu_1 S’mu_2 >

GAGACACCTTAGAAGGACAGATTCTGTTCCGAATCACCGATGCGGCGTCAGCAGGACTGGCCTAGCGGAGGCTCT

|||||||||||||||||||||||||||||||||||||||||||||||

GAGACACCTTAGAAGGACAGATTCTGTTCCGAATCACCGATGCGGCGCTCTTGCCAAATGCTTTGGCTCCAAGCTTTCTATGTGTATCTATTGATATAAATGTAT

|||||||||||||||||||||||||||| ||||||||||||||||||||||||||||||| CCGCTGCTCATGATCATTATTTTACCTTTTAATTCTTTTTTTTTCCGCTCTTGCCAAATGCTTTGGCTCCAAGTTTTCTATGTGTATCTATTGATATAAATGTAT

< BCL6_1

**BJAB, S’μ-BCL6 junctions from 3’DSBs, short overhangs (Cas9 N863A, S’μ_1 + S’μ_2 + BCL6_1 + BCL6_2)**

< S’mu_1 S’mu_2 >

CTTAGAAGGACAGATTCTGTTCCGAATCACCGATGCGGCGTCAGCAGGACTGGCCTAGCGGAGGCTCT

|||||||||||||||||||||||||||||||||||||||||||||||||||||||||||

CTTAGAAGGACAGATTCTGTTCCGAATCACCGATGCGGCGTCAGCAGGACTGGCCTAGCTTCTTTTTTTTTCCGCTCTTGCCAAATGCTTTGGCTCCAAGTTTTCTATGTGTA

||||||||||||||||||||||||||||||||||||||||||||||||||||||

CCGCTGCTCATGATCATTATTTTACCTTTTAATTCTTTTTTTTTCCGCTCTTGCCAAATGCTTTGGCTCCAAGTTTTCTATGTGTA

< BCL6_1 BCL6_2 >

< S’mu_1 S’mu_2 >

GATTCTGTTCCGAATCACCGATGCGGCGTCAGCAGGActggcctagcggaggctct

||||||||||||||||||||||||||||||||||||||||||||

GATTCTGTTCCGAATCACCGATGCGGCGTCAGCAGGACTGGCCTTACCTTTTAATTCttttttttTCCGCTCTTGCCAAATGCTTTGGCTCCAAGTTTTCTAT

||||||||||||||||||||||||||||||||||||||||||||||||||||||||||||

CCGCTGCTCATGATCATTATTTTACCTTTTAATTCTTTTTTTTTCCGCTCTTGCCAAATGCTTTGGCTCCAAGTTTTCTAT

< BCL6_1 BCL6_2 >

< S’mu_1 S’mu_2 >

AGAAGGACAGATTCTGTTCCGAATCACCGATGCGGCGTCAGCAGGActggcctagcggaggctct

|||||||||||||||||||||||||||||||||||||||||||||||||||||

AGAAGGACAGATTCTGTTCCGAATCACCGATGCGGCGTCAGCAGGACTGGCCTATTATTTTACCTTTCAATTCttttttttTCCGCTCTTGCCAAATGCTTTGGCTCCAAG

|||||||||||||| |||||||||||||||||||||||||||||||||||||||||||

CCGCTGCTCATGATCATTATTTTACCTTTTAATTCttttttttTCCGCTCTTGCCAAATGCTTTGGCTCCAAG

< BCL6_1 BCL6_2 >

< S’mu_1 S’mu_2 >

TTAGAAGGACAGATTCTGTTCCGAATCACCGATGCGGCGTCAGCAGGActggcctagcggaggctct

||||||||||||||||||||||||||||||||||||||||||||||||||||||||

TTAGAAGGACAGATTCTGTTCCGAATCACCGATGCGGCGTCAGCAGGACTGGCCTACCTTTTAATTCttttttttTCCGCTCTTGCCAAATGCTTTGGCTCCAAGTTTTCTA

||||||||||||||||||||||||||||||||||||||||||||||||||||||||||

CCGCTGCTCATGATCATTATTTTACCTTTTAATTCTTTTTTTTTCCGCTCTTGCCAAATGCTTTGGCTCCAAGTTTTCTA

< BCL6_1 BCL6_2 >

< S’mu_1 S’mu_2 >

GACACCTTAGAAGGACAGATTCTGTTCCGAATCACCGATGCGGCGTCAGCAGGActggcctagcggaggctct

||||||||||||||||||||||||||||||||||||||||||||||| ||||||||

GACACCTTAGAAGGACAGATTCTGTTCCGAATCACCGATGCGGCGTCGGCAGGACttttttTCCGCTCTTGCCAAATGCTTTGGCTCCAAGTTTTCTATGTGTATCTATTGATA

|||||||||||||||||||||||||||||||||||||||||||||||||||||||||||

CCGCTGCTCATGATCATTATTTTACCTTTTAATTCTTTTTTTTTCCGCTCTTGCCAAATGCTTTGGCTCCAAGTTTTCTATGTGTATCTATTGATA

< BCL6_1 BCL6_2 >

< S’mu_1 S’mu_2 >

AGTGCTGAAGACAGGACTGTGGAGACACCTTAGAAGGACAGATTCTGTTCCGAatcaccgatgcggcgtcagcaggactggcctagcggaggctct

||||| ||||||| |||||||||||||||||||||||||||||||||||||||

AGTGCCGAAGACAAGACTGTGGAGACACCTTAGAAGGACAGATTCTGTTCCGATATTTTACCTTTTAATTCttttttttTCCGCTCTTGCCAAATGCTTTGGCTCCAAGT

|||||||||||||||||||||||||||||||||||||||||||||||||||||||||

CCGCTGCTCATGATCATTATTTTACCTTTTAATTCTTTTTTTTTCCGCTCTTGCCAAATGCTTTGGCTCCAAGT

< BCL6_1 BCL6_2 >

< S’mu_1 S’mu_2 >

AGACACCTTAGAAGGACAGATTCTGTTCCGAATCACCGATGCGGCGTCAGCAGGActggcctagcggaggctct

||||||||||||||||||||||||||||||||||||||||||||||||||||||||

AGACACCTTAGAAGGACAGATTCTGTTCCGAATCACCGATGCGGCGTCAGCAGGACCCTTTTAATTCttttttttttCCSCTCTKGCCAAAKGCTTTGGCTCCAAGTTTTCTAT

||||||||||| ||||||||||| |||| |||||| ||||||||||||||||||||||

CCGCTGCTCATGATCATTATTTTACCTTTTAATTC-TTTTTTTTTCCGCTCTTGCCAAATGCTTTGGCTCCAAGTTTTCTAT

< BCL6_1 BCL6_2 >

< S’mu_1 S’mu_2 >

GGACAGATTCTGTTCCGAATCACCGATGCGGCGTCAGCAGGActggcctagcggaggctct

|||||| ||||||||||||||||||||||||||||||||||||

GGACAGGTTCTGTTCCGAATCACCGATGCGGCGTCAGCAGGACGATGCGGCGTCATTTTTCTCCGCTCTTGCCAAATGCTTTGGCTCCAAGTTTTCTATGTGTATCT

||||| ||||||||||||||||||||||||||||||||||||||||||||||

CCGCTGCTCATGATCATTATTTTACCTTTTAATTCTTTTTTTTTCCGCTCTTGCCAAATGCTTTGGCTCCAAGTTTTCTATGTGTATCT

< BCL6_1 BCL6_2 >

< S’mu_1 S’mu_2 >

GACTGTGGAGACACCTTAGAAGGACAGATTCTGTTCCGAATCACCGA-TGCggcgtcagcaggactggcctagcggaggctct

||||||||||||||||||||||||||||||||||||||||||||||| |||

GACTGTGGAGACACCTTAGAAGGACAGATTCTGTTCCGAATCACCGAATGCTTTGGCTCCAAGTTTTCTATGTGTATCTATTGATATAAATGTATATATTTA

|| ||||||||||||||||||||||||||||||||||||||||||||||||||||||||

CCGCTGCTCATGATCATTAT...27 nt...CTCTTGCCAAATGCTTTGGCTCCAAGTTTTCTATGTGTATCTATTGATATAAATGTATATATTTA

< BCL6_1 BCL6_2 >

< S’mu_1 S’mu_2 >

CGATGCGGCGTCAGCAGGActggcctagcggaggctct

||||||||||||||||||||||||||||||||||||

CGATGCGGCGTCAGCAGGACTGGCCTAGCGGAGGCTTTTAATTCtttttttttCCGCTCTTGCCAAATAGGCTTTTAATTCtttttttttCCGCTCTTGCCAAAT

||||||||||||||||||||||||||||||||||

CCGCTGCTCATGATCATTATTTTACCTTTTAATTCTTTTTTTTTCCGCTCTTGCCAAAT

< BCL6_1 BCL6_2 >

< S’mu_1 S’mu_2 >

CGATGCGGCGTCAGCAGGactggcctagcggaggctct

||||||||||||||||||

CGATGCGGCGTCAGCAGGGCGTTTTACCTTTTAATTCttttttttCCGCTCTTGCCAAATGATCATTATTTTACCTTTTAATTCTTTTTTTTTCCGCTCTTGCCAAA

|||||||||||||||||||||||||||||||||||||||||||||||||

CCGCTGCTCATGATCATTATTTTACCTTTTAATTCTTTTTTTTTCCGCTCTTGCCAAA

< BCL6_1 BCL6_2 >

< S’mu_1 S’mu_2 >

GGAGACACCTTAGAAGGACAGATTCTGTTCCGAATCACCGATGCGGCGTCAGCAGGActggcctagcggaggctct

|||||||||||||||||||||||||||||||||||||||||||||||||||||||||

GGAGACACCTTAGAAGGACAGATTCTGTTCCGAATCACCGATGCGGCGTCAGCAGGATCATTATTTTACCTTTTAATTCttttttttTCCGCTCTTGCCAAATGCTTTGGCT

|| |||||||||||||||||||||||||||||||||||||||||||||||||||||||||

CCGCTGCTCATGATCATTATTTTACCTTTTAATTCTTTTTTTTTCCGCTCTTGCCAAATGCTTTGGCT

< BCL6_1 BCL6_2 >

< S’mu_1 S’mu_2 >

GATGCGGCGTCAGCAggactggcctagcggaggctct

|||||||||||||||

GATGCGGCGTCAGCATGATCATTATTTTACCTTTTAATTCTTTTTTTTTCCGCTCTTGCCATGCGGCGTCAGCATGATCATTATTTTACCTTTTAATTCCttttttttCCGCTC

||||||||||||||||||||||||||| ||||||||||||||

CCGCTGCTCATGATCATTATTTTACCTTTTAATTCTTTTTTTTTCCGCTC

< BCL6_1 BCL6_2 >

< S’mu_1 S’mu_2 >

TCTGTTCCGAATCACCGATGCGGCGTCAGCAGGActggcctagcggaggctct

||||||||||||||||||||||||||||||||||||||||||||||||||

TCTGTTCCGAATCACCGATGCGGCGTCAGCAGGACTGGCCTAGCGGAGGCCTGCTCATGATCATTATTTTACCTTTTAATTCttttttttTCCGCTCTTGCCAAATGCTTT

|||||||||||||||||||||||||||||||||||||||||||||||||||||||||||||

CCGCTGCTCATGATCATTATTTTACCTTTTAATTCttttttttTCCGCTCTTGCCAAATGCTTT

< BCL6_1 BCL6_2 >

< S’mu_1 S’mu_2 >

AGAAAACTCAATCAGATAGTGCTGAAGACAGGACTGTGGAGACACCTTAGAAGGACAgattctgttccgaatcaccgatgcggcgtcagcaggactggcctagcggaggctct

|||||||||||||||||||||||||||||||||||||||||||||||||||||||||

AGAAAACTCAATCAGATAGTGCTGAAGACAGGACTGTGGAGACACCTTAGAAGGACATTATTTTACCTTTTAATTCttttttttTCCGCTCTTGCCAAATGCTTTGGCT

||||||||||||||||||||||||||||||||||||||||||||||||||||||

CCGCTGCTCATGATCATTATTTTACCTTTTAATTCTTTTTTTTTCCGCTCTTGCCAAATGCTTTGGCT

< BCL6_1 BCL6_2 >

S’mu_2 >

AGTTTTAGGTAAAATGTGCATCATTATCCTGAATTATTTCAGTTAAGcatgtt...108 nt...actggcctagcggaggctct

|||||||||||||||||||||||||||||||||||||||||||||||

AGTTTTAGGTAAAATGTGCATCATTATCCTGAATTATTTCAGTTAAGGGCGGCCGGAGCAGAGAGGACGAGACAGTGCTTGGGGGGTGATTCGGGCTAGTCTGGG

|||||||||||||||||||||||||||||||||||||||||||||||||||||||||||

CCGCTGCTCATGATCATTAT...290 nt...ATTAGGGGGCGGCCGGAGCAGAGAGGACGAGACAGTGCTTGGGGGGTGATTCGGGCTAGTCTGGG

< BCL6_1

< S’mu_1 S’mu_2 >

TGTGGAGACACCTTAGAAGGACAGATTCTGTTCCGAATCACCGATGCGGCGTCAGCAGgactggcctagcggaggctct

||||||||||||||||||||||||||||||||||||||||||||||||||||||||||

TGTGGAGACACCTTAGAAGGACAGATTCTGTTCCGAATCACCGATGCGGCGTCAGCAGATCATTATTTTACCTTTTAATTCttttttttttCCSCTCTKGCCAAAKGCT

|||||||||||||||||||||||| ||||||||||| |||| |||||| |||

CCGCTGCTCATGATCATTATTTTACCTTTTAATTC-TTTTTTTTTCCGCTCTTGCCAAATGCT

< BCL6_1 BCL6_2 >

< S’mu_1 S’mu_2 >

GAGACACCTTAGAAGGACAGATTCTGTTCCGAATCACCGATGCGGCGTCAGCAGgactggcctagcggaggctct

||||||||||||||||||||||||||||||||||||||||||||||||||||||

GAGACACCTTAGAAGGACAGATTCTGTTCCGAATCACCGATGCGGCGTCAGCAGGCTCATGATCATTATTTTACCTTTTAATTCtttttttttCCGCTCTTGCCAA

||||||||||||||||||||||||||||||||||||||||||||||||||||

CCGCTGCTCATGATCATTATTTTACCTTTTAATTCTTTTTTTTTCCGCTCTTGCCAA

< BCL6_1 BCL6_2 >

< S’mu_1 S’mu_2 >

GAGACACCTTAGAAGGACAGATTCTGTTCCGAATCACCGATGCGGCGTCAGCAGGActggcctagcggaggctct

||||||||||||||||||||||||||||||||||||||||||||||||||||||||||

GAGACACCTTAGAAGGACAGATTCTGTTCCGAATCACCGATGCGGCGTCAGCAGGACttttttTCCGCTCTTGCCAAATGCTTTGGCTCCAAGTTTTCTATGTGTATCTATT

|||||||||||||||||||||||||||||||||||||||||||||||||||||||

CCGCTGCTCATGATCATTATTTTACCTTTTAATTCTTTTTTTTTCCGCTCTTGCCAAATGCTTTGGCTCCAAGTTTTCTATGTGTATCTATT

< BCL6_1 BCL6_2 >

< S’mu_1 S’mu_2 >

TCACCGATGCGGCGTCAGCAGGactggcctagcggaggctct

||||||||||||||||||||||

TCACCGATGCGGCGTCAGCAGGGCGTTTTACCTTTTAATTCtttttttttCCGCTCTTGCCAAATGATCATTATTTTACCTTTTAATTCtttttttttCCGCTCTTGCCAAA

|||||||||||||||||||||||||||||||||||||||||||||||||

CCGCTGCTCATGATCATTATTTTACCTTTTAATTCTTTTTTTTTCCGCTCTTGCCAAA

< BCL6_1 BCL6_2 >

< S’mu_1 S’mu_2 >

TTAGAAGGACAGATTCTGTTCCGAATCACCGATGCGGCGTCAGCAGGActggcctagcggaggctct

||||||||||||||||||||||||||||||||||||||||||||||||||||||||

TTAGAAGGACAGATTCTGTTCCGAATCACCGATGCGGCGTCAGCAGGACTGGCCTAGCTCATGATCATTATTTTACCTTTTAATTCTAtttttttCCGCTCTTGCCA

||||||||||||||||||||||||||||||| |||||||||||||||||||

CCGCTGCTCATGATCATTATTTTACCTTTTAATTCTTTTTTTTTCCGCTCTTGCCA

< BCL6_1 BCL6_2 >

< S’mu_1 S’mu_2 >

AGAAGGACAGATTCTGTTCCGAATCACCGATGCGGCGTCAGCAGGActggcctagcggaggctct

|||||||||||||||||||||||||||||||||||||||||||||||

AGAAGGACAGATTCTGTTCCGAATCACCGATGCGGCGTCAGCAGGACGATGCGGCGTCAttttttTCCGCTCTTGCCAAATGCTTTGGCCCCAAGTTTTCTATGTGT

|||||||||||||||||||||||||||||| |||||||||||||||||

CCGCTGCTCATGATCATTATTTTACCTTTTAATTCTTTTTTTTTCCGCTCTTGCCAAATGCTTTGGCTCCAAGTTTTCTATGTGT

< BCL6_1 BCL6_2 >

< S’mu_1 S’mu_2 >

GAGACACCTTAGAAGGACAGATTCTGTTCCGAATCACCGATGCGGCGTCAGCAGGActggcctagcggaggctct

|||||||||||||||||||||||||||||||||||||||||||||||||||||||||

GAGACACCTTAGAAGGACAGATTCTGTTCCGAATCACCGATGCGGCGTCAGCAGGACATGAGCATTATTTTACCTTTTAATTC-tttttttTCCGCTCTTGCCAAATGCTTT

||||| ||||||||||||||||||||| ||||||||||||||||||||||||||||

CCGCTGCTCATGATCATTATTTTACCTTTTAATTCTTTTTTTTTCCGCTCTTGCCAAATGCTTT

< BCL6_1 BCL6_2 >

< S’mu_1 S’mu_2 >

TGTGGAGACACCTTAGAAGGACAGATTCTGTTCCGAATCACCGATGCGGCGTCAGCaggactggcctagcggaggctct

||||||||||||||||||||||||||||||||||||||||||||||||||||||||

TGTGGAGACACCTTAGAAGGACAGATTCTGTTCCGAATCACCGATGCGGCGTCAGCTTTTCCGCTCTTGCCAAATGCTTTGGCTCCAAGTTTTCTATGTGTATCTATTGATA

||||||||||||||||||||||||||||||||||||||||||||||||||||||||

CCGCTGCTCATGATCATTATTTTACCTTTTAATTCTTTTTTTTTCCGCTCTTGCCAAATGCTTTGGCTCCAAGTTTTCTATGTGTATCTATTGATA

< BCL6_1 BCL6_2 >

< S’mu_1 S’mu_2 >

GTGGAGACACCTTAGAAGGACAGATTCTGTTCCGAATCACCGATGCGGCGTCAGCAggactggcctagcggaggctct

||||||||||||||||||| ||||||||||||||||||||||||||||||||||||

GTGGAGACACCTTAGAAGGGCAGATTCTGTTCCGAATCACCGATGCGGCGTCAGCATTATTTTACCTTTTAATTC-ttttttttCCGCTCTTGCCTAATGC

||||||||||||||||||||| ||||||||||||||||||| |||||

CCGCTGCTCATGATCATTATTTTACCTTTTAATTCTTTTTTTTTCCGCTCTTGCCAAATGC

< BCL6_1 BCL6_2 >

< S’mu_1 S’mu_2 >

CTGAAGACAGGACTGTGGAGACACCTTAGAAGGACAGATTCTGTTCCGaatcaccgatgcggcgtcagcaggactggcctagcggaggctct

||||||||||||||||||||||||||||||||||||||||||||||||

CTGAAGACAAGACTGTGGAGACACCTTAGAAGGACAGATTCTGTTCCGTCGTCAGCAGGACTGGCATGATCATTATTTTACCTTTTAATTCtttttttttCCGCTCTTGCCAA

|||||||||||||||||||||||||||||||||||||||||||||||||

CCGCTGCTCATGATCATTATTTTACCTTTTAATTCTTTTTTTTTCCGCTCTTGCCAA

< BCL6_1 BCL6_2 >

< S’mu_1 S’mu_2 >

GTGGAGACACCTTAGAAGGACAGATTCTGTTCCGAatcaccgatgcggcgtcagcaggactggcctagcggaggctct

||||||||||||||||||||||||||||||||||||||||||||||||||||

GTGGAGACACCTTAGAAGGACAGATTCTGTTCCGAATCACCGATGCGGCGTCGGCTTTTCCGCTCTTGCCAAATGCTTTGGCTCCAAGTCTTCTATGTGTATCTAT

|||||||||||||||||||||||||||||||||| ||||||||||||||||

CCGCTGCTCATGATCATTATTTTACCTTTTAATTCTTTTTTTTTCCGCTCTTGCCAAATGCTTTGGCTCCAAGTTTTCTATGTGTATCTAT

< BCL6_1 BCL6_2 >

< S’mu_1 S’mu_2 >

GACACCTTAGAAGGACAGATTCTGTTCCGAATCACCGATGCGGCGTCAGCAGGActggcctagcggaggctct

|||||||||||||||||||||||||||||||||||||||||||||||||||||||

GACACCTTAGAAGGACAGATTCTGTTCCGAATCACCGATGCGGCGTCAGCAGGACATTATTTTACCTTTTAATTCttttttttTCCGCTCTTGCCAAATGCTTTGGCTCCAA

||||||||||||||||||||||||||||||||||||||||||||||||||||||||||

CCGCTGCTCATGATCATTATTTTACCTTTTAATTCTTTTTTTTTCCGCTCTTGCCAAATGCTTTGGCTCCAA

< BCL6_1 BCL6_2 >

S’mu_2 >

GTTGGTGGCATAAGAGAAAACTCAATCAGATAGTGCTGAAGACAGGACTGTGGagacac...48 nt...actggcctagcggaggctct

|||||||||||||||||||||||||||||||||||||||||||| ||||||||

GTTGGTGGCATAAGAGAAAACTCAATCAGATAGTGCTGAAGACAAGACTGTGGCTCCAAGTTTTCTATGTGTATCTATTGATATAAATGTATATATTTATTTATT

|| |||||||||||||||||||||||||||||||||||||||||||||||||||||||

CCGCTGCTCATGATCATTAT...34 nt...CAAATGCTTTGGCTCCAAGTTTTCTATGTGTATCTATTGATATAAATGTATATATTTATTTATT

< BCL6_1 BCL6_2 >

**BJAB, S’μ-BCL6 junctions from 3’DSBs, long overhangs (Cas9 N863A, S’μ_1 + S’μ_2 + BCL6_1 + BCL6_3)**

< S’mu_1 S’mu_2 >

AGAAGGACAGATTCTGTTCCGAATCACCGATGCGGCGTCAGCAGGACTGGCCTAGCGGAGGCTCT

||||||||||||||||||||||||||||||||||||||||||||||||||||||

AGAAGGACAGATTCTGTTCCGAATCACCGATGCGGCGTCAGCAGGACTGGCCTATTTTACCTTTTAATTCTTTTTTTTTCCGCTCTTGCCAAATGCTTTGGCTCCAAG

||||||||||||||||||||||||||||||||||||||||||||||||||||||||

CCGCTGCTCATGATCATTATTTTACCTTTTAATTCTTTTTTTTTCCGCTCTTGCCAAATGCTTTGGCTCCAAG

< BCL6_1

< S’mu_1 S’mu_2 >

CTGAAGACAGGACTGTGGAGACACCTTAGAAGGACAGATTCTGTTCCGAATCACCGATGCGGCGTCAGCAGGACTGGCCTAGCGGAGGCTCT

|||||||||||||||||||||||||||||||||||||||||||||||||||

CTGAAGACAGGACTGTGGAGACACCTTAGAAGGACAGATTCTGTTCCGAATTATTTTACCTTTTAATTC-TTTTTTTTCCGCTCTTGCCAAATGCTTTGGCTCCAA

|||||||||||||||||||| ||||||||||||||||||||||||||||||||||||

CCGCTGCTCATGATCATTATTTTACCTTTTAATTCTTTTTTTTTCCGCTCTTGCCAAATGCTTTGGCTCCAA

< BCL6_1

< S’mu_1 S’mu_2 >

CACCTTAGAAGGACAGATTCTGTTCCGAATCACCGATGCGGCGTCAGCAGGACTGGCCTAGCGGAGGCTCT

|||||||||||||||||||||||||||||||||||||||||||||||||||||||||

CACCTTAGAAGGACAGATTCTGTTCCGAATCACCGATGCGGCGTCAGCAGGACTGGCCTGCTCATGATCATTATTTTACCTTTTAATTC-TTTTTTTTCCGCTCTTG

|||||||||||||||||||||||||||||||| |||||||||||||||||

CCGCTGCTCATGATCATTATTTTACCTTTTAATTCTTTTTTTTTCCGCTCTTG

< BCL6_1

< S’mu_1 S’mu_2 >

ACTGTGGAGACACCTTAGAAGGACAGATTCTGTTCCGAATCACCGATGCGGCG-TCAGCAGGACTGGCCTAGCGGAGGCTCT

||||||||||||||||||||||||||||||||||||||||||||||||||||| |||

ACTGTGGAGACACCTTAGAAGGACAGATTCTGTTCCGAATCACCGATGCGGCGCTCATGATCATTATTTTACCTTTTAATTCTTTTTTTTTCCGCTCTTGCCAAATGCTTTG

||||||||||||||||||||||||||||||||||||||||||||||||||||||||||||

CCGCTGCTCATGATCATTATTTTACCTTTTAATTCTTTTTTTTTCCGCTCTTGCCAAATGCTTTG

< BCL6_1

S’mu_2 >

GTTGGTGGCATAAGAGAAAACTCAATCAGATAGTGCTGAAGACAGGACTGTGGAGACAC...48 nt...ACTGGCCTAGCGGAGGCTCT

||||||||||||||| ||||||||||| |||||||| || ||| ||||||||

GTTGGTGGCATAAGAKAAAACTCAATCMRATAGTGCTSAASACAAGACTGTGGCTCCAAGTTTTCTATGTGTATCTATTGATATAAATGWATATATTTATTTA

||||||||||||||||||||||||||||||||||||||| |||||||||||||

CCGCTGCTCATGATCATTAT...37 nt...ATGCTTTGGCTCCAAGTTTTCTATGTGTATCTATTGATATAAATGTATATATTTATTTA

< BCL6_1

< S’mu_1 S’mu_2 >

GACAGATTCTGTTCCGAATCACCGATGCGGCGTCAGCAGGActggcctagcggaggctct

|||||||||||||||||||||||||||||||||||||||||||||||||||

GACAGATTCTGTTCCGAATCACCGATGCGGCGTCAGCAGGACTGGCCTAGCTCATGATCATTATTTTACCTTTTAATTCtttttttttCCGCTCTCGCCAAATG

|||||||||||||||||||||||||||||||||||||||||||||| ||||||||

CCGCTGCTCATGATCATTATTTTACCTTTTAATTCTTTTTTTTTCCGCTCTTGCCAAATG

< BCL6_1

< S’mu_1 S’mu_2 >

CCTTAGAAGGACAGATTCTGTTCCGAATCACCGATGCGGCGTCAGCAGGActggcctagcggaggctct

||||||||||||||||||||||||||||||||||||||||||||||||||||

CCTTAGAAGGACAGATTCTGTTCCGAATCACCGATGCGGCGTCAGCAGGACTTTCCGCTCTTGCCAAATGCTTTGGCTCCAAGTTTTCTATGTGTATCTATTGATATAA

||||||||||||||||||||||||||||||||||||||||||||||||||||||||||

CCGCTGCTCATGATCATTATTTTACCTTTTAATTCTTTTTTTTTCCGCTCTTGCCAAATGCTTTGGCTCCAAGTTTTCTATGTGTATCTATTGATATAA

< BCL6_1

< S’mu_1 S’mu_2 >

TGTGGAGACACCTTAGAAGGACAGATTCTGTTCCGAATCACCGATGCGGCGTCAGCaggactggcctagcggaggctct

||||||||||||||||||||||||||||||||||||||||||||||||||||||||

TGTGGAGACACCTTAGAAGGACAGATTCTGTTCCGAATCACCGATGCGGCGTCAGCTCATGATCATTATTTTACCTTTTAATTCtttttttttCCGCTCTTGCCAAATGCTTT

|||||||||||||||||||||||||||||||||||||||||||||||||||||||||||

CCGCTGCTCATGATCATTATTTTACCTTTTAATTCTTTTTTTTTCCGCTCTTGCCAAATGCTTT

< BCL6_1

< S’mu_1 S’mu_2 >

TGGAGACACCTTAGAAGGACAGATTCTGTTCCGAATCACCGATGCGGCGTCAGCAGgactggcctagcggaggctct

||||||||||||||||||||||||||||||||||||||||||||||||||||||||

TGGAGACACCTTAGAAGGACAGATTCTGTTCCGAATCACCGATGCGGCGTCAGCAGCTCCAAGTTTTCTATGTGTATCTATTGATATAAATGTATATATTTATTTATTCTAG

|||||||||||||||||||||||||||||||||||||||||||||||||||||||||

CCGCTGCTCATGATCATTAT...39 nt...GCTTTGGCTCCAAGTTTTCTATGTGTATCTATTGATATAAATGTATATATTTATTTATTCTAG

< BCL6_1

< S’mu_1 S’mu_2 >

GAAGGACAGATTCTGTTCCGAATCACCGATGCGGCGTCAGCAGGActggcctagcggaggctct

||||||||||||||||||||||||||||||||||||||||||||||||||||||||||||

GAAGGACAGATTCTGTTCCGAATCACCGATGCGGCGTCAGCAGGACTGGCCTAGCGGAGGTGCTCATGATCATTATTTTACCTTTTAATTCtttttttttCCGCTCTTGCCAAA

||||||||||||||||||||||||||||||||||||||||||||||||||||||

CCGCTGCTCATGATCATTATTTTACCTTTTAATTCtttttttttCCGCTCTTGCCAAA

< BCL6_1

< S’mu_1 S’mu_2 >

AATCAGATAGTGCTGAAGACAGGACTGTGGAGACACCTTAGAAGGACAGATtctgttccgaatcaccgatgcggcgtcagcaggactggcctagcggaggctct

||||||||||||||||||||| ||||||||||||||||||||||||| |||

AATCAGATAGTGCTGAAGACAAGACTGTGGAGACACCTTAGAAGGACGGAT[insert]TCCCACCATAGGATATAGATTGTTATGTATTTATTATTATTATTGTTGTCTTTGA

|||||||||||||||||||||||||||||||||||||||||||||||||||||||

CCGCTGCTCATGATCATTAT...140 nt...CGTCTCTCCCACCATAGGATATAGATTGTTATGTATTTATTATTATTATTGTTGTCTTTGA

< BCL6_1 BCL6_3 >

[insert]: TCTGTTCCGAATCACCAACTAAAGACATCTAAGTGGAAAGAGAGGGAGTCAAACTATTCCTGTTTGCAGATGACATGATCCTATA

< S’mu_1 S’mu_2 >

TGGAGACACCTTAGAAGGACAGATTCTGTTCCGAATCACCGATGCGGCGTCAGCaggactggcctagcggaggctct

||||||||||||||||||||||||||||||||||||||||||||||||||||||

TGGAGACACCTTAGAAGGACAGATTCTGTTCCGAATCACCGATGCGGCGTCAGCTTTTAATTCtttttttttCCGCTCTTGCCAAATGCTTTGGCTCCAAGTTTTCTAT

||||||||||||||||||||||||||||||||||||||||||||||||||||||||

CCGCTGCTCATGATCATTATTTTACCTTTTAATTCTTTTTTTTTCCGCTCTTGCCAAATGCTTTGGCTCCAAGTTTTCTAT

< BCL6_1

< S’mu_1 S’mu_2 >

GAAGACAGGACTGTGGAGACACCTTAGAAGGACAGATTCTGTTCCGAatcaccgatgcggcgtcagcaggactggcctagcggaggctct

||||||| |||||||||||||||||||||||||||||||||||||||||||||

GAAGACAAGACTGTGGAGACACCTTAGAAGGACAGATTCTGTTCCGAATCACC[insert]TCCCACCATAGGATATAGATTGTTATGTATTTATTATTATTATTGTTGTCT

|||||||||||||||||||||||||||||||||||||||||||||||||||

CCGCTGCTCATGATCATTAT...140 nt...CGTCTCTCCCACCATAGGATATAGATTGTTATGTATTTATTATTATTATTGTTGTCT

< BCL6_1 BCL6_3 >

[insert]: AACTAAAGACATCTAAGTGGAAAGAGAGGGAGTCAAACTATTCCTGTTTGCAGATGACATGATCCTATA

< S’mu_1 S’mu_2 >

GACTGTGGAGACACCTTAGAAGGACAGATTCTGTTCCGAatcaccgatgcggcgtcagcaggactggcctagcggaggctct

|||||||||||||||||||| |||||||| |||||||||||||||||||||

GACTGTGGAGACACCTTAGATGGACAGATACTGTTCCGAATCACCGATGCGTATCTATTGATATAAATGTATATATTTATTTATTCTAGCTGTCAGGTGTTAA

||| |||||||||||||||||||||||||||||||||||||||||||||||||||||

CCGCTGCTCATGATCATTAT...53 nt...TTTTCTATGTGTATCTATTGATATAAATGTATATATTTATTTATTCTAGCTGTCAGGTGTTAA

< BCL6_1

< S’mu_1 S’mu_2 >

GACTGTGGAGACACCTTAGAAGGACAGATTCTGttccgaatcaccgatgcggcgtcagcaggactggcctagcggaggctct

|||||||||||||||||||||||||||||||||

GACTGTGGAGACACCTTAGAAGGACAGATTCTG[insert]TCATTATTTTACCTTTTAATTCttttttttttCCGCTCTTGCCAAA

|||||||||||||||||||||| |||||||||||||||||||||||

CCGCTGCTCATGATCATTATTTTACCTTTTAATTC-TTTTTTTTTCCGCTCTTGCCAAA

< BCL6_1

[insert]: GGGTAGTGGTCATCGTGCAAGGCAGACAAGGGCCCTAGGGTGCAGGCAGGCTGGGAAGATCTCCAGAGAATGAGGTGGGCCATCTGGGGG

< S’mu_1 S’mu_2 >

TCTGTTCCGAATCACCGATGCGGCGTCAGCAGGActggcctagcggaggctct

||||||||||||||||||||||||||||||||||||||||||||

TCTGTTCCGAATCACCGATGCGGCGTCAGCAGGACTGGCCTAGCGATCATTATTTTACCTTTTAATTCtttttttttCCGCTCTTGCCAAATGCTTT

|||||||||||||||||||||||||||||||||||||||||||||||||||||

CCGCTGCTCATGATCATTATTTTACCTTTTAATTCTTTTTTTTTCCGCTCTTGCCAAATGCTTT

< BCL6_1

< S’mu_1 S’mu_2 >

TCTGTTCCGAATCACCGATGCGGCGTCAGCAGGActggcctagcggaggctct

||||||||||||||||||||||||||||||||||||||||

TCTGTTCCGAATCACCGATGCGGCGTCAGCAGGACTGGCCCTGCTCATGATCATTATTTTACCTTTTAATTCtttttttttCCGCTCTTGCCAAA

|||||||||||||||||||||||||||||||||||||||||||||||||||||||

CCGCTGCTCATGATCATTATTTTACCTTTTAATTCTTTTTTTTTCCGCTCTTGCCAAA

< BCL6_1

< S’mu_1 S’mu_2 >

TGTGGAGACACCTTAGAAGGACAGATTCTGTTCCGAatcaccgatgcggcgtcagcaggactggcctagcggaggctct

||||||||||||||||||||||||||||||||||||||||||||||||||

TGTGGAGACACCTTAGAAGGACAGATTCTGTTCCGAATCACCGATGCGGCCACCCTCCCTTGTGTTGTTTTGGTTTTTGGAAAGGAGGTGGAGGA

|||||||||||||||||||||||||||||||||||||||||||||||

CCGCTGCTCATGATCATTAT...223 nt...GCTTTTGCCACCCTCCCTTGTGTTGTTTTGGTTTTTGGAAAGGAGGTGGAGGA

< BCL6_1

< S’mu_1 S’mu_2 >

CTGTGGAGACACCTTAGAAGGACAGATTCTGTTCCGAATCACCGATGCGGCGTCAGCAGGActggcctagcggaggctct

|||||||||||||||||||||||||||||||||||||||||||||||||||||||||||||||

CTGTGGAGACACCTTAGAAGGACAGATTCTGTTCCGAATCACCGATGCGGCGTCAGCAGGACTCCCTCTGTTCTCGCTTGCAAACTGCTTTCCTTGCTCCGTCGCT

||||||| ||||||||||||||||||||||||||||||||||||

CCGCTGCTCATGATCATTAT...458 nt...GTTTTTTCCCTCTTTTCTCGCTTGCAAACTGCTTTCCTTGCTCCGTCGCT

< BCL6_1

< S’mu_1 S’mu_2 >

TCTGTTCCGAATCACCGATGCGGCGTCAGCAGGActggcctagcggaggctct

|||||||||||||||||||||||||||||||||||||||

TCTGTTCCGAATCACCGATGCGGCGTCAGCAGGACTGGC[insert]TCATTATTTTACCTTTTAATTCtttttttttCCGCTCTTGCCA

|||||||||||||||||||||||||||||||||||||||||||

CCGCTGCTCATGATCATTATTTTACCTTTTAATTCTTTTTTTTTCCGCTCTTGCCA

< BCL6_1

[insert]: ATTATTTTACCTTTTAATTCTTTTTTTTTCCGCTCTTGCCAAATACTTTGGCTCCAAGTTTTCTATGTGTATCTATTGATATAAATGTATATATTTATTTATTCTAGCTGTCAGGTGTTAAATAAATGCCGAAGAC

< S’mu_1 S’mu_2 >

TCTGTTCCGAATCACCGATGCGGCGTCAGCAGGActggcctagcggaggctct

||||||||||||||||||||||||||||||||||||||||||

TCTGTTCCGAATCACCGATGCGGCGTCAGCAGGACTGGCCTAGCTCATGATCATTATTTTACCTTTTAATTCtttttttttCCGCTCTTGCCA

|||||||||||||||||||||||||||||||||||||||||||||||||||

CCGCTGCTCATGATCATTATTTTACCTTTTAATTCTTTTTTTTTCCGCTCTTGCCA

< BCL6_1

< S’mu_1 S’mu_2 >

AAGGACAGATTCTGTTCCGAATCACCGATGCGGCGTCAGCAGGactggcctagcggaggctct

|||||||||||||||||||||||||||||||||||||||||||

AAGGACAGATTCTGTTCCGAATCACCGATGCGGCGTCAGCAGGTCTGCTCATGATCATTATTTTACCTTTTAATTCtttttttttCCGCTCTTGCCAAAT

||||||||||||||||||||||||||||||||||||||||||||||||||||||||

CCGCTGCTCATGATCATTATTTTACCTTTTAATTCTTTTTTTTTCCGCTCTTGCCAAAT

< BCL6_1

S’mu_2 >

TTATCCTGAATTATTTCAGTTAAGCATGTTAGTTGGTGGCATAAGAGAAAACTCAATcagata...75 nt...actggcctagcggaggctct

|||||||||||||||||||||||||||||||||||||||||||||||||||||||||

TTATCCTGAATTATTTCAGTTAAGCATGTTAGTTGGTGGCATAAGAGAAAACTCAATTATTTTACCTTTTAGTTCtttttttttCCGCTCTTGCCAAATGCTTTGGCTCC

|||||||||||||||| ||||||||||||||||||||||||||||||||||||||

CCGCTGCTCATGATCATTATTTTACCTTTTAATTCTTTTTTTTTCCGCTCTTGCCAAATGCTTTGGCTCC

< BCL6_1

< S’mu_1 S’mu_2 >

GAAGGACAGATTCTGTTCCGAATCACCGATGCGGCGTCAGCAGGActggcctagcggaggctct

||||||||||||||||||||||||||||||||||||||||||||||||||||

GAAGGACAGATTCTGTTCCGAATCACCGATGCGGCGTCAGCAGGACTGGCCtttttttttCCGCTCTTGCCAAATGCTTTGGCTCCAAGTTTTCTATGTGTATC

||||||||||||||||||||||||||||||||||||||||||||||||||||||

CCGCTGCTCATGATCATTATTTTACCTTTTAATTCTTTTTTTTTCCGCTCTTGCCAAATGCTTTGGCTCCAAGTTTTCTATGTGTATC

< BCL6_1

< S’mu_1 S’mu_2 >

TTAGAAGGACAGATTCTGTTCCGAATCACCGATGCGGCGTCAGCAGGActggcctagcggaggctct

|||||||||||||||||||||||||||||||||||||||||||||||||

TTAGAAGGACAGATTCTGTTCCGAATCACCGATGCGGCGTCAGCAGGACCTTTTAATTCtttttttttCCGCTCTTGCCAAATGCTTTGGCTCCA

||||||||||||||||||||||||||||||||||||||||||||||||

CCGCTGCTCATGATCATTATTTTACCTTTTAATTCTTTTTTTTTCCGCTCTTGCCAAATGCTTTGGCTCCA

< BCL6_1

< S’mu_1 S’mu_2 >

AGAAGGACAGATTCTGTTCCGAATCACCGATGCGGCGTCAGCAGGActggcctagcggaggctct

||||||||||||||||||||||||||||||||||||||||||||||||||||

AGAAGGACAGATTCTGTTCCGAATCACCGATGCGGCGTCAGCAGGACTGGCCCTTTTAATTCtttttttttCCGCTCTTGCCAAATGCTTTGGCTCCAAGTTTTC

||||||||||||||||||||||||||||||||||||||||||||||||||||||

CCGCTGCTCATGATCATTATTTTACCTTTTAATTCTTTTTTTTTCCGCTCTTGCCAAATGCTTTGGCTCCAAGTTTTC

< BCL6_1

**Ramos, S’μ-S’α junctions from blunt DSBs (WT Cas9, S’μ_1 + S’α_1)**

< S’mu_1

AGACAGGACTGTGGAGACACCTTAGAAGGACAGATTCTGTTCCGAATCACCGATGCGGCGTCAG

|||||||||||||||||||||||||||||||||||||||||||||||

AGACAGGACTGTGGAGACACCTTAGAAGGACAGATTCTGTTCCGAAT[insert]TGACAGCCGTCATCCCAGAGTGAGAGCATGCAGAGCTGGGGCCTCAGGGAGG

||||||||||||||||||||||||||||||||||||||||||||||||||||

GTGTGACAGCCGTCATCCCAGAGTGAGAGCATGCAGAGCTGGGGCCTCAGGGAGG

< S’alpha_1

[insert]: ACCAGAAAAGAGATTTTCTTGTCCCGCATGGAGCAGATTCTGCCATGGCAAAACATGGTGGAAGTCATCGAGCCGTTTTACCCCAAGGCTGGTAATGGCCGGCGAC

< S’mu_1

ATAGTGCTGAAGACAGGACTGTGGAGACACCTTAGAAGGACAGATTCTGTTCCGAATCACCGATGCGGCGTCAG

|||||||||||||||||||||||||||||||||||||||||||||||||||||||||

ATAGTGCTGAAGACAGGACTGTGGAGACACCTTAGAAGGACAGATTCTGTTCCGAATTGACAGCCGTCATCCCAGAGTGAGAGCATGCAGAGCTGGGGCCTCGGGGAGG

||||||||||||||||||||||||||||||||||||||||||||| ||||||

GTGTGACAGCCGTCATCCCAGAGTGAGAGCATGCAGAGCTGGGGCCTCAGGGAGG

< S’alpha_1

< S’mu_1

ATAGTGCTGAAGACAGGACTGTGGAGACACCTTAGAAGGACAGATTCTGTTCCGAATCACCGATGCGGCGTCAG

|||||||||||||||||||||||||||||||||||||||||||||||||| ||||

ATAGTGCTGAAGACAGGACTGTGGAGACACCTTAGAAGGACAGATTCTGTCCCGAGTTGACAGCCGTCATCCCAGAGTGAGAGCATGCAGAGCTGGGGCCTCAGGGAGGTGGTT

|||||||||||||||||||||||||||||||||||||||||||||||||||||||||

GTGTGACAGCCGTCATCCCAGAGTGAGAGCATGCAGAGCTGGGGCCTCAGGGAGGTGGTT

< S’alpha_1

< S’mu_1

AGTGCTGAAGACAGGACTGTGGAGACACCTTAGAAGGACAGATTCTGTTCCGAATCACCGATGCGGCGTCAG

|||||||||||||||||||||||||||||||||||||||||||||||||||||||

AGTGCTGAAGACAGGACTGTGGAGACACCTTAGAAGGACAGATTCTGTTCCGAATTGACAGCCGTCATCCCAGAGTGAGAGCATGCAGAGCTGGGGCCTCAGGGAGGTGGTT

|||||||||||||||||||||||||||||||||||||||||||||||||||||||||

GTGTGACAGCCGTCATCCCAGAGTGAGAGCATGCAGAGCTGGGGCCTCAGGGAGGTGGTT

< S’alpha_1

< S’mu_1

CAGGACTGTGGAGACACCTTAGAAGGACAGATTCTGTTCCGAATCA-CCGATGCGGCGTCAG

|||||||||||||||||||||||||||||||||||||||||||||| |||

CAGGACTGTGGAGACACCTTAGAAGGACAGATTCTGTTCCGAATCAGCCGTCATCCCAGAGTGAGAGCATGCAGAGCTGGGGCCTCGGGGAGGTGGTTAGGGCC

|||||||||||||||||||||||||||||||||||||||||| |||||||||||||||||

GTGTGACAGCCGTCATCCCAGAGTGAGAGCATGCAGAGCTGGGGCCTCAGGGAGGTGGTTAGGGCC

< S’alpha_1

< S’mu_1

AGTGCTGAAGACAGGACTGTGGAGACACCTTAGAAGGACAGATTCTGTTCCGAATCACCGATGCGGCGTCAG

|||||||||||||||||||||||||||||||||||||||||||||||||||||||

AGTGCTGAAGACAGGACTGTGGAGACACCTTAGAAGGACAGATTCTGTTCCGAATTGACAGCCGTCATCCCAGAGTGAGAGCATGCAGAGCTGGGGCCTCAGGGAGGTGGTT

|||||||||||||||||||||||||||||||||||||||||||||||||||||||||

GTGTGACAGCCGTCATCCCAGAGTGAGAGCATGCAGAGCTGGGGCCTCAGGGAGGTGGTT

< S’alpha_1

< S’mu_1

TTTAGGATAAGTTTTAGGTAAAATGTGCATCATTATCCTGAATTATTTCAGTTAAGCAT...81 nt...AATCACCGATGCGGCGTCAG

|||||||||||||||||||||||||||||||||||||||||||||||||||||||||||

TTTAGGATAAGTTTTAGGTAAAATGTGCATCATTATCCTGAATTATTTCAGTTAAGCATCCCAAGGTTCATAACAAAAGCTCCAGAGCTTCCCTGAACCGCCAGGTGTGTC

||||||||||||||||||||||||||||||||||||||||||||||||||||||||

GTGTGACAGCCGTCATCCCA...349 nt...CCGGGGGCATCCCAAGGTTCATAACAAAAGCTCCAGAGCTTCCCTGAACCGCCAGGTGTGTC

< S’alpha_1

< S’mu_1

AGACAGGACTGTGGAGACACCTTAGAAGGACAGATTCTGTTCCGAATCACCGATGCGGCGTCAG

||||||||||||||||||||||||||||||||||||||||||||

AGACAGGACTGTGGAGACACCTTAGAAGGACAGATTCTGTTCCGGATTGACAGCCGTCATCCCAGAGTGAGAGCATGCAGAGCTGGGGCCTCAGGGAGGTGGT

||||||||||||||||||||||||||||||||||||||||||||||||||||||||

GTGTGACAGCCGTCATCCCAGAGTGAGAGCATGCAGAGCTGGGGCCTCAGGGAGGTGGT

< S’alpha_1

< S’mu_1

AGATAGTGCTGAAGACAGGACTGTGGAGACACCTTAGAAGGACAGATTCTGTTCCGAATCACCGATGCGGCGTCAG

||||||||||||||||||||||||||||||||||||||||||||||||||||||||

AGATAGTGCTGAAGACAGGACTGTGGAGACACCTTAGAAGGACAGATTCTGTTCCGTCATCCCAGAGTGAGAGCATGCAGAGCTGGGGCCTCGGGGAGGTGGTTAG

||||||||||||||||||||||||||||||||||||||| |||||||||||||

GTGTGACAGCCGTCATCCCAGAGTGAGAGCATGCAGAGCTGGGGCCTCAGGGAGGTGGTTAG

< S’alpha_1

< S’mu_1

TGCTGAAGACAGGACTGTGGAGACACCTTAGAAGGACAGATTCTGTTCCGAATCACCGATGCGGCGTCAG

||||||||||||||||||||||||| |||||||||||||||||||||||||

TGCTGAAGACAGGACTGTGGAGACA-CTTAGAAGGACAGATTCTGTTCCGATGACAGCCGTCATCCCAGAGTGAGAGCATGCAGAGCTGGGGCCTCGGGGAGGTGGTT

||||||||||||||||||||||||||||||||||||||||||||| |||||||||||

GTGTGACAGCCGTCATCCCAGAGTGAGAGCATGCAGAGCTGGGGCCTCAGGGAGGTGGTT

< S’alpha_1

< S’mu_1

TGCTAGTTTGTGCAAACAGCATATCAACTTCTAAACTGCATTCATTTTTAAAGTAAGATG...403 nt...AATCACCGATGCGGCGTCAG

||||||||||||||||||||||||||||||||||||||||||||||||||||||

TGCTAGTTTGTGCAAACAGCATATCAACTTCTAAACTGCATTCATTTTTAAAGTGAAAAGAAGGGGGTCTGTGAACCAGGAATCGACCCTTCCTGGAACTTGACCC

|||||||||| ||||||||||||||||||||||||||||||||||||||||||||

GTGTGACAGCCGTCATCCCA...148 nt...GGACGCAGTGAAAAGATGGGGGTCTGTGAACCAGGAATCGACCCTTCCTGGAACTTGACCC

< S’alpha_1

< S’mu_1

ACTCAATCAGATAGTGCTGAAGACAGGACTGTGGAGACACCTTAGAAGGACAGATTCTGTTCCGAATCACCGATGCGGCGTCAG

|||||||||||||||||||||||||||||||||

ACTCAATCAGATAGTGCTGAAGACAGGACTGTGTGCGAGCGAGCGCGCGGCTGCCTGCAGGGGCGCCTGTGACAGCCGTCATCCCAGAGTGAGAGCATGCAGAGC

||||||||||||||||||||||||||||||||||||||

GTGTGACAGCCGTCATCCCAGAGTGAGAGCATGCAGAGC

< S’alpha_1

< S’mu_1

GTGCTGAAGACAGGACTGTGGAGACACCTTAGAAGGACAGATTCTGTTCCGAATCACCGATGCGGCGTCAG

||||||||||||||||||||||||||||||||||||||||||||||||||||

GTGCTGAAGACAGGACTGTGGAGACACCTTAGAAGGACAGATTCTGTTCCGATGACAGCCGTCATCCCAGAGTGAGAGCATGCAGAGCTGGGGCCTCAGGGAGGTGGT

||||||||||||||||||||||||||||||||||||||||||||||||||||||||

GTGTGACAGCCGTCATCCCAGAGTGAGAGCATGCAGAGCTGGGGCCTCAGGGAGGTGGT

< S’alpha_1

< S’mu_1

TAGTGCTGAAGACAGGACTGTGGAGACACCTTAGAAGGACAGATTCTGTTCCGAATCACCGATGCGGCGTCAG

||||||||||||||||||||||||||||||||||||||||||||||||||||||||

TAGTGCTGAAGACAGGACTGTGGAGACACCTTAGAAGGACAGATTCTGTTCCGAATTGACAGCCGTCATCCCAGAGTGAGAGCATGCAGAGCTGGGGCCTCAGGGAGGTGG

|||||||||||||||||||||||||||||||||||||||||||||||||||||||

GTGTGACAGCCGTCATCCCAGAGTGAGAGCATGCAGAGCTGGGGCCTCAGGGAGGTGG

< S’alpha_1

< S’mu_1

TAGTGCTGAAGACAGGACTGTGGAGACACCTTAGAAGGACAGATTCTGTTCCGAATCACCGATGCGGCGTCAG

||||||||||||||||||||||||||||||||||||||||||||||||||||||||

TAGTGCTGAAGACAGGACTGTGGAGACACCTTAGAAGGACAGATTCTGTTCCGAATTGACAGCCGTCATCCCAGAGTGAGAGCATGCAGAGCTGGGGCCTCAGGGAGGTGG

|||||||||||||||||||||||||||||||||||||||||||||||||||||||

GTGTGACAGCCGTCATCCCAGAGTGAGAGCATGCAGAGCTGGGGCCTCAGGGAGGTGG

< S’alpha_1

< S’mu_1

TAGTGCTGAAGACAGGACTGTGGAGACACCTTAGAAGGACAGATTCTGTTCCGAATCACCGATGCGGCGTCAG

||||||||||||||||||||||||||||||||||||||||||||||||||||||||

TAGTGCTGAAGACAGGACTGTGGAGACACCTTAGAAGGACAGATTCTGTTCCGAATTGACAGCCGTCATCCCAGAGTGAGAGCATGCAGAGCTGGGGCCTCAGGGAGGTGG

|||||||||||||||||||||||||||||||||||||||||||||||||||||||

GTGTGACAGCCGTCATCCCAGAGTGAGAGCATGCAGAGCTGGGGCCTCAGGGAGGTGG

< S’alpha_1

< S’mu_1

AGACAGGACTGTGGAGACACCTTAGAAGGACAGATTCTGTTCCGAATCACCGATGCGGCGTCAG

|||||||||||||||||||||||||||||||||||||||||||||||

AGACAGGACTGTGGAGACACCTTAGAAGGACAGATTCTGTTCCGAATGGACAGCCGTCATCCCAGAGTGAGAGCATGCAGAGCTGGGGCCTCAGGGAGGTGG

||||||||||||||||||||||||||||||||||||||||||||||||||||||

GTGTGACAGCCGTCATCCCAGAGTGAGAGCATGCAGAGCTGGGGCCTCAGGGAGGTGG

< S’alpha_1

< S’mu_1

TTATTTCAGTTAAGCATGTTAGTTGGTGGCATAAGAGAAAACTCAATCA...55 nt...AATCACCGATGCGGCGTCAG

|||||||||||||||||||||||||||||||||||||||||||

TTATTTCAGTTAAGCATGTTAGTTGGTGGCATAAGAGAAAACTTATGTGTCCCAGAGTGAGAGCATGCAGAGCTGGGGCCTCAGGGAGGTGGTTAGGGCCTGAG

|||||||||||||||||||||||||||||||||||||||||||||||||||||||

GTGTGACAGCCGTCATCCCAGAGTGAGAGCATGCAGAGCTGGGGCCTCAGGGAGGTGGTTAGGGCCTGAG

< S’alpha_1

< S’mu_1

GTGCTGAAGACAGGACTGTGGAGACACCTTAGAAGGACAGATTCTGTTCCGAATCACCGATGCGGCGTCAG

||||||||||||||||||||||||||||||||||||||||||||||||||||||

GTGCTGAAGACAGGACTGTGGAGACACCTTAGAAGGACAGATTCTGTTCCGAATTTGACAGCCGTCATCCCAGAGTGAGAGCATGCAGAGCTGGGGCCTCGGGGAGGTGGT

||||||||||||||||||||||||||||||||||||||||||||| ||||||||||

GTGTGACAGCCGTCATCCCAGAGTGAGAGCATGCAGAGCTGGGGCCTCAGGGAGGTGGT

< S’alpha_1

< S’mu_1

TAGTGCTGAAGACAGGACTGTGGAGACACCTTAGAAGGACAGATTCTGTTCCGAATCACCGATGCGGCGTCAG

||||||||||||||||||||||||||||||||||||||||||||||||||||||||

TAGTGCTGAAGACAGGACTGTGGAGACACCTTAGAAGGACAGATTCTGTTCCGAATTGACAGCCGTCATCCCAGAGTGAGAGCATGCAGAGCTGGGGCCTCAGGGAGGTGG

|||||||||||||||||||||||||||||||||||||||||||||||||||||||

GTGTGACAGCCGTCATCCCAGAGTGAGAGCATGCAGAGCTGGGGCCTCAGGGAGGTGG

< S’alpha_1

< S’mu_1

TAAGAGAAAACTCAATCAGATAGTGCTGAAGACAGGACTGTGGAGACACCTTAGAAGGACAGATTCTGTTCCGAATCACCGATGCGGCGTCAG

|||||||||||||||||||||||||||||||||||||||||||||||||||||||||

TAAGAGAAAACTCAATCAGATAGTGCTGAAGACAGGACTGTGGAGACACCTTAGAAGAGGGGGAACACTCAAAAATGTGACTCGGTCCCTGATCTCACTAGAGACCCAAA

|||||||||| ||||||||||||| ||||||||| |||||||||||||||| ||

GTGTGACAGCCGTCATCCCA...41 nt...GGGCCTGAGGGGGAACCCTCAAAAATGTGATTCGGTCCCTAATCTCACTAGAGACCCTAA

< S’alpha_1

< S’mu_1

ATTAAACAGTCTTAGGGAGAGTTTATGACTGTATTCAAAAAGTTTTTTAAATTAGCTT...337 nt...AATCACCGATGCGGCGTCAG

||||||||||||||||||||||||||||||||||||||||||||||||||||

ATTAAACAGTCTTAGGGAGAGTTTATGACTGTATTCAAAAAGTTTTTTAAATCATCCCAGAGTGAGAGCATGCAGAGCTGGGGCCTCGGGGAGGTGGTTAGGGCC

|||||||||||||||||||||||||||||||||||| |||||||||||||||||

GTGTGACAGCCGTCATCCCAGAGTGAGAGCATGCAGAGCTGGGGCCTCAGGGAGGTGGTTAGGGCC

< S’alpha_1

**Ramos, S’μ-S’α junctions from 5’DSBs, short overhangs (Cas9 D10A, S’μ_1 + S’μ_2 + S’α_1 + S’α_2)**

S’mu_2 >

TAAGTTTTAGGTAAAATGTGCATCATTATCCTGAATTATTTCAGTTAAGCATGTTAGTT...104 nt...ACTGGCCTAGCGGAGGCTCT

|||||||||||||||||||||||||||||||||||||||||||||||||||||

TAAGTTTTAGGTAAAATGTGCATCATTATCCTGAATTATTTCAGTTAAGCATGCAGAGCTGGGGCCTCGGGGAGGTGGTTAGGGCCTGAGGGGGAACACTCAAAAAT

||||||||||||||||||||| |||||||||||||||||||||||||||| |||||||||

GTGTGACAGCCGTCATCCCAGAGTGAGAGCATGCAGAGCTGGGGCCTCAGGGAGGTGGTTAGGGCCTGAGGGGGAACCCTCAAAAAT

< S’alpha_1 S’alpha_2 >

< S’mu_1 S’mu_2 >

ACACCTTAGAAGGACAGATTCTGTTCCGAATCACCGATGCGGCGTCAGCAGGACTGGCCTAGCGGAGGCTCT

||||| ||||||||||||||||||||||||||||||||||||||||||||||

ACACCCTAGAAGGACAGATTCTGTTCCGAATCACCGATGCGGCGTCAGCAGGTGTGACTCGGTCCCTGATCTCACTAGAGACCCAAAAGAAAAGGGACCCCAGAGAGCAG

||||| ||||||||| |||||||||||||||| |||||||||||||||||||||||||

GTGTGACAGCCGTCATCCCA...60 nt...CAAAAATGTGATTCGGTCCCTAATCTCACTAGAGACCCTAAAGAAAAGGGACCCCAGAGAGCAG

< S’alpha_1

S’mu_2 >

CTGAATTATTTCAGTTAAGCATGTTAGTTGGTGGCATAAGAGAAAACTCAATCAGATAG...74 nt...ACTGGCCTAGCGGAGGCTCT

||||||||||||||||||||||||||| |||||||||||||||||||||||||

CTGAATTATTTCAGTTAAGCATGTTAGCTGGTGGCATAAGAGAAAACTCAATCTCTCACTAGAGACCCAAAAGAAAAGGGACCCCAGAGAGCAGCCCTGCCCTCTTTCC

||||||||||||||| ||||||||||||||||||||||||||||||||||||||||

GTGTGACAGCCGTCATCCCA...77 nt...CCCTAATCTCACTAGAGACCCTAAAGAAAAGGGACCCCAGAGAGCAGCCCTGCCCTCTTTCC

< S’alpha_1

< S’mu_1 S’mu_2 >

GAATCACCGATGCGGCGTCAGCAGGACTGGCCTAGCGGAGGCTCT

||||||||||||||||||||||||

GAATCACCGATGCGGCGTCAGCAGAGCTCTGAGGCCCCAGCTCTGCATGCTCTCACTCTGGAGGCCCCAGCTCTGCATGCTCTCGCTCTGGGGGAGGAGGTTAGGGCCTGAGGG

|||||| ||||||||||||||||

GTGTGACAGCCGTCATCCCAGAGTGAGAGCATGCAGAGCTGGGGCCTCAGGGAGGTGGTTAGGGCCTGAGGG

< S’alpha_1 S’alpha_2 >

< S’mu_1 S’mu_2 >

GTTCCGAATCACCGATGCGGCGTCAGCAGGACTGGCCTAGCGGAGGCTCT

|||||||||||||||||||||||||||||||||||||||||||||||

GTTCCGAATCACCGATGCGGCGTCAGCAGGACTGGCCTAGCGGAGGCATCGATGTGTCCCAGAGTGAGAGCATGCAGAGCTGGGGCCTCAGGGAGGTG

||||||||||||||||||||||||||||||||||||||||||

GTGTGACAGCCGTCATCCCAGAGTGAGAGCATGCAGAGCTGGGGCCTCAGGGAGGTG

< S’alpha_1

< S’mu_1 S’mu_2 >

TGGAGACACCTTAGAAGGACAGATTCTGTTCCGAATCACCGATGCGGCGTCAGCAGGACTGGCCTAGCGGAGGCTCT

|||||||||||||||||||||||||||||||||||||||||||||||||||||||

TGGAGACACCTTAGAAGGACAGATTCTGTTCCGAATCACCGATGCGGCGTCAGCACAGTTAGGGCCTGAGGGGGAACCCTC-AAAATGTGATTCGGTCCCTAATCTCACTAGA

|||||||||||||||||||||||| |||||||||||||||||||||||||||||||

GTGTGACAGCCGTCATCCCAGAGTGAGAGCATGCAGAGCTGGGGCCTCAGGGAGGTGGTTAGGGCCTGAGGGGGAACCCTCAAAAATGTGATTCGGTCCCTAATCTCACTAGA

< S’alpha_1 S’alpha_2 >

S’mu_2 >

AAAGTAAGATGTTTAAGAAATTAAACAGTCTTAGGGAGAGTTTATGACTGTATTCAAAAAGTTTTT...372 nt...ACTGGCCTAGCGGAGGCTCT

||||||||||||||||||||||||||||||||||||||||||||||||||||||||||||

AAAGTAAGATGTTTAAGAAATTAAACAGTCTTAGGGAGAGTTTATGACTGTATTCAAAAATGTGATTCGGTCCCTAATCTCACTAGAGACCCTAAAGAAAAGGGACCCCAGAG

||||||||||||||||||||||||||||||||||||||||||||||||||||||||||||

GTGTGACAGCCGTCATCCCA...53 nt...GAACCCTCAAAAATGTGATTCGGTCCCTAATCTCACTAGAGACCCTAAAGAAAAGGGACCCCAGAG

< S’alpha_1

S’mu_2 >

CGATGCGGCGTCAGCAGGACTGGCCTAGCGGAGGCTCT

|||||||||||||||||

CGATGCGGCGTCAGCAGAGCTCCGAGGCCCCAGCTCTGCATGCTCTCACTCTGGAGGCCCCAGCTCTGCATGCTCTCACTCTGGGGGAGGTGGTTAGGGCCT

||||||||||||||||||

GTGTGACAGCCGTCATCCCAGAGTGAGAGCATGCAGAGCTGGGGCCTCAGGGAGGTGGTTAGGGCCT

< S’alpha_1

S’mu_2 >

GTTAACTCGTTTTCTCTTTGTGATTAAGGAGAAACACTTTGATATTCTGATAGAGTGGCCTTCA...207 nt...ACTGGCCTAGCGGAGGCTCT

||||||||||||||||||||||||||||||||||||||||||||||||||||||||||

GTTAACTCGTTTTCTCTTTGTGATTAAGGAGAAACACTTTGATATTCTGATAGAGTGGTTAGGGCCTGAGGGGGAACCCTCAAAAATGTGATTCGGTCCCTAATCTCACTAG

||||||||||||||||||||||||||||||||||||||||||||||||||||||||||

GTGTGACAGCCGTCATCCCAGAGTGAGAGCATGCAGAGCTGGGGCCTCAGGGAGGTGGTTAGGGCCTGAGGGGGAACCCTCAAAAATGTGATTCGGTCCCTAATCTCACTAG

< S’alpha_1 S’alpha_2 >

< S’mu_1 S’mu_2 >

TGCTGAAGACAGGACTGTGGAGACACCTTAGAAGGACAGATTCTGTTCCGAATCACCGATGCGGCGTCAGCAGGACTGGCCTAGCGGAGGCTCT

|||||||||||||||||||||||||||||||||||||||||||||||||||||||

TGCTGAAGACAGGACTGTGGAGACACCTTAGAAGGACAGATTCTGTTCCGAATCAAGAGCATGCAGAGCTGGGGCCTCAGGGAGGTGGCTAGGGCCTGAGGGGGAACCCT

||||||||||||||||||||||||||||||||| |||||||||||||||||||||

GTGTGACAGCCGTCATCCCAGAGTGAGAGCATGCAGAGCTGGGGCCTCAGGGAGGTGGTTAGGGCCTGAGGGGGAACCCT

< S’alpha_1 S’alpha_2 >

S’mu_2 >

AAAACTCAATCAGATAGTGCTGAAGACAGGACTGTGGAGACACCTTAGAAGGACAGATTCTGT...28 nt...ACTGGCCTAGCGGAGGCTCT

|||||||||||||||||||||||||||||||||||||||||||||||||||||||||

AAAACTCAATCAGATAGTGCTGAAGACAGGACTGTGGAGACACCTTAGAAGGACAGAACCAGTCTATGGTATTTTTGTTATTGCAGCCAGAAATGACTAAGACGCAGCAGC

||||||||||||||||||||||||||||||||||||||||||||| ||||||||

GTGTGACAGCCGTCATCCCA...270 nt...GAACCCACCAGTCTATGGTATTTTTGTTATTGCAGCCAGAAATGACTAAGATGCAGCAGC

< S’alpha_1

S’mu_2 >

TTCATTTTTAAAGTAAGATGTTTAAGAAATTAAACAGTCTTAGGGAGAGTTTATGACTGT...387 nt...ACTGGCCTAGCGGAGGCTCT

||||||||||||||||||||||||||||||||||||||||||||||||||||||

TTCATTTTTAAAGTAAGATGTTTAAGAAATTAAACAGTCTTAGGGAGAGTTTATAGAGCTGGGGCCTCGGGGAGGTGGTTAGGGCCTGAGGGGGAACACTCAAAAATGT

|||||||||||||| |||||||||||||||||||||||||||| |||||||||||

GTGTGACAGCCGTCATCCCAGAGTGAGAGCATGCAGAGCTGGGGCCTCAGGGAGGTGGTTAGGGCCTGAGGGGGAACCCTCAAAAATGT

< S’alpha_1 S’alpha_2 >

< S’mu_1 S’mu_2 >

AATCAGATAGTGCTGAAGACAGGACTGTGGAGACACCTTAGAAGGACAGATTCTGTTCCGAATCACCGATGCGGCGTCAGCAGGACTGGCCTAGCGGAGGCTCT

|||||||||||||||||||||||||||||||||||||||||||||||||

AATCAGATAGTGCTGAAGACAGGACTGTGGAGACACCTTAGAAGGACAGCCTGCTGCCATCCTGACCTTGAACTTCCAGCTTC-----CC-TGAGAAATGAATG

|||||||||||||||||||||||||||||||||| || |||||||||||||

GTGTGACAGCCGTCATCCCA...201 nt...CTTGACCCTGCTGCCATCCTGACCTTGAACTTCCAGCTTCGAGAACCGTGAGAAATGAATG

< S’alpha_1

< S’mu_1 S’mu_2 >

CTCAATCAGATAGTGCTGAAGACAGGACTGTGGAGACACCTTAGAAGGACAGATTCTGTTCCGAATCACCGATGCGGCGTCAGCAGGACTGGCCTAGCGGAGGCTCT

||||||||||||||||||||||||||||||||||||||||||||||||||||||||||

CTCAATCAGATAGTGCTGAAGACAGGACTGTGGAGACACCTTAGAAGGACAGATTCTGAGGGGGAACCCTCAAAAATGTGATTCGGTCCCTAATCTCCCTAGAGACCC

|||||||||||||||||||||||||||||||||||||||||| ||||||||||

GTGTGACAGCCGTCATCCCA...39 nt...TAGGGCCTGAGGGGGAACCCTCAAAAATGTGATTCGGTCCCTAATCTCACTAGAGACCC

< S’alpha_1

< S’mu_1 S’mu_2 >

GAAAACTCAATCAGATAGTGCTGAAGACAGGACTGTGGAGACACCTTAGAAGGACAGATTCTGTTCCGAATCACCGATGCGGCGTCAGCAGGACTGGCCTAGCGGAGGCTCT

|||||||||||||||||||||||||||| |||||||||||||||||||||||||||

GAAAACTCAATCAGATAGTGCTGAAGACGGGACTGTGGAGACACCTTAGAAGGACATGCAGAGCTGGGGCCTCGGGGAGGTGGTTAGGGCCTGAGGGGGAACACTCAAAAA

||||||||||||||||||| |||||||||||||||||||||||||||| ||||||||

GTGTGACAGCCGTCATCCCAGAGTGAGAGCATGCAGAGCTGGGGCCTCAGGGAGGTGGTTAGGGCCTGAGGGGGAACCCTCAAAAA

< S’alpha_1 S’alpha_2 >

< S’mu_1 S’mu_2 >

AGACAGGACTGTGGAGACACCTTAGAAGGACAGATTCTGTTCCGAATCACCGATGCGGCGTCAGCAGGACTGGCCTAGCGGAGGCTCT

|||||||||||||||||||||||||||||||||||||||||||||||

AGACAGGACTGTGGAGACACCTTAGAAGGACAGATTCTGTTCCGAATTGACAGCCGTCATCCCAGAGTGAGAGCATGCAGAGCTGGGGCCTCGGGGAGGTGG

||||||||||||||||||||||||||||||||||||||||||||| |||||||||

GTGTGACAGCCGTCATCCCAGAGTGAGAGCATGCAGAGCTGGGGCCTCAGGGAGGTGG

< S’alpha_1

< S’mu_1 S’mu_2 >

ACTGTGGAGACACCTTAGAAGGACAGATTCTGTTCCGAATCACCGATGCGGCGTCAGCAGGACTGGCCTAGCGGAGGCTCT

||||||||||||||||||||||||||||||||||||||||||||||||||||||

ACTGTGGAGACACCTTAGAAGGACAGATTCTGTTCCGAATCACCGATGCGGCGTAGAGAGCATGCAGAGCTGGGGCCTCAGGGAGGTGGTTAGGGCCTGAGGGGGA

|||||||||||||||||||||||||||||||||||||||||||||||||||

GTGTGACAGCCGTCATCCCAGAGTGAGAGCATGCAGAGCTGGGGCCTCAGGGAGGTGGTTAGGGCCTGAGGGGGA

< S’alpha_1 S’alpha_2 >

< S’mu_1 S’mu_2 >

GACAGGACTGTGGAGACACCTTAGAAGGACAGATTCTGTTCCGAATCACCGATGCGGCGTCAGCAGGACTGGCCTAGCGGAGGCTCT

|||||||||||||||||||||||||||||||||||||||||||||||||||||||

GACAGGACTGTGGAGACACCTTAGAAGGACAGATTCTGTTCCGAATCACCGATGCAGAGTGAGAGCATGCAGAGCTGGGGCCTCGGGGAGGTGGTTAGGGCCTGAGGG

|||||||||||||||||||||||||||||| |||||||||||||||||||||||

GTGTGACAGCCGTCATCCCAGAGTGAGAGCATGCAGAGCTGGGGCCTCAGGGAGGTGGTTAGGGCCTGAGGG

< S’alpha_1 S’alpha_2 >

S’mu_2 >

GTTAGTTGGTGGCATAAGAGAAAACTCAATCAGATAGTGCTGAAGACAGGACTGTGGAGACA...49 nt...ACTGGCCTAGCGGAGGCTCT

||||||||||||||||||||||||||||||||||||||||||||||||||||||||

GTTAGTTGGTGGCATAAGAGAAAACTCAATCAGATAGTGCTGAAGACAGGACTGTGAAGCGCCTCTGGCCTCCCATTGCCGGTGCCACCCGAGCCCCCAGACATAG

|||||||||||||||||||||||||||||||||||||||||||||||||||||||

GTGTGACAGCCGTCATCCCA...589 nt...GCTCCTCTGTGAAGCGCCTCTGGCCTCCCATTGCCGGTGCCACCCGAGCCCCCAGACATAG

< S’alpha_1

< S’mu_1 S’mu_2 >

CTGTGGAGACACCTTAGAAGGACAGATTCTGTTCCGAATCACCGATGCGGCGTCAGCAGGACTGGCCTAGCGGAGGCTCT

|||||||||||||||||||||||||||||||||||||||||||||||||||||||

CTGTGGAGACACCTTAGAAGGACAGATTCTGTTCCGAATCACCGATGCGGCGTCAAAAATGTGACTCGGTCCCTGATCTCACTAGAGACCCAAAAGAAAAGGGACCC

|||||||||||| ||||||||| |||||||||||||||| |||||||||||||||

GTGTGACAGCCGTCATCCCA...53 nt...GAACCCTCAAAAATGTGATTCGGTCCCTAATCTCACTAGAGACCCTAAAGAAAAGGGACCC

< S’alpha_1

S’mu_2 >

ATGTGCATCATTATCCTGAATTATTTCAGTTAAGCATGTTAG...106 nt...ACTGGCCTAGCGGAGGCTCT

||||||||||||||||||||||||||||||||||||

ATGTGCATCATTATCCTGAATTATTTCAGTTAAGCAGGGGCCTCGGGGAGGTGGATCATCCCAGCAGAGCTGGGGCCTCGGGGAGGT

|||||||||||||||| |||||||

GTGTGACAGCCGTCATCCCAGAGTGAGAGCATGCAGAGCTGGGGCCTCAGGGAGGT

< S’alpha_1

< S’mu_1 S’mu_2 >

GAGACACCTTAGAAGGACAGATTCTGTTCCGAATCACCGATGCGGCGTCAGC-AGGACTGGCCTAGCGGAGGCTCT

|||||||||||||||||||||||||||||||||||||||||||||||||||| |||

GAGACACCTTAGAAGGACAGATTCTGTTCCGAATCACCGATGCGGCGTCAGCGAGGGGGAACACTCAAAAATGTGACTCGGTCCCTGATCTCACTAGAGACCCAAAAGA

|||||||||| ||||||||||||| ||||||||| |||||||||||||||| |||||

GTGTGACAGCCGTCATCCCA...41 nt...GGGCCTGAGGGGGAACCCTCAAAAATGTGATTCGGTCCCTAATCTCACTAGAGACCCTAAAGA

< S’alpha_1

< S’mu_1 S’mu_2 >

TAGAAGGACAGATTCTGTTCCGAATCACCGATGCGGCGTCAGCAGGACTGGCCTAGCGGAGGCTCT

|||||||||||||||||||||||||||||||||| |||||||||||

TAGAAGGACAGATTCTGTTCCGAATCACCGATGCAGCGTCAGCAGGGAGGGTGGCTGCCAGTCTATGGTATTTTTGTTATTGCAGCCAGAAATGACTAAGACGCAGCA

|||||||||||||||||||||||||||||||||||||||||||| ||||||

GTGTGACAGCCGTCATCCCA...271 nt...AACCCACCAGTCTATGGTATTTTTGTTATTGCAGCCAGAAATGACTAAGATGCAGCA

< S’alpha_1

S’mu_2 >

TTATTTCAGTTAAGCATGTTAGTTGGTGGCATAAGAGAAAACTCAATCAGATAGTGCTGAAG...66 nt...ACTGGCCTAGCGGAGGCTCT

||||||||||||||||||||||||||||||||||||||||||||||||||| ||||

TTATTTCAGTTAAGCATGTTAGTTGGTGGCATAAGAGAAAACTCAATCAGAGAGTGAGAGCATGCAGAGCTGGGGCCTCGGGGAGGTGGTTAGGGCCTGAGGG

||||||||||||||||||||||||||||| |||||||||||||||||||||||

GTGTGACAGCCGTCATCCCAGAGTGAGAGCATGCAGAGCTGGGGCCTCAGGGAGGTGGTTAGGGCCTGAGGG

< S’alpha_1 S’alpha_2 >

< S’mu_1 S’mu_2 >

AGGACTGTGGAGACACCTTAGAAGGACAGATTCTGTTCCGAATCACCGATGCGGCGTCAGCAGGACTGGCCTAGCGGAGGCTCT

||||||||||||||||||||||||||||||||||||||||||||||||||||

AGGACTGTGGAGACACCTTAGAAGGACAGATTCTGTTCCGAATCACCGATGCCCAGAGTGAGAGCATGCAGAGCTGGGGCCTCAGGGAGGTGGTTAGGGCCTGA

|||||||||||||||||||||||||||||||||||||||||||||||||||||

GTGTGACAGCCGTCATCCCAGAGTGAGAGCATGCAGAGCTGGGGCCTCAGGGAGGTGGTTAGGGCCTGA

< S’alpha_1 S’alpha_2 >

< S’mu_1 S’mu_2 >

TGCTGAAGACAGGACTGTGGAGACACCTTAGAAGGACAGATTCTGTTCCGAATCACCGATGCGGCGTCAGCAGGACTGGCCTAGCGGAGGCTCT

|||||||||||||||||||||||||||||||||||||||||||||||||||||||||

TGCTGAAGACAGGACTGTGGAGACACCTTAGAAGGACAGATTCTGTTCCGAATCACCTGAGGGGGAACACTCAAAAATGTGACTCGGTCCCTGATCTCACTAGAGACCCAAA

||||||||||||| ||||||||||||| ||||||||| |||||||||||||||| ||

GTGTGACAGCCGTCATCCCA...38 nt...TTAGGGCCTGAGGGGGAACCCTCAAAAATGTGATTCGGTCCCTAATCTCACTAGAGACCCTAA

< S’alpha_1

S’mu_2 >

TCGATACCTCAGAGCATTATTTTCATAATGACTGTGTTCACAATCTTTTTAGGTTAACTC...263 nt...ACTGGCCTAGCGGAGGCTCT

||||||||||||||||||||||||||||||||||||||||||||||||||||||

TCGATACCTCAGAGCATTATTTTCATAATGACTGTGTTCACAATCTTTTTAGGTCCCTAATCTCACTAGGGACCCTAAAGAGAAGGGACCCCAGAGAGCAGCCCTGCC

|||||||||||||||||| ||||||||||| ||||||||||||||||||||||||||

GTGTGACAGCCGTCATCCCA...68 nt...TGATTCGGTCCCTAATCTCACTAGAGACCCTAAAGAAAAGGGACCCCAGAGAGCAGCCCTGCC

< S’alpha_1

S’mu_2 >

GCATGTTAGTTGGTGGCATAAGAGAAAACTCAATCAGATAGTGCTGAAGACAGGACTGTGGAGA...51 nt...ACTGGCCTAGCGGAGGCTCT

||||||||||||||||||||||||||||||||||||||||||||||||||||||||||

GCATGTTAGTTGGTGGCATAAGAGAAAACTCAATCAGATAGTGCTGAAGACAGGACTGCCCTCTTTCCACCATGTGAGGACGCAGCGAAAAGATGGGGGTCTGTGAACCAGG

||||||||||||||||| |||||||||||| ||||||||||||||||||||||||||

GTGTGACAGCCGTCATCCCA...120 nt...GCAGCCCTGCCCTCTTTCCACCACGTGAGGACGCAGTGAAAAGATGGGGGTCTGTGAACCAGG

< S’alpha_1

S’mu_2 >

TGGTGGCATAAGAGAAAACTCAATCAGATAGTGCTGAAGACAGGACTGTGGAGAC...50 nt...ACTGGCCTAGCGGAGGCTCT

||| |||||||||||||||||||||||||||||||||||||||||||||

TGGCGGCATAAGAGAAAACTCAATCAGATAGTGCTGAAGACAGGACTGT[insert]TGTGATTCGGTCCCTAATCTCACTAGAGACCCTAAAGAAAAGGGACCC

||||||||||||||||||||||||||||||||||||||||||||||||

GTGTGACAGCCGTCATCCCA...60 nt...CAAAAATGTGATTCGGTCCCTAATCTCACTAGAGACCCTAAAGAAAAGGGACCC

< S’alpha_1

[insert]: CATGGCTATCAGGGGTGGCGGGGCCGTGGTGAGGCCTCAGGTCTTTGTCCAAGGCTGCTGGGGCTGTCCTTCTAAGG

< S’mu_1 S’mu_2 >

CTGTGGAGACACCTTAGAAGGACAGATTCTGTTCCGAATCACCGATGCGGCGTCAGCAGGACTGGCCTAGCGGAGGCTCT

|||||||||||||||||||||||||||||||||||||||||||||||

CTGTGGAGACACCTTAGAAGGACAGATTCTGTTCCGAATCACCGATGACCCTCAAAAATGTGATTCGGTCCCTATTCTCACTAGAGACCCTAAAGAAAAGG

||||||||||||||||||||||||||| ||||||||||||||||||||||||||

GTGTGACAGCCGTCATCCCA...49 nt...GGGGGAACCCTCAAAAATGTGATTCGGTCCCTAATCTCACTAGAGACCCTAAAGAAAAGG

< S’alpha_1

< S’mu_1 S’mu_2 >

CCTTAGAAGGACAGATTCTGTTCCGAATCACCGATGCGGCGTCAGCAGGACTGGCCTAGCGGAGGCTCT

||||||||||||||||||||||||||||||||||||||||||||||||||||||| |||

CCTTAGAAGGACAGATTCTGTTCCGAATCACCGATGCGGCGTCAGCAGGACTGGC-TAGGGCCTGAGGGGGAACCCTCAAAAATGTGATTCGGTCCCTAATCTC

||| |||||||||||||||||||||||||||||||||||||||||||||||||

GTGTGACAGCCGTCATCCCA...29 nt...GGGAGGTGGT-TAGGGCCTGAGGGGGAACCCTCAAAAATGTGATTCGGTCCCTAATCTC

< S’alpha_1 S’alpha_2 >

< S’mu_1 S’mu_2 >

GACTGTGGAGACACCTTAGAAGGACAGATTCTGTTCCGAATCACCGATGCGGCGTCAGCAGGACTGGCCTAGCGGAGGCTCT

|||||||||||||||||||||||||||||||||||| ||||||||||||||||||

GACTGTGGAGACACCTTAGAAGGACAGATTCTGTTCTGAATCACCGATGCGGCGTGACAGCCGTCATCCCAGAGTGAGAGCATGCAGAGCTGGGGCCTCAGGGAGG

|||||||||||||||||||||||||||||||||||||||||||||||||||||

GTGTGACAGCCGTCATCCCAGAGTGAGAGCATGCAGAGCTGGGGCCTCAGGGAGG

< S’alpha_1

< S’mu_1 S’mu_2 >

TAGAAGGACAGATTCTGTTCCGAATCACCGATGCGGCGTCAGCAGGACTGGCCTAGCGGAGGCTCT

|||||||||||||||||||||||||||||||||||||||||||||||| |||||

TAGAAGGACAGATTCTGTTCCGAATCACCGATGCGGCGTCAGCAGGACCGGCCTGTGGTTAGGGCCTGAGGGGGAACCCTCAAAAATGTGATTCGGTCCCTAATCTCACT

||||||||||||||||||||||||||||||||||||||||||||||||||||||||

GTGTGACAGCCGTCATCCCAGAGTGAGAGCATGCAGAGCTGGGGCCTCAGGGAGGTGGTTAGGGCCTGAGGGGGAACCCTCAAAAATGTGATTCGGTCCCTAATCTCACT

< S’alpha_1 S’alpha_2 >

< S’mu_1 S’mu_2 >

AGACAGGACTGTGGAGACACCTTAGAAGGACAGATTCTGTTCCGAATCACCGATGCGGCGTCAGCAGGACTGGCCTAGCGGAGGCTCT

||||||||||||||||||||||||||||||||||||||||||||||||||||||||

AGACAGGACTGTGGAGACACCTTAGAAGGACAGATTCTGTTCCGAATCACCGATGCAGCTGGGGCCTCGGGGAGGTGGTTAGGGCCTGAGGGGGAACACTCAAAAATGTGA

|||||||||||| |||||||||||||||||||||||||||| |||||||||||||

GTGTGACAGCCGTCATCCCAGAGTGAGAGCATGCAGAGCTGGGGCCTCAGGGAGGTGGTTAGGGCCTGAGGGGGAACCCTCAAAAATGTGA

< S’alpha_1 S’alpha_2 >

< S’mu_1 S’mu_2 >

TCTGTTCCGAATCACCGATGCGGCGTCAGCAGGACTGGCCTAGCGGAGGCTCT

||||||||||||||||||||||||||||||||||||||||||||||||

TCTGTTCCGAATCACCGATGCGGCGTCAGCAGGACTGGCCTAGCGGAGCTGAGGGGGAACACTCAAAAATGTGACTCGGTCCCTGATCTCACTAGAGACCC

|||||||||||| ||||||||||||| ||||||||| ||||||||||||||||

GTGTGACAGCCGTCATCCCA...39 nt...TAGGGCCTGAGGGGGAACCCTCAAAAATGTGATTCGGTCCCTAATCTCACTAGAGACCC

< S’alpha_1

< S’mu_1 S’mu_2 >

ACCTTAGAAGGACAGATTCTGTTCCGAATCACCGATGCGGCGTCAGCAGGACTGGCCTAGCGGAGGCTCT

|||||||||||||||

ACCTTAGAAGGACAGGGCTGCTCTCTGGGGTCCCTTTTCTTTAGGGTCTCTAGTGAGATTAGGGACCGAATCACATCTCTCCAACGCAACGGCCAGCTCTCT

||||||||||||||||||||||||||||

GTGTGACAGCCGTCATCCCA...683 nt...GGCGGGATCTCTCCAACGCAACGGCCAGCTCTCT

< S’alpha_1

S’mu_2 >

TAAACTGCATTCATTTTTAAAGTAAGATGTTTAAGAAATTAAACAGTCTTAGGGAGAGTTTATG...392 nt...ACTGGCCTAGCGGAGGCTCT

||||||||||||||||||||||||||||||||||||||||||||||||||||||||||

TAAACTGCATTCATTTTTAAAGTAAGATGTTTAAGAAATTAAACAGTCTTAGGGAGAGCATGCAGAGCTGGGGCCTCAGGGAGGTGGTT

||||||||||||||||||||||||||||||||||||

GTGTGACAGCCGTCATCCCAGAGTGAGAGCATGCAGAGCTGGGGCCTCAGGGAGGTGGTT

< S’alpha_1

**Ramos, S’μ-S’α junctions from 3’DSBs, short overhangs (Cas9 N863A, S’μ_1 + S’μ_2 + S’α_1 + S’α_2)**

< S’mu_1 S’mu_2 >

GGAGACACCTTAGAAGGACAGATTCTGTTCCGAATCACCGATGCGGCGTCAGCAGGACTGGCCTAGCGGAGGCTCT

||||||||||||||||||||||||||||||||||||||||||||||||||||||||

GGAGACACCTTAGAAGGACAGATTCTGTTCCGAATCACCGATGCGGCGTCAGCAGGTGTGACTCGGTCCCTGATCTCACTAGAGACCCAAAAGAAAAGGGACCCCTGAGA

||||| ||||||||| |||||||||||||||| |||||||||||||||| ||||

GTGTGACAGCCGTCATCCCA...60 nt...CAAAAATGTGATTCGGTCCCTAATCTCACTAGAGACCCTAAAGAAAAGGGACCCCAGAGA

< S’alpha_1

< S’mu_1 S’mu_2 >

TGGAGACACCTTAGAAGGACAGATTCTGTTCCGAATCACCGATGCGGCGTCAGCAGGACTGGCCTAGCGGAGGCTCT

||||||||||||||||||||||||||||||||||||||||||||||||||||||||

TGGAGACACCTTAGAAGGACAGATTCTGTTCCGAATCACCGATGCGGCGTCAGCAGGACAGCCGTCATCCCAGAGTGAGAGCATGCAGAGCTGGGGCCTC

||||||||||||||||||||||||||||||||||||||||||||

GTGTGACAGCCGTCATCCCAGAGTGAGAGCATGCAGAGCTGGGGCCTC

< S’alpha_1

< S’mu_1 S’mu_2 >

AGACACCTTAGAAGGACAGATTCTGTTCCGAATCACCGATGCGGCGTCAGCAGGACTGGCCTAGCGGAGGCTCT

|||||||||||||||||||||||||||||||||||||||||||||||||||||||

AGACACCTTAGAAGGACAGATTCTGTTCCGAATCACCGATGCGGCGTCAGCAGGAGTGAGAGCATGCAGAGCTGGGGCCTCGGGGAGGTGGTTAGGGCCTGAGGGGGA

|||||||||||||||||||||||||||| ||||||||||||||||||||||||||

GTGTGACAGCCGTCATCCCAGAGTGAGAGCATGCAGAGCTGGGGCCTCAGGGAGGTGGTTAGGGCCTGAGGGGGA

< S’alpha_1 S’alpha_2 >

< S’mu_1 S’mu_2 >

AGACACCTTAGAAGGACAGATTCTGTTCCGAATCACCGATGCGGCGTCAGCAGGACTGGCCTAGCGGAGGCTCT

||||||||||||||||||||||||||||||||||||||||||||||||||||||||||

AGACACCTTAGAAGGACAGATTCTGTTCCGAATCACCGATGCGGCGTCAGCAGGACTGTTAGGGCCTGAGGGGGAACACTCAAAAATGTGACTCGGTCCCTGATCTCACTAGAG

|||||||||||||||||||| ||||||||||||| ||||||||| ||||||||||||

GTGTGACAGCCGTCATCCCAGAGTGAGAGCATGCAGAGCTGGGGCCTCAGGGAGGTGGTTAGGGCCTGAGGGGGAACCCTCAAAAATGTGATTCGGTCCCTAATCTCACTAGAG

< S’alpha_1 S’alpha_2 >

< S’mu_1 S’mu_2 >

CACCTTAGAAGGACAGATTCTGTTCCGAATCACCGATGCGGCGTCAGCAGGACTGGCCTAGCGGAGGCTCT

|||||||||||||||||||||||||||||||||| ||||||||||||||||||||

CACCTTAGAAGGACAGATTCTGTTCCGAATCACCTATGCGGCGTCAGCAGGACTGAGGGCCGGGGGCATCCCAAGGTTCATAACAAAAGCTCCAGAGCTTCCCTGAAC

||||||||||||||||||||||||||||||||||||||||||||||||||||||

GTGTGACAGCCGTCATCCCA...338 nt...CCTGGCGAGGGCCGGGGGCATCCCAAGGTTCATAACAAAAGCTCCAGAGCTTCCCTGAAC

< S’alpha_1

< S’mu_1 S’mu_2 >

AAGGACAGATTCTGTTCCGAATCACCGATGCGGCGTCAGCAGGACTGGCCTAGCGGAGGCTCT

||||||||||||||||||||||||||||||||||||| |||||||||||||||||

AAGGACAGATTCTGTTCCGAATCACCGATGCGGCGTCMKCAGGACTGGCCTAGCGGCATCCCASAGTGAGAGCATGCASAGCTGGGGCCTCGGGSASGWGG

||||||| |||||||||||||| |||||||||||| || | | ||

GTGTGACAGCCGTCATCCCAGAGTGAGAGCATGCAGAGCTGGGGCCTCAGGGAGGTGG

< S’alpha_1

< S’mu_1 S’mu_2 >

GACAGATTCTGTTCCGAATCACCGATGCGGCGTCAGCAGGACTGGCCTAGCGGAGGCTCT

||||||||||||||||||||||||||||||||||||||||||||||||||

GACAGATTCTGTTCCGAATCACCGATGCGGCGTCAGCAGGACTGGCCTAG[insert]AGTGAGAGCATGCAGAGCTGGGGCCTCAGGGAGGTGGTTAG

|||||||||||||||||||||||||||||||||||||||||

GTGTGACAGCCGTCATCCCAGAGTGAGAGCATGCAGAGCTGGGGCCTCAGGGAGGTGGTTAG

< S’alpha_1 S’alpha_2 >

[insert]: TGAGAGCATGCAGAGCTGGGGCGTCAGGGAGGTGGTTAGCAGGACTGGCCT

< S’mu_1 S’mu_2 >

GTGGAGACA-CTTAGAAGGACAGATTCTGTTCCGAATCACCGATGCGGCGTCAGCAGGACTGGCCTAGCGGAGGCTCT

||||||||| |||||||||||||||||||||||||||||||||||||||||||||||

GTGGAGACACCTTAGAAGGACAGATTCTGTTCCGAATCACCGATGCGGCGTCAGCAGCCGTCATCCCAGAGTGAGAGCATGCAGAGCTGGGGCCTCGGGGAGGTGGTTAG

|||||||||||||||||||||||||||||||||||||||||| |||||||||||||

GTGTGACAGCCGTCATCCCAGAGTGAGAGCATGCAGAGCTGGGGCCTCAGGGAGGTGGTTAG

< S’alpha_1

< S’mu_1 S’mu_2 >

TTAGAAGGACAGATTCTGTTCCGAATCACCGATGCGGCGTCAGCAGGACTGGCCTAGCGGAGGCTCT

||||||||||||||||||||||||||||||||||||||||||||||||||||||||||

TTAGAAGGACAGATTCTGTTCCGAATCACCGATGCGGCGTCAGCAGGACTGGCCTAGCTGACAGCCGTCATCCCAGAGTGAGAGCATGCAGAGCTGGGGCCTC

|||||||||||||||||||||||||||||||||||||||||||||

GTGTGACAGCCGTCATCCCAGAGTGAGAGCATGCAGAGCTGGGGCCTC

< S’alpha_1

< S’mu_1 S’mu_2 >

AGATTCTGTTCCGAATCACCGATGCGGCGTCAGCAGGACTGGCCTAGCGGAGGCTCT

||||||||||||||||||||||||||||||||||||||||||||||||||||||

AGATTCTGTTCCGAATCACCGATGCGGCGTCAGCAGGACTGGCCTAGCGGAGGCCT-AGCGGAGGCGGTTAGGGCCTGAGGGGGAACACTCAAAAATGTGACTCGG

||||| || ||||| ||||||||||||||||||||| ||||||||||||| ||||

GTGTGACAGCCGTCATCCCAGAGTGAGAGCATGCAGAGCTGGGGCCTCAG-GGAGGTGGTTAGGGCCTGAGGGGGAACCCTCAAAAATGTGATTCGG

< S’alpha_1 S’alpha_2 >

< S’mu_1 S’mu_2 >

TAGTGCTGAAGACAGGACTGTGGAGACACCTTAGAAGGACAGATTCTGTTCCGAATCACCGATGCGGCGTCAGCAGGACTGGCCTAGCGGAGGCTCT

||||||||||||||||||||||||||||||||||||||||||||||||||||||||

TAGTGCTGAAGACAGGACTGTGGAGACACCTTAGAAGGACAGATTCTGTTCCGAATTAGGGCCTGAGGGGGAACACTCAAAAATGTGACTCGGTCCCTGATCTCACTAG

||||||||||||||||||| ||||||||||||| ||||||||| ||||||||||

GTGTGACAGCCGTCATCCCA...32 nt...AGGTGGTTAGGGCCTGAGGGGGAACCCTCAAAAATGTGATTCGGTCCCTAATCTCACTAG

< S’alpha_1

< S’mu_1 S’mu_2 >

CAGGACTGTGGAGACACCTTAGAAGGACAGATTCTGTTCCGAATCACCGATGCGGCGTCAGCAGGACTGGCCTAGCGGAGGCTCT

|||||||||||||||||||||||||||||||||||||||||||||||||||||||||

CAGGACTGTGGAGACACCTTAGAAGGACAGATTCTGTTCCGAATCACCGATGCGGCGGAGAGCATGCAGAGCTGGGGCCTCAGGGAGGTGGTTAGGGCCTGAGGGGGAACCCT

||||||||||||||||||||||||||||||||||||||||||||||||||||||||

GTGTGACAGCCGTCATCCCAGAGTGAGAGCATGCAGAGCTGGGGCCTCAGGGAGGTGGTTAGGGCCTGAGGGGGAACCCT

< S’alpha_1 S’alpha_2 >

< S’mu_1 S’mu_2 >

CAGATTCTGTTCCGAATCACCGATGCGGCGTCAGCAGGACTGGCCTAGCGGAGGCTCT

|||||||||||||||||||||||||||||||||||||||||||||||| |

CAGATTCTGTTCCGAATCACCGATGCGGCGTCAGCAGGACTGGCCTAGTGGAGTGAGAGCATGCAGAGCTGGGGCCTCAGGGAGGTGGTTAGGGC

|||||||||||||||||||||||||||||||||||||||||||||

GTGTGACAGCCGTCATCCCAGAGTGAGAGCATGCAGAGCTGGGGCCTCAGGGAGGTGGTTAGGGC

< S’alpha_1 S’alpha_2 >

< S’mu_1 S’mu_2 >

TGCTGAAGACAGGACTGTGGAGACACCTTAGAAGGACAGATTCTGTTCCGAATCACCGATGCGGCGTCAGCAGGACTGGCCTAGCGGAGGCTCT

||||||||||||||||||||||||||||||||||||||||||||||||||||||||

TGCTGAAGACAGGACTGTGGAGACACCTTAGAAGGACAGATTCTGTTCCGAATCACAGAGTGAGAGCATGCAGAGCTGGGGCCTCGGGGAGGTGGTTAGGGCCTGAG

||| |||||||||||||||||||||||||||||| |||||||||||||||||||||

GTGTGACAGCCGTCATCCCAGAGTGAGAGCATGCAGAGCTGGGGCCTCAGGGAGGTGGTTAGGGCCTGAG

< S’alpha_1 S’alpha_2 >

< S’mu_1 S’mu_2 >

CTTAGAAGGACAGATTCTGTTCCGAATCACCGATGCGGCGTCAGCAGGACTGGCCTAGCGGAGGCTCT

||||||||||| |||||||||||||| ||||||||||||||||||||||||||||

CTTAGAAGGACTGATTCTGTTCCGAACCACCGATGCGGCGTCAGCAGGACTGGCCCAGAGTGAGAGCATGCAGAGCTGGGGCCTCAGGGAGGTGGTTAGGGCCT

|||||||||||||||||||||||||||||||||||||||||||||||||||

GTGTGACAGCCGTCATCCCAGAGTGAGAGCATGCAGAGCTGGGGCCTCAGGGAGGTGGTTAGGGCCT

< S’alpha_1 S’alpha_2 >

S’mu_2 >

AAATGTGCATCATTATCCTGAATTATTTCAGTTAAGCATGTTAGTTGGTGGCATAAGAGAAAAC...86 nt...ACTGGCCTAGCGGAGGCTCT

||||||||||||||||||||||||||||||||||||||||||||||||||||||||||

AAATGTGCATCATTATCCTGAATTATTTCAGTTAAGCATGTTAGTTGGTGGCATAAGATAAGGGACCCCAGAGAGCAGCCCTGCCCTCTTTCCACCATGTGAGGACGC

|||| |||||||||||||||||||||||||||||||||||||| ||||||||||

GTGTGACAGCCGTCATCCCA...94 nt...ACCCTAAAGAAAAGGGACCCCAGAGAGCAGCCCTGCCCTCTTTCCACCACGTGAGGACGC

< S’alpha_1

< S’mu_1 S’mu_2 >

AGATTCTGTTCCGAATCACCGATGCGGCGTCAGCAGGACTGGCCTAGCGGAGGCTCT

|||||||||||||||||||||||||||||||| |||||||||||||||||||

AGATTCTGTTCCGAATCACCGATGCGGCGTCAACAGGACTGGCCTAGCGGAGGCCGTCATCCCAGAGTGAGAGCATGCAGAGCTGGGGCCTCGGGGAGGTGGTTAGG

|||||||||||||||||||||||||||||||||||||||| ||||||||||||||

GTGTGACAGCCGTCATCCCAGAGTGAGAGCATGCAGAGCTGGGGCCTCAGGGAGGTGGTTAGG

< S’alpha_1 S’alpha_2 >

< S’mu_1 S’mu_2 >

CTTAGAAGGACAGATTCTGTTCCGAATCACCGATGCGGCGTCAGCAGGACTGGCCTAGCGGAGGCTCT

||||||||||||||||||||||||||||||||||||||||||||||||||||||||

CTTAGAAGGACAGATTCTGTTCCGAATCACCGATGCGGCGTCAGCAGGACTGGCCTAGCCGTCATCCCAGAGTGAGAGCATGCAGAGCTGGGGCCTCAGGGAGGTGGTT

|||||||||||||||||||||||||||||||||||||||||||||||||||||

GTGTGACAGCCGTCATCCCAGAGTGAGAGCATGCAGAGCTGGGGCCTCAGGGAGGTGGTT

< S’alpha_1 S’alpha_2 >

< S’mu_1 S’mu_2 >

AGACACCTTAGAAGGACAGATTCTGTTCCGAATCACCGATGCGGCGTCAGCAGGACTGGCCTAGCGGAGGCTCT

||||||||||||||||||||||||||||||||||||||||||||||||||||||

AGACACCTTAGAAGGACAGATTCTGTTCCGAATCACCGATGCGGCGTCAGCAGGTGTGACTCGGTCCCTGATCTCACTAGAGACCCAAAAGAAAAGGGACCCCAGAGAGC

||||| ||||||||| |||||||||||||||| |||||||||||||||||||||||

GTGTGACAGCCGTCATCCCA...60 nt...CAAAAATGTGATTCGGTCCCTAATCTCACTAGAGACCCTAAAGAAAAGGGACCCCAGAGAGC

< S’alpha_1

< S’mu_1 S’mu_2 >

GACAGATTCTGTTCCGAATCACCGATGCGGCGTCAGCAGGACTGGCCTAGCGGAGGCTCT

|||||||||||||||||||||||||||||||||||||||||| |||||||

GACAGATTCTGTTCCGAATCACCGATGCGGCGTCAGCAGGACCGGCCTAGAGCATGCAGAGCTGGGGCCTCGGGGAGGTGGTTAGGGCCTGAGGGGGAAC

||||||||||||||||||||||| ||||||||||||||||||||||||||||

GTGTGACAGCCGTCATCCCAGAGTGAGAGCATGCAGAGCTGGGGCCTCAGGGAGGTGGTTAGGGCCTGAGGGGGAAC

< S’alpha_1 S’alpha_2 >

< S’mu_1 S’mu_2 >

CTTAGAAGGACAGATTCTGTTCCGAATCACCGATGCGGCGTCAGCAGGACTGGCCTAGCGGAGGCTCT

|||||||||||||||||||||||||||||||||||||||||||||||||||||||

CTTAGAAGGACAGATTCTGTTCCGAATCACCGATGCGGCGTCAGCAGGACTGGCCTCATCCCAGAGTGAGAGCATGCAGAGCTGGGGCCTCGGGGAGGTGGTT

|||||||||||||||||||||||||||||||||||| |||||||||||

GTGTGACAGCCGTCATCCCAGAGTGAGAGCATGCAGAGCTGGGGCCTCAGGGAGGTGGTT

< S’alpha_1

< S’mu_1 S’mu_2 >

TGTGGAGACACCTTAGAAGGACAGATTCTGTTCCGAATCACCGATGCGGCGTCAGCAGGACTGGCCTAGCGGAGGCTCT

||||||||||||||||||||||||||||||||||||||||||||||||||||||||||

TGTGGAGACACCTTAGAAGGACAGATTCTGTTCCGAATCACCGATGCGGCGTCAGCAGCCACGTCCCCCTGGCGAGGGCCGGGGGCATCCCAAGGTTCATAACA

||||||||||||||||||||||||||||||||||||||||||||||||||||

GTGTGACAGCCGTCATCCCA...317 nt...AAGATGCAGCAGCCACGTCCCCCTGGCGAGGGCCGGGGGCATCCCAAGGTTCATAACA

< S’alpha_1

S’mu_2 >

TATTTTTCAAGACCACTTTTCAACTACTCA...171 nt...ACTGGCCTAGCGGAGGCTCT

||||||||||||||||||||||||

TATTTTTCAAGACCACTTTTCAACATCCCAGAGTGAGAGCATGCAGAGCTGGGGCCTCGGGGAGCATGCAGAGCTGGGGCCTC

||||||||||||||||||||||

GTGTGACAGCCGTCATCCCAGAGTGAGAGCATGCAGAGCTGGGGCCTC

< S’alpha_1

< S’mu_1 S’mu_2 >

AGACAGGACTGTGGAGACACCTTAGAAGGACAGATTCTGTTCCGAATCACCGATGCGGCGTCAGCAGGACTGGCCTAGCGGAGGCTCT

||||||||||||||||||||||||||||||||||||||||||||| |||||

AGACAGGACTGTGGAGACACCTTAGAAGGACAGATTCTGTTCCGAGTCACCTCGGGGAGGTGGTTAGGGCCTGAGGGGGAACACTCAAAAATGTGACTCGGTCCCT

|||| |||||||||||||||||||||||||||| ||||||||||||| |||||||||

GTGTGACAGCCGTCATCCCAGAGTGAGAGCATGCAGAGCTGGGGCCTCAGGGAGGTGGTTAGGGCCTGAGGGGGAACCCTCAAAAATGTGATTCGGTCCCT

< S’alpha_1 S’alpha_2 >

< S’mu_1 S’mu_2 >

TAGAAGGACAGATTCTGTTCCGAATCACCGATGCGGCGTCAGCAGGACTGGCCTAGCGGAGGCTCT

|||||||||||||||||||||||||||||||||||||||||||||||||||

TAGAAGGACAGATTCTGTTCCGAATCACCGATGCGGCGTCAGCAGGACTGGAGAGCATGCAGAGCTGGGGCCTCGGGGAGGTGGTTAGGGCCTGAGGGGGAAC

|||||||||||||||||||||||| ||||||||||||||||||||||||||||

GTGTGACAGCCGTCATCCCAGAGTGAGAGCATGCAGAGCTGGGGCCTCAGGGAGGTGGTTAGGGCCTGAGGGGGAAC

< S’alpha_1 S’alpha_2 >

S’mu_2 >

AGGATAAGTTTTAGGTAAAATGTGCATCATTATCCTGAATTATTTCAGTTAAGCATGTTAGTT...104 nt...ACTGGCCTAGCGGAGGCTCT

|||||||||||||||||||||||||||||||||||||||||||||||||||||||||

AGGATAAGTTTTAGGTAAAATGTGCATCATTATCCTGAATTATTTCAGTTAAGCATGCAGAGCTGGGGCCTCGGGGAGGTGGTTAGGGCCTGAGGGGGAACACTCAA

||||||||||||||||||||| |||||||||||||||||||||||||||| |||||

GTGTGACAGCCGTCATCCCAGAGTGAGAGCATGCAGAGCTGGGGCCTCAGGGAGGTGGTTAGGGCCTGAGGGGGAACCCTCAA

< S’alpha_1 S’alpha_2 >

< S’mu_1 S’mu_2 >

GAGACACCTTAGAAGGACAGATTCTGTTCCGAATCACCGATGCGGCGTCAGCAGGACTGGCCTAGCGGAGGCTCT

||||||||||||||||||||||||||||||||||||||||||||||||||||||

GAGACACCTTAGAAGGACAGATTCTGTTCCGAATCACCGATGCGGCGTCAGCAGGACAGCCGTCATCCCAGAGTGAGAGCATGCAGAGCTGGGGCCTC

||||||||||||||||||||||||||||||||||||||||||||

GTGTGACAGCCGTCATCCCAGAGTGAGAGCATGCAGAGCTGGGGCCTC

< S’alpha_1

< S’mu_1 S’mu_2 >

GAAGGACAGATTCTGTTCCGAATCACCGATGCGGCGTCAGCAGGACTGGCCTAGCGGAGGCTCT

||||||||||||||||||||||||||||||||||||||||||||||||||||||

GAAGGACAGATTCTGTTCCGAATCACCGATGCGGCGTCAGCAGGACTGGCCTAGAGTGAGAGCATGCAGAGCTGGGGCCTCAGGGAGGTGGTTAGGGCCTGAGGGGG

|||||||||||||||||||||||||||||||||||||||||||||||||||||||

GTGTGACAGCCGTCATCCCAGAGTGAGAGCATGCAGAGCTGGGGCCTCAGGGAGGTGGTTAGGGCCTGAGGGGG

< S’alpha_1 S’alpha_2 >

< S’mu_1 S’mu_2 >

CTGAAGACAGGACTGTGGAGACACCTTAGAAGGACAGATTCTGTTCCGAATCACCGATGCGGCGTCAGCAGGACTGGCCTAGCGGAGGCTCT

||||||||||||||||||||||| ||||||||||||||||||||||||||| |||

CTGAAGACAGGACTGTGGAGACA-CTTAGAAGGACAGATTCTGTTCCGAAT-ACC[insert]TGACAGCCGTCATCCCAGAGTGAGAGCATGCAGAGCTGGGGCCTCAGGGA

||||||||||||||||||||||||||||||||||||||||||||||||||

GTGTGACAGCCGTCATCCCAGAGTGAGAGCATGCAGAGCTGGGGCCTCAGGGA

< S’alpha_1

[insert]: AGAAAAGAGATTTTCTTGTCCCGCATGGAGCAGATTCTGCCATGGCAAAACATGGTGGAAGTCATCGAGCCGTTTTACCCCAAGGCTGGTAATGGCCGGCGAC

< S’mu_1 S’mu_2 >

TGTTCCGAATCACCGATGCGGCGTCAGCAGGACTGGCCTAGCGGAGGCTCT

||||||||||||||||||||||||||||||||||||||||||||||

TGTTCCGAATCACCGATGCGGCGTCAGCAGGACTGGCCTAGCGGAGTCAGCAGGACTAGAGTGAGAGCATGCAGAGCTGGGGCCTTGGGGAGGTGGTTAG

|||||||||||||||||||||||||||| |||||||||||||

GTGTGACAGCCGTCATCCCAGAGTGAGAGCATGCAGAGCTGGGGCCTCAGGGAGGTGGTTAG

< S’alpha_1 S’alpha_2 >

< S’mu_1 S’mu_2 >

GAAGGACAGATTCTGTTCCGAATCACCGATGCGGCGTCAGCAGGACTGGCCTAGCGGAGGCTCT

|||||||||||||||||||||||||||||||||||||||||||||||

GAAGGACAGATTCTGTTCCGAATCACCGATGCGGCGTCAGCAGGACTATCACTCCGTCATCCCAGAGTGAGAGCATGCAGAGCTGGGGCCTCGGGGAGGTGGTTAGGGC

||||||||||||||||||||||||||||||||||||||| ||||||||||||||||

GTGTGACAGCCGTCATCCCAGAGTGAGAGCATGCAGAGCTGGGGCCTCAGGGAGGTGGTTAGGGC

< S’alpha_1 S’alpha_2 >

< S’mu_1 S’mu_2 >

GACAGATTCTGTTCCGAATCACCGATGCGGCGTCAGCAGGACTGGCCTAGCGGAGGCTCT

|||||||||||||||||||||||||||||||||||||||||||||||||

GACAGATTCTGTTCCGAATCACCGATGCGGCGTCAGCAGGACTGGCCTAAGGGGGAACACTCAAAAATGTGGCTCGGTCCCTGATCTCACTAGAGACCC

||||| ||||||||| |||||||||||| ||||||||| ||||||||||||||||

GTGTGACAGCCGTCATCCCA...36 nt...GGTTAGGGCCTGAGGGGGAACCCTCAAAAATGTGATTCGGTCCCTAATCTCACTAGAGACCC

< S’alpha_1 S’alpha_2 >

S’mu_2 >

TAGGTAAAATGTGCATCATTATCCTGAATTATTTCAGTTAAGCATGTTAGTTGGTGGCATAAGAG...91 nt...ACTGGCCTAGCGGAGGCTCT

|||||||||||||||||||||||||||||||||||||||||||||||||||||||||||

TAGGTAAAATGTGCATCATTATCCTGAATTATTTCAGTTAAGCATGTTAGTTGGTGGCAGCCGTCATCCCAGAGTGAGAGCATGCAGAGCTGGGGCCTCAGGGAGGTGGTT

||| ||||||||||||||||||||||||||||||||||||||||||||||||||||||

GTGTGACAGCCGTCATCCCAGAGTGAGAGCATGCAGAGCTGGGGCCTCAGGGAGGTGGTT

< S’alpha_1 S’alpha_2 >

< S’mu_1 S’mu_2 >

GAAGGACAGATTCTGTTCCGAATCACCGATGCGGCGTCAGCAGGACTGGCCTAGCGGAGGCTCT

||||||||||||||||||||||||||||||||||||||||||||||

GAAGGACAGATTCTGTTCCGAATCACCGATGCGGCGTCAGCAGGACAGCAGTCATCCCAGAGTGAGAGCATGCAGAGCTGGGGCCTC

||| |||||||||||||||||||||||||||||||||||||

GTGTGACAGCCGTCATCCCAGAGTGAGAGCATGCAGAGCTGGGGCCTC

< S’alpha_1

S’mu_2 >

TTATTTCAGTTAAGCATGTTAGTTGGTGGCATAAGAGAAAACTCAATCAGAT...76 nt...ACTGGCCTAGCGGAGGCTCT

||||||||||||||||||||||||||||||||||||||||||||||

TTATTTCAGTTAAGCATGTTAGTTGGTGGCATAAGAGAAAACTCAAGAGTGAGAGCATGCAGAGCTGGGGCCTCGGGGAGGTGGTTAGGGCCTGAGGGG

||||||||||||||||||||||||||||| ||||||||||||||||||||||||

GTGTGACAGCCGTCATCCCAGAGTGAGAGCATGCAGAGCTGGGGCCTCAGGGAGGTGGTTAGGGCCTGAGGGG

< S’alpha_1 S’alpha_2 >

< S’mu_1 S’mu_2 >

GAGACACCTTAGAAGGACAGATTCTGTTCCGAATCACCGATGCGGCGTCAGCAGGACTGGCCTAGCGGAGGCTCT

|||||||||||||||||||||||||||||||||||||||||||||||||||

GAGACACCTTAGAAGGACAGATTCTGTTCCGAATCACCGATGCGGCGTCAGAGAGAGCATGCGGAGCTGGGGCCTCGGGGAGGTGGTTAGGGCCTGAGGGGGAACACTC

|||||||||| ||||||||||||| |||||||||||||||||||||||||||| |||

GTGTGACAGCCGTCATCCCAGAGTGAGAGCATGCAGAGCTGGGGCCTCAGGGAGGTGGTTAGGGCCTGAGGGGGAACCCTC

< S’alpha_1 S’alpha_2 >

< S’mu_1 S’mu_2 >

AGACAGGACTGTGGAGACACCTTAGAAGGACAGATTCTGTTCCGAATCACCGATGCGGCGTCAGCAGGACTGGCCTAGCGGAGGCTCT

||||||| |||| |||||||||||||||||||||||||||||||||||||||

AGACAGGGCTGT-GAGACACCTTAGAAGGACAGATTCTGTTCCGAATCACCGTCATCCCAGAGTGAGAGCATGCAGAGCTGGGGCCTCGGGGAGGTGGTTAGGGC

||||||||||||||||||||||||||||||||||||||| ||||||||||||||||

GTGTGACAGCCGTCATCCCAGAGTGAGAGCATGCAGAGCTGGGGCCTCAGGGAGGTGGTTAGGGC

< S’alpha_1 S’alpha_2 >
